# Supramolecular Binding and Extraction of Phosphate, Phosphite and Fluorophosphate Anions from Water by Nanojars

**DOI:** 10.1021/acs.inorgchem.5c05810

**Published:** 2026-03-03

**Authors:** Wisam A. Al Isawi, Angel S. Philip, Pooja Singh, Matthias Zeller, Gellert Mezei

**Affiliations:** † Department of Chemistry, Western Michigan University, Kalamazoo, Michigan 49008, United States; ‡ Department of Chemistry, Purdue University, West Lafayette, Indiana 47907, United States

## Abstract

In this work, the supramolecular binding of HPO_4_^2–^, HPO_3_^2–^ and FPO_3_^2–^ ions by nanojars of the general formula [XPO_3_^2–^⊂{*cis*-Cu^II^(μ-OH)­(μ-pz)}_*n*_]^2–^ (**Cu**_***n***_ **XPO**_**3**_; X = HO, H, F; *n* = 27–33; pz = pyrazolate) was explored. The nanojar hosts, which consist of stacks of three Cu_*x*_ (*x* = 6–14, except 11) metallamacrocycles, were studied in solution by electrospray-ionization mass spectrometry, variable-temperature, paramagnetic ^1^H NMR and UV–vis spectroscopy, whereas the entrapped anion was probed using ^19^F and ^31^P NMR spectroscopy. In the solid state, X-ray diffraction on nine different single-crystals offers valuable information about the structure of the host–guest complexes (**Cu**_**7+13+9**_ **HPO**_**4**_, **Cu**_**8+13+8**_ **HPO**_**4**_, **Cu**_**8+13+8**_ **HPO**_**3**_, and three pseudopolymorphs each for **Cu**_**8+14+9**_ **HPO**_**3**_ and **Cu**_**8+14+9**_ **FPO**_**3**_) and details of the supramolecular binding of the different phosphorus anions. Multinuclear NMR studies reveal that the different XPO_3_^2–^ ions induce dramatic changes in the magnetism of a given nanojar, despite a less striking difference observed in their respective crystal structure, pointing to significantly different solution structures. The formation of HPO_3_^2–^ and HPO_4_^2–^ nanojars by anion exchange from CO_3_^2–^ was also studied, along with the effects of NH_3_ and Ba^2+^ ions on nanojar composition and thermal stability in solution. Furthermore, the liquid–liquid extraction of the HPO_4_^2–^, HPO_3_^2–^ and FPO_3_^2–^ anions from water into an organic solvent was demonstrated using nanojars as extractants.

## Graphical abstract

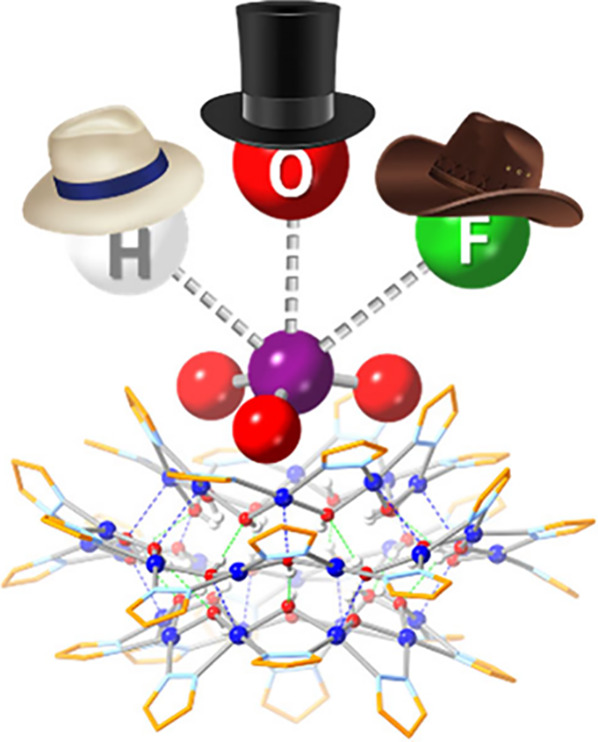

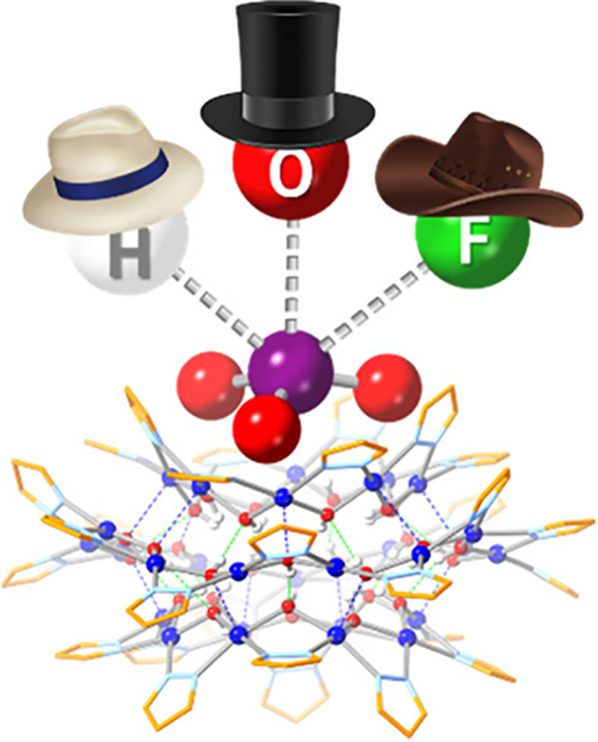

## Introduction

Phosphorus is the 11th most abundant element in Earth’s crust, and it is a vital constituent of every living cell.^1^ In nature, it is mostly found as phosphate, [PO_4_H_*n*_]^(3–*n*)–^ (*n* = 0–3), of which 65 million tons (in terms of P_2_O_5_ content) were commercially produced worldwide in 2024.^2^ Its vast majority (∼95%) is used as fertilizer for crops and animal feed supplements, and ultimately ends up in rivers, lakes and coastal waters where it contributes to the degradation of ecosystems and loss of biodiversity.^3^ Eutrophication is estimated to cost the US economy alone $2.2 billion annually.^3^ Another concern about phosphorus revolves around phosphate rock (apatite) resources, which are finite and are the only significant source of phosphate.^2,4^

Besides phosphate, in which the P atom is in its highest oxidation state (5+), lower oxidation state species, such as phosphite [HPO_3_H_*n*_]^(2–*n*)–^ (*n* = 0–2; oxidation state of P: +3) are also found in nature and play an important role in the biogeochemistry of phosphorus.^5–9^ To avoid confusion with RPO_3_^2–^ (R = organic group; oxidation state of P: +5), which are also called phosphonates, herein we will refer to HPO_3_^2–^ as phosphite instead of the IUPAC-recommended name of phosphonate. Under low phosphate concentrations, some bacteria are able to use phosphite as their phosphorus source.^10,11^ Phosphite originates both from natural sources, such as the reduction of phosphate by high-energy events,^12^ corrosion of phosphide minerals in meteorites,^13^ geothermal waters,^14^ and from anthropogenic sources, such as fungicides, fumigants, reducing agents used in electroless metal plating, and corrosion of cast iron and steel.^6,15,16^ Currently, there are no phosphite minerals known other than calcium phosphite (CaHPO_3_), which was recently discovered in fulgurites created by lightning strikes.^17^

Fluorophosphate (FPO_3_^2–^; oxidation state of P: +5) is a derivative of phosphate in which one of the OH groups is substituted by fluorine. While no fluorophosphate minerals have been identified in nature,^18^ it is used in dentrifices,^19,20^ anticorrosion agents,^21^ wood preservatives,^22^ scale control,^23^ glasses for optics and photonics,^24^ and novel battery materials.^25^ Amid the historical use of fluorophosphorus compounds as chemical weapons,^26^ there is a renewed interest in fluorophosphorus chemistry in synthesis, chemical biology and drug development.^27^

To mitigate both the environmental impact caused by phosphorus pollution and its limited supply, there is an increasing interest in recovering phosphorus from water bodies.^28–30^ Current phosphate removal methods, such as precipitation/crystallization, adsorption, membrane filtration and ion exchange suffer from disadvantages, especially when the recovery of phosphate is sought.^31,32^ These disadvantages include lack of selectivity and low product purity/bioavailability, desorption/regeneration issues, membrane fouling/high sludge volume, residual contamination, operational complexity and high cost. Therefore, alternative methods are also being pursued. Liquid–liquid extraction is an attractive approach, as it offers advantages such as selectivity and recyclability, and avoids the generation of large amounts of contaminated byproducts.^33,34^

While various supramolecular receptors for the binding of phosphate have been developed,^35–47^ much less efforts have been focused on the supramolecular binding of other phosphorus anions.^48–54^ Therefore, the objective of this work is to study the supramolecular binding and extraction from water of the HPO_3_^2–^ and FPO_3_^2–^ anions, for which no supramolecular receptors have yet been developed, by *nanojars* of the general formula [XPO_3_⊂{*cis*-Cu^II^(μ-OH)­(μ-pz)}_*n*_]^2–^ (**Cu**_***n***_ **XPO**_**3**_; X = H or F; pz = pyrazolate anion, C_3_H_3_N_2_^–^; *n* = 27–33). These nanojars are composed of three neutral {*cis*-Cu^II^(μ-OH)­(μ-pz)}_*m*_ metallamacrocycles (*m* = 6–14, except 11), which will be indicated, for example, as **Cu**_**8+13+8**_ **HPO**_**4**_ (Scheme 1). Another objective is to contrast the binding of HPO_3_^2–^ and FPO_3_^2–^ with the binding of HPO_4_^2–^ by similar **Cu**_***n***_ **XPO**_**3**_ (X = OH) nanojars. In the solid state, the binding of the anions within nanojar cavities were studied using single-crystal X-ray diffraction. Crystal structures (as Bu_4_N^+^ salts) of two structural isomers of **Cu**_**29**_ **HPO**_**4**_, **Cu**_**7+13+9**_ **HPO**_**4**_ (**1**) and **Cu**_**8+13+8**_ **HPO**_**4**_ (**2**), were analyzed, along with **Cu**_**8+13+8**_ **HPO**_**3**_ (**3**), three different pseudopolymorphs of **Cu**_**8+14+9**_ **HPO**_**3**_ (**4a**–**4c**), and the first crystal structures of a host–guest complex with supramolecularly entrapped fluorophosphate anion, **Cu**_**8+14+9**_ **FPO**_**3**_ (three different pseudopolymorphs, **5a**–**5c**). The solution structure of nanojars was explored using electrospray-ionization mass spectrometry (ESI-MS), variable-temperature (VT) ^1^H NMR and UV–vis spectroscopy, whereas the entrapped anion was probed in solution using ^19^F and ^31^P NMR spectroscopy. Anion exchange from CO_3_^2–^ to HPO_3_^2–^ or HPO_4_^2–^ was also studied in nanojars, along with chemical stability studies toward NH_3_ and Ba^2+^ ions to gauge the anion binding affinities of the different nanojars. Moreover, liquid–liquid extraction of the HPO_4_^2–^, HPO_3_^2–^ and FPO_3_^2–^ anions from water into an organic solvent using nanojars as extracting agents was demonstrated.

**Scheme 1:**
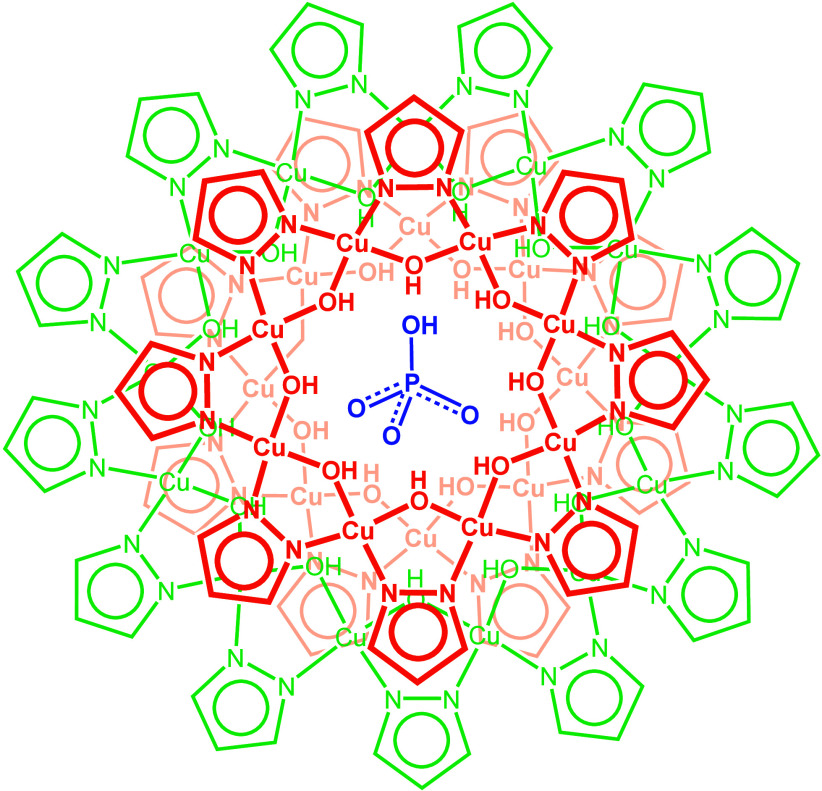
Schematic Representation of the Cu_29_ Nanojar, [HPO_4_⊂{Cu­(*μ*-OH)­(*μ*-pz)}_8+13+8_]^2–^ (**Cu**_**29**_ **HPO**_**4**_)­^a^

a Color code: persimmon, Cu_8_ rings; green, Cu_13_ ring.

## Results and Discussion

### Chemical Properties of HPO_3_^2–^ and FPO_3_^2–^

Because the phosphite and fluorophosphate anions are less well-known than phosphate, their properties pertinent to the work presented here will be discussed first. H_3_PO_3_ is a stronger acid (p*K*_a_: 1.3 and 6.70 at 20 °C) than H_3_PO_4_ (p*K*_a_: 2.16, 7.21, and 12.32 at 25 °C),^55^ with an inverted ratio of the di- and mononegative ions (HPO_3_^2–^/H_2_PO_3_^–^) of ∼60/40 at pH 7.^5^ Although the reduction of Cu^2+^ (Cu^2+^ + e^–^ → Cu^+^, *E*° = 0.153 V) by phosphite (HPO_3_^2–^ + H_2_O → HPO_4_^2–^ + 2H^+^ + 2e^–^, *E*° = 0.65 V; H_2_PO_3_^–^ + H_2_O → H_2_PO_4_^–^ + 2H^+^ + 2e^–^, *E*° = 0.37 V) at pH 7.0 is thermodynamically favorable (*E*_cell_^°^ = 0.80 or 0.52 V, depending on the protonation state), it does not readily occur in the absence of catalysts.^56^ In fact, aqueous solutions of phosphite can be stored unchanged for years under air.^57^ Moreover, Cu­(II) phosphite can be isolated and the crystal structure of CuHPO_3_·2H_2_O has been determined.^58^ The kinetic inertness of phosphite toward oxidation is due to the fact that the loss of electrons must be accompanied by the breaking of the P–H bond, which has a large activation energy of ∼370 kJ.^56^ No oxidation of phosphite has been observed during work with nanojars.

Fluorophosphoric acid (H_2_FPO_3_; p*K*_a_: 0.97 (predicted at 25 °C)^59^ and 4.8)^60^ undergoes hydrolysis in aqueous solution;^61,62^ indeed, ^19^F and ^31^P NMR spectra in DMSO-*d*_6_ of the commercial 70% solution in H_2_O indicate the presence of H_3_PO_4_, HF_2_PO_2_ and other hydrolysis products (Figures S1 and S2). Neutralization by 2 equiv of Bu_4_NOH (to obtain (Bu_4_N)_2_FPO_3_, which can be used as a DMSO-soluble reference) exacerbates hydrolysis (Figures S1 and S2).^61^ In contrast, the FPO_3_^2–^ ion obtained as Na_2_FPO_3_ by the fusion of solid NaF and NaPO_3_,^63^ is remarkably stable toward hydrolysis in neutral or moderately alkaline solutions.^64^ ^19^F and ^31^P NMR spectra of commercial Na_2_FPO_3_ in D_2_O (not soluble in DMSO-*d*_6_) confirm the absence of hydrolysis (Figure S3). Accordingly, no hydrolysis of the FPO_3_^2–^ ion was expected nor detected during the synthesis of nanojars with this anion in THF solution, in which both NaOH pellets and Na_2_FPO_3_ powder are insoluble.

### Synthesis and Mass Spectrometry

Nanojars containing supramolecularly bound HPO_3_^2–^, FPO_3_^2–^ and HPO_4_^2–^ ions were synthesized by self-assembly and their composition was analyzed by mass spectrometry. ESI-MS(−)­indicates that the reaction of Cu­(NO_3_)_2_, pyrazole, NaOH, Bu_4_NOH and Na_2_HPO_3_ in a 1:1:1.93:0.07:1 molar ratio in tetrahydrofuran (THF) produces a **Cu**_***n***_ **HPO**_**3**_ (*n* = 27–31) nanojar mixture which contains **Cu**_**27**_ **HPO**_**3**_ (*m*/*z* 2032.91), **Cu**_**29**_ **HPO**_**3**_ (*m*/*z* 2180.54) and **Cu**_**31**_ **HPO**_**3**_ (*m*/*z* 2328.16), and small amounts of **Cu**_**28**_ **HPO**_**3**_ (*m*/*z* 2106.73) and **Cu**_**30**_ **HPO**_**3**_ (*m*/*z* 2254.35) (Figure 1). A similar, clean **Cu**_***n***_ **FPO**_**3**_ (*n* = 27–29, 31) fluorophosphate-entrapping nanojar mixture was obtained using Na_2_FPO_3_ instead of Na_2_HPO_3_, which contains **Cu**_**27**_ **FPO**_**3**_ (*m*/*z* 2041.91), **Cu**_**29**_ **FPO**_**3**_ (*m*/*z* 2189.53) and **Cu**_**31**_ **FPO**_**3**_ (*m*/*z* 2337.16), along with a small amount of **Cu**_**28**_ **FPO**_**3**_ (*m*/*z* 2115.72) (Figure 1). An analogous reaction using Na_2_HPO_4_, however, did not produce clean **Cu**_***n***_ **HPO**_**4**_ nanojars. Instead, a mixture with **Cu**_***n***_ **CO**_**3**_ (*n* = 27, 29, 31) is obtained (Figure S6). Replacing Na_2_HPO_4_ with Na_3_PO_4_ only exacerbates the carbonate nanojar impurities whereas using Cu_2_(OH)­(PO_4_) as a combined copper and phosphate source offers only a slight improvement, pointing to a significant amount of carbonate in those reagents. Depolymerization of [*trans*-Cu^II^(OH)­(pz)]_∞_ by refluxing with *in situ* prepared (Bu_4_N)_2_HPO_4_ in toluene (bp 111 °C) does lead to carbonate-free **Cu**_***n***_ **HPO**_**4**_, although only the *n* = 29 and 31 species are observed (Figure S6). In turn, using (Bu_4_N)_2_HPO_4_ (prepared *in situ* from Bu_4_NOH and H_3_PO_4_) instead of Na_2_HPO_4_ allows for the preparation of a clean phosphate nanojar mixture by self-assembly in THF, composed mainly of **Cu**_**29**_ **HPO**_**4**_ (*m*/*z* 2188.54) and **Cu**_**31**_ **HPO**_**4**_ (*m*/*z* 2336.16), with small amounts of **Cu**_**27**_ **HPO**_**4**_ (*m*/*z* 2040.91), **Cu**_**30**_ **HPO**_**4**_ (*m*/*z* 2262.35), **Cu**_**32**_ **HPO**_**4**_ (*m*/*z* 2409.97) and **Cu**_**33**_ **HPO**_**4**_ (*m*/*z* 2483.78), and only a trace of **Cu**_**27**_ **CO**_**3**_ (Figure 1).

**Figure 1:**
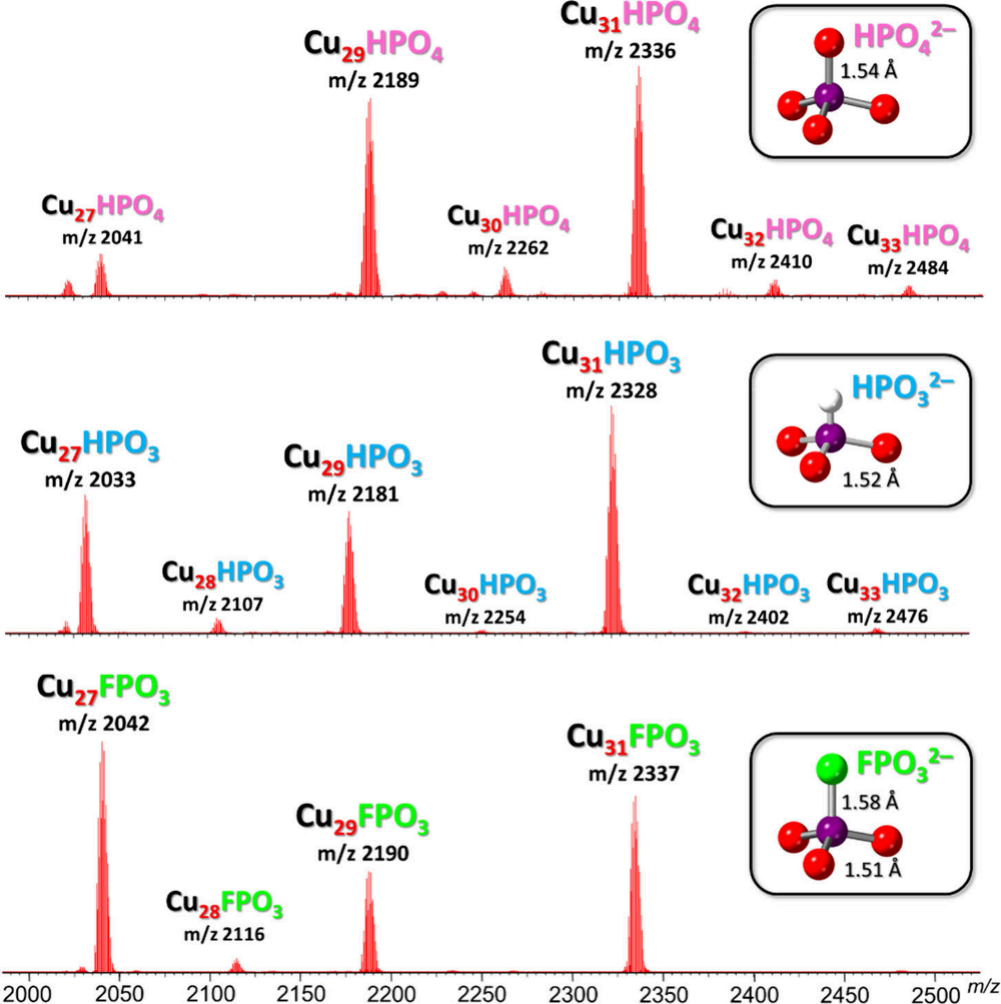
ESI-MS­(−) spectra (in CH_3_CN) of the phosphate nanojar mixture [HPO_4_⊂{Cu­(OH)­(pz)}_*n*_]^2–^ (**Cu**_***n***_ **HPO**_**4**_; *n* = 27–33) obtained using (Bu_4_N)_2_HPO_4_ and of the phosphite and fluorophosphate nanojar mixtures [XPO_3_⊂{Cu­(OH)­(pz)}_*n*_]^2–^ (**Cu**_***n***_ **XPO**_**3**_; X = H, F; *n* = 27–31) obtained using Na_2_XPO_3_. Detailed isotopic distributions are shown in Figures S4 and S5. Minor peaks correspond to adducts formed during ionization, [XPO_3_⊂{Cu_27_(OH)_27_(pz)_26_(HCOO)}]^2–^ (R = H, F; *m*/*z* 2021.89 and 2030.88), due to traces of formic acid in the mass spectrometer or to carbonate impurity (**Cu**_**27**_ **CO**_**3**_; *m*/*z* 2022.91) in the case of HPO_4_^2–^.

### Conversion of Cu_*n*_CO_3_ to Cu_*n*_HPO_3_ and Cu_*n*_HPO_4_ Nanojars by Anion Exchange

To explore alternative ways of nanojar synthesis and to test whether the CO_3_^2–^ ion (which is known to be strongly bound by nanojars),^65^ could be replaced by phosphorus oxoanions, anion exchange experiments were performed. First, the titration of carbonate nanojars **Cu**_***n***_ **CO**_**3**_ (*n* = 27, 29–31) with H_3_PO_3_ (1 to 100 equiv) was monitored by ESI-MS(−). Increasing amounts of the acid lead to a gradual and eventually complete transformation to phosphite nanojars, **Cu**_***n***_ **HPO**_**3**_ (*n* = 27, 29–31), which subsequently decompose at higher acidities (Figure 2). In the absence of any H_3_PO_3_, the following **Cu**_***n***_ **CO**_**3**_ species are observed in the carbonate nanojar solution: **Cu**_**27**_ **CO**_**3**_ (*m*/*z* 2022.91), **Cu**_**29**_ **CO**_**3**_ (*m*/*z* 2170.54), **Cu**_**30**_ **CO**_**3**_ (*m*/*z* 2244.61) and **Cu**_**31**_ **CO**_**3**_ (*m*/*z* 2318.16). At 1 equiv of H_3_PO_3_, the conversion of a fraction of **Cu**_**31**_ **CO**_**3**_ to **Cu**_**31**_ **HPO**_**3**_ (*m*/*z* 2328.16) is noted. At 2 equiv of H_3_PO_3_, the **Cu**_**30**_ **CO**_**3**_ and **Cu**_**31**_ **CO**_**3**_ species completely disappear and the amounts of **Cu**_**27**_ **CO**_**3**_ and **Cu**_**29**_ **CO**_**3**_ decrease, whereas the amount of **Cu**_**31**_ **HPO**_**3**_ increases. With increasing equivalents of H_3_PO_3_ added, the amounts of **Cu**_**27**_ **CO**_**3**_ and **Cu**_**29**_ **CO**_**3**_ decrease further, so that no **Cu**_**27**_ **CO**_**3**_ is present above 3 equiv of H_3_PO_3_ and no **Cu**_**29**_ **CO**_**3**_ is present above 7 equiv of H_3_PO_3_. Instead, small amounts of **Cu**_**27**_ **HPO**_**3**_ (*m*/*z* 2032.91), **Cu**_**29**_ **HPO**_**3**_ (*m*/*z* 2180.54) and **Cu**_**30**_ **HPO**_**3**_ (*m*/*z* 2254.35) are observed, along with small amounts of hitherto unidentified intermediate species (2– charged) at *m*/*z* 2142 and 2240. At 10 equiv of H_3_PO_3_, mostly **Cu**_**31**_ **HPO**_**3**_ and traces of **Cu**_**27**_ **HPO**_**3**_ and **Cu**_**29**_ **HPO**_**3**_ are observed, and only **Cu**_**31**_ **HPO**_**3**_ survives at 15 equiv of H_3_PO_3_. At 20 equiv of H_3_PO_3_ and above no nanojars are detected in solution, nor any soluble lower-nuclearity copper complexes. However, precipitates are observed in the samples with 15 or more equiv of H_3_PO_3_. The titration experiment reveals that H_3_PO_3_ protonates the CO_3_^2–^ ion in **Cu**_***n***_ **CO**_**3**_ to carbonic acid and replaces it with the HPO_3_^2–^ ion (**Cu**_***n***_ **CO**_**3**_ + H_3_PO_3_ → **Cu**_***n***_ **HPO**_**3**_ + CO_2_ + H_2_O), while the nanojars rearrange to the most favorable size for the new guest (**Cu**_**31**_ **HPO**_**3**_).

**Figure 2:**
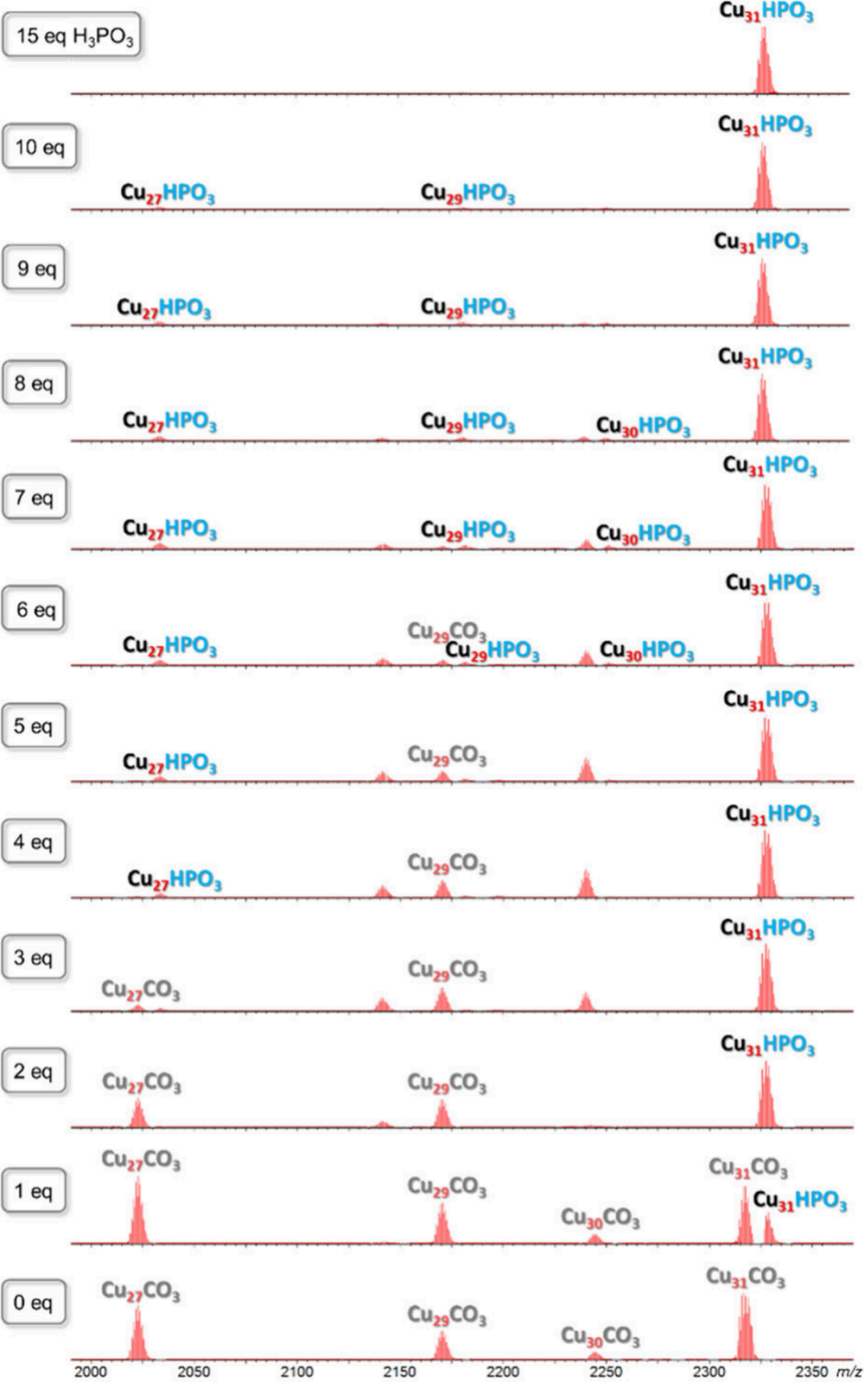
ESI-MS­(−) spectra in CH_3_CN of **Cu**_***n***_ **CO**_**3**_ (*n* = 27, 29–31) nanojars with varying amounts of added H_3_PO_3_.

The analogous titration of **Cu**_***n***_ **CO**_**3**_ with H_3_PO_4_ proceeds differently from the one with H_3_PO_3_ (Figure S7). With 1 equiv of H_3_PO_4_, **Cu**_**31**_ **HPO**_**4**_ (*m*/*z* 2336.16) begins to form, which becomes increasingly more abundant with increasing amounts of H_3_PO_4_ and eventually becomes the major component with 10 equiv of H_3_PO_4_. As opposed to the H_3_PO_3_ titration, however, only the **Cu**_**27**_ **CO**_**3**_, **Cu**_**30**_ **CO**_**3**_ and **Cu**_**31**_ **CO**_**3**_ species disappear completely and only at higher equiv of H_3_PO_4_, whereas **Cu**_**29**_ **CO**_**3**_ is still present at 10 equiv of H_3_PO_4_. Another major difference compared to H_3_PO_3_ is that no other **Cu**_***n***_ **HPO**_**4**_ species form at any point during the titration, and all nanojars decompose at 15 equiv of H_3_PO_4_. Similar results were obtained using (Bu_4_N)­H_2_PO_4_ instead of H_3_PO_4_. Although the H_2_PO_4_^–^ ion (p*K*_a_ = 7.2) is a much weaker acid than H_3_PO_4_, it is still approximately 3 orders of magnitude stronger than HCO_3_^–^ (p*K*_a_ = 10.3) and therefore it easily protonates the CO_3_^2–^ ion.

### Etching of the Cu_*n*_XPO_3_ (X = OH, H or F) Nanojar Mixtures with NH_3_

To test the relative stability of nanojars of different sizes with a given anion, solutions of nanojars were saturated with NH_3_(g) and analyzed using ESI-MS. THF was used as solvent, in which both nanojars and NH_3_ are highly soluble. Treatment of a **Cu**_***n***_ **XPO**_**3**_ (X = H, F; *n* = 27–31) nanojar mixture with an excess of gaseous ammonia in THF solution reveals that only **Cu**_**31**_ **XPO**_**3**_ survives the effect of the strongly coordinating NH_3_, which breaks up the smaller (*n* = 27–30) nanojars and cleanly converts them into the most stable nanojar with the XPO_3_^2–^ ion in the presence of NH_3_ (Figures S8 and S9). In the case of **Cu**_***n***_ **HPO**_**4**_ (*n* = 27, 29, 31), a small amount of **Cu**_**29**_ **HPO**_**4**_ also remains along with **Cu**_**31**_ **HPO**_**4**_ (Figure S10). By contrast, all **Cu**_***n***_ **CO**_**3**_ (*n* = 27, 29–31) species convert into **Cu**_**27**_ **CO**_**3**_ upon treatment with NH_3_ (Figure S10), whereas all **Cu**_***n***_ **EO**_**4**_ (E = Mo, W; *n* = 28–33) species convert into **Cu**_**32**_ **EO**_**4**_, the most stable nanojars with the smaller CO_3_^2–^ and larger MoO_4_^2–^ and WO_4_^2–^ ions, respectively.^65,66^ The behavior of **Cu**_***n***_ **XPO**_**3**_ is similar to that of nanojars with entrapped SO_4_^2–^ anions, **Cu**_***n***_ **SO**_**4**_, which also favor the **Cu**_**31**_ nanojar.^65^ With tetrahedral anions of intermediate size (CrO_4_^2–^, SeO_4_^2–^), both **Cu**_**31**_ and **Cu**_**32**_ species survive the NH_3_ treatment.^66,67^

### Structural Analysis by X-ray Crystallography

To study the supramolecular binding parameters between the different phosphorus anion guests and nanojar hosts, the structures of several such complexes were examined using single-crystal X-ray diffraction. Two crystallographically independent **Cu**_**7+13+9**_ **HPO**_**4**_ units, related by a pseudoinversion center, are found within the asymmetric unit of the triclinic (*P*1̅) crystal lattice of (Bu_4_N)_2_[HPO_4_⊂{*cis*-Cu^II^(μ-OH)­(μ-pz)}_7+13+9_] (**1**, from toluene/*n*-heptane) (Figures 3 and S16, Table S1). Only small structural differences are observed between the two pseudoenantiomeric units, which are almost superimposable when one of the units is inverted (Figure S25). Notably, no significant disorder is observed for either the pyrazolate or the entrapped anion, which is rather rare for nanojar structures. In contrast, (Bu_4_N)_2_[HPO_4_⊂{*cis*-Cu^II^(μ-OH)­(μ-pz)}_8+13+8_] (**2**, from chlorobenzene/*n*-heptane) crystallizes in a higher, monoclinic (*C*2/*c*) lattice, in which the nanojar moiety is located on a 2-fold rotation axis running through the center of the nanojar and the asymmetric unit contains one Cu_8_ ring and half of the Cu_13_ ring (Figures 3 and S17, Table S1). Also, a partially occupied H_2_O molecule (with occupancy 0.25) is incorporated between one of the Cu_8_ rings and an adjacent Bu_4_N^+^ counterion, with closest O···O distances between H_2_O and nanojar OH groups (for H-bonds) of 2.974(11) and 3.248(12) Å (Figure S26). Different types of disorder are observed in **2**: symmetry-related disorder (around the *C*_2_ axis) which affects the central anion, part of the Cu_13_ ring and the H_2_O molecule, and nonsymmetry related disorder associated with a different part of the Cu_13_ ring and an OH group of the Cu_8_ rings (Figure S27). Structural parameters, including Cu–O and Cu–N bond lengths, average *trans* and *cis* N–Cu–O bond angles, average Cu···Cu distances within Cu_*x*_ rings, average inter-ring Cu···O distances less than the sum of the van der Waals radii of Cu and O (2.92 Å), H-bonding parameters between Cu_*x*_ rings and the entrapped anion as well as between Cu_*x*_ rings, average dihedral, twist and fold angles between pyrazolate moieties and adjacent Cu–O–Cu units (as defined earlier)^68^ as well as between adjacent pyrazolate moieties (as defined earlier),^53^ average copper coordination geometry indexes, and average deviations of Cu atoms in different Cu_*x*_ rings from the Cu_*x*_ mean-planes are summarized in Tables S4–S34. Hydrogen bonding patterns around the entrapped HPO_4_^2–^ ions and packing diagrams for **1** and **2** are shown in Figures 4 and S28, respectively. In both **1** and **2**, the protonation site of HPO_4_^2–^ is confirmed by one significantly longer P–O bond (1.610(3)/1.607(3) and 1.611(5) Å, respectively) compared to the other three (1.503(3)–1.519(3)/1.505(3)–1.518(3) and 1.455(4)–1.546(5) Å). Indeed, the average P–O distance for protonated oxygen atoms of noncovalently bonded HPO_4_^2–^ ions in the Cambridge Structural Database (CSD) is 1.59(1) Å, whereas the corresponding value for nonprotonated oxygen atoms is 1.52(1) Å.^69^ This protonated OH group protrudes through the Cu_9_ ring in **1** and through the Cu_8_ ring in **2**, and forms an H bond with an OH group of those rings at donor–acceptor O···O distances of 2.820(4)/2.803(4) and 2.823(6) Å, respectively.

**Figure 3:**
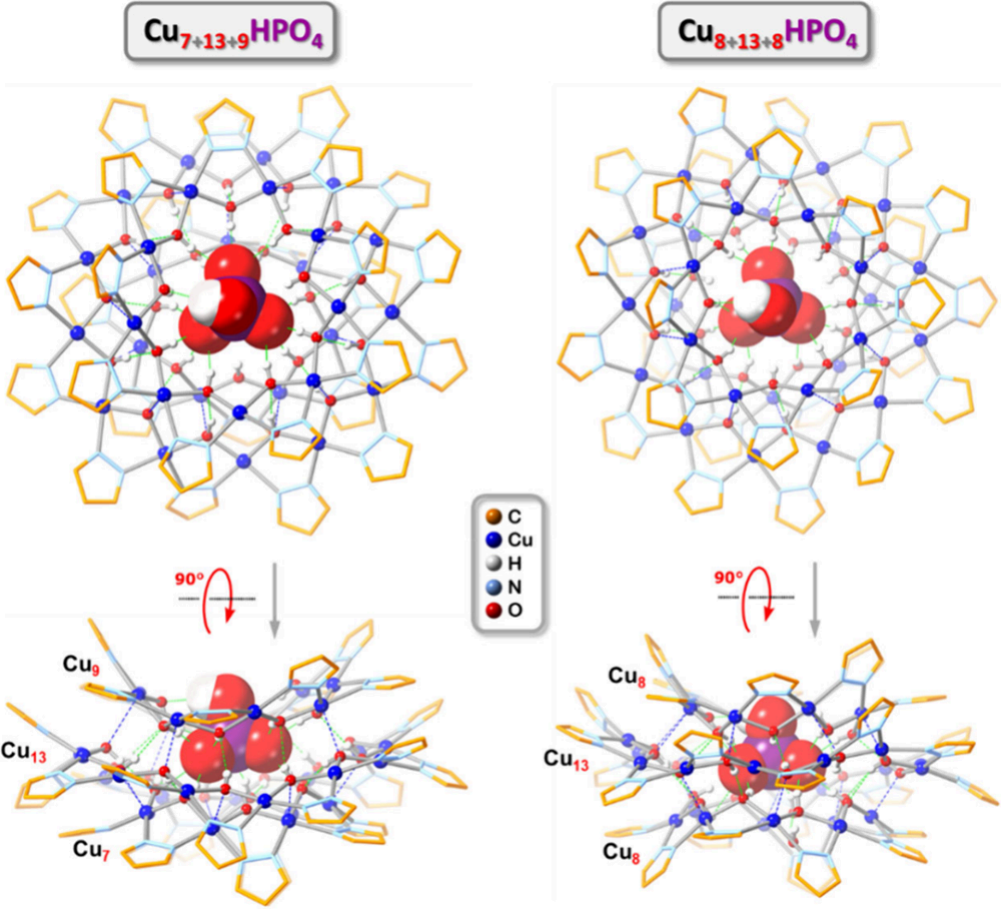
Ball-and-stick representation of the crystal structures of **Cu**_**7+13+9**_ **HPO**_**4**_ (**1**; unit 1 shown) and **Cu**_**8+13+8**_ **HPO**_**4**_ (**2**) (top- and side-views). Green and blue dotted lines indicate hydrogen bonds and axial Cu···O interactions, respectively. Counterions, lattice solvent molecules and C–H bond H atoms are omitted for clarity, and only the major component is shown for disordered moieties.

**Figure 4:**
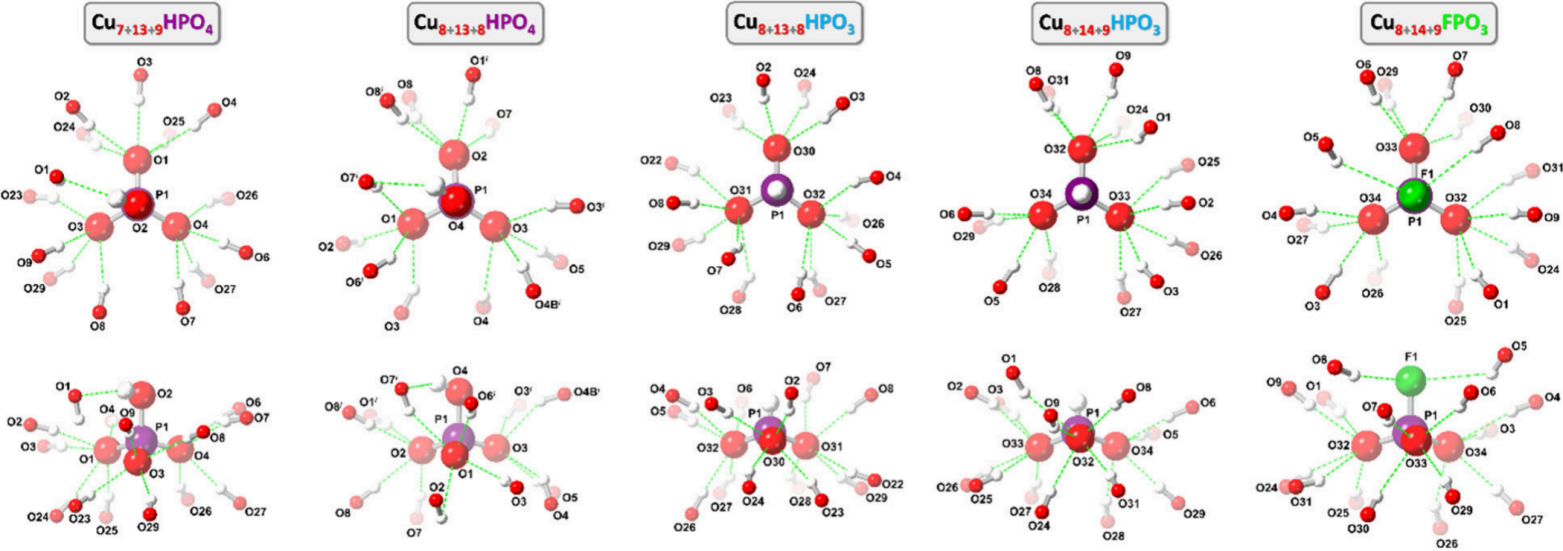
Comparison of the H-bonding patterns (top- and side-views) in **Cu**_**29**_ **HPO**_**4**_ (**1**, unit 1), **Cu**_**29**_ **HPO**_**4**_ (**2**), **Cu**_**29**_ **HPO**_**3**_ (**3**), **Cu**_**31**_ **HPO**_**3**_ (**4a**) and **Cu**_**31**_ **FPO**_**3**_ (**5a**). Only the major component is shown for disordered anions in **2**, **3** and **4a**.

The supramolecular binding site of HPO_4_^2–^ in nanojars is similar to the one in phosphate binding proteins, where the HPO_4_^2–^ ion is surrounded by 11–12 O–H/N–H hydrogen bond donors from the protein backbone. The major difference in proteins is that the H bond acceptor for the protonated phosphate O atom is an anionic aspartate group with a much shorter donor–acceptor O···O distance of 2.43–2.52 Å. This very short, charge-assisted H bond is responsible for the specific binding of the monoprotonated phosphate dianion.^70–72^

The monoclinic (*P*2_1_/*c*) crystal structure of (Bu_4_N)_2_[HPO_3_^2–^⊂{*cis*-Cu^II^(μ-OH)­(μ-pz)}_8+13+8_] (**3**, from 1,2-dichlorobenzene/*n*-pentane) is of lower symmetry than the one of its analog (monoclinic, *C*2/*c*) obtained earlier with a different counterion (Bn_3_MeN^+^) and from a different solvent system (nitrobenzene/*n*-pentane).^54^ As opposed to the earlier structure, in which the nanojar moiety is located on a 2-fold rotation axis running through the center of the nanojar (as in **2**), the whole nanojar is within the asymmetric unit of **3** (Figure 5, Table S1). Similarly to the higher symmetry structure, in which the HPO_3_^2–^ anion is disordered over two positions due to the additional symmetry, in **3** the HPO_3_^2–^ anion is also disordered over two positions (0.63/0.37) by a pseudo-*C*_2_ operation. In **3**, however, an additional, partially occupied H_2_O molecule site (with occupancy 0.31) is found between one of the Cu_8_ rings and an adjacent Bu_4_N^+^ counterion (as in **2**), with closest O···O distances between H_2_O and nanojar OH groups (for H-bonds) of 2.924(14) and 3.323(15) Å.

**Figure 5:**
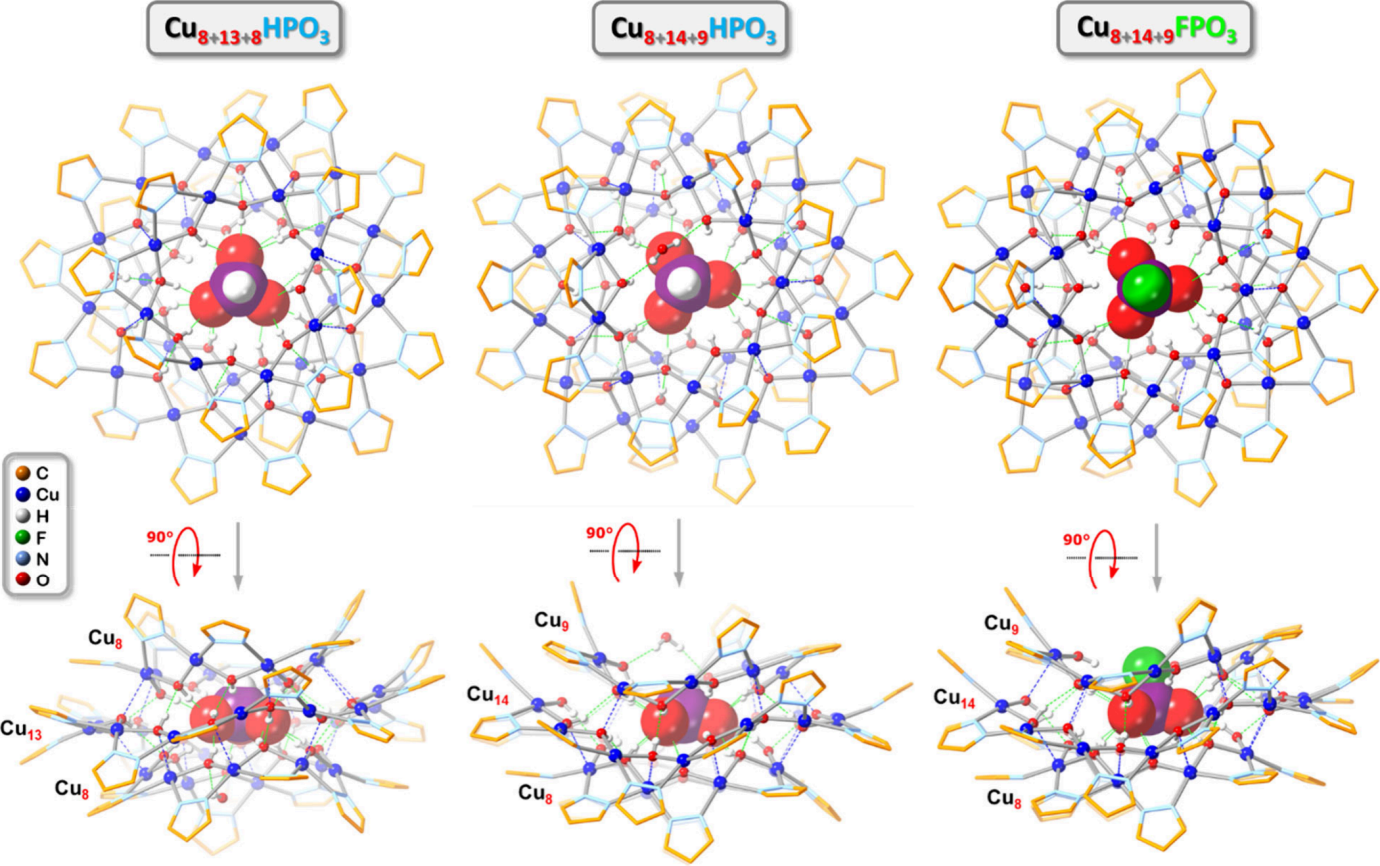
Ball-and-stick representation of the crystal structures of **Cu**_**29**_ **HPO**_**3**_ (**3**), **Cu**_**31**_ **HPO**_**3**_ (**4a**) and **Cu**_**31**_ **FPO**_**3**_ (**5a**) (top- and side-views). Green and blue dotted lines indicate hydrogen bonds and axial Cu···O interactions, respectively. Counterions, lattice solvent molecules and C–H bond H atoms are omitted for clarity, and only the major component is shown for disordered moieties.

While the nanojar bonding parameters in **3** are virtually identical to those of the previously studied analog (avg. Cu–O and Cu–N bond lengths: 1.922(3) vs 1.920(4) and 1.979(4) vs 1.978(5) Å; avg. *trans* and *cis* N–Cu–O bond angles: 170.4(2) vs 170.5(2) and 85.6(2) vs 85.5(2)°; avg. Cu···Cu distance within Cu_*x*_ rings: 3.328(1) vs 3.329(2) Å),^54^ slightly longer average inter-ring H-bonded O···O distances less than 3.20 Å (2.826(5) vs 2.805(5) Å) and Cu···O distances less than the sum of the van der Waals radii of Cu and O of 2.92 Å (2.541(4) vs 2.490(5) Å) are observed in **3** (Tables S4–S34). Also, the number and the average of H-bonded O···O distances less than 3.20 Å between the nanojar and the entrapped HPO_3_^2–^ ion (major disordered position) is higher in **3** (14 vs 12 and 2.92(1) vs 2.80(2) Å) (Figure S29). In terms of deviation from planarity, the range of distances of individual Cu atoms from the average Cu_8_ and Cu_13_ mean-planes is considerably larger in **3** (0.026–0.296 and 0.037–0.317 vs 0.007–0.275 and 0.007–0.275 Å for the Cu_8_ rings, and 0.003–0.914 vs 0.000–0.647 Å for the Cu_13_ ring), despite the average of all deviations being comparable (0.238 vs 0.228). Lastly, the conformations of the Cu_8_ and Cu_13_ rings in **3** and the earlier analog are quite similar, so that the Cu_13_ and one of the two Cu_8_ rings are close to being superimposable, whereas two of the pyrazolate moieties of the other Cu_8_ ring are rather far from being superimposable, with centroid-centroid distances of 1.341 and 2.120 Å between the two structures (Figure 6). The different packing diagrams of **3** and the higher symmetry analog are illustrated in Figure S30. The structure of **3** (**Cu**_**8+13+8**_ **HPO**_**3**_) is also similar to the one of **2** (**Cu**_**8+13+8**_ **HPO**_**4**_), though they are not superimposable (Figure S31).

**Figure 6:**
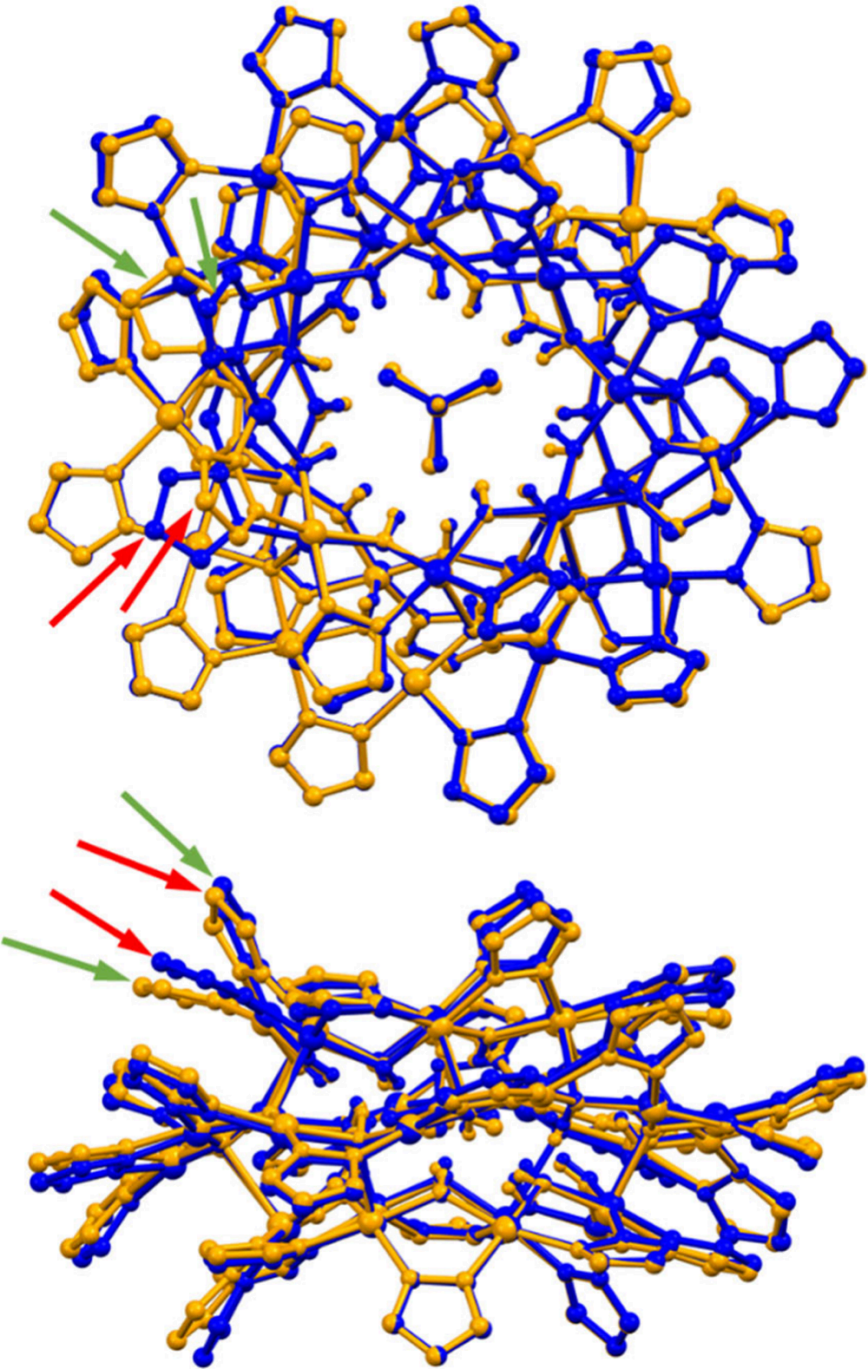
Overlay (top- and side-views) of the structure of **3** (*P*2_1_/*c*; blue) and the previously described analog (*C*2/*c*; orange).^54^ Red and green arrows identify the two pairs of pz^–^ moieties that are significantly deviated from superimposability. C–H bond hydrogen atoms, counterions and solvent molecules are omitted for clarity, and only the major component is shown for disordered moieties.

Three different pseudopolymorphs (all triclinic, *P*1̅) of the (Bu_4_N)_2_[HPO_3_^2–^⊂{*cis*-Cu^II^(μ-OH)­(μ-pz)}_8+14+9_] (**Cu**_**31**_ **HPO**_**3**_) nanojar were crystallized, from a 1,2-dichlorobenzene solution by vapor diffusion of *n*-pentane (**4a**), hexanes (**4b**), or *n*-heptane (**4c**) (Figures 5 and S32, Table S2). The structures of **4a**–**4c** are isomorphous with each other but not with the ones of **Cu**_**31**_ **EX**_**4**_ (X = O, E = HP, S, Se, Cr, Mo, W; X = F, E = Be).^66,73–75^ In all three structures, the HPO_3_^2–^ ion is disordered over two positions (0.85/0.15 in **4a**, 0.75/0.25 in **4b**, and 0.77/0.23 in **4c**) by a pseudo 2-fold rotation. As in **2** and **3**, the structures of **4a**–**4c** also include an H_2_O molecule, with three major differences: a) the H_2_O molecule has full occupancy; b) it is incorporated between the Cu_9_ ring (instead of Cu_8_ ring in **3**) and an adjacent Bu_4_N^+^ counterion; c) it is closer to the nanojar unit, with shorter O···O distances (2.867(13) and 2.977(10) Å in **4a**; 2.890(6) and 2.965(5) Å in **4b**; 2.883(11) and 2.980(9) Å in **4c**). Whereas the structures of **4a**–**4c** are almost completely superimposable (Figure S32), a slight difference is observed in the H-bonding network around the central HPO_3_^2–^ ion. Thus, the number of H-bonds shorter than 3.20 Å varies from 13 in **4a** to 14 in **4b** and 12 in **4c** (Figure S33, Table S5).

In the structure of **4b**, a partial substitution of the pyrazolate by acetate moieties is observed. Two such substitutions are present in both the Cu_8_ and Cu_9_ rings, with occupancies of 0.18 and 0.21 and 0.36 and 0.77, respectively (Figure S34). The source of the acetate ion has not been identified; nevertheless, occasionally acetate has been observed in other nanojar structures.^76,77^

The binding of phosphite by nanojars also resembles its binding by proteins, although with significant differences. In the anion binding pocket of periplasmic binding proteins, phosphite is found in its monoanionic, protonated form (HPO_3_H^–^), bound by both O–H (tyrosine, serine and threonine) and N–H hydrogen bond donors (histidine, serine and threonine), as well as a hydrogen bond acceptor (aspartate or tyrosine O atom).^78,79^ In the protein, the specificity for phosphite is conferred by the presence of a P–H···π interaction with an aromatic residue (tyrosine) in the anion-binding pocket.

With the FPO_3_^2–^ ion, three different pseudopolymorphs of (Bu_4_N)_2_[FPO_3_^2–^⊂{*cis*-Cu^II^(μ-OH)­(μ-pz)}_8+14+9_] (**Cu**_**31**_ **FPO**_**3**_) nanojars have been obtained (all triclinic, *P*1̅), of which **5a** (from 1,2-dichlorobenzene/*n*-pentane) and **5b** (from chlorobenzene/*n*-pentane) are isomorphous, whereas **5c** (from bromobenzene/nitrobenzene/hexanes) has a different unit cell (Figure 5, Table S3). **5a** and **5b** are also isomorphous with **Cu**_**31**_ **EX**_**4**_ (X = O, E = HP, S, Se, Cr, Mo, W; X = F, E = Be),^66,73–75^ but not with **Cu**_**31**_ **HPO**_**3**_ (**4a**–**4c**), although their structures are close to being superimposable (Figure S35). Unlike the **Cu**_**31**_ **HPO**_**3**_ analogs, the **Cu**_**31**_ **FPO**_**3**_ structures do not incorporate an H_2_O molecule, and the FPO_3_^2–^ ion is not disordered. In all three structures, the FPO_3_^2–^ ion is H-bonded to 13 OH groups through its O atoms (with average O···O distances of 2.953 (11), 2.949(4) and 2.946(7) Å) and to two additional OH groups through its F atom with average F···O distances of 3.247 (11), 3.234(5) and 3.169(7) Å in **5a**, **5b** and **5c**, respectively (Figure S36). Figure 4 provides a comparison of the H-bonding patterns in the different nanojars (**1**–**5**). While **5c** is not isomorphous with **5a** and **5b**, their structures are again close to being superimposable (Figure S37). The packing diagrams of **4a**–**4c**, however, are distinct from the ones of **5a**/**5b**, and more like the one of **5c** (Figure 7).

**Figure 7:**
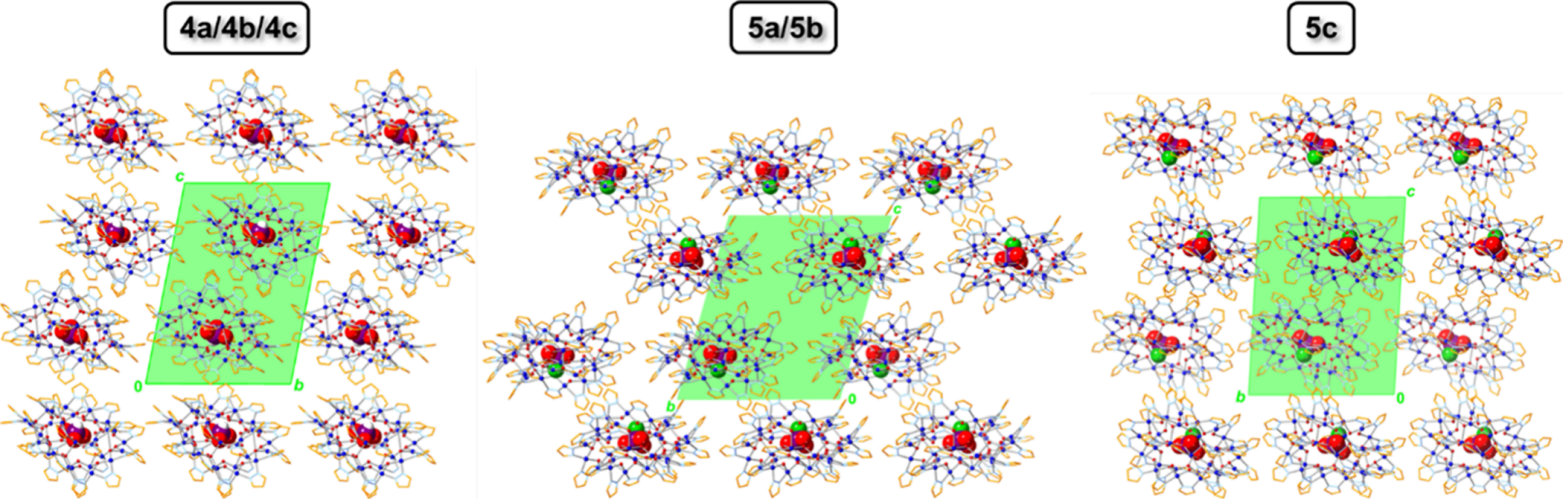
Comparison of the packing diagrams (along the *a* axis) of **4a**–**4c** and **5a**–**5c**. C–H and O–H bond H atoms, counterions and solvent molecules are omitted for clarity, and only the major component is shown for disordered moieties.

The binding of the FPO_3_^2–^ ion by **Cu**_**31**_ nanojars is practically identical to that of the previously studied hydrogen phosphate ion (HPO_4_^2–^).^73^ Indeed, the structure of **Cu**_**31**_ **FPO**_**3**_ is almost completely superimposable with the one of **Cu**_**31**_ **HPO**_**4**_ (Figure S38). Fluorophosphate has also been found to bind similarly to hydrogen phosphate in phosphate binding proteins, but not in an identical position.^60^ The observed difference might be attributable to the fact that whereas in **Cu**_**31**_ **HPO**_**4**_ the phosphate OH proton does not form a strong H-bond with the host (closest O···O distance: 3.20 Å), in the protein the phosphate proton forms a short H-bond with an aspartate residue in the binding pocket. This additional strong H-bond is responsible for the extreme selectivity of proteins for HPO_4_^2–^ vs SO_4_^2–^ and vice versa,^71,80,81^ and to some extent, even for the differentiation between HPO_4_^2–^ and the structurally very similar but highly toxic HAsO_4_^2–^ ion.^82^

### Variable-Temperature ^1^H NMR Spectroscopy

While mass spectrometry does provide the quickest characterization of nanojar mixtures in solution, it is unable to distinguish between nanojar isomers. Yet, previous X-ray crystallographic studies have clearly established the existence of two different isomers for the **Cu**_**29**_ (**Cu**_**7+13+9**_ and **Cu**_**8+13+8**_),^65^ **Cu**_**30**_ (**Cu**_**7+14+9**_ and **Cu**_**8+14+8**_)^83^ and **Cu**_**32**_ (**Cu**_**9+14+9**_ and **Cu**_**8+14+10**_) nanojars,^66^ whereas only one isomer has been observed for **Cu**_**27**_ (**Cu**_**6+12+9**_), **Cu**_**28**_ (**Cu**_**6+12+10**_) and **Cu**_**31**_ (**Cu**_**8+14+9**_). Therefore, NMR spectroscopy is a crucial technique for a thorough characterization of nanojar mixtures in solution, owing to the distinct NMR signatures of the different isomers of a given **Cu**_***n***_ nanojar. Although the presence of paramagnetic Cu^2+^ ions causes broadening and a strong downfield (for pz^–^ protons) or upfield (for HO^–^ protons) shift of the signals, the strong antiferromagnetic coupling between Cu^2+^ centers allows for the observation of fairly narrow peaks in most cases.^84^

In the **Cu**_***n***_ **XPO**_**3**_ (X = H or F) mixtures, three major components are indicated by ESI-MS: **Cu**_**27**_ **XPO**_**3**_, **Cu**_**29**_ **XPO**_**3**_ and **Cu**_**31**_ **XPO**_**3**_ (Figure 1). Of these, two isomers are expected for **Cu**_**29**_ **XPO**_**3**_: **Cu**_**7+13+9**_ **XPO**_**3**_ and **Cu**_**8+13+8**_ **XPO**_**3**_. Only the latter could be identified and characterized by X-ray crystallography (with the HPO_3_^2–^ ion), as described above. Nevertheless, ^1^H NMR reveals the presence of the other isomer, **Cu**_**7+13+9**_ **XPO**_**3**_ as well in solution (Figures 8 and S39). In fact, a much larger amount of this isomer is observed compared to the symmetrical one, identifying it as the kinetically favored isomer during nanojar synthesis by self-assembly. As discussed below, however, heating leads to its decomposition and transformation to **Cu**_**31**_ **XPO**_**3**_, making **Cu**_**8+13+8**_ **XPO**_**3**_ the thermodynamically more stable **Cu**_**29**_ nanojar isomer.

**Figure 8:**
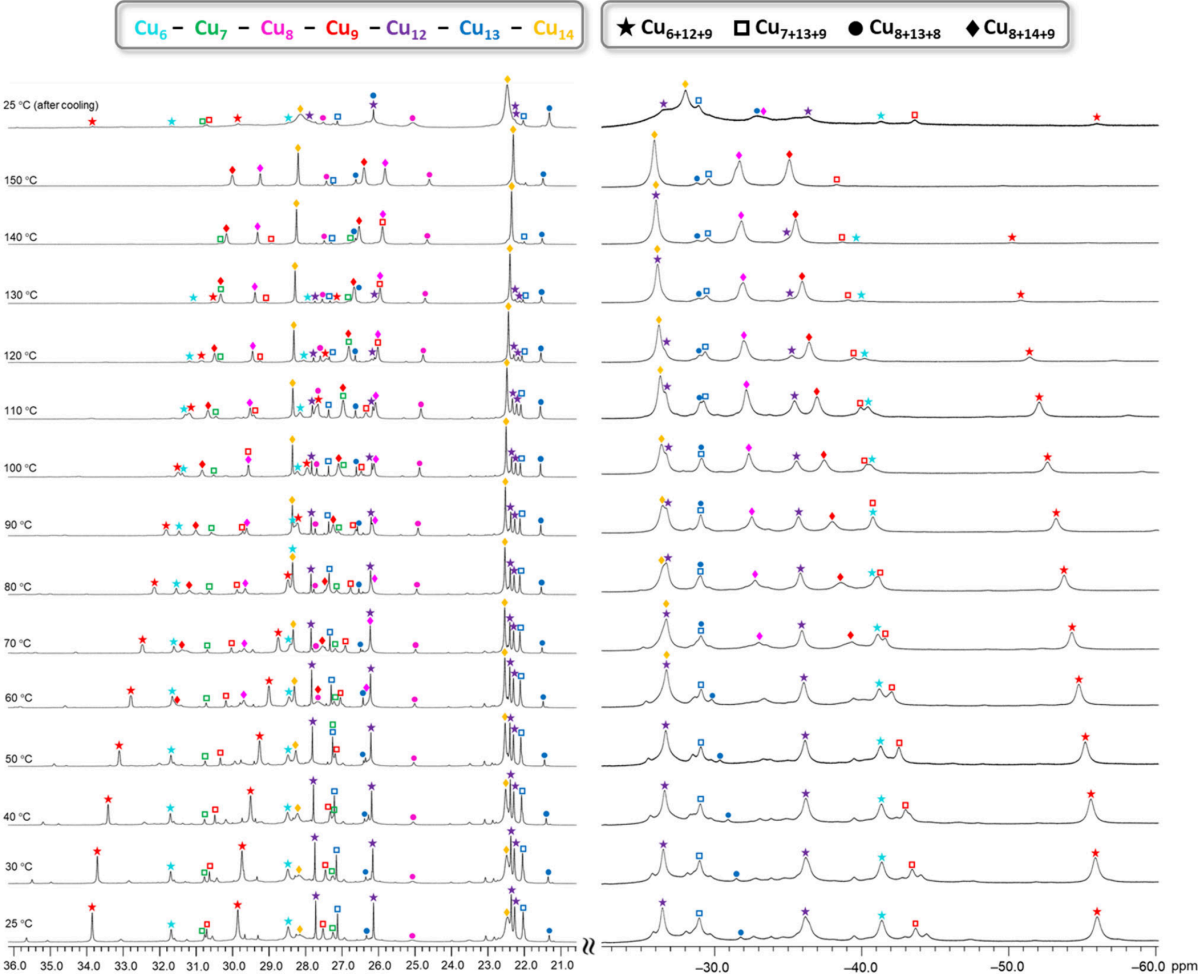
Variable-temperature ^1^H NMR spectra of the Cu_*n*_HPO_3_ (*n* = 27–31) nanojar mixture in DMSO-*d*_6_, showing pyrazolate and OH proton signals in the 21 to 36 and −25 to −58 ppm windows, respectively. Assignments were made based on correlations with ESI-MS spectra and previous results with different anions. The temperatures shown are the target temperatures of the probe.

The chemical shifts (δ) of ^1^H NMR signals for the pz^–^ and OH^–^ groups of **Cu**_***n***_ **HPO**_**3**_ and **Cu**_***n***_ **FPO**_**3**_ are similar (with differences ranging from 0.01 to 0.59 ppm), except for the OH proton signals of the Cu_9_ rings in **Cu**_**6+12+9**_ **XPO**_**3**_, **Cu**_**7+13+9**_ **XPO**_**3**_ and **Cu**_**8+14+9**_ **XPO**_**3**_ (Table S35). At 25 °C, the difference between the chemical shifts for those signals is 4.67 (3.45) and 3.87 (3.17) ppm for **Cu**_**6+12+9**_ **XPO**_**3**_ and **Cu**_**7+13+9**_ **XPO**_**3**_, respectively. The corresponding peaks of **Cu**_**31**_ **XPO**_**3**_ are too broad at 25 °C to be unambiguously assigned, but they become increasingly sharper at higher temperatures. Thus, at 80 °C the corresponding difference for **Cu**_**31**_ **XPO**_**3**_ is 3.65 ppm. This marked difference in the case of the Cu_9_ ring is due to the fact that the H or F atom of the XPO_3_^2–^ ion points toward the Cu_9_ ring in nanojars, thus affecting the corresponding H-bonded H atoms the most. The Cu_12_–Cu_14_ rings do not form H-bonds with the XPO_3_^2–^ ion, and therefore they are affected the least.

The VT ^1^H NMR experiments reveal a different temperature dependence of the chemical shifts of the various Cu_*x*_ rings in **Cu**_***n***_ **XPO**_**3**_ (X = H or F) (Figures 9 and S42, Table S35). Most peaks become less paramagnetically shifted at higher temperatures indicating a Curie behavior (δ ∝ 1/*T*),^84^ except the Cu_12_–Cu_14_ ring pyrazolate signals which are rather insensitive to temperature changes.

**Figure 9:**
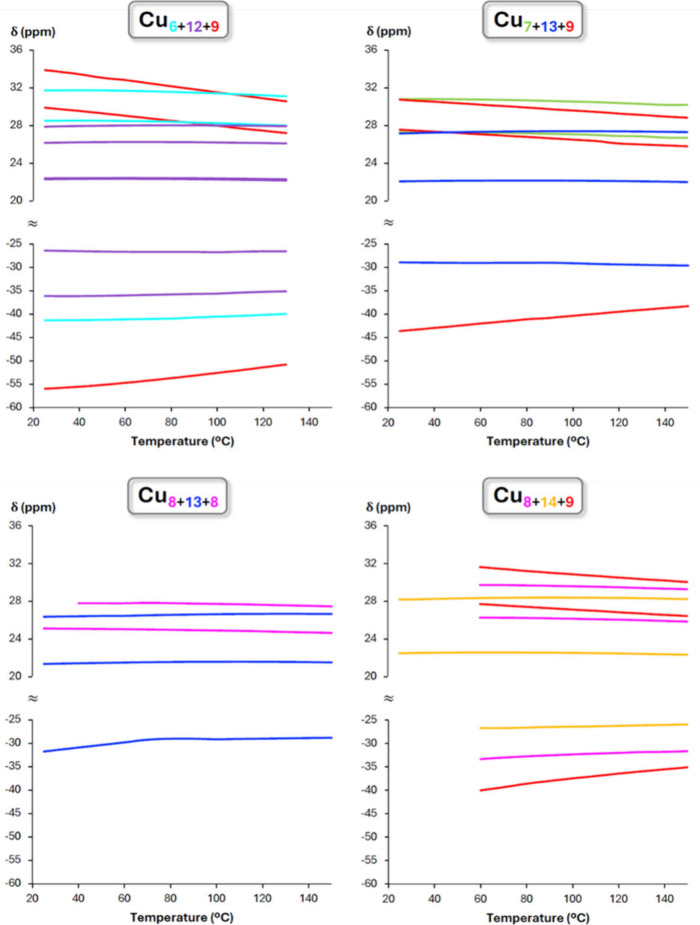
Temperature-dependent variation of the ^1^H NMR chemical shifts (δ) in DMSO-*d*_6_ of the different Cu_*x*_ ring protons in **Cu**_***n***_ **HPO**_**3**_.

The differently sized **Cu**_***n***_ **XPO**_**3**_ (X = H or F) nanojars have different thermal stabilities in DMSO-*d*_6_ solution. As shown in Figures 8 and S39, the **Cu**_**27**_ **XPO**_**3**_ and **Cu**_**7+13+9**_ **XPO**_**3**_ nanojars gradually disappear on heating from ambient temperature to 150 °C, as they transform into the most stable nanojar at that temperature, **Cu**_**31**_ **XPO**_**3**_. The only other nanojar stable at 150 °C is **Cu**_**8+13+8**_ **XPO**_**3**_, which does not seem to be affected by heating. The behavior of the HPO_3_^2–^ and FPO_3_^2–^ entrapping nanojars is similar and resembles the one of the SO_4_^2–^ analog,^85^ but different from the one of the CO_3_^2–^ analog. In the case of **Cu**_***n***_ **CO**_**3**_ (*n* = 27, 29, 31), heating induces decomposition of **Cu**_**31**_ **CO**_**3**_ and transformation into **Cu**_**8+13+8**_ **CO**_**3**_, whereas **Cu**_**27**_ **CO**_**3**_ and both isomers of **Cu**_**29**_ **CO**_**3**_ are present up to 150 °C.^65,84^ Upon cooling the **Cu**_***n***_ **XPO**_**3**_ (X = H or F) solution from 150 °C back to ambient temperature, re-equilibration is observed affording small amounts of **Cu**_**27**_ **XPO**_**3**_ and **Cu**_**7+13+9**_ **XPO**_**3**_, and an additional amount of **Cu**_**8+13+8**_ **XPO**_**3**_.

In the case of **Cu**_***n***_ **HPO**_**4**_, only the **Cu**_**29**_ and **Cu**_**31**_ species form in larger amounts, whereas **Cu**_**27**_ and the other sizes are much less abundant (Figures 1 and S6). ^1^H NMR confirms that as in the case of the **Cu**_***n***_ **XPO**_**3**_ (X = H, F) analogues, the **Cu**_**7+13+9**_ **HPO**_**4**_ isomer is much more abundant in the as-synthesized **Cu**_***n***_ **HPO**_**4**_ mixture than the other **Cu**_**29**_ isomer, **Cu**_**8+13+8**_ **HPO**_**4**_ (Figure S40). Likewise, on heating **Cu**_**7+13+9**_ **HPO**_**4**_ decomposes whereas small amounts of **Cu**_**8+13+8**_ **HPO**_**4**_ are still present at 150 °C, along with **Cu**_**8+14+9**_ **HPO**_**4**_ as the major component. The corresponding Curie plots are shown in Figure S43. A VT ^1^H NMR experiment was also performed on the almost pure **Cu**_**31**_ **HPO**_**4**_ sample obtained by extraction of phosphate from water, which shows chemical shift values practically identical to the ones of **Cu**_**31**_ **HPO**_**4**_ in the **Cu**_***n***_ **HPO**_**4**_ mixture (Figure S41, Table S35).

### ^19^F and ^31^P NMR Spectroscopy

Whereas the structure of the nanojar host can be studied in solution by ^1^H NMR, the entrapped anion guest can be probed using ^19^F and/or ^31^P NMR spectroscopy. The **Cu**_***n***_ **FPO**_**3**_ nanojars contain only 0.4% F and 0.6% P by mass; nevertheless, the 100% natural abundance of the ^19^F (sensitivity relative to ^1^H: 0.83) and ^31^P (sensitivity relative to ^1^H: 0.00663) nuclei allow for the detection of the FPO_3_^2–^ ions trapped inside different nanojars. The ^19^F NMR signal of the free FPO_3_^2–^ anion in (Bu_4_N)_2_FPO_3_ is found at −3.83 ppm at 25 °C in DMSO-*d*_6_ (referenced to C_6_H_5_CF_3_ as internal standard). Upon binding inside nanojars, the ^19^F chemical shift of the FPO_3_^2–^ anion moves either upfield to −8.54 ppm in **Cu**_**8+13+8**_ **FPO**_**3**_ and to −14.28 ppm in **Cu**_**6+12+9**_ **FPO**_**3**_, or downfield to 4.27 ppm in **Cu**_**6+12+10**_ **FPO**_**3**_, 7.93 ppm in **Cu**_**7+13+9**_ **FPO**_**3**_ and to 20.31 ppm in **Cu**_**8+14+9**_ **FPO**_**3**_ (Figure 10). These ^19^F NMR results corroborate the ^1^H NMR observation that the different **Cu**_***n***_ **FPO**_**3**_ nanojars have different thermal stabilities in DMSO-*d*_6_ solution. Indeed, heating the **Cu**_***n***_ **FPO**_**3**_ mixture to 150 °C followed by cooling to 25 °C leads to the almost complete disappearance of the ^19^F NMR peaks of **Cu**_**6+12+9**_ **FPO**_**3**_ (initially the most abundant species), **Cu**_**7+13+9**_ **FPO**_**3**_ and **Cu**_**8+13+8**_ **FPO**_**3**_, confirming **Cu**_**8+14+9**_ **FPO**_**3**_ to be the thermally most stable **Cu**_***n***_ **FPO**_**3**_ species. The large ^1^ *J*_F–P_ coupling values of 866–892 Hz in the **Cu**_***n***_ **FPO**_**3**_ nanojars (comparable to 837 Hz for the free FPO_3_^2–^ ion) along with the absence of other peaks confirm the identity of the FPO_3_^2–^ ion in nanojars and documents the lack of its hydrolysis. For Na_2_FPO_3_ in D_2_O, a ^1^ *J*_F–P_ value of 862 Hz (Figure S3) practically identical to the one reported (863 Hz) was obtained.^86^

**Figure 10:**
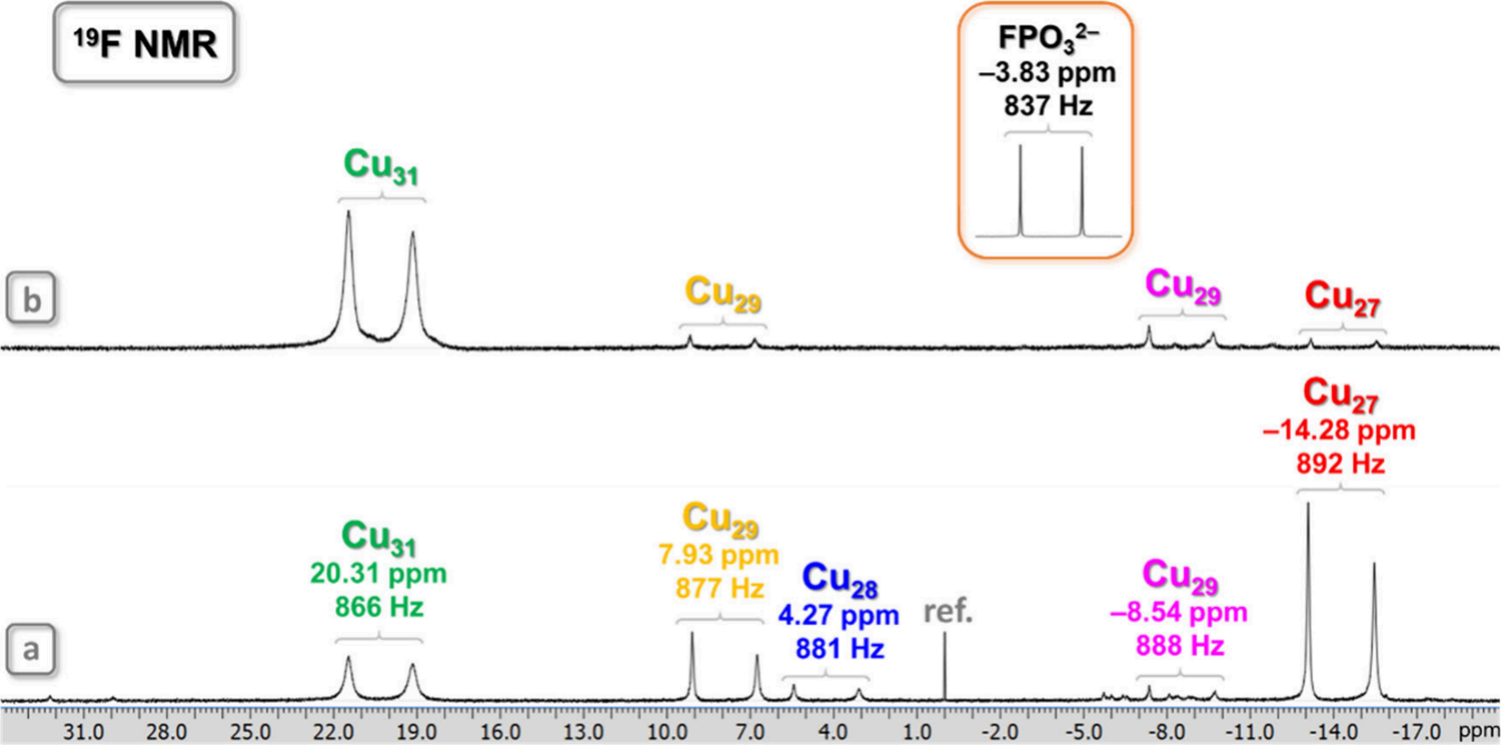
^19^F NMR spectra of the **Cu**_***n***_ **FPO**_**3**_ (Cu_*n*_; *n* = 27–29, 31) nanojar mixture in DMSO-*d*_6_ at ambient temperature, referenced to C_6_H_5_CF_3_ as internal standard, (a) before heating and (b) after heating to 150 °C. Chemical shift and ^1^ *J*_F–P_ coupling values are shown under the nanojar symbols. Color code for the Cu_29_ nanojars: yellow, Cu_7+13+9_; magenta, Cu_8+13+8_. The inset shows the signal of the free FPO_3_^2–^ ion as (Bu_4_N)_2_FPO_3_ in DMSO-*d*_6_ at 25 °C. Assignments were made based on correlations with ESI-MS and ^1^H NMR spectra.

The ^31^P NMR signals of the **Cu**_***n***_ **FPO**_**3**_ (*n* = 27–29, 31) nanojar mixture in DMSO-*d*_6_ are all shifted downfield compared to the free FPO_3_^2–^ ion (Figure 11). Thus, signals are observed at 9.70 ppm for **Cu**_**7+13+9**_ **FPO**_**3**_, 12.63 ppm for **Cu**_**8+14+9**_ **FPO**_**3**_, 13.35 ppm for **Cu**_**8+13+8**_ **FPO**_**3**_, 13.87 ppm for **Cu**_**6+12+9**_ **FPO**_**3**_ and 15.46 ppm for **Cu**_**6+12+10**_ **FPO**_**3**_, whereas the signal of the free FPO_3_^2–^ ion appears at 3.45 ppm (referenced to 85% H_3_PO_4_ in H_2_O as external standard in a coaxial NMR tube). As in the case of the ^19^F NMR signals, large ^1^ *J*_F–P_ coupling values of 868–893 Hz are observed for the ^31^P NMR signals of **Cu**_***n***_ **FPO**_**3**_, and heating confirms the superior thermal stability of **Cu**_**31**_ **FPO**_**3**_ compared to smaller nanojars. The high electronegativity of the F atom in FPO_3_^2–^ causes the window of ^31^P NMR signals to shift and shrink from 37.7–62.7 ppm in the case of phosphonate nanojars (**Cu**_***n***_ **RPO**_**3**_; R = alkyl, benzyl, phenyl)^53^ to 9.7–15.5 ppm for **Cu**_***n***_ **FPO**_**3**_.

**Figure 11:**
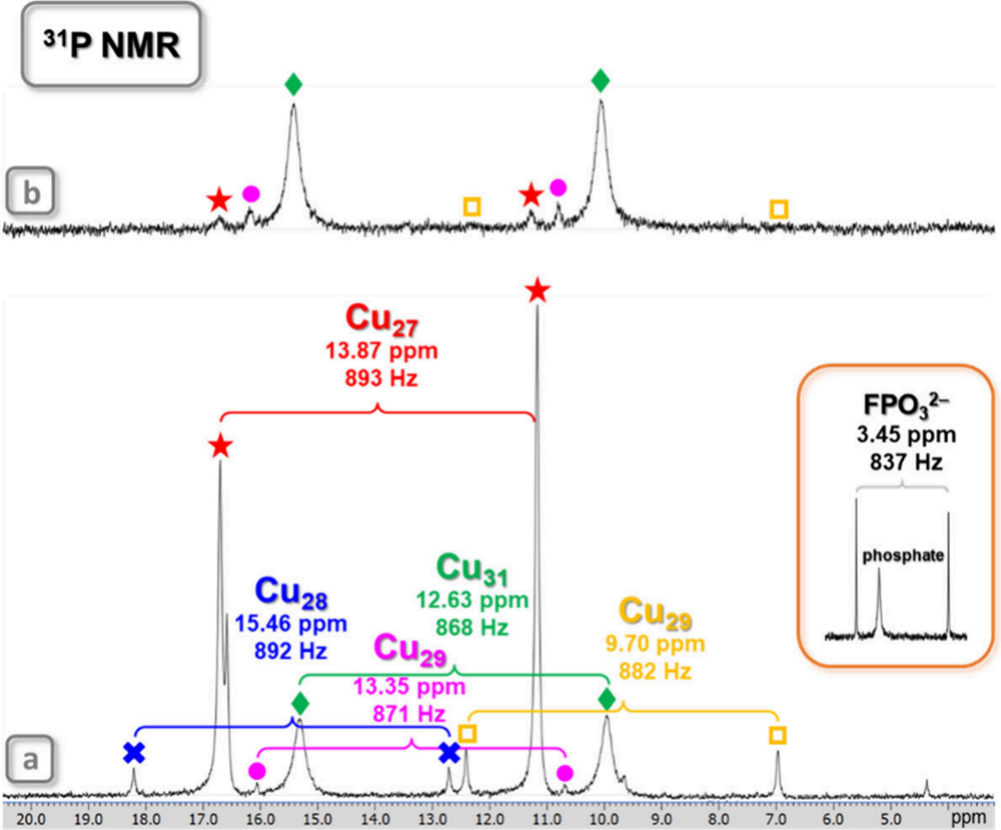
^31^P NMR spectra of the **Cu**_***n***_ **FPO**_**3**_ (Cu_*n*_; *n* = 27–29, 31) nanojar mixture in DMSO-*d*_6_ at ambient temperature referenced to 85% H_3_PO_4_ in H_2_O as external standard in a coaxial NMR tube, (a) before heating and (b) after heating to 150 °C. Chemical shift and ^1^ *J*_F–P_ coupling values are shown under the nanojar symbols. Color code for the Cu_29_ nanojars: yellow, Cu_7+13+9_; magenta, Cu_8+13+8_. The inset shows the signal of the free FPO_3_^2–^ ion as (Bu_4_N)_2_FPO_3_ in DMSO-*d*_6_ at 25 °C. Assignments were made based on correlations with ESI-MS and ^1^H/^19^F NMR spectra.

For **Cu**_***n***_ **HPO**_**3**_ (*n* = 27–29, 31), the ^31^P NMR signals are also shifted downfield compared to the free HPO_3_^2–^ ion (1.32 ppm), although to a much larger extent than in the case of **Cu**_***n***_ **FPO**_**3**_ (Figure 12). The corresponding signals are observed at 42.34 ppm for **Cu**_**8+13+8**_ **HPO**_**3**_, 58.46 ppm for **Cu**_**6+12+9**_ **HPO**_**3**_, 81.25 ppm for **Cu**_**6+12+10**_ **HPO**_**3**_, 95.11 ppm for **Cu**_**7+13+9**_ **HPO**_**3**_ and 100.00 ppm for **Cu**_**8+14+9**_ **HPO**_**3**_. The larger deshielding in **Cu**_***n***_ **HPO**_**3**_ compared to **Cu**_***n***_ **FPO**_**3**_ (despite the strong electron-withdrawing effect of F) points to a different magnetic environment within the same Cu_*n*_ nanojar with HPO_3_^2–^ vs FPO_3_^2–^ ions. This difference is further corroborated by the different magnitudes of shift for a given nanojar, with **Cu**_**7+13+9**_ and **Cu**_**6+12+10**_ having the least and the most shifted signals with FPO_3_^2–^, but **Cu**_**8+13+8**_ and **Cu**_**8+14+9**_ having the least and the most shifted signals with HPO_3_^2–^. The window of ^31^P NMR signals (42.3–100.0 ppm) for **Cu**_***n***_ **HPO**_**3**_ is larger than for phosphonate nanojars (**Cu**_**31**_ **RPO**_**3**_; R = alkyl, benzyl, phenyl; δ 37.7–62.7 ppm).^53^ The ^1^ *J*_H–P_ coupling constants are smaller compared to the ones of ^1^ *J*_F–P_, with rather consistent values of 555–559 Hz within the different **Cu**_***n***_ nanojars. As in the case of **Cu**_***n***_ **FPO**_**3**_, heating indicates that **Cu**_**8+14+9**_ **HPO**_**3**_ and **Cu**_**8+13+8**_ **HPO**_**3**_ are the most robust nanojars that survive heating to 150 °C in DMSO-*d*_6_.

**Figure 12:**
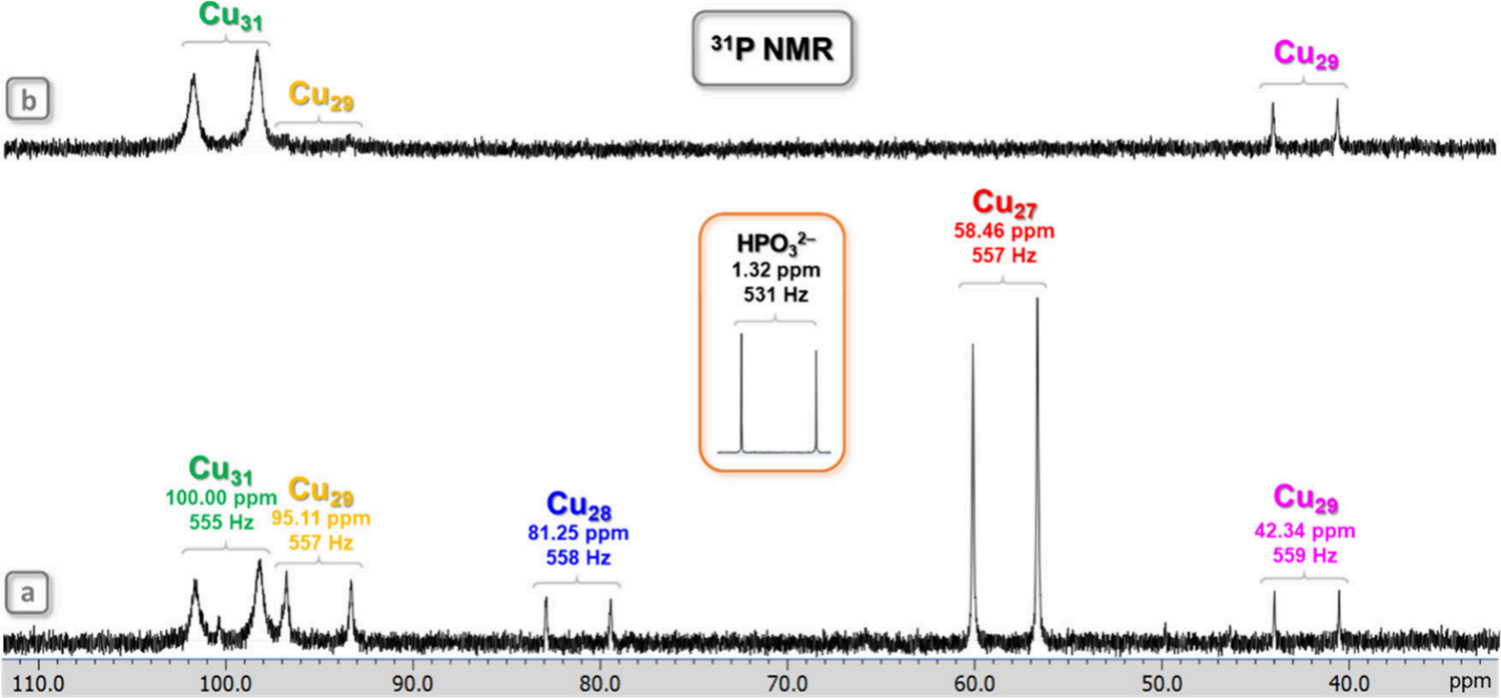
^31^P NMR spectra of the **Cu**_***n***_ **HPO**_**3**_ (Cu_*n*_; *n* = 27–31) nanojar mixture in DMSO-*d*_6_ at ambient temperature referenced to 85% H_3_PO_4_ in H_2_O as external standard in a coaxial NMR tube, (a) before heating and (b) after heating to 150 °C. Chemical shift and ^1^ *J*_H–P_ coupling values are shown under the nanojar symbols. Color code for the Cu_29_ nanojars: yellow, Cu_7+13+9_; magenta, Cu_8+13+8_. The inset shows the signal of the free HPO_3_^2–^ ion as (Bu_4_N)_2_HPO_3_ in DMSO-*d*_6_ at 25 °C. Assignments were made based on correlations with ESI-MS and ^1^H NMR spectra.

For **Cu**_***n***_ **HPO**_**4**_ (*n* = 29 and 31), ^31^P NMR spectra were recorded at three different temperatures (20, 50, and 80 °C), documenting a temperature-dependence similar to the one observed in the case of ^1^H NMR signals. Thus, a massive shift of ∼8 ppm units is observed for the **Cu**_**7+13+9**_ **HPO**_**4**_ signal upon heating from 20 and 80 °C, whereas the corresponding shifts for the **Cu**_**8+14+9**_ **HPO**_**4**_ and **Cu**_**8+13+8**_ **HPO**_**4**_ signals are only 0.5 and 0.25 ppm, respectively. It is also notable that only the signals of **Cu**_**7+13+9**_ **HPO**_**4**_ and **Cu**_**8+14+9**_ **HPO**_**4**_ display a Curie behavior (δ ∝ 1/*T*), whereas in the case of **Cu**_**8+13+8**_ **HPO**_**4**_ a slight anti-Curie behavior (δ ∝ *T*) is observed (Figure 13).

**Figure 13:**
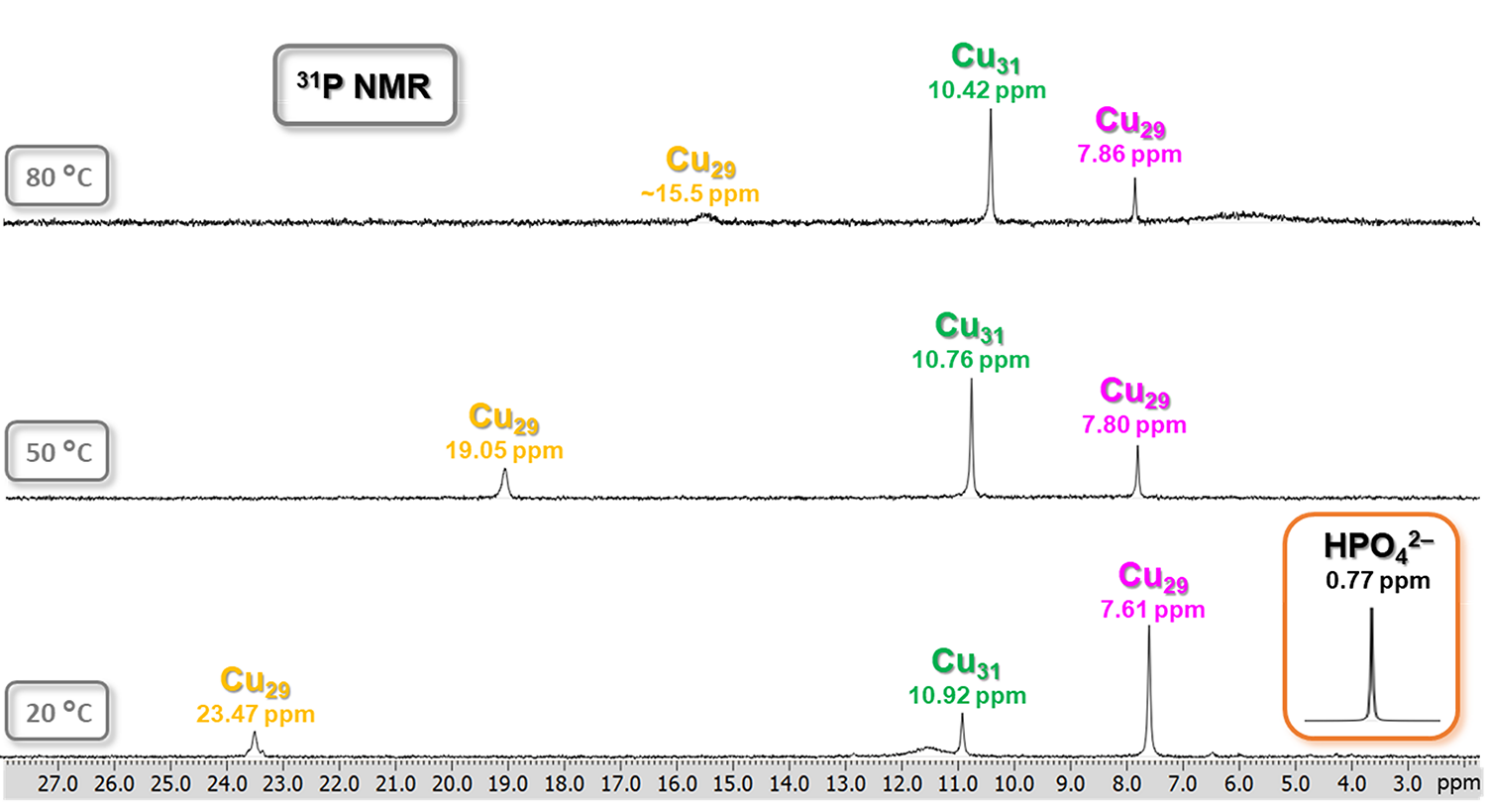
VT ^31^P NMR spectra of the **Cu**_***n***_ **HPO**_**4**_ (Cu_*n*_; *n* = 29, 31) nanojar mixture in DMSO-*d*_6_ (unreferenced). Color code for the Cu_29_ nanojars: yellow, Cu_7+13+9_; magenta, Cu_8+13+8_. The inset shows the signal of the free HPO_4_^2–^ ion as (Bu_4_N)_2_HPO_4_ in DMSO-*d*_6_ at 20 °C. Assignments were made based on correlations with ESI-MS (Figure S11) and ^1^H NMR spectra.

### UV–Vis Spectroscopy

The deep blue color of **Cu**_***n***_ **XPO**_**3**_ (R = HO, H, F; *n* = 27–31) nanojars originates from *d*–*d* transitions of the Cu^2+^ ions, which give rise to a peak in the UV–vis spectra in THF solutions at 600 nm (Figure 14). This color is identical to the one of the aqueous [Cu­(NH_3_)_4_(H_2_O)_2_]^2+^ ion (λ_max_ = 600 nm).^87^ The high extinction coefficients (*ε* = 3 × 10^3^ L mol^–1^ cm^–1^) of nanojars are due to the high density of chromophores (27–31 closely spaced Cu^2+^ ions). For comparison, a trinuclear copper pyrazolate complex with similar chromophores, Cu_3_(OH)­(pz)_3_(H_2_O)­(NO_3_)_2_, has an extinction coefficient (at λ_max_ = 630 nm) of only 0.16 × 10^3^ L mol^–1^ cm^–1^ in THF solution.^88^ In addition, a peak with absorption maximum at 349 nm is observed, which corresponds to ligand-to-metal charge transfer (*ε* = 3 × 10^4^ L mol^–1^ cm^–1^).

**Figure 14:**
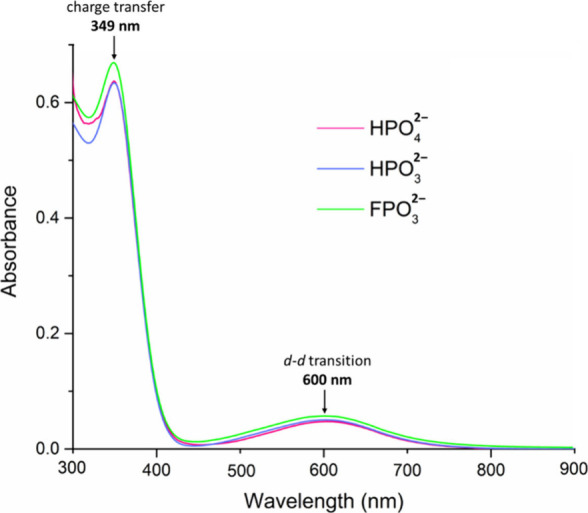
UV–vis spectra of **Cu**_***n***_ **XPO**_**3**_ (X = HO, H, F; *n* = 27–31) in THF (20 μM).

### Assessment of Phosphate, Phosphite and Fluorophosphate Binding Strength by Competitive Anion Binding

As with other anions, the binding constant of nanojars with XPO_3_^2–^ (X = OH, H, F) cannot be determined by host–guest titrations due to the fact that empty nanojar hosts cannot be isolated (nanojars form by self-assembly around the central anion). Alternatively, the strength of XPO_3_^2–^ binding was assessed by competitive binding experiments with Ba^2+^, which forms insoluble salts with these anions (for BaHPO_4_, *K*_sp_ = 2.75 × 10^–8^ in H_2_O at 38 °C).^89^ Since FPO_3_^2–^ is isoelectronic and isostructural with SO_4_^2–^, BaFPO_3_ is expected to have a very low solubility compared to the one of BaSO_4_ (*K*_sp_ = 1.08 × 10^–10^ in H_2_O at 25 °C).^90^ Although BaHPO_3_ is expected to be more soluble in water than BaFPO_3_, its solubility in 2-methyltetrahydrofuran (2-MeTHF) should be negligible. Therefore, competitive binding experiments were carried out under two different conditions: a) heterogeneously, by vigorously stirring a solution of **Cu**_***n***_ **XPO**_**3**_ in water-immiscible 2-MeTHF with an aqueous solution of Ba­(NO_3_)_2_, and b) homogeneously, by using barium dioctyl sulfosuccinate, Ba­(DOSS)_2_, which is soluble in 2-MeTHF together with the nanojar mixture. No BaXPO_3_ precipitate was observed in either case, and ESI-MS(−) of the organic layer shows no nanojar degradation products (such as low-nuclearity copper pyrazolate complexes). However, a diminishing in the amounts of **Cu**_**27**_ **XPO**_**3**_ and **Cu**_**29**_ **XPO**_**3**_ is observed, which apparently convert to **Cu**_**31**_ **XPO**_**3**_ in the presence of Ba^2+^ ions (Figures S12–S14). This conversion is most prominent in the case of the heterogeneous reaction with **Cu**_***n***_ **HPO**_**4**_, where only **Cu**_**31**_ **HPO**_**4**_ survives the treatment whereas the smaller analogues completely disappear. In contrast, the **Cu**_***n***_ **CO**_**3**_ species are much less affected (Figure S14).

### Liquid–Liquid Extraction of HPO_4_^2–^, HPO_3_^2–^ and FPO_3_^2–^ from Water into an Organic Solvent

The free energy of hydration (Δ*G*_h_°) of the highly hydrophilic HPO_4_^2–^ ion is −1089 kJ/mol, similar to the one of the SO_4_^2–^ ion (−1080 kJ/mol).^91,92^ Although experimental data is not available, the corresponding values for the HPO_3_^2–^ and FPO_3_^2–^ ions are expected to be very large as well. Therefore, the extraction of these ions from aqueous solutions into an organic solvent is expected to be challenging, and their liquid–liquid extraction has not yet been documented. We performed extraction experiments by stirring aqueous solutions of the sodium salts of those ions with nanojar ingredients (Cu­(NO_3_)_2_, pyrazole, NaOH and Bu_4_NOH) in THF. Although pure THF is miscible with pure water in all proportions, in the presence of inorganic salts the THF layer, which contains the hydrophobic nanojars, separates from the aqueous layer. Upon self-assembly, the **Cu**_***n***_ **HPO**_**4**_, **Cu**_***n***_ **HPO**_**3**_ and **Cu**_***n***_ **FPO**_**3**_ nanojars extract the corresponding anions from water into the THF layer which separates from the aqueous phase. Analysis of the organic phase by ESI-MS indicates the presence of **Cu**_***n***_ **HPO**_**3**_ (*n* = 27, 29, 31) in the case of HPO_3_^2–^, and almost exclusively **Cu**_**31**_ **HPO**_**4**_ or **Cu**_**31**_ **FPO**_**3**_ in the case of HPO_4_^2–^ and FPO_3_^2–^ (Figure S15). The extraction efficiencies (as measured by the obtained yields of nanojars after separating the THF layer and removing the solvent in vacuum) are 84%, 89% and 79% for HPO_4_^2–^, HPO_3_^2–^ and FPO_3_^2–^, respectively.

## Conclusions

In this work, we extended our studies of anion binding and extraction from water by nanojars to a class of inorganic, phosphorus-based anions, which are currently of high interest due to the shortage of phosphorus resources and the environmental impact caused by their use as fertilizer. Previous phosphorus anion binding studies have largely been focused on the singly charged H_2_PO_4_^–^ anion, which has a relatively low hydration energy of −473 kJ/mol.^91^ Thus, we explored the supramolecular binding of three different doubly charged phosphorus anions, phosphate (HPO_4_^2–^), phosphite (HPO_3_^2–^) and fluorophosphate (FPO_3_^2–^), and performed their extraction from water into an organic solvent using nanojars as extracting agents.

ESI-MS indicates that in the as-synthesized **Cu**_***n***_ **XPO**_**3**_ (X = HO, H or F) mixtures (by self-assembly from Cu^2+^, pyrazole, base and the anion) the major nanojar species are **Cu**_**27**_ **XPO**_**3**_, **Cu**_**29**_ **XPO**_**3**_ and **Cu**_**31**_ **XPO**_**3**_, except in the case of X = HO where **Cu**_**27**_ **HPO**_**4**_ forms in much smaller amounts. This might be due to the presence of the protonated O atom in HPO_4_^2–^, which is sterically more demanding than the nonprotonated HPO_3_^2–^ and FPO_3_^2–^ ions, disfavoring the smaller **Cu**_**27**_ nanojar. Smaller amounts of **Cu**_**28**_, **Cu**_**30**_, **Cu**_**32**_ and **Cu**_**33**_ species are also detected in solution. ^1^H NMR spectroscopy further reveals two different structural isomers for the **Cu**_**29**_ nanojar, **Cu**_**7+13+9**_ **XPO**_**3**_ and **Cu**_**8+13+8**_ **XPO**_**3**_. Of these, the former is kinetically favored and forms in much larger amounts during synthesis, whereas the latter, which is a minor component in the as-synthesized nanojar mixtures, is the thermodynamically more stable isomer as indicated by VT-NMR studies in DMSO-*d*_6_. Indeed, small amounts of **Cu**_**8+13+8**_ **XPO**_**3**_ persist even at 150 °C along with **Cu**_**8+14+9**_ **XPO**_**3**_ as the major component, whereas all other sizes break down on heating and convert to **Cu**_**31**_ **XPO**_**3**_. **Cu**_**31**_ appears to be the most stable nanojar size with all three XPO_3_^2–^ ions, corroborated by the observation that treatment with NH_3_ or Ba^2+^ ions also favors **Cu**_**31**_ species. The reactions with Ba^2+^ ions, which lead to no breakdown of the nanojars (other than conversion to the more stable species), suggest a very strong binding of the XPO_3_^2–^ ions which are not precipitated out even from organic solvents (such as 2-MeTHF) as the insoluble Ba salts. UV–vis spectroscopy indicates that the deep-blue color of nanojars caused by *d*–*d* transitions (λ_max_ = 600 nm) and the corresponding ligand-to-metal charge transfer (λ_max_ = 349 nm) are invariant of the entrapped anion.

Studies of anion exchange from CO_3_^2–^ to HPO_3_^2–^ or HPO_4_^2–^ by titration of **Cu**_***n***_ **CO**_**3**_ (*n* = 27, 29–31) with the corresponding acids show a different behavior for the two anions. Thus, with 10 equiv of H_3_PO_3_ in acetonitrile solution, all **Cu**_***n***_ **CO**_**3**_ species convert to **Cu**_***n***_ **HPO**_**3**_ (*n* = 27, 29, 31) and only **Cu**_**31**_ **HPO**_**3**_ is present with 15 equiv of H_3_PO_3_. Conversely, with 10 equiv of H_3_PO_4_, **Cu**_**29**_ **CO**_**3**_ is still present along with **Cu**_**31**_ **HPO**_**4**_, whereas with 15 equiv of H_3_PO_4_ all nanojars decompose despite the fact that H_3_PO_4_ is a weaker acid than H_3_PO_3_.

Single-crystal X-ray crystallography confirms the structures of various **Cu**_***n***_ **XPO**_**3**_ species detected in solution by ESI-MS and NMR, and provides the very first crystal structures with a supramolecularly bound FPO_3_^2–^ anion in a host–guest complex. No such structures have been reported with HPO_3_^2–^ either, except for a pseudopolymorph of **Cu**_**8+13+8**_ **HPO**_**3**_ presented herein. Both isomers of **Cu**_**29**_ **HPO**_**4**_ (**Cu**_**7+13+9**_ and **Cu**_**8+13+8**_) could be crystallized and their structures and anion binding patterns contrasted. Whereas in solution an x-fold symmetry is observed for each Cu_*x*_ ring, most crystal lattices studied here are triclinic (*P*1̅) and the nanojar moieties have no symmetry, except for **Cu**_**8+13+8**_ **XPO**_**3**_ (X = HO or H) which are found in monoclinic (*C*2/*c* or *P*2_1_/*c*) lattices and are located on *C*_2_ rotation axes.

The study of three different pseudopolymorphs for both **Cu**_**31**_ **HPO**_**3**_ and **Cu**_**31**_ **FPO**_**3**_ verifies consistency of the structure of a given nanojar crystallized from different solvent systems. Nevertheless, the different anions (HPO_3_^2–^ vs FPO_3_^2–^) and even the incorporation of different solvents of crystallization within the crystal lattice (in the case of **5c** compared to **5a** and **5b**) alter the resulting unit cells and overall crystal packing. Moreover, an additional H_2_O molecule is found in-between the nanojar and an adjacent Bu_4_N^+^ moiety in the **Cu**_**31**_ **HPO**_**3**_ pseudopolymorphs, similarly as in the **Cu**_**8+13+8**_ **XPO**_**3**_ (X = HO or H) species.

Multinuclear NMR studies unveil surprising changes in the magnetism of a given nanojar induced by different entrapped XPO_3_^2–^ (X = HO, H, F) anions, in spite of only slight changes observed in their structure by X-ray diffraction. While the effect of the paramagnetic Cu^2+^ centers on ^1^H NMR signals (especially of OH^–^ protons two bonds away compared to pz^–^ protons which are 3 or 4 bonds away) is obvious and rather consistent for different XPO_3_^2–^ anions, ^31^P NMR yields unexpected results. Specifically, widely different magnitudes of shift are observed for the signals of a given nanojar with the different anions, leading not only to disparate chemical shift windows, but even to a different order of the signals and a counterintuitive effect of the strongly electronegative F atom. For example, the ^31^P signals of **Cu**_**29**_ and **Cu**_**31**_ are at 58.46 and 100.00 ppm with HPO_3_^2–^, compared to 13.87 and 12.63 ppm with FPO_3_^2–^. These results clearly indicate that the solution phase structure of nanojars is significantly different from their corresponding solid-state structure, leading to rather different magnetic environments by changing the entrapped anion.

## Experimental Section

### General

All commercially available chemicals were used as received (solvents are ACS or HPLC grade, and THF is inhibited with 250 ppm BHT). Na_2_HPO_4_ (BioUltra, >99.5%), Na_3_PO_4_·12H_2_O (ACS reagent, >98%), Na_2_HPO_3_·5H_2_O (≥98%), Na_2_FPO_3_ (95%), H_2_FPO_3_ (70 wt % in H_2_O), Cu_2_(OH)­PO_4_ (>97%), Cu­(NO_3_)_2_·2.5H_2_O (ACS reagent, 98%) and NaOH (ACS reagent, 97%) were purchased from Sigma-Aldrich, pyrazole (99%) and ^*n*^Bu_4_NOH (55% in H_2_O) from Oakwood Chemical, and ^*n*^Bu_4_NOH (HPLC grade, 1.0 M in H_2_O) from Thermo Scientific. (Bu_4_N)_2_[CO_3_⊂{Cu­(OH)­(pz)}_*n*_] (Cu_*n*_CO_3_; *n* = 27, 29–31), [*trans*-Cu^II^(μ-OH)­(μ-pz)]_∞_ and Ba­(DOSS)_2_ were prepared according to the published procedures.^53,65,75^ The synthesis and reactions of nanojars were carried out under an N_2_(g) atmosphere (except for the liquid–liquid extraction experiments), and yields are based on the Cu­(II) starting materials. NH_3_(g) was generated by gently heating an NH_4_OH solution in a stoppered Erlenmeyer flask with a side arm, connected to a Pasteur pipet with Tygon 2375 tubing. Deionized water was freshly boiled and cooled to room temperature under N_2_(g). ^1^H (400 MHz), ^19^F (376 MHz) and ^31^P (162 MHz) NMR spectra were collected on a Jeol JNM-ECZS instrument, and UV–vis measurements were carried out on a Shimadzu UV-1650PC spectrophotometer.

### Synthesis of (Bu_4_N)_2_[XPO_3_⊂{Cu­(OH)­(pz)}_*n*_] (Cu_*n*_XPO_3_; X = OH, H or F; *n* = 27–31)

#### Method **a)**

Cu­(NO_3_)_2_·2.5H_2_O (1.000 g, 4.30 mmol) and pyrazole (0.293 g, 4.30 mmol) were dissolved in THF (20 mL) to obtain a clear, blue solution. Na_2_HPO_4_ (0.610 g, 4.30 mmol), Na_2_HPO_3_·5H_2_O (0.929 g, 4.30 mmol) or Na_2_FPO_3_ (0.619 g, 4.30 mmol) was added, followed by NaOH (0.333 g, 8.33 mmol) and ^*n*^Bu_4_NOH (55% in H_2_O; 0.135 g, 0.29 mmol). The reaction mixture was stirred in a stoppered flask at room temperature for 3 days. Then, a brown solid was filtered out and rinsed with THF (20 mL). Evaporation of the solvent in high vacuum affords a dark blue powder. Yields: 0.361 g (∼50%;X = OH), 0.614 g (∼85%;X = H) and 0.484 g (∼67%;X = F). The corresponding ESI-MS(−) spectra are shown in Figures 1 and S6.

#### Method **b)**

Cu­(NO_3_)_2_·2.5H_2_O (1.000 g, 4.30 mmol) and pyrazole (0.293 g, 4.30 mmol) were dissolved in THF (25 mL) to obtain a clear, blue solution, followed by the addition of (Bu_4_N)_2_HPO_4_ (2.498 g, 4.30 mmol) prepared *in situ* from H_3_PO_4_ (85% in H_2_O; 0.295 mL, 0.497 g, 4.30 mmol) and Bu_4_NOH (1 M in H_2_O; 8.60 mL, 8.60 mmol). Then, Bu_4_NOH (1 M in H_2_O; 8.60 mL, 8.60 mmol) was added dropwise under stirring. The dark blue solution was filtered and cannulated into H_2_O (500 mL) under stirring. The resulting blue precipitate was filtered out, washed with water (200 mL) and dried in high vacuum. Yield: 0.626 g (∼87%). The corresponding ESI-MS(−) spectrum is shown in Figure 1.

#### Method **c)**

To (Bu_4_N)_2_HPO_4_ obtained *in situ* by stirring Bu_4_NOH (1 M in H_2_O, 6.774 mL, 6.774 mmol) and H_3_PO_4_ (85% in H_2_O; 0.232 mL, 0.391 g, 3.387 mmol) in toluene (50 mL) was added [*trans*-Cu­(OH)­(pz)]_∞_ (0.5000 g, 3.387 mmol) and the mixture was refluxed overnight (14–16 h). The resulting reaction mixture was filtered, the solid was rinsed with toluene, and the solvent was removed from the deep blue filtrate under vacuum. The dark blue solid product was washed with water and was dried under vacuum. Yield based on [*trans*-Cu­(OH)­(pz)]_∞_ (for an average *n* = 30): 0.393 g (70%). The ESI-MS spectrum of the product is shown in Figure S6.

### Titration of (Bu_4_N)_2_[CO_3_⊂{Cu­(OH)­(pz)}_*n*_] (Cu_*n*_CO_3_; *n* = 27, 29–31) with Phosphoric Acid (H_3_PO_4_) and Phosphorous Acid (H_3_PO_3_)

A 1.0 × 10^–4^ M solution of nanojars was prepared by dissolving **Cu**_***n***_ **CO**_**3**_ (12.1 mg, 2.5 × 10^–3^ mmol, based on an average *n* = 29) in acetonitrile and diluting to volume in a 25 mL volumetric flask. 5.0 × 10^–3^ M H_3_PO_4_ and H_3_PO_3_ solutions were prepared by diluting 8.56 mL of H_3_PO_4_ (85% in H_2_O; 14.6 M) or 2.5 mL of a 5.0 × 10^–2^ M H_3_PO_3_ solution in H_2_O (prepared from 0.205 g, 2.5 × 10^–3^ mol H_3_PO_3_ in a 50 mL volumetric flask) with acetonitrile to volume in a 25 mL volumetric flask. Aliquots of the **Cu**_***n***_ **CO**_**3**_ solution (1 mL) were transferred into dram vials using a 1000 μL micropipette. Twenty μL of the 5.0 × 10^–3^ M acid solutions are required per 1 mL of the 1.0 × 10^–4^ M **Cu**_***n***_ **CO**_**3**_ solution for each molar equivalent. A series of solutions containing 0–15, 20, 25, 30, 60, and 100 mol equiv of acid were prepared. After the addition of the acid, the vials were capped, swirled, and allowed to stand overnight (approximately 14 h) at ambient conditions. The following day, any solids formed were filtered before analysis by ESI-MS. Precipitate formation was observed only for samples containing 15–100 mol equiv of acid.

### Reaction of Cu_*n*_XPO_3_ (R = OH, H or F; *n* = 27–33) with NH_3_

**Cu**_***n***_ **XPO**_**3**_ (0.100 g) was dissolved in THF (25 mL) and gaseous NH_3_ was bubbled through the resulting solution for 20 min. Then, the flask was stoppered and left standing. After 10 days, the solution was filtered and the solvent was evaporated to give a dark blue residue. ESI-MS(−) spectra of the products are shown in Figures S8–S10.

### Competitive Anion Binding under Heterogeneous Conditions

**Cu**_***n***_ **XPO**_**3**_ (X = OH, H or F; *n* = 27–33; 0.0400 g, 7.5–7.7 μmol) was dissolved in 2-MeTHF (5 mL) to give a clear, blue solution, which was then cannulated over a solution of Ba­(NO_3_)_2_ (0.0040 g, 15 μmol) in water (5 mL). After stirring vigorously for 1 h, the aqueous and organic layers were separated and the 2-MeTHF layer was analyzed by ESI-MS (Figures S12–S14).

### Competitive Anion Binding under Homogeneous Conditions

**Cu**_***n***_ **XPO**_**3**_ (X = OH, H or F; *n* = 27–33; 0.0400 g, 7.5–7.7 μmol) and Ba­(DOSS)_2_ (0.0076 g, 7.8 μmol) were dissolved in 2-MeTHF (10 mL) to give a clear, blue solution. The solution was stirred for 1 h and then it was analyzed by ESI-MS (Figures S12–S14).

### Extraction of XPO_3_^2–^ (X = OH, H, F) Ions from Water into THF

To a solution of sodium phosphate (4.30 mmol prepared from 0.294 mL of 85% H_3_PO_4_ and 0.183 g NaOH), Na_2_HPO_3_·5H_2_O (0.929 g; 4.30 mmol) or Na_2_FPO_3_ (0.619 g; 4.30 mmol) in H_2_O (10 mL) were added NaOH (0.344 g, 8.60 mmol) and Bu_4_NOH (1 M in H_2_O, 0.287 mL, 0.287 mmol). The resulting solution was stirred together vigorously with a solution of Cu­(NO_3_)_2_·2.5H_2_O (1.000 g, 4.30 mmol) and pyrazole (0.293 g, 4.30 mmol) in THF (10 mL) in a sealed flask. The deep-blue THF layer was separated from the aqueous layer using a separatory funnel, then it was filtered and evaporated. The blue solid residue was washed with H_2_O (150 mL) and dried in vacuum. Yield: 0.603, 0.638, and 0.567 g (84%, 89% and 79%, based on the corresponding nanojar with average *n* = 30). The ESI-MS(−) spectra of the products are shown in Figure S15.

### Mass Spectrometry

Mass spectrometric analysis of the nanojars was performed with a Waters Synapt G1 HDMS or a Waters Synapt XS instrument, using electrospray ionization (ESI). 10^–6^ M solutions were prepared in CH_3_CN using either solids or aliquots taken from solutions. Samples were infused by a syringe pump at 5 μL/min and nitrogen was supplied as the nebulizing gas at 500 L/h. The electrospray capillary voltage was set to −2.5 or +2.5 kV, respectively, with a desolvation temperature of 110 °C. The sampling and extraction cones were maintained at 40 and 4.0 V, respectively, at 80 °C. Reported *m*/*z* values represent averages of the observed isotopic distributions.

### X-ray Crystallography

All single-crystals were grown at room temperature by vapor diffusion of *n*-pentane (**3**, **4a** and **5a**), hexanes (**4b**) or *n*-heptane (**4c**) into a 1,2-dichlorobenzene solution, of *n*-pentane (**5b**) or *n*-heptane (**2**) into a chlorobenzene solution, of *n*-heptane (**1**) into a toluene solution, and of hexanes into a nitrobenzene/bromobenzene solution (**5c**) of **Cu**_***n***_ **XPO**_**3**_ (R = OH, H or F; *n* = 27–33). Once removed from the mother liquor, the crystals are extremely sensitive to solvent loss at ambient conditions and were quickly mounted under a cryostream (150 K) to prevent decomposition. X-ray diffraction data were collected from a single-crystal mounted atop a MiTeGen micromesh mount under Fomblin or polybutene oil with Bruker AXS D8 Quest diffractometers equipped with a Photon II charge-integrating and photon counting pixel array detector (CPAD) using graphite-monochromated Mo-*K*_α_ (λ = 0.71073 Å) radiation (for **4a**–**4c**, **5a**, **5c**) or a with Photon III C14 CPAD using either Cu-*K*_α_ (λ = 1.54178 Å) (for **1**–**3**) or Mo-*K*_α_ (λ = 0.71073 Å) radiation monochromated using X-ray mirror optics (for **5b**). The data were collected using APEX 3 or APEX4,^93^ integrated using SAIN^94^ and scaled and corrected for absorption and other effects using SADABS.^95^ The structure was solved by employing direct methods using ShelXS^96^ or ShelXT^97^ and refined by full-matrix least-squares on *F* ^2^ using ShelXL^98^ with ShelXle as the graphical interface.^99^ Refinement details and thermal ellipsoid plots (Figures S16–S24) are provided in the Supporting Information. Crystallographic figures were generated using CrystalMaker^100^ or Mercury (structural overlays),^101^ and supramolecular features (angles and distances) were measured using OLEX2.^102^

## Supplementary Material



## Notes

Additional data for mass spectrometry, X-ray crystallography and ^1^H NMR spectroscopy (PDF)
