# Supplementary material for: Supramolecular Binding and Extraction of Phosphate, Phosphite and Fluorophosphate Anions from Water by Nanojars
Source: Inorg Chem. 2026 Mar 3;65(10):5579–95. doi: 10.1021/acs.inorgchem.5c05810 (PMC12997160; doi:10.1021/acs.inorgchem.5c05810)
Supplement: Supplementary file 1 [file ic5c05810_si_001.pdf]

Supporting Information for

Supramolecular Binding and Extraction of Phosphate, Phosphite and Fluorophosphate Anions from Water by Nanojars

Wisam A. Al Isawi,[a] Angel S. Philip,[a] Pooja Singh,[a] Matthias Zeller[b] and Gellert Mezei[a]*

[a] *Department of Chemistry, Western Michigan University, Kalamazoo, Michigan 49008, USA*

[b] *Department of Chemistry, Purdue University, West Lafayette, Indiana 47907, USA*

* Corresponding author. Email: gellert.mezei@wmich.edu

CONTENTS **PAGE**

1. Characterization of the fluorophosphate precursors (Figures S1−S3) S2−S4
2. Mass spectrometric data (Figures S4−S15) S5−S13
3. X-ray crystallographic data and refinement details S13−S71
   (Figures S16−S38 and Tables S1−S34)
4. NMR spectroscopic data (Table S35, Figures S39–S43) S72−S78
5. References S79

1. CHARACTERIZATION OF THE FLUOROPHOSPHATE PRECURSORS

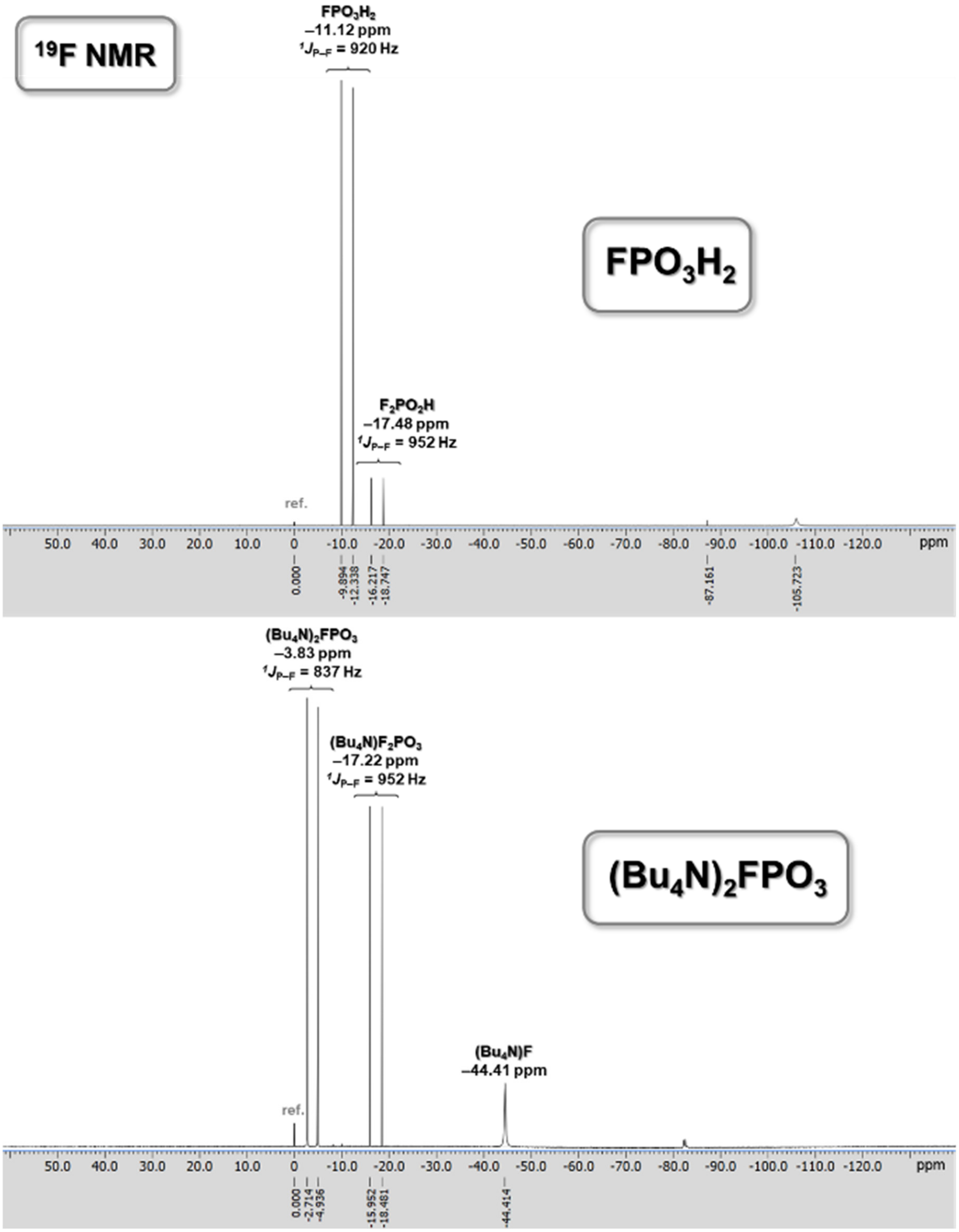

Figure S1. ^{19}F NMR spectra in DMSO-d_6 (at ambient temperature, referenced to $C_6H_5CF_3$) of fluorophosphoric acid (70% in H_2O) and the corresponding Bu_4N^+ salt obtained by neutralization with 2 equiv. of Bu_4NOH in H_2O.

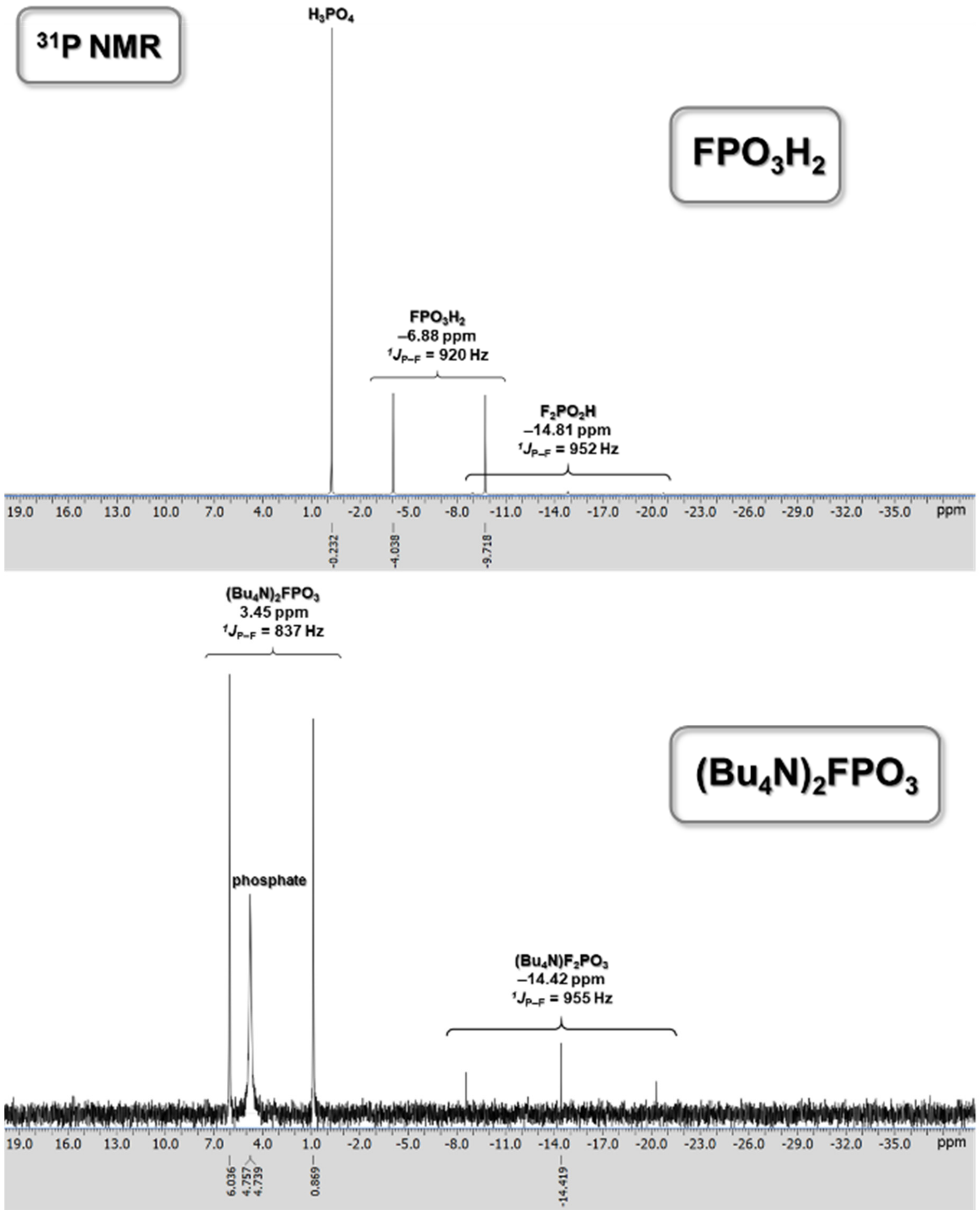

Figure S2. ^{31}P NMR spectra in DMSO-d_6 (at ambient temperature, without added reference) of fluorophosphoric acid (70% in H_2O) and the corresponding Bu_4N^+ salt obtained by neutralization with 2 equiv. of Bu_4NOH in H_2O.

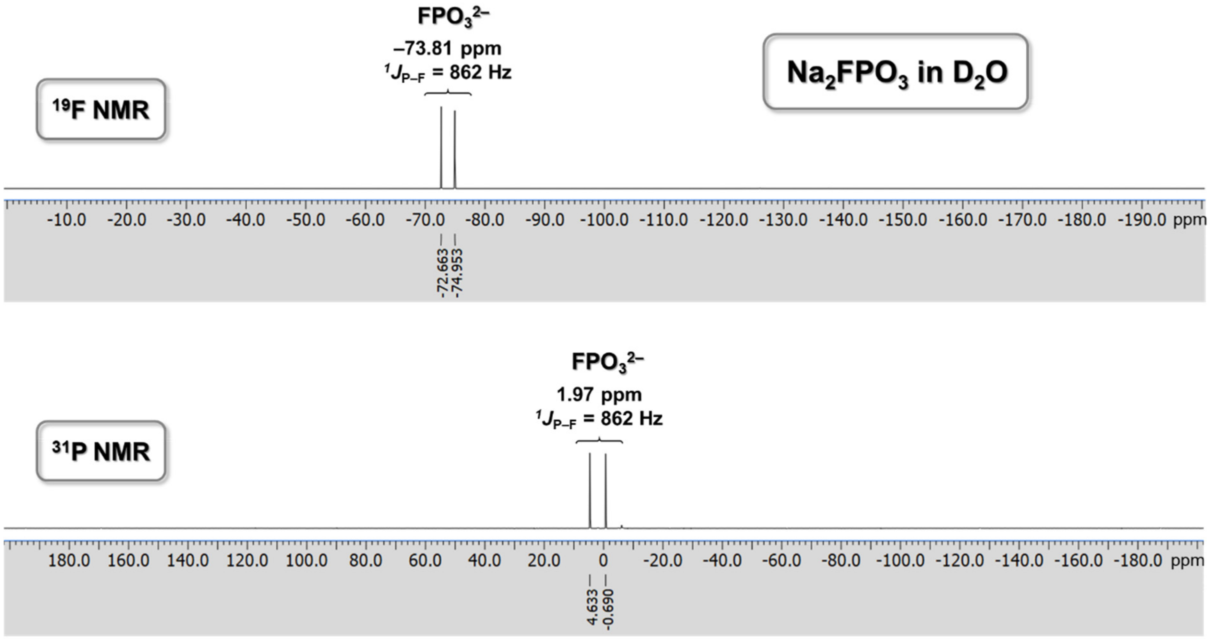

Figure S3. ^{19}F and ^{31}P NMR spectra in D_2O (at ambient temperature, without reference) of sodium fluorophosphate (Na_2FPO_3).

2. MASS SPECTROMETRIC DATA

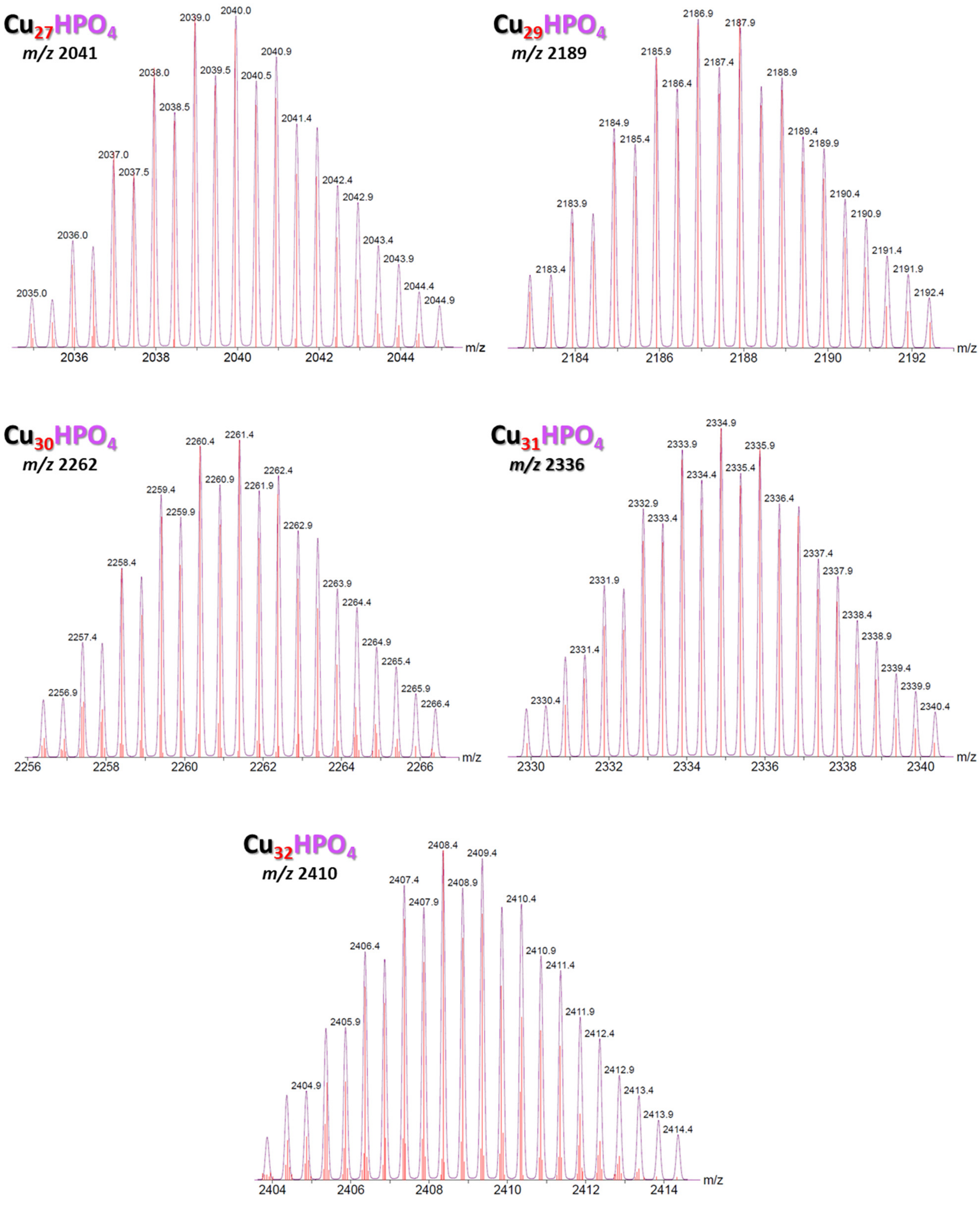

Figure S4. Isotopic distribution patterns observed (centroid) and predicted (continuum) for the major phosphate-entrapping nanojars.

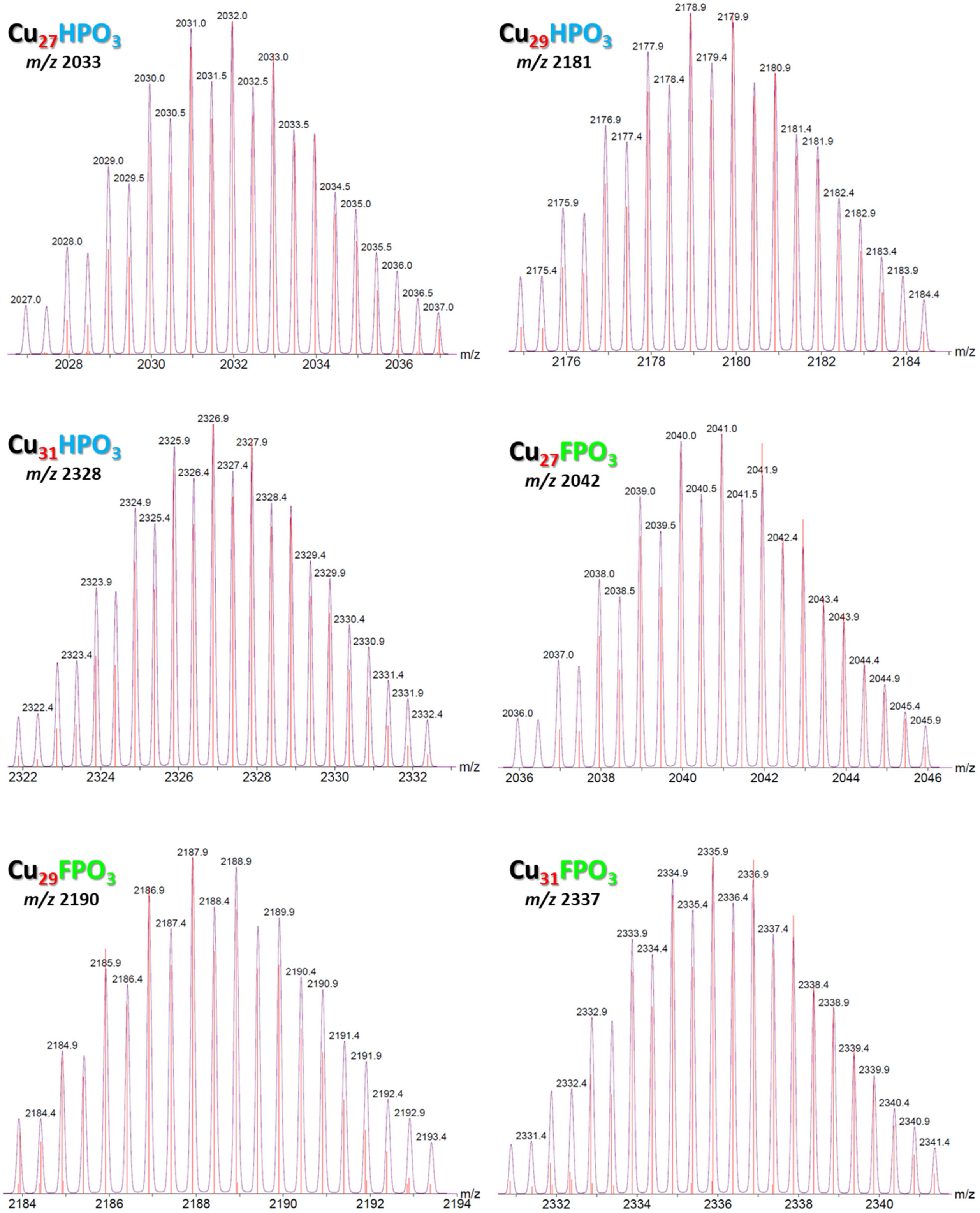

Figure S5. Isotopic distribution patterns observed (centroid) and predicted (continuum) for the major phosphite- and fluorophosphate-entrapping nanojars.

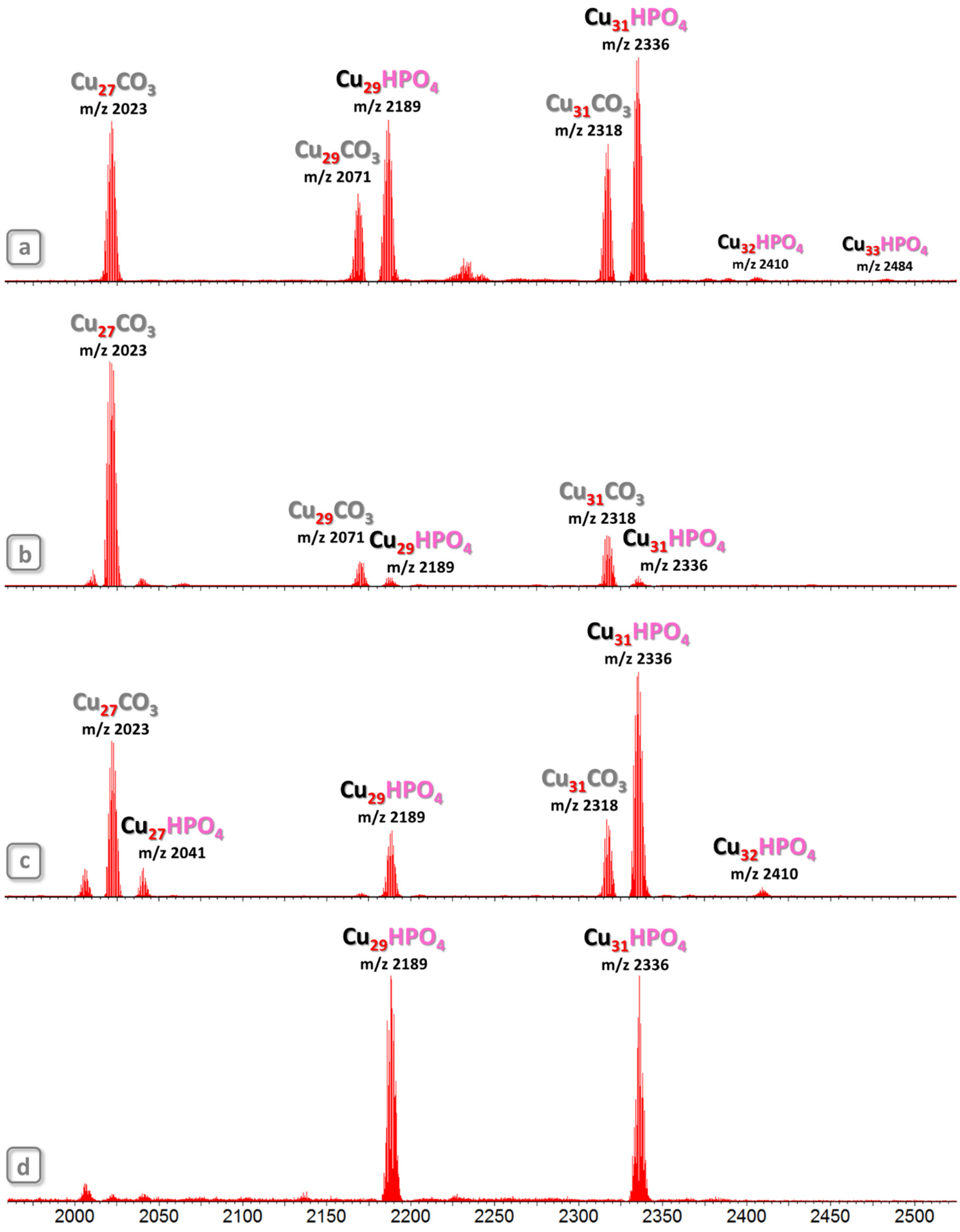

Figure S6. ESI-MS(−) spectra (in CH_3CN) of the mixture of phosphate nanojars $\mathbf{Cu_nHPO_4}$ (n = 27, 29, 31−33) and carbonate nanojars $\mathbf{Cu_nCO_3}$ (n = 27, 29, 31) obtained from a) $Cu(NO_3)_2$, pyrazole, NaOH, Bu_4NOH and Na_2HPO_4 in a 1:1:1.93:0.07:1 molar ratio in THF, b) $Cu(NO_3)_2$, pyrazole, NaOH and Na_3PO_4 in a 1:1:1.93:1 molar ratio in THF, c) $Cu_2(OH)(PO_4)$, pyrazole, NaOH and Bu_4NOH in a 0.5:1:1.4:0.07 molar ratio in THF, and d) depolymerization of $[Cu(OH)(pz)]_\infty$ by refluxing with *in-situ* prepared $(Bu_4N)_2HPO_4$ in a 1:1 molar ratio in toluene.

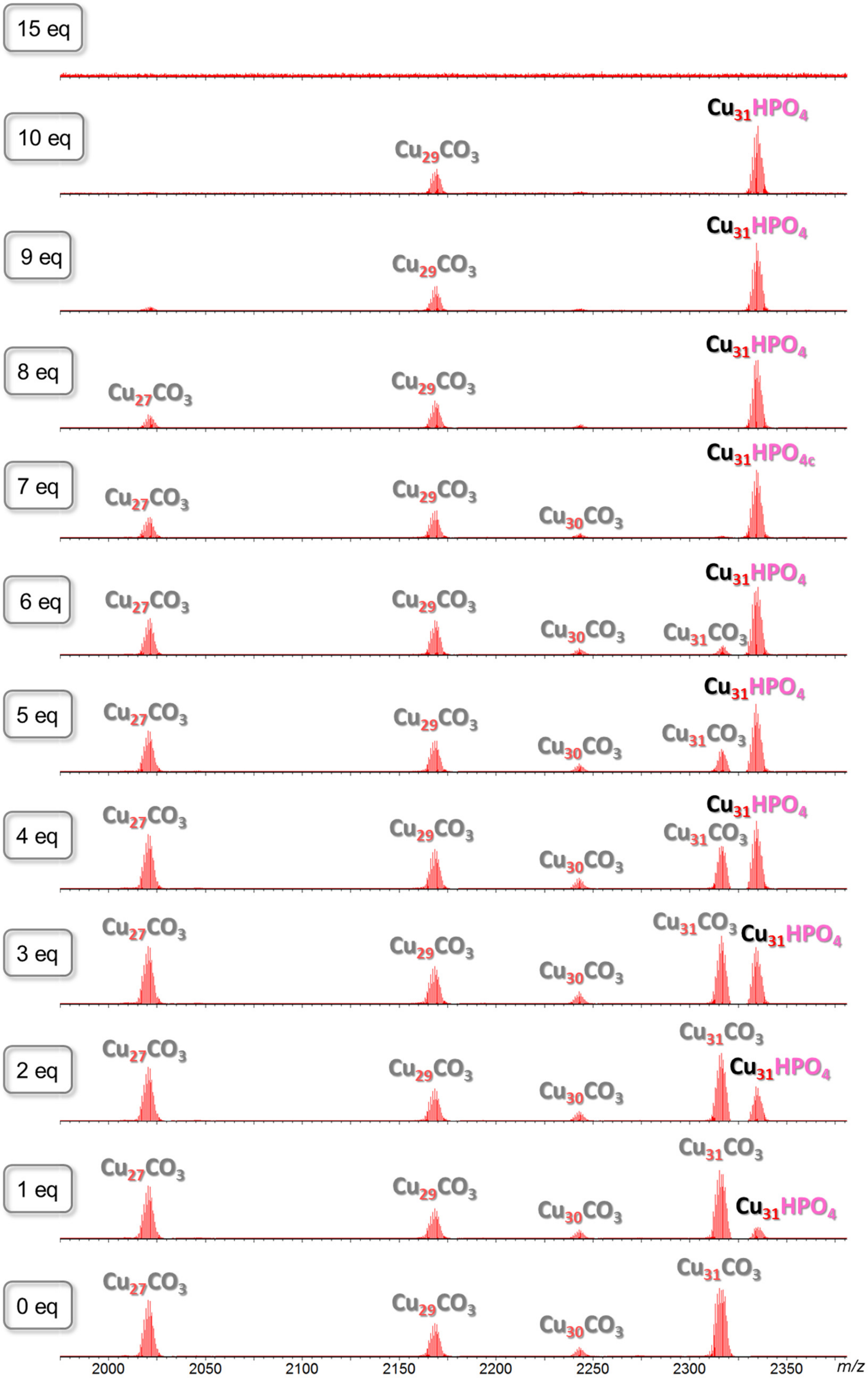

Figure S7. ESI-MS(−) spectra in CH_3CN of $\mathbf{Cu_nCO_3}$ (n = 27, 29–31) nanojars with varying amounts of added H_3PO_4.

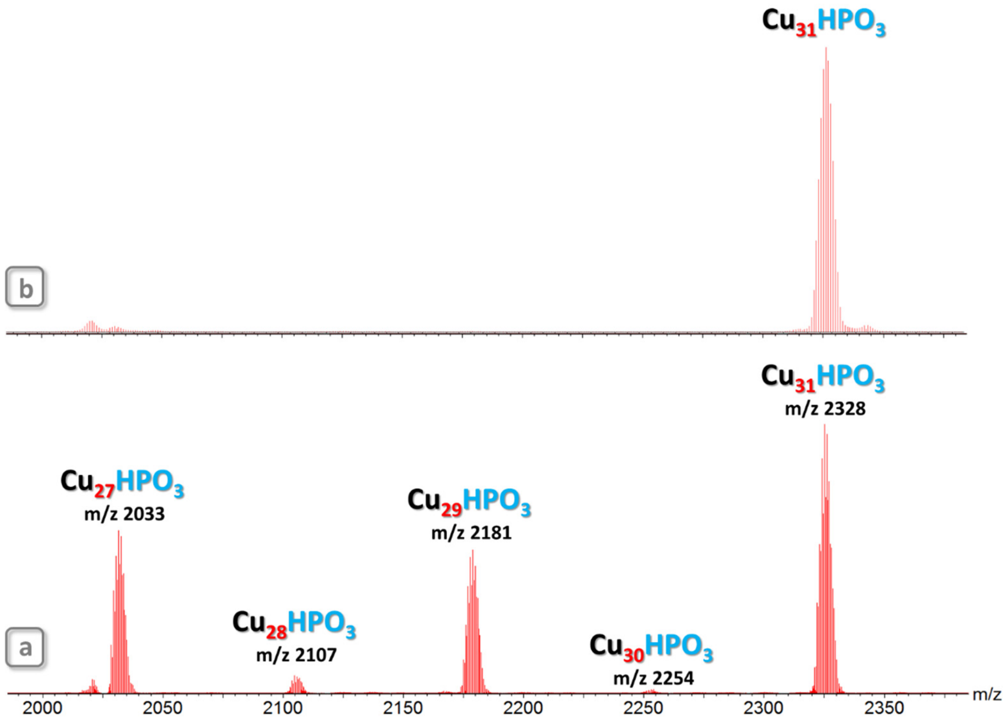

Figure S8. ESI-MS(−) spectra (in CH_3CN) of $[HPO_3 \subset \{Cu(OH)(pz)\}_n]^{2-}$ (n = 27−31): (a) before and (b) after treatment with NH_3(g) in a THF solution.

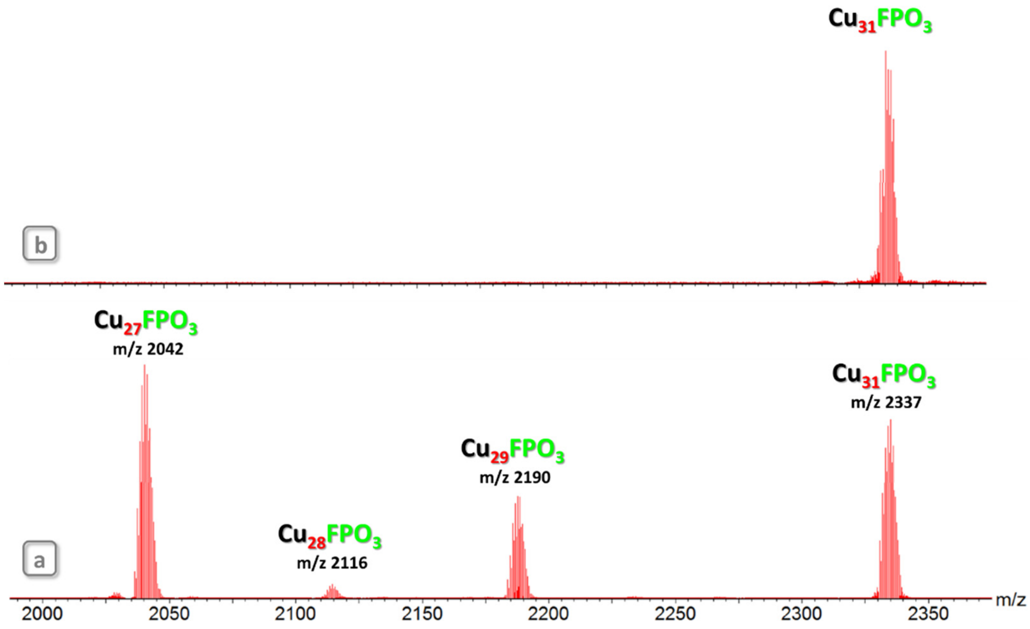

Figure S9. ESI-MS(−) spectra (in CH_3CN) of $[FPO_3 \subset \{Cu(OH)(pz)\}_n]^{2-}$ (n = 27−29, 31): (a) before and (b) after treatment with NH_3(g) in a THF solution.

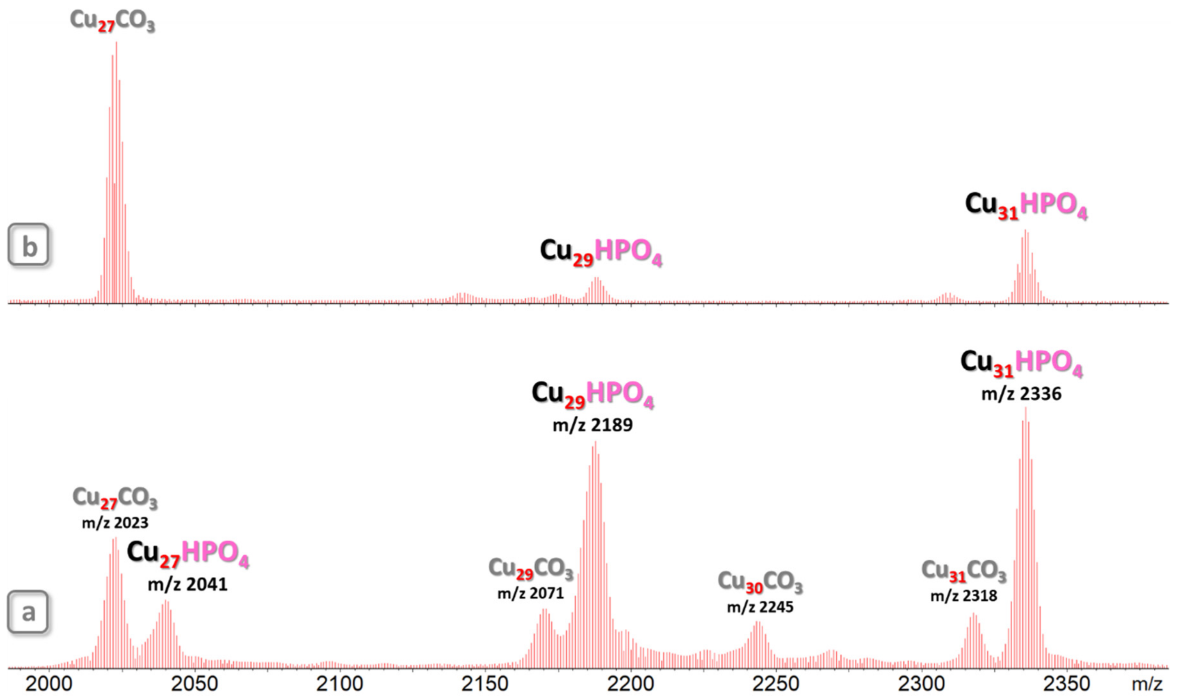

Figure S10. ESI-MS(−) spectra (in CH_3CN) of $[HPO_4 \subset \{Cu(OH)(pz)\}_n]^{2-}$ (n = 27, 29, 31): (a) before and (b) after treatment with NH_3(g) in a THF solution.

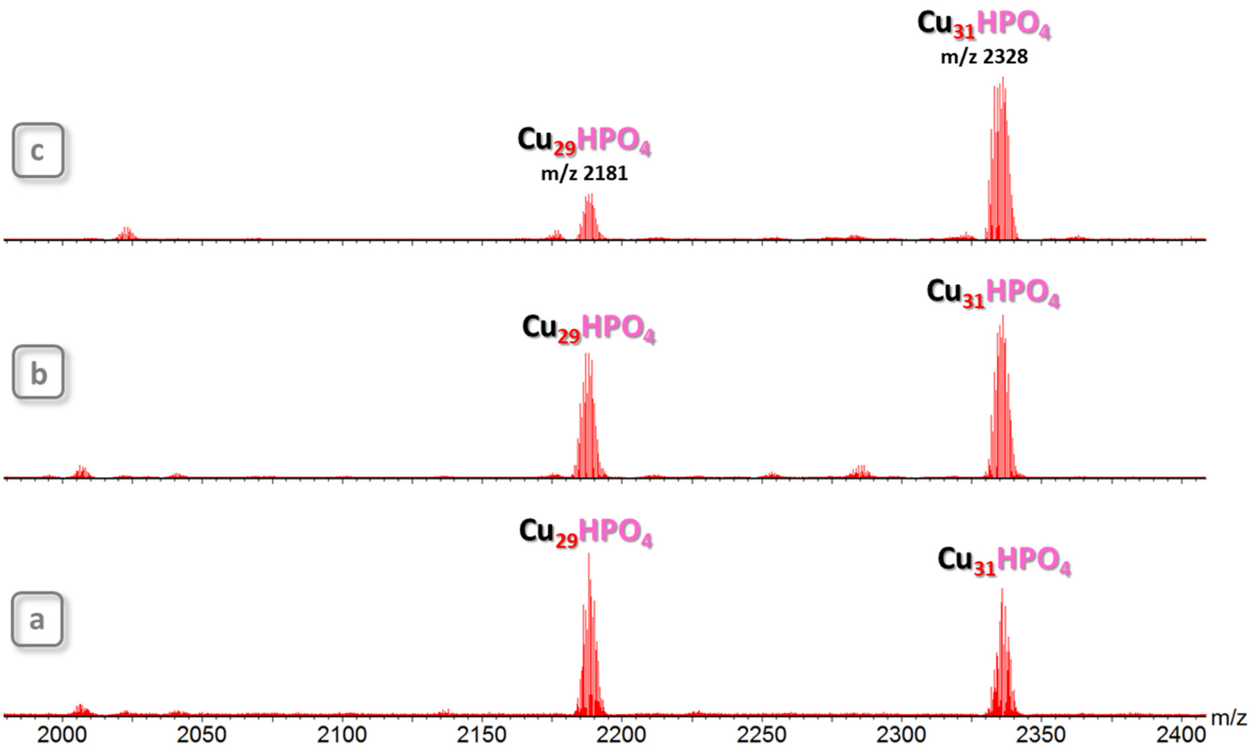

Figure S11. ESI-MS(−) spectra in CH_3CN of the $\mathbf{Cu_nHPO_4}$ (Cu_n; n = 29, 31) nanojar mixture a) before heating, b) after heating to 50 °C, and c) after heating to 80 °C in DMSO-d_6.

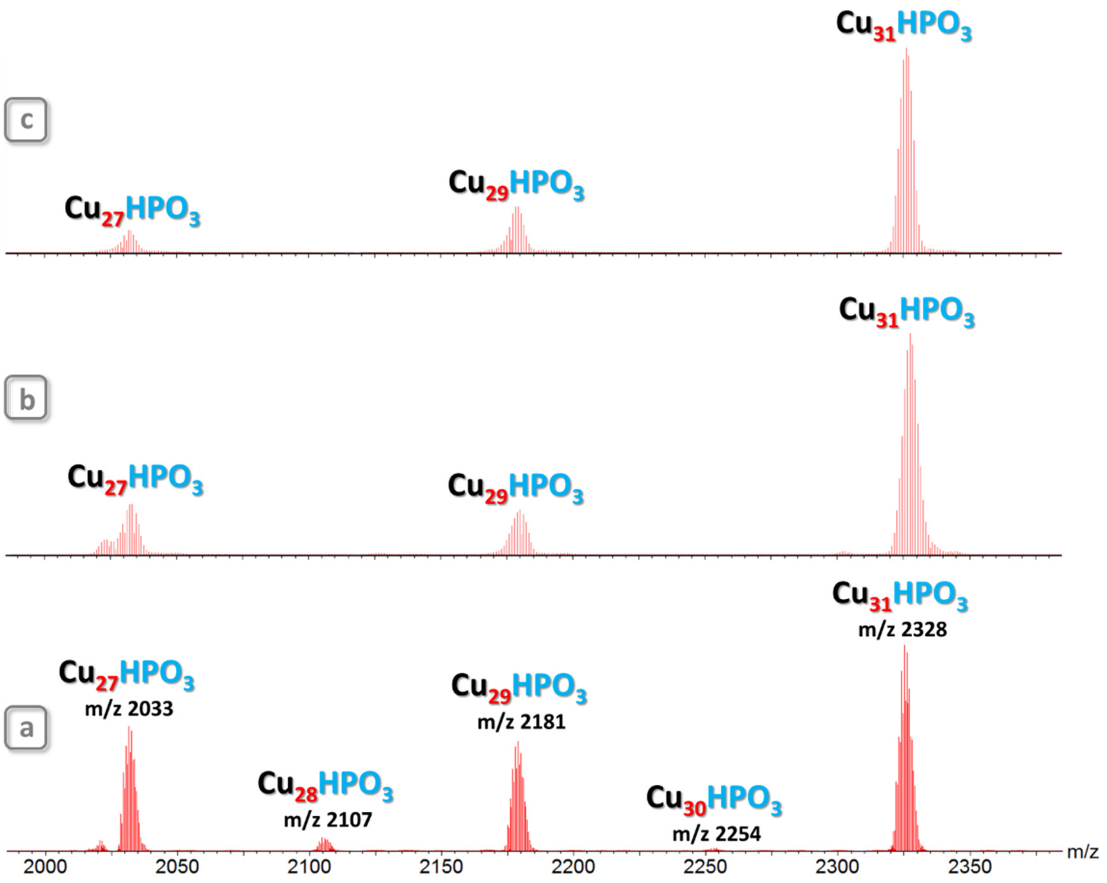

Figure S12. ESI-MS(−) spectra in CH_3CN of (a) the as-synthesized $[HPO_3\subset\{Cu(OH)(pz)\}_n]^{2-}$ (**Cu_nHPO_3**; n = 27−31) nanojar mixture, (b) the 2-MeTHF solution of **Cu_nHPO_3** after stirring for 1 h with an aqueous $Ba(NO_3)_2$ solution, and (c) the 2-MeTHF solution of **Cu_nHPO_3** containing $Ba(DOSS)_2$ after stirring for 1 h.

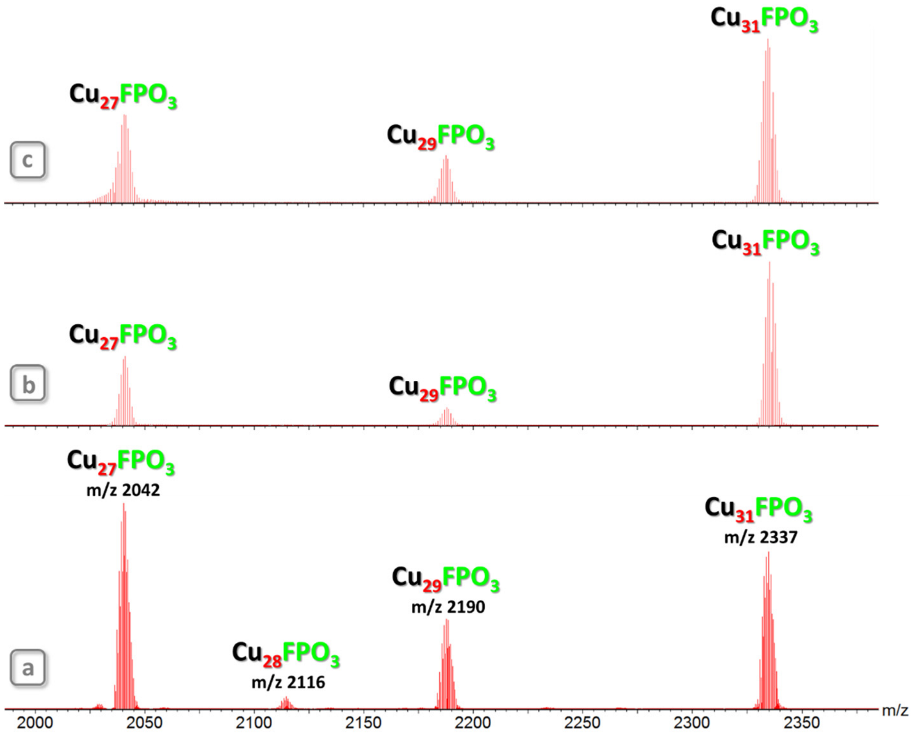

Figure S13. ESI-MS(−) spectra in CH_3CN of (a) the as-synthesized $[FPO_3\subset\{Cu(OH)(pz)\}_n]^{2-}$ (**Cu$_n$FPO$_3$**; n = 27−29, 31) nanojar mixture, (b) the 2-MeTHF solution of **Cu$_n$FPO$_3$** after stirring for 1 h with an aqueous $Ba(NO_3)_2$ solution, and (c) the 2-MeTHF solution of **Cu$_n$FPO$_3$** containing $Ba(DOSS)_2$ after stirring for 1 h.

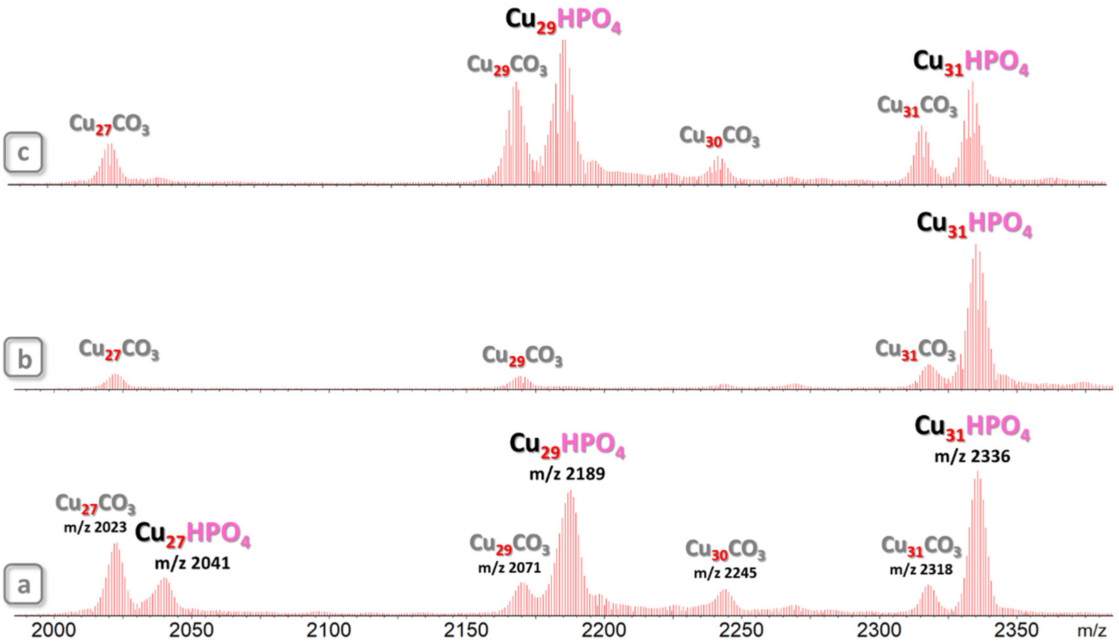

Figure S14. ESI-MS(−) spectra in CH_3CN of (a) the as-synthesized $[HPO_4\subset\{Cu(OH)(pz)\}_n]^{2-}$ (**Cu$_n$HPO$_4$**; n = 27−29, 31) nanojar mixture, (b) the 2-MeTHF solution of **Cu$_n$HPO$_4$** after stirring for 1 h with an aqueous $Ba(NO_3)_2$ solution, and (c) the 2-MeTHF solution of **Cu$_n$HPO$_4$** containing $Ba(DOSS)_2$ after stirring for 1 h.

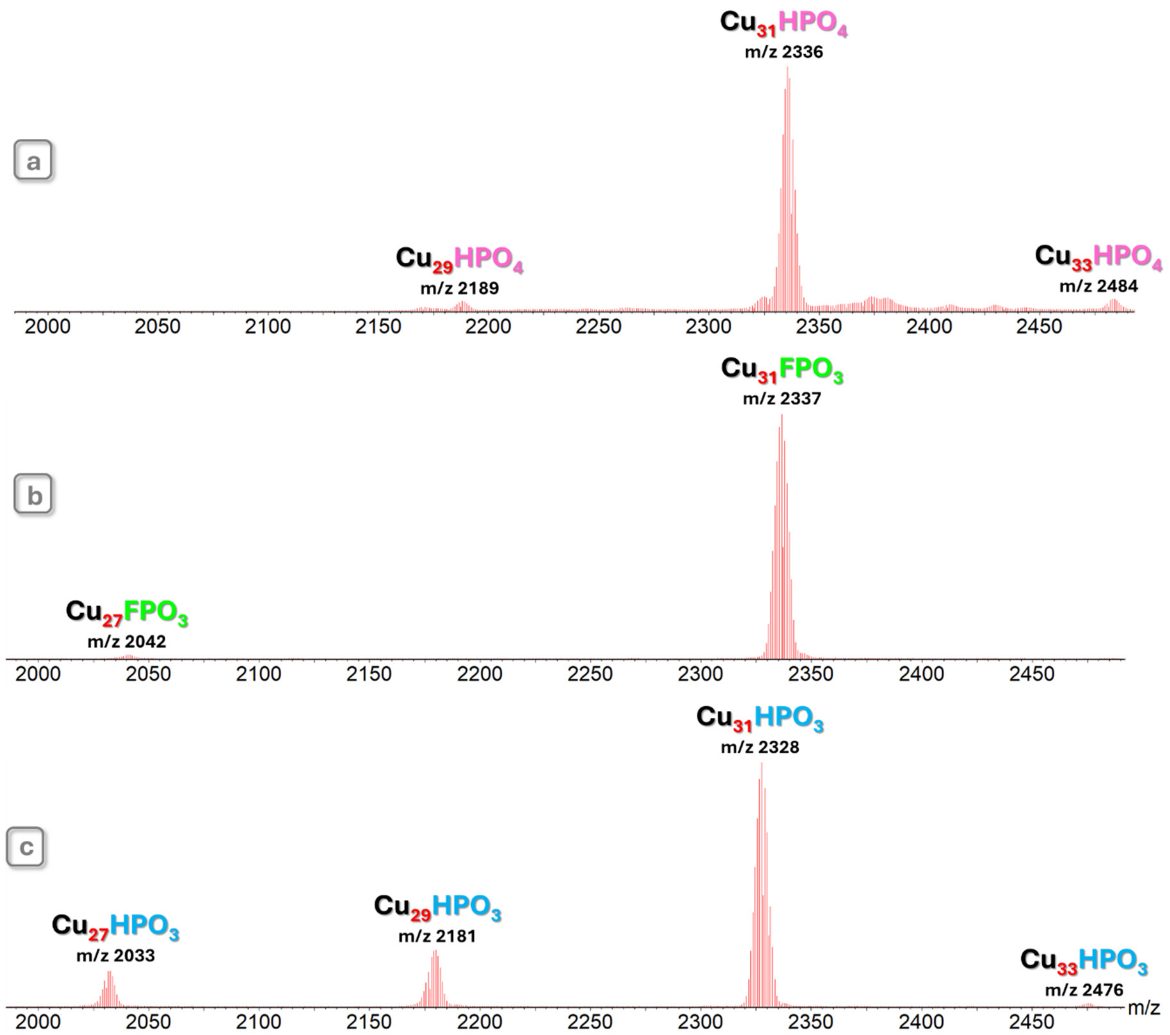

Figure S15. ESI-MS(−) spectra in CH_3CN of the $[XPO_3\subset\{Cu(OH)(pz)\}_n]^{2-}$ (**Cu_nXPO_3**; R = H, F, OH) obtained by the extraction of the (a) HPO_4^{2-}, (b) FPO_3^{2-} and (c) HPO_3^{2-} ions from water into THF.

3. X-RAY CRYSTALLOGRAPHIC DATA

1: H atoms of the OH groups of the nanojars were refined and O–H distances were restrained to 0.84(2) Å, respectively. One of the Bu_4N^+ counterions shows disorder of one of the butyl groups. The two disordered moieties were restrained to have similar geometries as another non-disordered butyl group. The other Bu_4N^+ ion was refined as disordered (whole-cation disorder). The two disordered moieties were restrained to have similar geometries as the other less-disordered Bu_4N^+ ion. U_{ij} components of ADPs for disordered atoms closer to each other than 2.0 Å were restrained to be similar. Subject to these conditions the occupancy ratio refined

to 0.477(13)/0.523(13) for the butyl group and 0.617(6)/0.383(6) for the whole-cation disorder.

Heptane and toluene solvate molecules are extensively disordered, with two- and three-fold disorder and overlapping disorder of neighboring solvate clusters. All heptane and all toluene moieties were restrained to have similar geometries. U_{ij} components of ADPs for disordered atoms closer to each other than 2.0 Å were restrained to be similar. The atoms of the two molecules of residues 11, 14 and 15 were also restrained to be close to isotropic. Subject to these conditions the occupancy rates of solvate molecules refined to the values given in the atom_site tables of the CIF.

2: Hydroxyl H atom positions were refined and O–H distances were restrained to 0.84(2) Å. Some hydroxyl H atom positions were further restrained based on hydrogen bonding considerations. The hydrogenphosphate dianion is disordered around a two-fold axis. The P–O bonds of the non-protonated O atoms were restrained to be similar. A half-occupied water molecule is associated with the phosphate disorder.

Three pyrazolate ligands, two copper atoms and one hydroxyl group are disordered around the same two-fold axis. One chlorobenzene solvate molecule is included in the disorder. Cu12 and the two surrounding pyrazolate ligands are independently disordered. All disordered pyrazolate ligands were restrained to have similar geometries as another well defined pyrazolate unit. Equivalent Cu–N and Cu–O bonds of disordered moieties were restrained to be similar. The chlorobenzene molecule was subjected to a rigid-bond restraint and to have a similar geometry as all other chlorobenzene molecules. U_{ij} components of ADPs for disordered atoms closer to each other than 2.0 Å were restrained to be similar. Subject to these conditions the occupancy ratio of Cu12 and the attached pyrazolates refined to 0.895(5)/0.105(5).

Two butyl groups of the Bu_4N^+ counterion were refined as disordered. One was refined as 1:1 disordered (due to proximity of chlorobenzene molecules disordered around inversion centers and two-fold axes). The other butyl group was refined as independently disordered. The disordered moieties were restrained to have a similar geometry as another better-defined butyl moiety. N–C bond distances of the cation were restrained to be similar. U_{ij} components of ADPs for disordered atoms closer to each other than 2.0 Å were restrained to be similar. Subject to

these conditions the occupancy ratio for the independently disordered butyl group refined to 0.676(11)/0.324(11).

Chlorobenzene solvate molecules are extensively disordered around inversion centers and two-fold axis. Positions are additionally split and partially occupied. All chlorobenzene molecules were restrained to have similar geometries. U_{ij} components of ADPs for disordered atoms closer to each other than 2.0 Å were restrained to be similar. Some molecules were also restrained to be close to planar and/or their atoms to be close to isotropic. Subject to these conditions the occupancy rates refined to the values given in the atom_site tables of the CIF.

3: Hydroxyl H atom positions were refined and O–H distances were restrained to 0.84(2) Å. The HPO_3^{2-} anion was refined as disordered by a pseudo-2-fold rotation axis. The two disordered moieties were restrained to have similar geometries. U_{ij} components of ADPs for disordered atoms closer to each other than 2.0 Å were restrained to be similar. Subject to these conditions the occupancy ratio refined to 0.630(7)/0.370(7).

A partially occupied water molecule is present in a void between the nanojar and one of the Bu_4N^+ cations. H atom positions were refined and O–H and H···H distances were restrained to 0.84(2) and 1.36 Å, respectively.

One of the pyrazole ligands was refined as disordered. The two disordered moieties were restrained to have similar geometries. Each moiety was restrained to be close to planar. U_{ij} components of ADPs for disordered atoms closer to each other than 2.0 Å were restrained to be similar. Subject to these conditions the occupancy ratio refined to 0.487(12)/0.513(12).

One of the Bu_4N^+ cations is partially disordered, the other exhibits whole cation disorder. The geometries of disordered equivalent moieties were restrained to be similar. U_{ij} components of ADPs for disordered atoms closer to each other than 2.0 Bu_4N^+ were restrained to be similar. Subject to these conditions the occupancy ratio refined to 0.525(7)/0.475(7) for the whole cation disorder (cation of N6), to 0.241(14)/0.759(14) for the butyl group of C88 to C91 (C88 was exempt from the disorder), and to 0.692(9)/0.308(9) for the butyl group of C100 to C103.

One chlorobenzene molecule was refined as disordered over two moieties. Two other chlorobenzene molecules were refined as disordered with a pentane molecule. Another chlorobenzene molecule was refined as disordered over two molecules as well as with a

pentane molecule. Two chlorobenzene molecules were refined as not disordered. All disordered chlorobenzene moieties were restrained to have similar geometries. Pentane bond lengths and angles were restrained to expected target values. For the triple disordered solvate, the two chlorobenzene moieties were restrained to be coplanar. Atoms C169 and C334 were constrained to have identical positions. Atoms C133, C169 and C334 as well as C132 and C333, and C137 and C332 were each constrained to have identical ADPs. U_{ij} components of ADPs for disordered atoms closer to each other than 2.0 Å were restrained to be similar. Subject to these conditions the occupancy ratio for the chlorobenzene molecule of Cl1/Cl2 refined to 0.810(7)/0.190(7). The occupancy ratio for the chlorobenzene/pentane disorder refined to 0.503(5)/0.497(5) for Cl9/Cl10 and to 0.428(6)/0.572(6) for Cl11/Cl12. The chlorobenzene/chlorobenzene/pentane disorder (involving the chlorobenzene molecule of Cl5/Cl6) refined to 0.610(3)/0.141(3)/0.249(3).

4a: Hydroxyl and water H atom positions were refined. O–H distances were restrained to 0.84(2) Å, water H···H positions to 1.36(2) Å. Several hydroxyl H atom positions were further restrained based on hydrogen bonding considerations.

Two of the pyrazole ligands (pyrazole of N49/N50 and N51/N52) were refined as disordered. The disordered moieties were restrained to have similar geometries as another, not disordered pyrazole ligand. Each moiety was restrained to be close to planar. U_{ij} components of ADPs for disordered atoms closer to each other than 2.0 Å were restrained to be similar. Subject to these conditions the occupancy ratios refined to 0.504(11)/0.496(11) and 0.523(11)/0.477(11).

The HPO_3^{2-} anion was refined as disordered. The two moieties were restrained to have similar geometries. U_{ij} components of ADPs for disordered atoms closer to each other than 2.0 Å were restrained to be similar. ADPs of O34 and O34B were constrained to be identical. Subject to these conditions the occupancy ratio refined to 0.854(11)/0.146(11).

For one of the Bu_4N^+ cations two butyl groups are disordered. The geometries of the two disordered moieties were restrained to be similar to an equivalent non-disordered moiety. The other Bu_4N^+ cation is disordered over four positions. The central two moieties are symmetry equivalent by inversion and overlap with each other and are thus mutually exclusive. Each of the two central cation moieties do overlap with one of the two other cation positions (which are also symmetry equivalent with each other). All four moieties were thus refined as half-

occupied. The outer two cation moieties are disordered with a half-occupied dichlorobenzene solvate molecule. The half-occupied Bu_4N^+ cation moieties were restrained to be similar to the major moiety of the other cation. Subject to these conditions the occupancy ratios for the Bu_4N^+ cation in the general position refined to 0.69(2)/0.31(2) (butyl of C94) and 0.59(2)/0.41(2) (butyl of C106).

A 1,2-dichlorobenzene molecule was refined as partially occupied (Cl3/Cl4). A series of four 1,2-dichlorobenzene molecules were refined as disordered with each other. All 1,2-dichlorobenzene molecules were restrained to have similar geometries. For the partially occupied moiety, a rigid bond restraint was applied (RIGU) and the atoms were restrained to be close to isotropic. Subject to these conditions the occupancy rate refined to 0.616(10). No other interpretable electron density is present at the site. For the disordered 1,2-dichlorobenzene moieties U_{ij} components of ADPs for disordered atoms closer to each other than 2.0 Å were restrained to be similar. The total occupancy for the site was constrained to unity. Subject to these conditions the occupancy rates refined to 0.382(3) (Cl5/Cl6), 0.251(3) (Cl7/Cl8), 0.199(3) (Cl9/Cl10) and 0.168(3) (Cl13/Cl14).

The structure contains additional 557 Å^3 of solvent accessible voids. Difference densities indicate substantially disordered pentane and/or 1,2-dichlorobenzene molecule. The largest electron density peaks were less than 2.62 e$^-$/Å^3 and the residual electron density peaks are not arranged in an interpretable pattern. The structure factors were instead augmented via reverse Fourier transform methods using the SQUEEZE routine (P. van der Sluis & A.L. Spek (1990). Acta Cryst. A46, 194-201) as implemented in the program Platon. The resultant FAB file containing the structure factor contribution from the electron content of the void space was used together with the original HKL file in further refinement. The FAB file with details of the SQUEEZE results is appended to the CIF file. The SQUEEZE procedure corrected for 125 electrons within the solvent accessible voids.

4b: The structure was solved by isomorphous replacement from its counterpart without acetate admixture (**30b**). Hydroxyl and water H atom positions were refined. O–H distances were restrained to 0.84(2) Å, water H···H positions to 1.36(2) Å.

One of the pyrazole ligands (pyrazole of N49/N50) was refined as disordered. Two of the pyrazole ligands (pyrazole of N5/N6, N57/N58) were refined as disordered with an acetate ion.

One other pyrazole ligand (pyrazole of N51/N52) was refined as disordered with both an acetate and another pyrazole ligand. Disordered pyrazole moieties were restrained to have similar geometries as another, not disordered pyrazole ligand. Each pyrazole moiety was restrained to be close to planar. Disordered acetate moieties were restrained to have similar geometries. U_{ij} components of ADPs for disordered atoms closer to each other than 2.0 Å were restrained to be similar. Subject to these conditions the occupancy ratios refined to 0.491(7)/0.509(7) for the pyrazole of N49/N50, 0.452(3)/0.366(3)/0.183(3) for N51/N52, N51B/N52B and the acetate disordered with them. The pyrazole to acetate ratios refined to 0.226(8)/0.774(8) for the pyrazole of N5/N6 (in favor of acetate) and to 0.641(8)/0.359(8) for the pyrazole of N17/N18 (in favor of pyrazole).

The HPO_3^{2-} anion was refined as disordered. The two moieties were restrained to have similar geometries. ADPs of O33 and O33B, and of O34 and O34B were constrained to be identical. Subject to these conditions the occupancy ratio refined to 0.747(11)/0.253(11).

Both Bu_4N^+ cations are disordered. For the cation of N63, all butyl chains are disordered. U_{ij} components of ADPs for disordered with three chains sharing a common occupancy ratio and the forth being independently disordered. The other Bu_4N^+ cation is disordered over four positions. The central two moieties are symmetry equivalent by inversion and overlap with each other and are thus mutually exclusive. Each of the two central cation moieties do overlap with one of the two other cation positions (which are also symmetry equivalent with each other). All four moieties were thus refined as half-occupied. The outer two cation moieties are disordered with a half-occupied dichlorobenzene solvate molecule. For the cation of N63, all butyl chains were restrained to have similar geometries and the N–C bonds were restrained to be similar to each other. The two half-occupied Bu_4N^+ cation moieties were restrained to be similar to the major moiety of the other cation. Disordered atoms closer to each other than 2.0 Å were restrained to be similar. Subject to these conditions the occupancy ratios for the Bu_4N^+ cation in the general position refined to 0.664(5)/0.336(5) (butyl groups of C94, 97 and 102) and 0.772(13)/0.228(13) (butyl group of C106).

A 1,2-dichlorobenzene molecule was refined as disordered and partially occupied (Cl3/Cl4, Cl3B/Cl4B). A series of four 1,2-dichlorobenzene molecules were refined as disordered with each other. All 1,2-dichlorobenzene molecules were restrained to have similar geometries. The total occupancy for the site was constrained to unity. U_{ij} components of ADPs for disordered

atoms closer to each other than 2.0 Å were restrained to be similar. Subject to these conditions the occupancy rates refined to 0.347(7) (Cl3/Cl4), 0.247(6) (Cl3B/Cl4B), 0.138(3) (Cl5/Cl6), 0.377(3) (Cl7/Cl8), 0.185(3)(Cl9/Cl10) and 0.300(3) (Cl13/Cl14).

The structure contains additional 532 Å^3 of solvent-accessible voids. Difference densities indicate substantially disordered pentane and/or 1,2-dichlorobenzene molecule. The largest electron density peaks were less than 2.62 e$^-$/Å^3 and the residual electron density peaks are not arranged in an interpretable pattern. The structure factors were instead augmented via reverse Fourier transform methods using the SQUEEZE routine (P. van der Sluis & A.L. Spek (1990). Acta Cryst. A46, 194-201) as implemented in the program Platon. The resultant FAB file containing the structure factor contribution from the electron content of the void space was used together with the original HKL file in further refinement. The FAB file with details of the SQUEEZE results is appended to the CIF file. The SQUEEZE procedure corrected for 104 electrons within the solvent accessible voids.

4c: Hydroxyl H atom positions were refined and O–H distances were restrained to a target value of 0.84(2) Å. H5O and H27O were further restrained based on hydrogen bonding considerations (H5O to be 2.60(2) Angstrom from O32; H27O to have equal distances to O32 and O34). The water H atoms were restrained to be 1.36 Å apart from each other.

The HPO_3^{2-} anion was refined as two-fold disordered. The geometries of the two moieties were restrained to be similar. U_{ij} components of ADPs for disordered atoms closer to each other than 2.0 Å were restrained to be similar. Subject to these conditions, the occupancy ratio refined to 0.767(13)/0.233(13).

Two pyrazole ligands (of N59 and N61) were refined as disordered. The disordered moieties were restrained to have similar geometries as another better-defined pyrazole ligand. U_{ij} components of ADPs for disordered atoms closer to each other than 2.0 Å were restrained to be similar. Subject to these conditions, the occupancy ratio refined to 0.449(12)/0.551(12) and 0.508(10)/0.492(10).

One of the Bu_4N^+ cations (N63) exhibits minor disorder of one butyl chain. The other cation is disordered over two half-occupied positions with some overlap (and thus mutually exclusive). One relatively well-defined half-occupied cation (N64) is located in the "bowl" of the nanojar,

and is disordered around an inversion center with its own counterpart and are mutually exclusive. The other of these two cation positions (N65) is shifted away from the nanojar into a region of a disordered dichlorobenzene solvate molecule.

For the first Bu_4N^+ cation (N63) atoms C103 through C105 were refined as disordered and were restrained to have similar geometries as another better-defined fragment of the same kind. The two half-occupied Bu_4N^+ cations (of N64 and N65) were restrained to have a similar geometry as the major moiety of the first cation. U_{ij} components of ADPs for disordered atoms closer to each other than 2.0 Å were restrained to be similar. Subject to these conditions the occupancy ratio for C103 through C105 of the Bu_4N^+ cation in the general position (N63) refined to 0.49(2)/0.51(2).

A 1,2-dichlorobenzene molecule is disordered with two of the butyl chains of the cation of N65. The 1,2-dichlorobenzene molecule is further disordered over two orientations. Another 1,2-dichlorobenzene molecule was refined as three-fold disordered, and a third 1,2-dichlorobenzene molecule was refined as disordered with a heptane molecule. Another heptane molecule was refined as two-fold disordered, with the minor moiety being incompatible with its counterpart by inversion. All 1,2-dichlorobenzene molecules were restrained to have similar geometries. Some 1,2-dichlorobenzene benzene rings were constrained to resemble ideal hexagons with C–C distances of 1.39 Å and some were restrained to be close to planar. U_{ij} components of ADPs for disordered atoms closer to each other than 2.0 Å were restrained to be similar. Atoms of the disordered heptane molecule were also restrained to be close to isotropic. Subject to these conditions, the occupancy ratios refined to the values as given in the CIF file.

5a: The crystal under investigation was found to be non-merohedrally twinned. The orientation matrices for the two components were identified using the program Cell_Now, with the two components being related by a 180° rotation around the reciprocal axis (0 1 −1). The two components were integrated using SAINT and corrected for absorption using TWINABS, resulting in the following statistics:

17419 data (6789 unique) involve domain 1 only, mean I/σ 35.6

17080 data (6120 unique) involve domain 2 only, mean I/ σ 32.8

308872 data (50202 unique) involve 2 domains, mean I/ σ 24.9

1578 data (1304 unique) involve 3 domains, mean I/ σ 15.3

4 data (3 unique) involve 4 domains, mean I/ σ 2.2

3 data (1 unique) involve 6 domains, mean I/ σ 86.1

The exact twin matrix identified by the integration program was found to be:

$$\begin{matrix} -0.99924 & 0.00166 & 0.00083 \\ 0.40991 & 0.22369 & -0.77733 \\ -0.41180 & -1.22240 & -0.22444 \end{matrix}$$

The structure was solved using direct methods with only the non-overlapping reflections of component 1. The structure was refined using the HKLF 5 routine with all reflections of component 1 (including the overlapping ones), resulting in a BASF value of 0.4104(7).

The R_{int} value given is for all reflections and is based on agreement between observed single and composite intensities and those calculated from refined unique intensities and twin fractions (TWINABS (Sheldrick, 2012)).

Hydroxyl H atom positions were refined and O–H distances were restrained to 0.84(2) Å. Several hydroxyl H atom positons were further restrained based on hydrogen bonding considerations.

Five pyrazole ligands were refined as disordered. For each the two disordered moieties were restrained to have similar geometries. Each moiety was restrained to be coplanar with the copper ions it is bonded to. U_{ij} components of ADPs for disordered atoms closer to each other than 2.0 Å were restrained to be similar. Subject to these conditions the occupancy ratio refined to 0.64(2)/0.36(2) (pyrazole of N17), 0.722(14)/0.278(14) (pyrazole of N23), 0.58(3)/0.42(3) (pyrazole of N39), 0.34(3)/0.66(3) (pyrazole of N41) and 0.677(18)/0.323(18) (pyrazole of N43).

Of the two Bu_4N^+ cations one exhibits minor disorder of one of the butyl chains. The other exhibits whole cation disorder. The two moieties of the single disordered chain were restrained to have a similar geometry as another non-disordered butyl chain. The two moieties of the disordered cation were restrained to have a similar geometry as the other less disordered cation. U_{ij} components of ADPs for disordered atoms closer to each other than 2.0 Å were restrained to be similar. Subject to these conditions the occupancy ratio refined to

0.61(3)/0.39(3) for the disordered butyl chain, and to 0.526(14)/0.474(14) for the whole cation disorder.

One 1,2-dichlorobenzene molecule was refined disordered. Three other 1,2-dichlorobenzene molecules were refined as partially occupied. All 1,2-dichlorobenzene molecules were restrained to have similar geometries and all benzene rings were constrained to resemble ideal hexagons with C–C bond distances of 1.39 Å. No attempts were made to ensure full occupancy for all solvate sites. Minor ill-defined residual electron density, especially around some 1,2-dichlorobenzene molecules, was ignored. Subject to these conditions the occupancy ratio refined to 0.575(14)/0.425(14) for the disordered 1,2-dichlorobenzene molecule (Cl1, Cl2), and the occupancy rates for the others refined to 0.596(12), 0.508(12) and 0.474(15).

The structure contains additional 1059 $Å^3$ of solvent-accessible voids. No substantial electron density peaks were found in the solvent-accessible voids (less than 2 $e^-/Å^3$) and the residual electron density peaks are not arranged in an interpretable pattern. The structure factors were instead augmented via reverse Fourier transform methods using the SQUEEZE routine (P. van der Sluis & A.L. Spek (1990). Acta Cryst. A46, 194-201) as implemented in the program Platon. The resultant FAB file containing the structure factor contribution from the electron content of the void space was used together with the original HKL file in further refinement. The FAB file with details of the SQUEEZE results is appended to the CIF file. The SQUEEZE procedure corrected for 271 electrons within the solvent-accessible voids.

5b: The crystal structure was solved from its 1,2-dichlorobenzene solvate analogue by isomorphous replacement. The solvate model was changed as required.

The crystal under investigation was found to be non-merohedrally twinned. The orientation matrices for the two components were identified using the program Cell_Now, with the two components being related by a 180° rotation around the reciprocal axis (0 1 −1). The two components were integrated using SAINT and corrected for absorption using TWINABS, resulting in the following statistics:

100596 data (22085 unique) involve domain 1 only, mean I/ σ 14.8
100545 data (22019 unique) involve domain 2 only, mean I/ σ 13.3
141284 data (33992 unique) involve 2 domains, mean I/ σ 21.1

22 data (22 unique) involve 3 domains, mean I/ σ 18.7

The exact twin matrix identified by the integration program was found to be:

−0.99986	0.00058	−0.00088
0.42564	0.24419	−0.75620
−0.42452	−1.24286	−0.24434

The structure was solved using direct methods with only the non-overlapping reflections of component 1. The structure was refined using the HKLF 5 routine with all reflections of component 1 (including the overlapping ones), resulting in a BASF value of 0.4569(4).
The R_{int} value given is for all reflections and is based on agreement between observed single and composite intensities and those calculated from refined unique intensities and twin fractions (TWINABS (Sheldrick, 2012)).
Hydroxyl H atom positions were refined and O−H distances were restrained to 0.84(2) Å. Several hydroxyl H atom positons were further restrained based on hydrogen bonding considerations.

Three pyrazole ligands were refined as disordered. For each the two disordered moieties were restrained to have similar geometries. Each moiety was restrained to be coplanar with the copper ions it is bonded to. U_{ij} components of ADPs for disordered atoms closer to each other than 2.0 Å were restrained to be similar. Subject to these conditions the occupancy ratio refined to 0.703(17)/0.297(17)(pyrazole of N17), 0.44(2)/0.56(2) (pyrazole of N23) and 0.54(2)/0.46(2) (pyrazole of N39).

Of the two Bu_4N^+ cations one exhibits minor disorder of one of its butyl chains. The other exhibits whole cation disorder. The two moieties of the single disordered chain were restrained to have a similar geometry as another non-disordered butyl chain. The two moieties of the disordered cation were restrained to have a similar geometry as the other less disordered cation. U_{ij} components of ADPs for disordered atoms closer to each other than 2.0 Å were restrained to be similar. Subject to these conditions the occupancy ratio refined to 0.606(17)/0.394(17) for the disordered butyl chain, and to 0.595(7)/0.405(7) for the whole cation disorder.

Chlorobenzene and pentane molecules were refined disordered or as partially occupied. All chlorobenzene molecules were restrained to have similar geometries and all benzene rings

were constrained to resemble ideal hexagons with C–C bond distances of 1.39 Å. No attempts were made to ensure full occupancy for all solvate sites. Minor ill-defined residual electron density around some pentane and chlorobenzene molecules was ignored. Subject to these conditions the occupancy ratio refined to the values given in the _atom_site_ in the CIF file.

5c: Hydroxyl H atom positions were refined and O–H distances were restrained to 0.84(2) Å. Some hydroxyl H atom positions were further restrained based on hydrogen bonding considerations.

Two butyl chains of one Bu_4N^+ cation were refined as disordered. The disordered moieties were restrained to have similar geometries as another non-disordered butyl chain. U_{ij} components of ADPs for disordered atoms closer to each other than 2.0 Å were restrained to be similar. A general anti-bumping restraint was applied to avoid close contact with disordered solvate molecules (see below). Subject to these conditions the occupancy ratio refined to 0.728(16)/0.272(16) for the butyl group involving C94, and to 0.715(17)/0.285(17) for the butyl group involving C98.

Several solvate molecules were refined as disordered between nitrobenzene and bromobenzene. One site was refined as disordered bromobenzene. All nitrobenzene moieties (including two non-disordered solvate molecules) were restrained to have similar geometries. All bromobenzene moieties were restrained to have similar geometries. Benzene rings of bromobenzene molecules were constrained to resemble ideal hexagons with C–C bond distances of 1.39 Å (AFIX 66 command). The bromine atoms were restrained to be coplanar with the benzene rings. For the disordered bromobenzene molecule, three orientations were refined. Two of them share their benzene rings (i.e. only the position of the bromine atom differs). Minor occupied bromobenzene moieties were restrained to be coplanar with the nitrobenzene moiety of the same site to avoid drifting of the minor moiety (moieties of Br4 and Br5). ADPs for disordered atoms closer to each other than 2.0 Å were restrained to be similar. Subject to these conditions the occupancy ratio refined to 0.821(5)/0.179(5) (nitrobenzene of N67, bromobenzene of Br2), to 0.579(12)/0.174(7) (nitrobenzene of N68, bromobenzene of Br3, site not fully occupied), to 0.929(5)/0.0719(5) (nitrobenzene of N69, bromobenzene of Br4), to 0.934(8)/0.066(8) (nitrobenzene of N70, bromobenzene of Br5). The total occupancy

of the bromobenzene site refined to less than unity. Occupancy rates refined to 0.340(5) (Br1), 0.248(5) (Br6) and 0.167(5) (Br7, shares benzene ring with Br1).

The structure contains another solvate filled pocket of 480 $Å^3$. The residual electron density peaks are not arranged in an interpretable pattern (they did not match nitrobenzene or bromobenzene or hexane, the solvents used in crystallization). The structure factors were instead augmented via reverse Fourier transform methods using the SQUEEZE routine (P. van der Sluis & A.L. Spek (1990). Acta Cryst. A46, 194-201) as implemented in the program Platon. The resultant FAB file containing the structure factor contribution from the electron content of the void space was used together with the original HKL file in further refinement. The FAB file with details of the SQUEEZE results is appended to the CIF file. The SQUEEZE procedure corrected for 206 electrons within the solvent accessible voids, or e.g. ca. 2.4 hexane molecules.

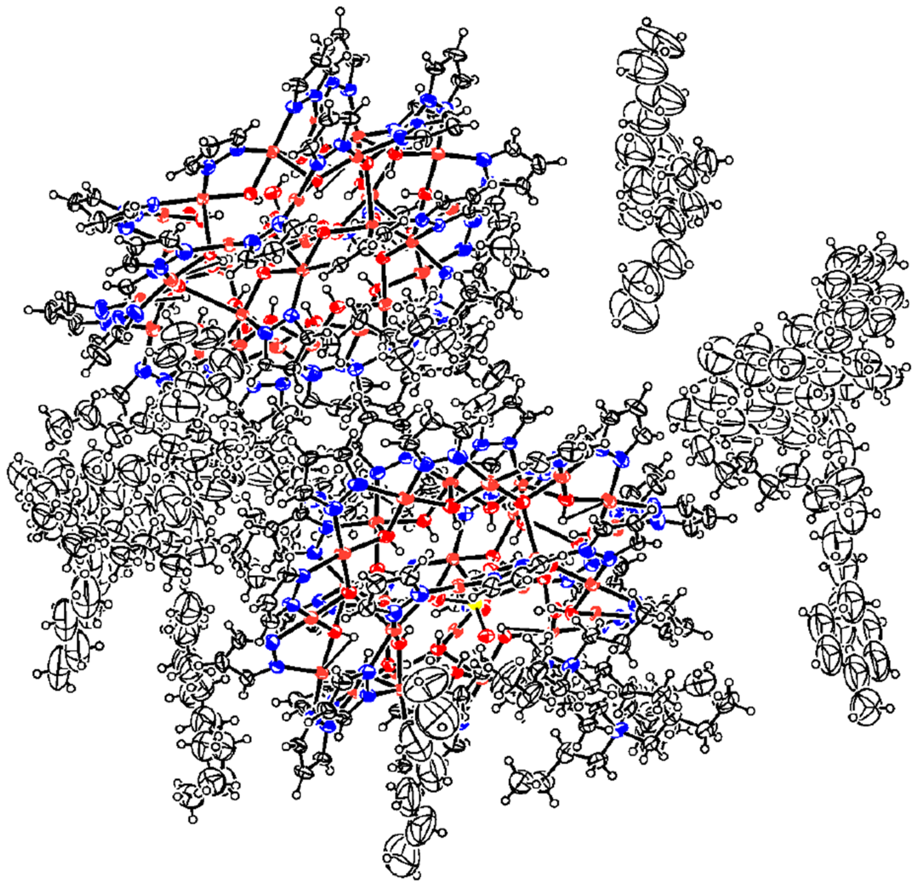

Figure S16. Thermal ellipsoid plot of the crystal structure of **1**.

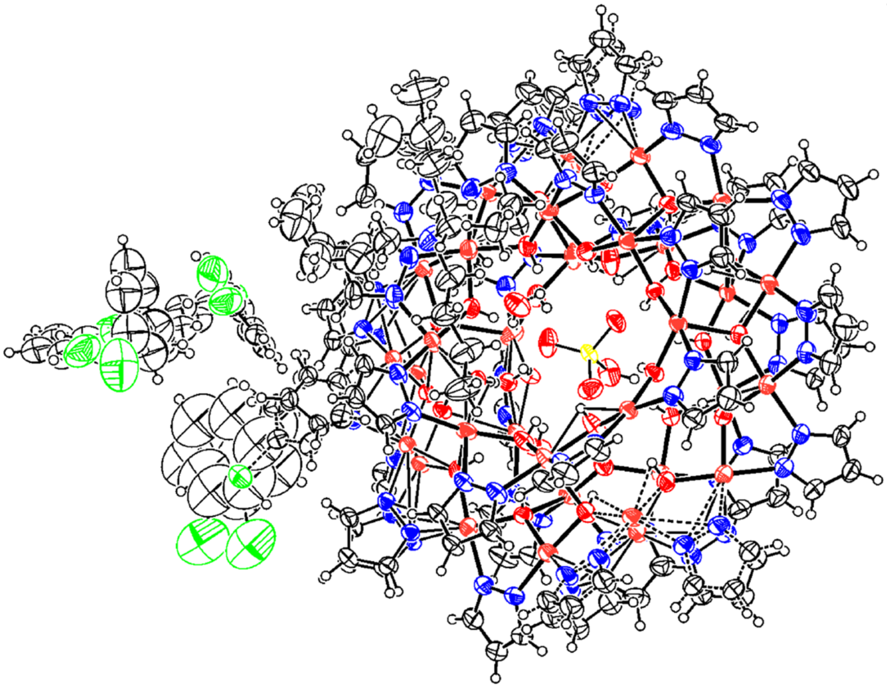

Figure S17. Thermal ellipsoid plot of the crystal structure of **2**.

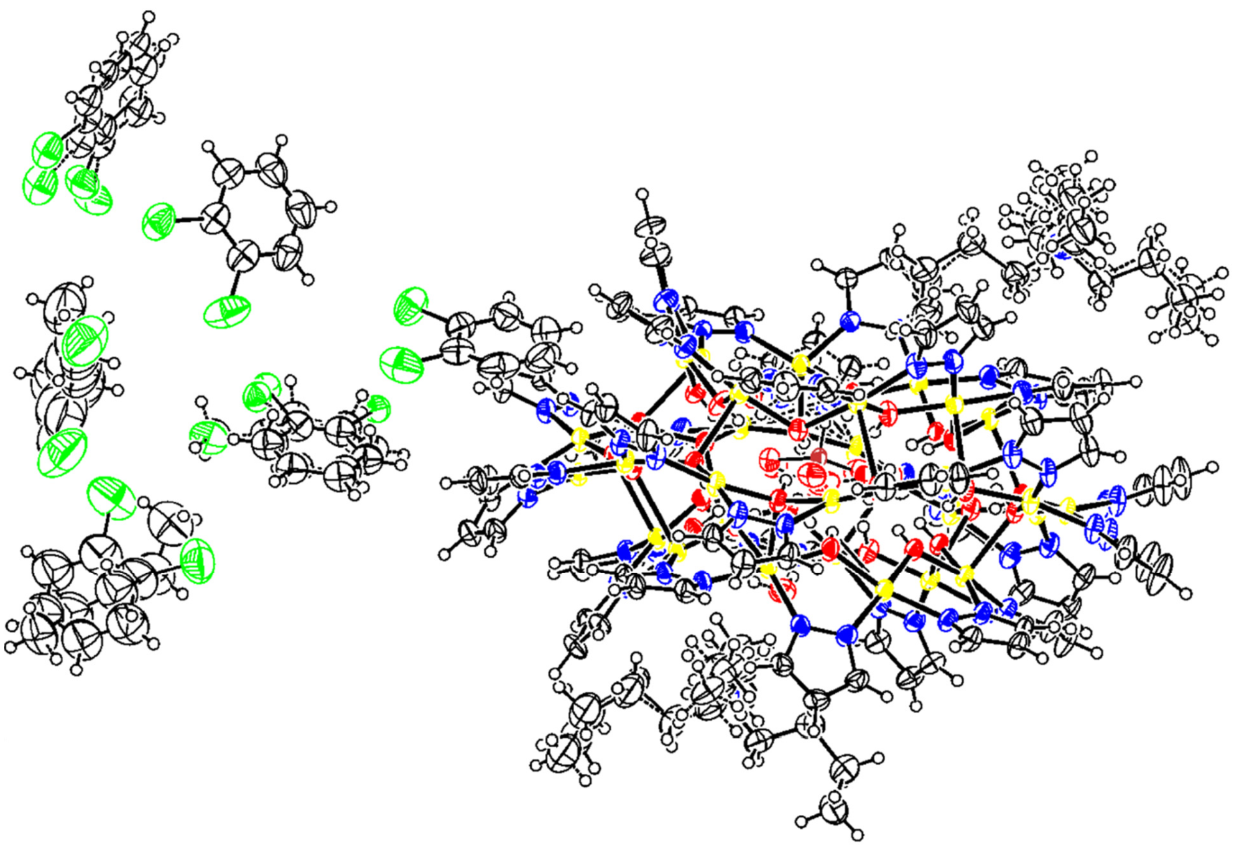

Figure S18. Thermal ellipsoid plot of the crystal structure of **3**.

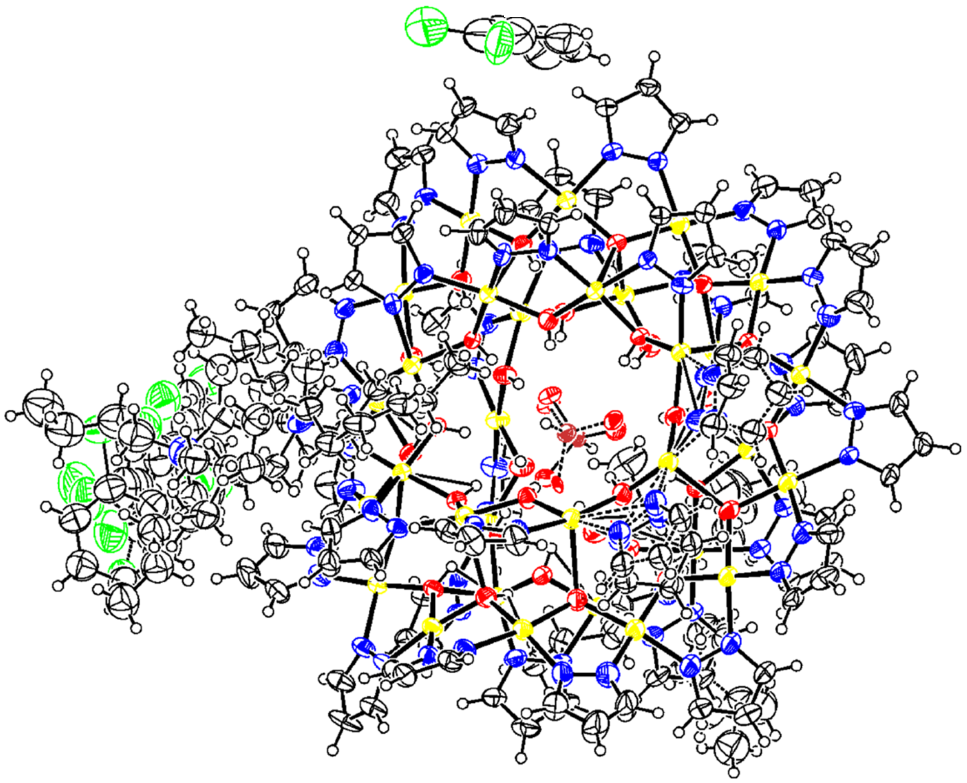

Figure S19. Thermal ellipsoid plot of the crystal structure of **4a**.

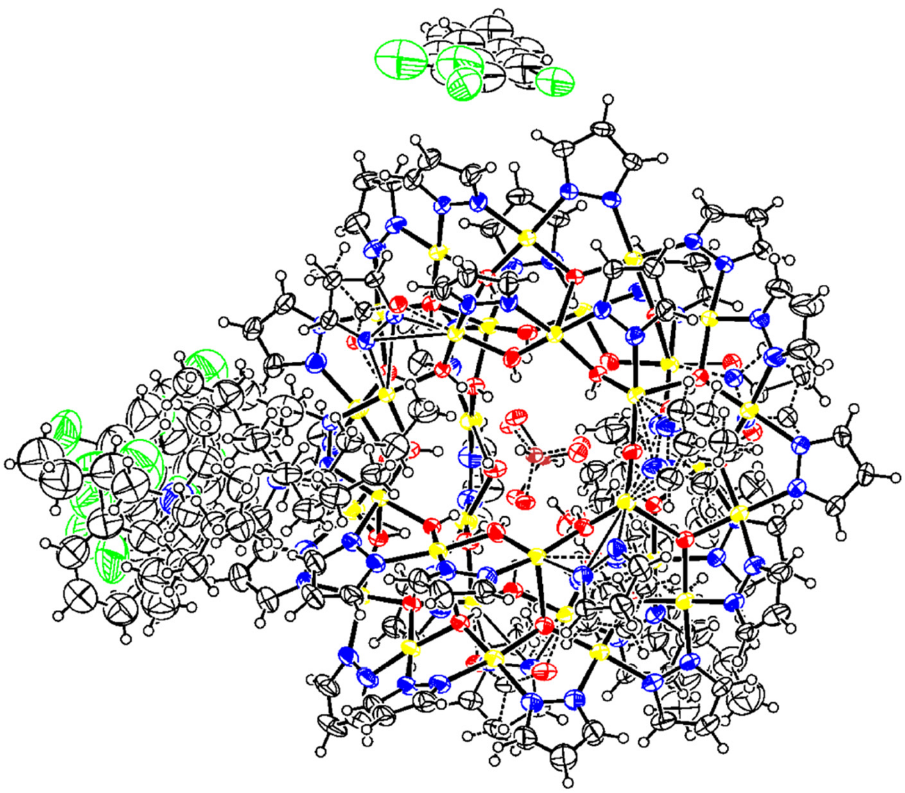

Figure S20. Thermal ellipsoid plot of the crystal structure of **4b**.

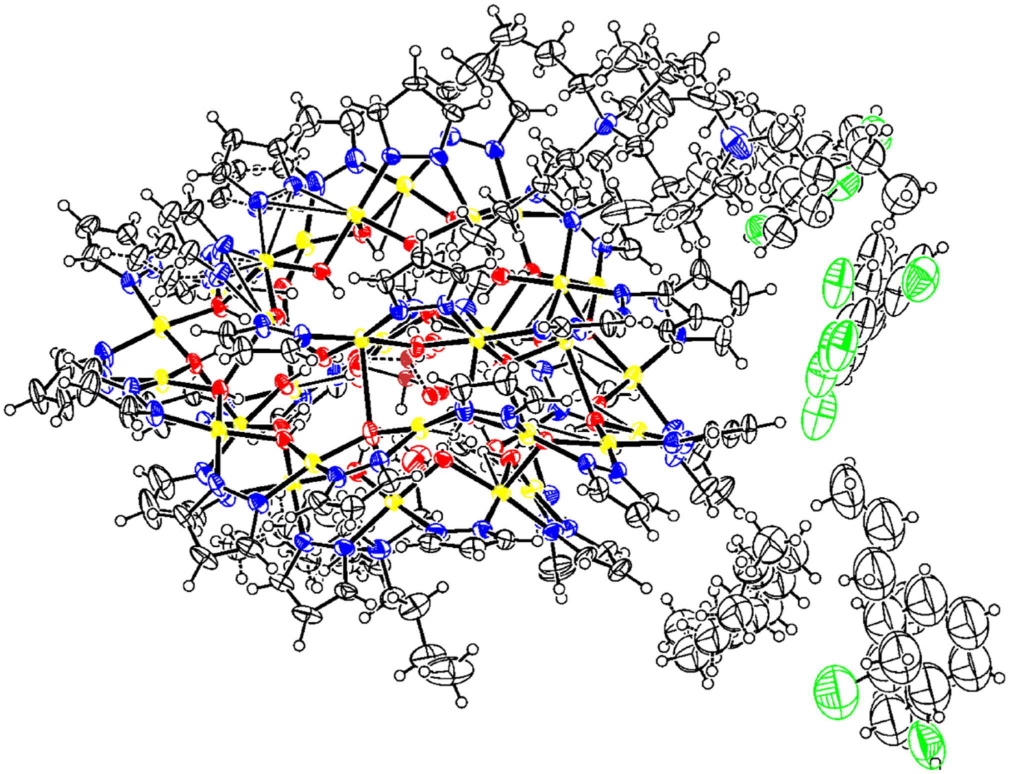

Figure S21. Thermal ellipsoid plot of the crystal structure of **4c**.

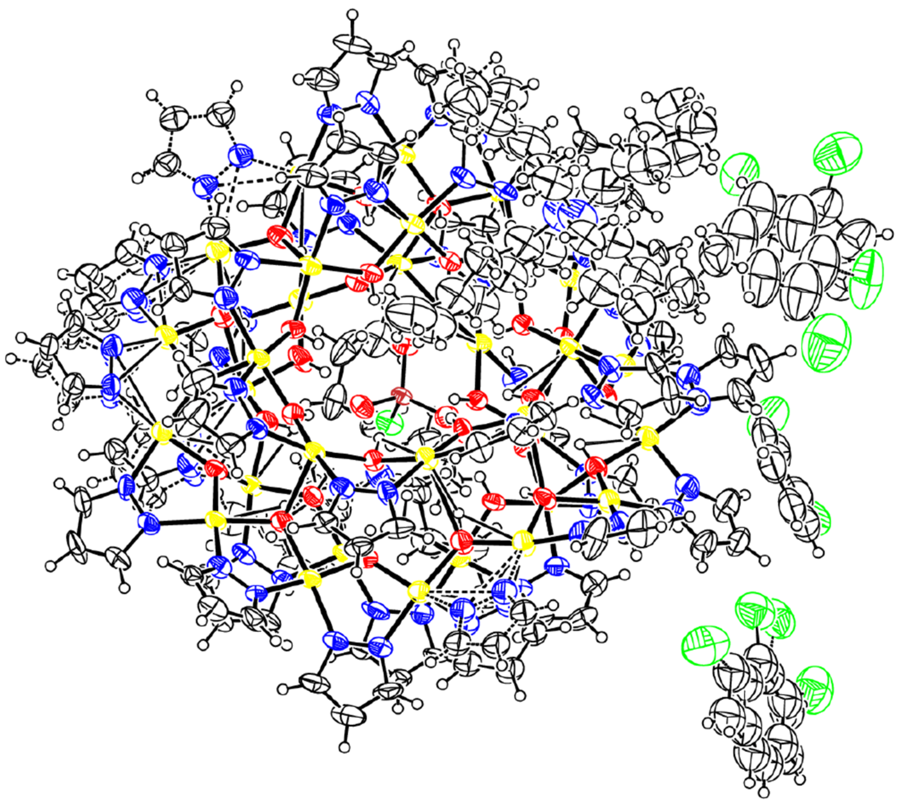

Figure S22. Thermal ellipsoid plot of the crystal structure of **5a**.

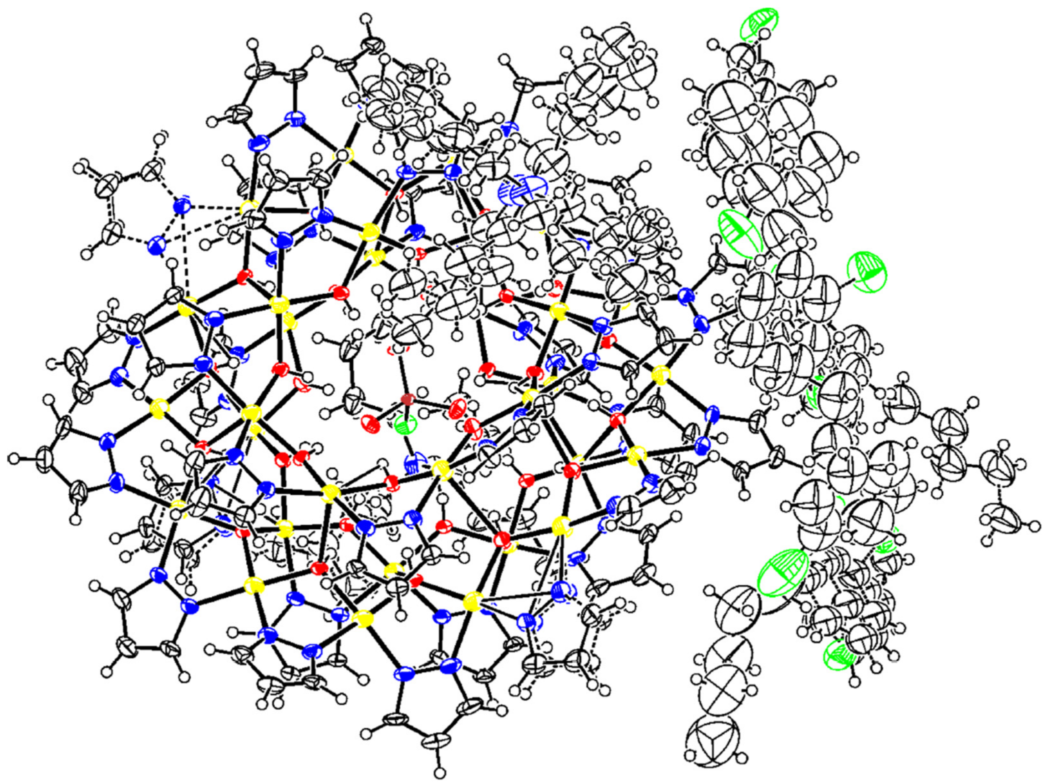

Figure S23. Thermal ellipsoid plot of the crystal structure of **5b**.

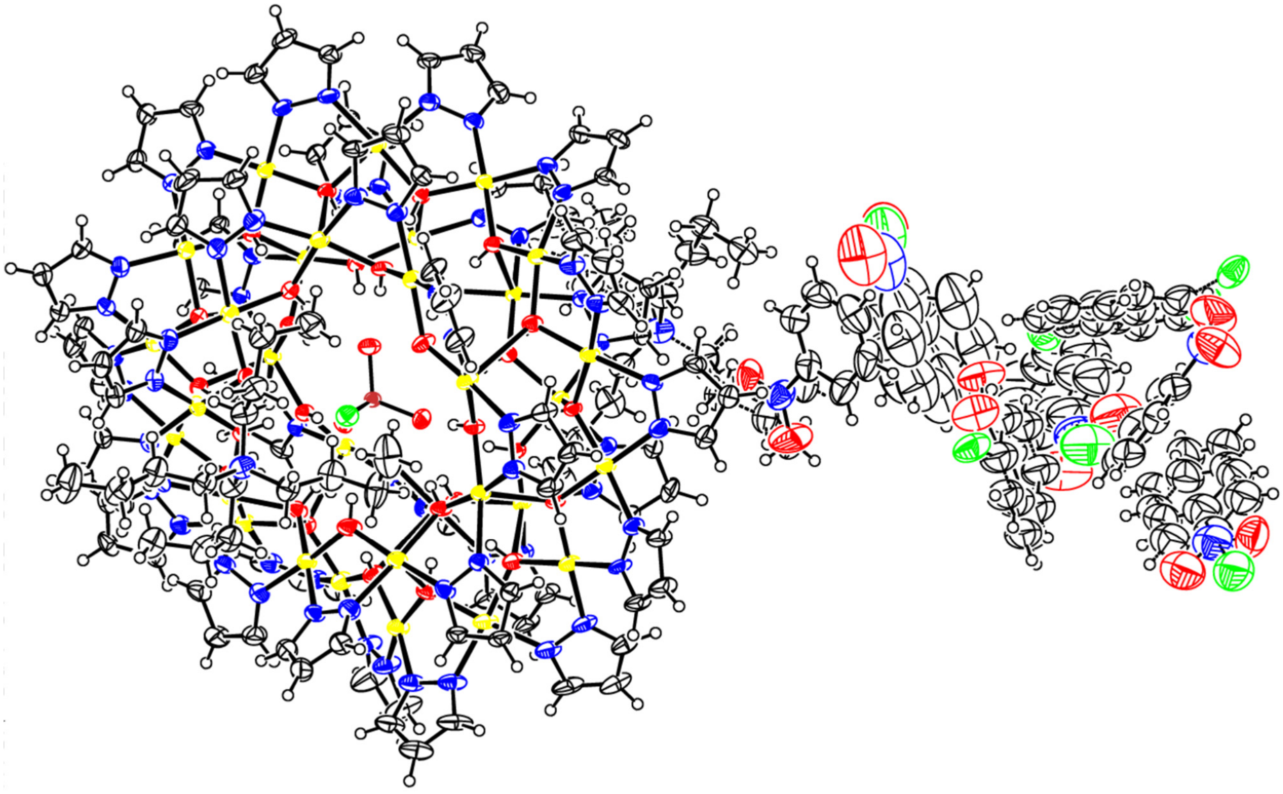

Figure S24. Thermal ellipsoid plot of the crystal structure of **5c**.

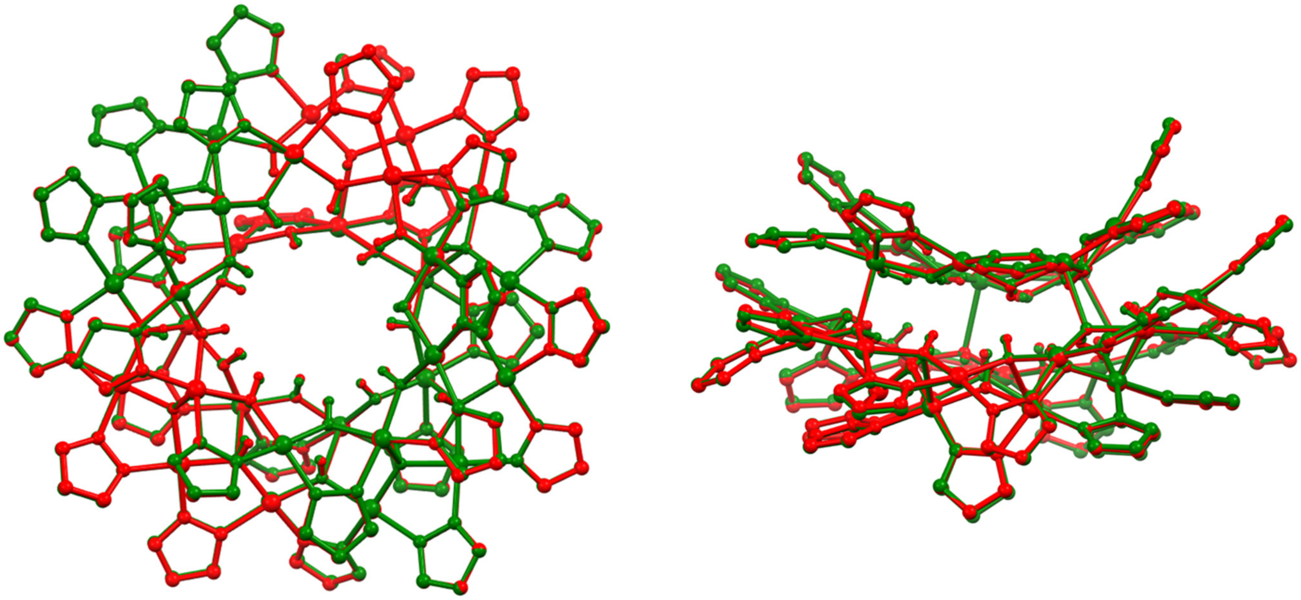

Figure S25. Overlay (top- and side-views) of the two crystallographically independent, pseuodoenantiomeric units of **1** (green and red; one inverted). C–H bond hydrogen atoms, counterions and solvent molecules are omitted for clarity.

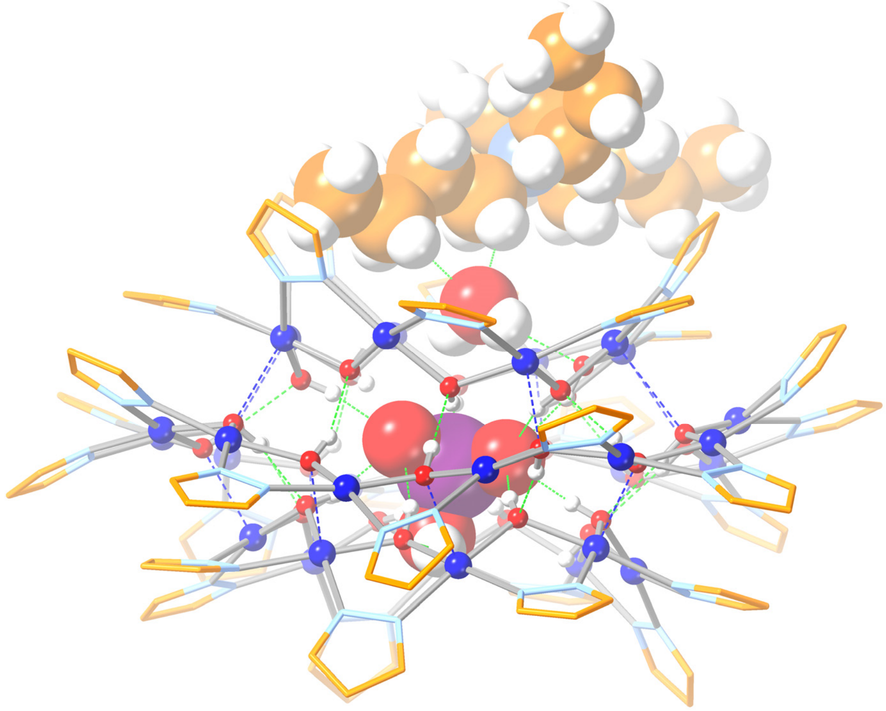

Figure S26. Illustration of the partially occupied H_2O molecule located between a Cu_8 ring and an adjacent Bu_4N^+ counterion within the crystal structure of **2**. Green and blue dotted lines indicate hydrogen bonds and axial Cu···O interactions, respectively. C–H bond H atoms are omitted for clarity and only the major component is shown for disordered moieties.

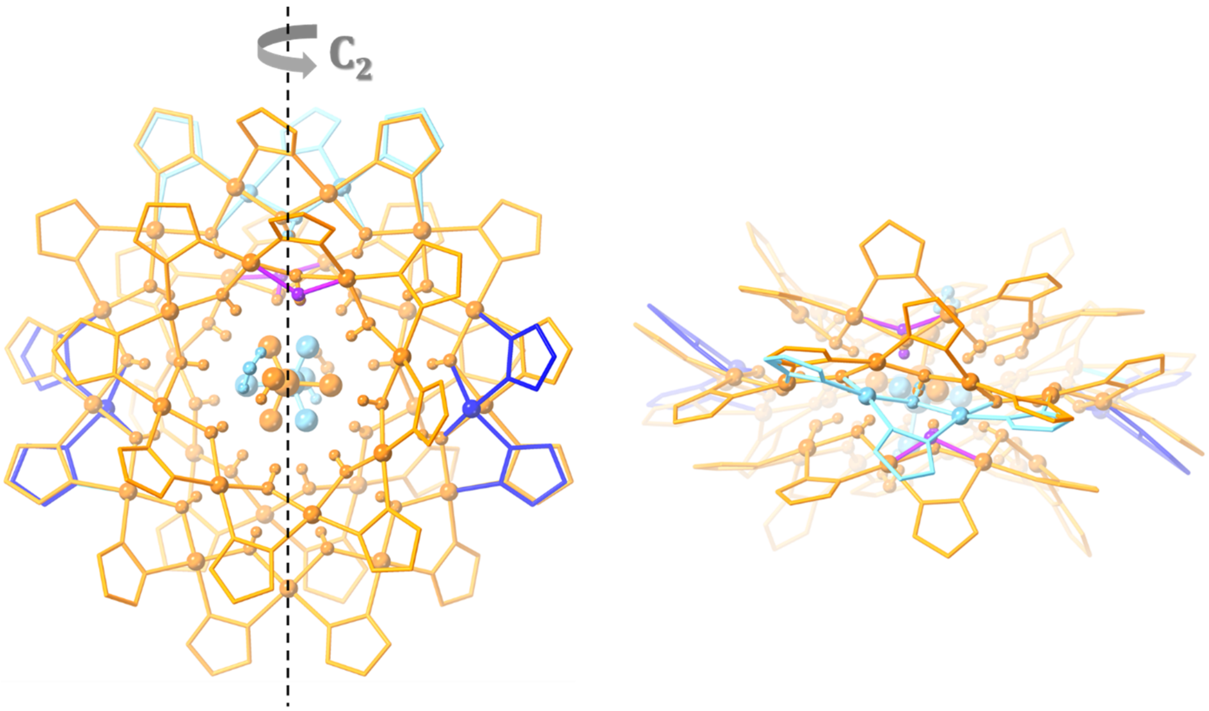

Figure S27. Illustration (top- and side-views) of the different types of disorder in **2**. Light blue: 50% (symmetry-generated); purple: 50%; dark blue: 10%. C–H bond hydrogen atoms, counterions and solvent molecules are omitted for clarity.

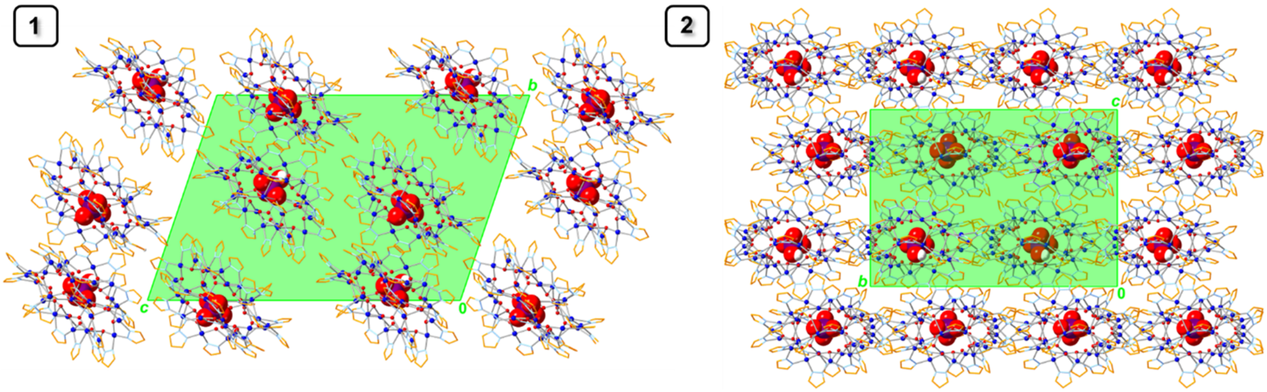

Figure S28. Comparison of the packing diagrams (along the *a* axis) of **1** and **2**. C–H and O–H bond H atoms, counterions and solvent molecules are omitted for clarity, and only the major component is shown for disordered moieties.

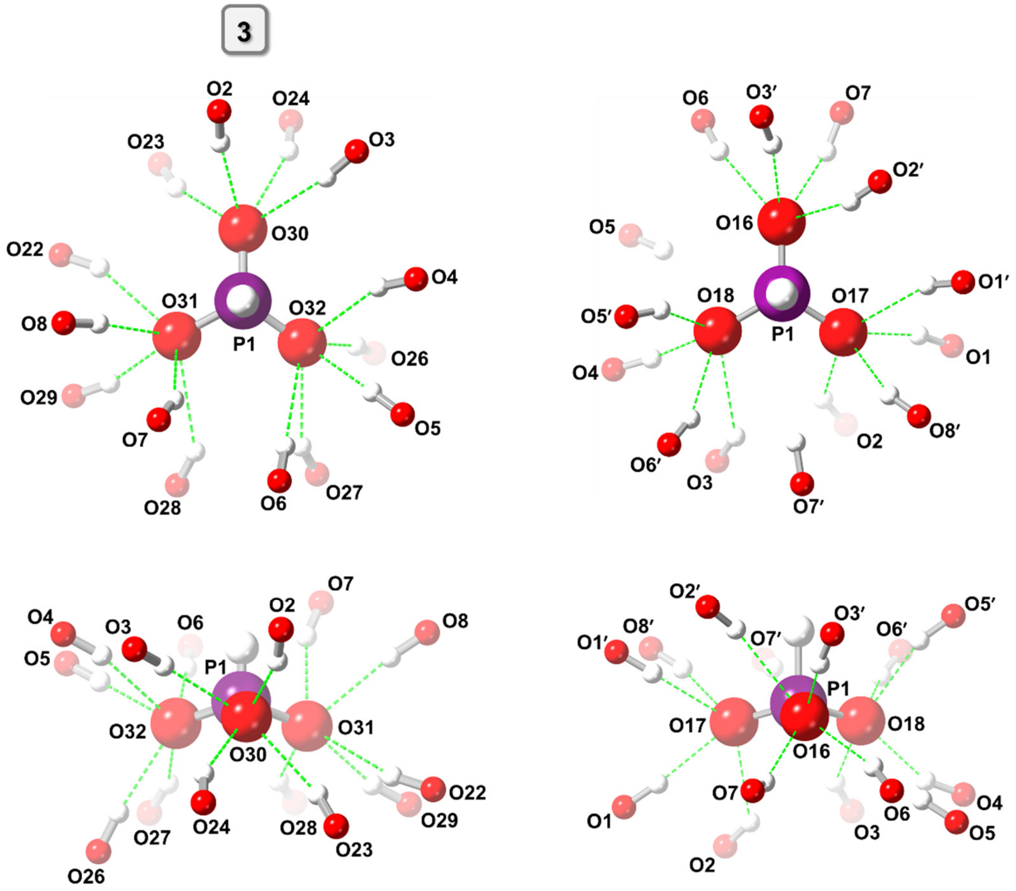

Figure S29. Comparison of the H-bonding patterns (top- and side-views) of **$Cu_{29}HPO_3$** in **3** ($P2_1/c$) with the one of the higher symmetry ($C2/c$) analog.[1] The positions of the H atoms are approximate, and only the major component is shown for the disordered HPO_3^{2-} ions.

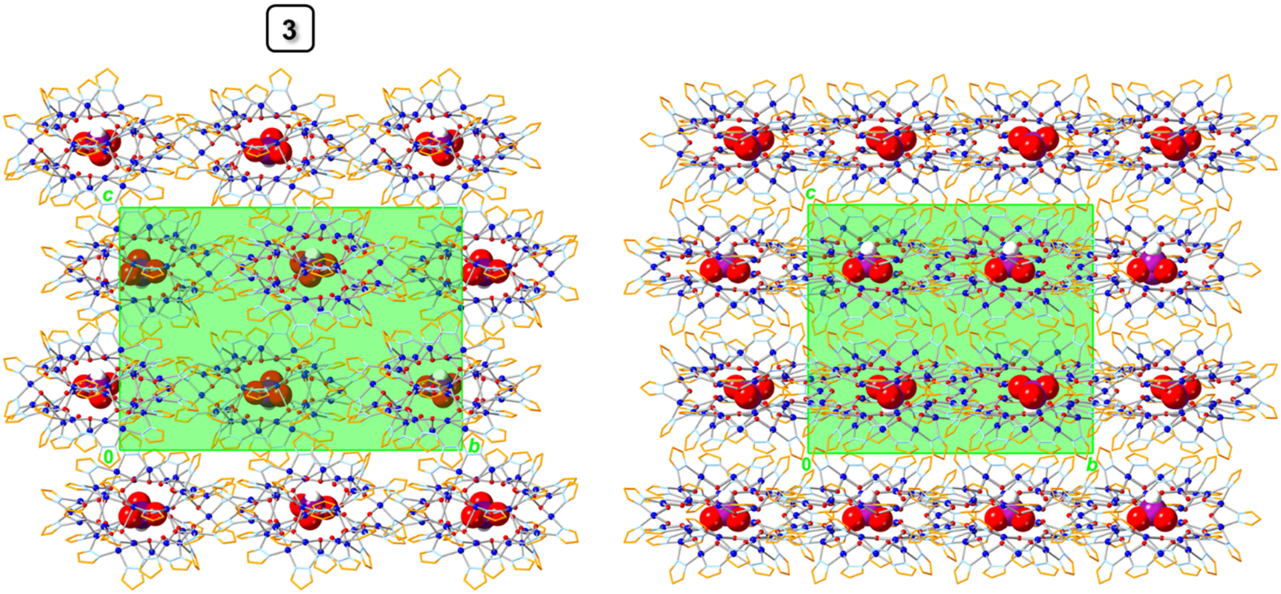

Figure S30. Comparison of the packing diagrams (along the *a* axis) of **3** (left; $P2_1/c$) and the higher symmetry analog (right; $C2/c$). C–H and O–H bond H atoms, counterions and solvent molecules are omitted for clarity, and only the major component is shown for disordered moieties.

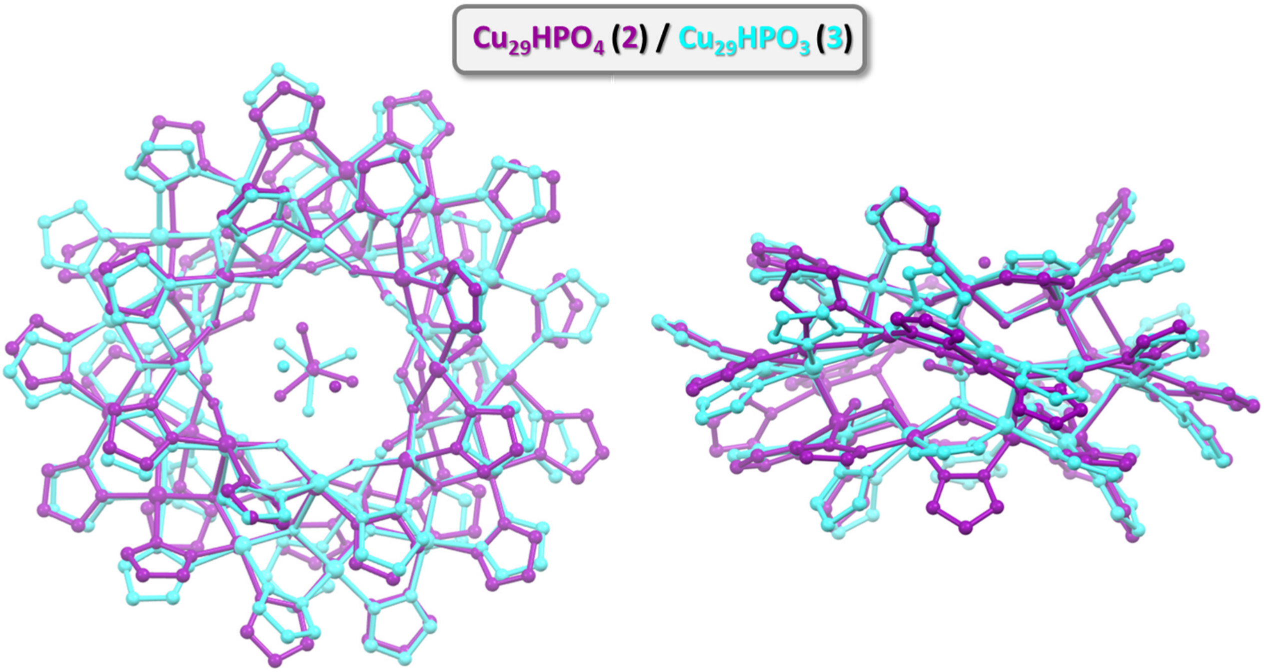

Figure S31. Overlays (top- and side-views) of the structures of **2** (violet) and **3** (light blue). Hydrogen atoms, counterions and solvent molecules are omitted for clarity, and only the major component is shown for disordered moieties. The isolated atoms next to the central anions represent the O atoms of H_2O molecules.

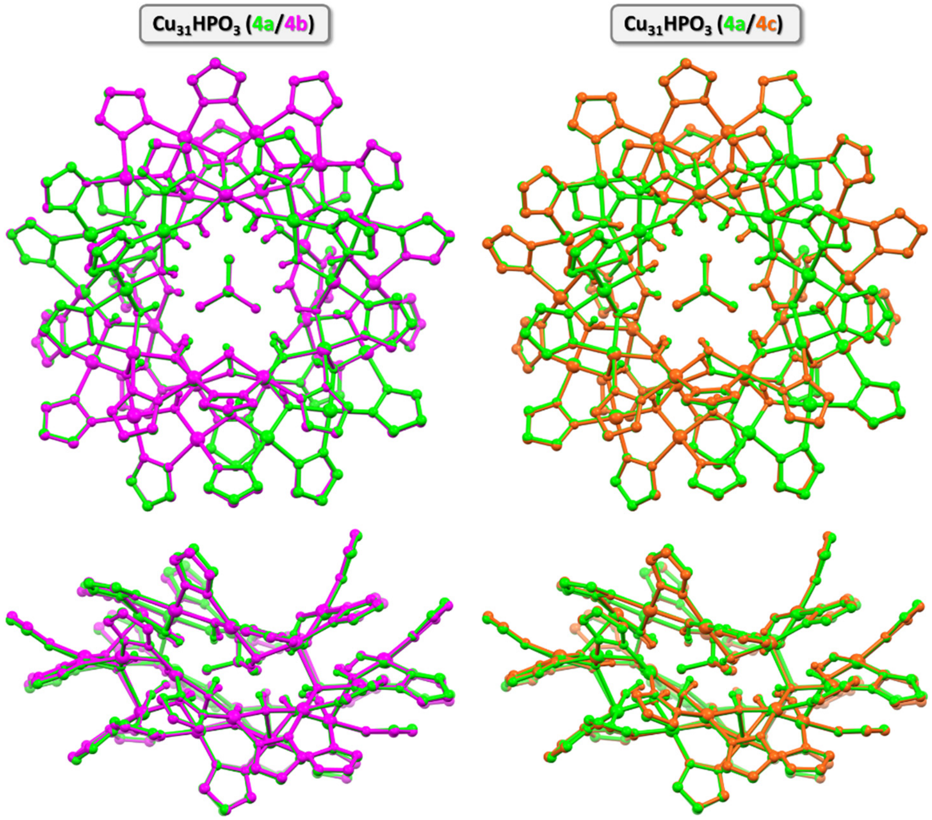

Figure S32. Overlays (top- and side-views) of the structures of **4a** (green), **4b** (magenta) and **4c** (persimmon). C–H bond hydrogen atoms, counterions and solvent molecules are omitted for clarity, and only the major component is shown for disordered moieties.

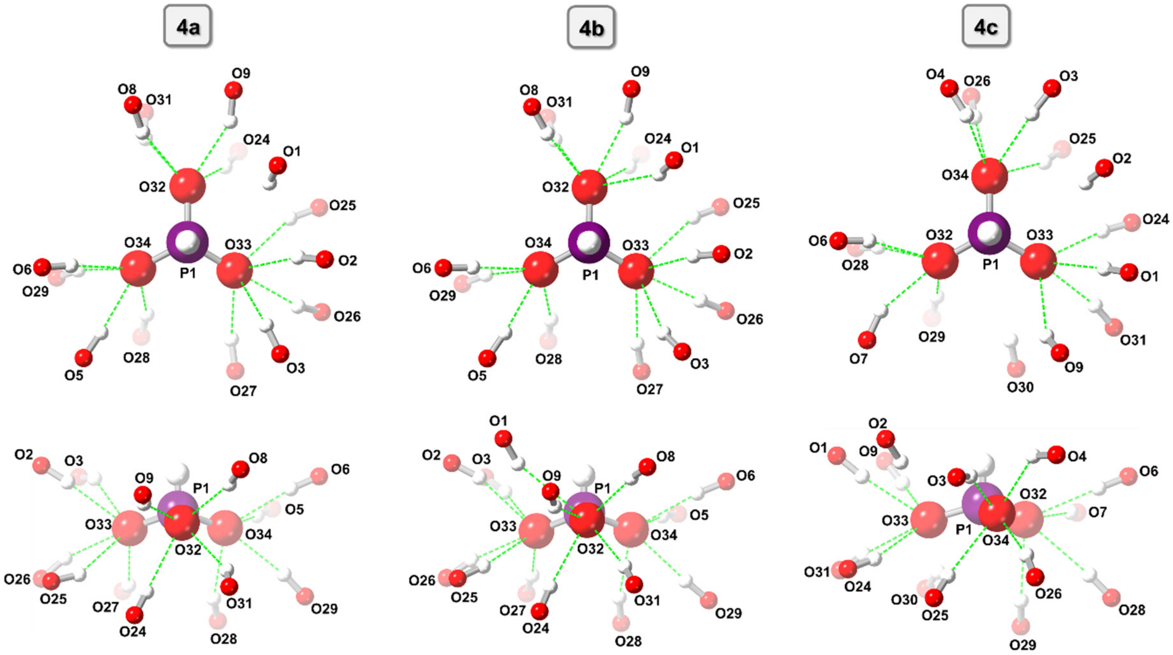

Figure S33. Comparison of the H-bonding patterns (top- and side-views) in the three different pseudopolymorphs of **$Cu_{31}HPO_3$** (**4a–4c**). The positions of the H atoms are approximate, and only the major component is shown for the disordered HPO_3^{2-} ions.

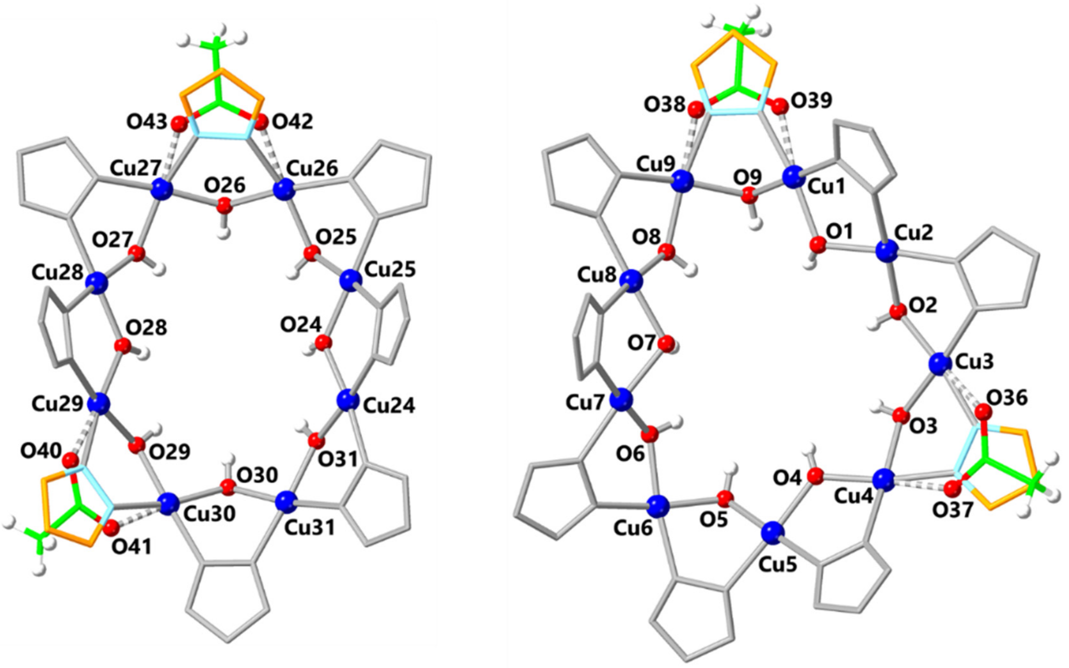

Figure S34. Illustration of the pyrazolate/acetate disorder in the Cu_8 (left) and Cu_9 (right) rings of **$Cu_{31}HPO_3$** (**4b**).

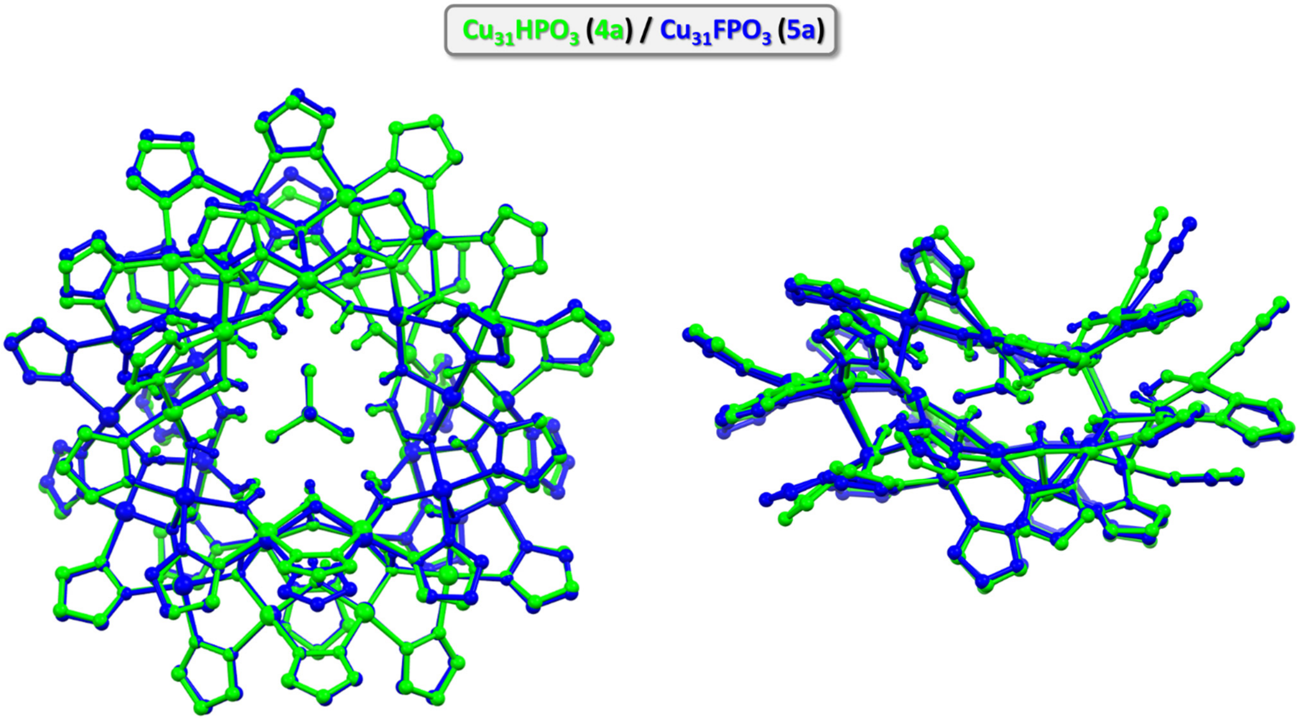

Figure S35. Overlay (top- and side-views) of the structures of **4a** (green) and **5a** (blue). C–H bond hydrogen atoms, counterions and solvent molecules are omitted for clarity, and only the major component is shown for disordered moieties.

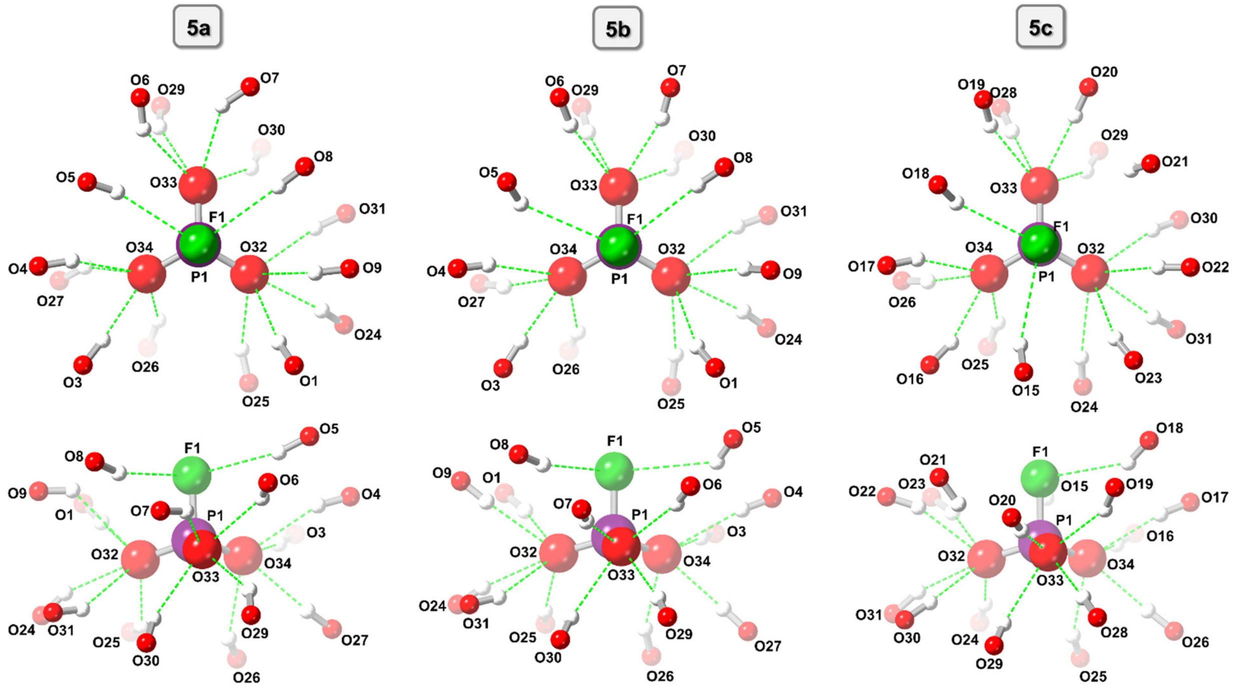

Figure S36. Comparison of the H-bonding patterns (top- and side-views) in the three different pseudopolymorphs of $\mathbf{Cu_{31}FPO_3}$ (**5a**–**5c**). The positions of the H atoms are approximate.

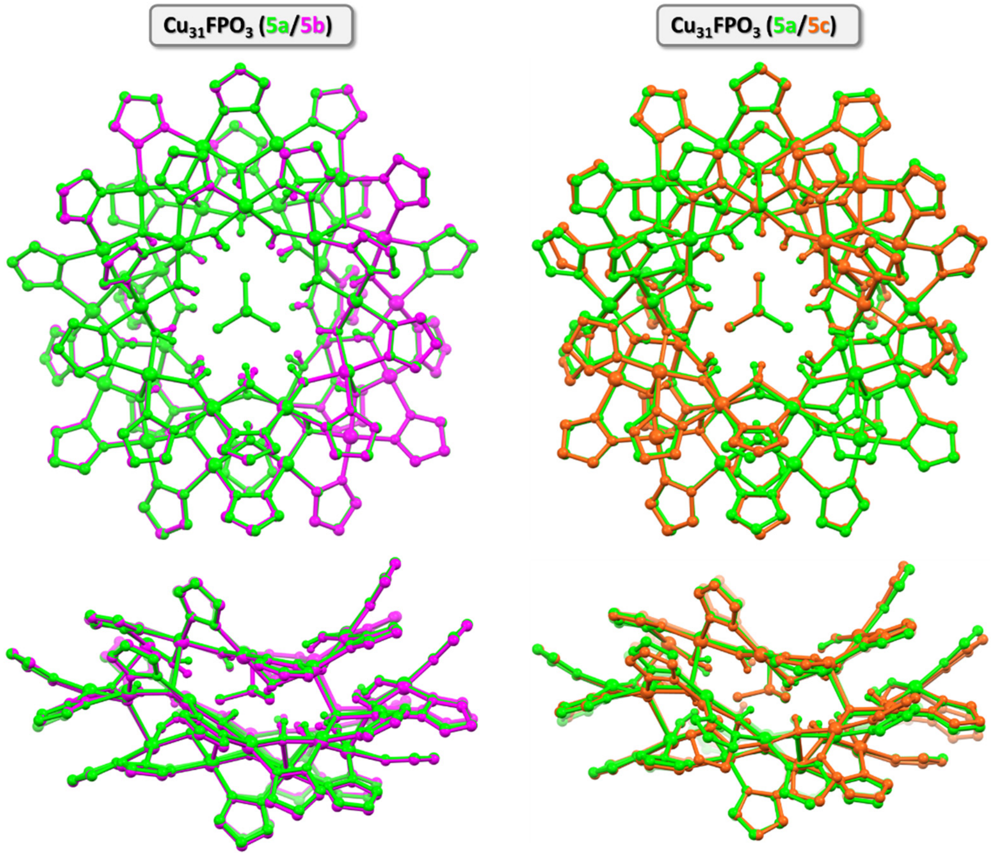

Figure S37. Overlays (top- and side-views) of the structures of **5a** (green), **5b** (magenta) and **5c** (persimmon). C–H bond hydrogen atoms, counterions and solvent molecules are omitted for clarity.

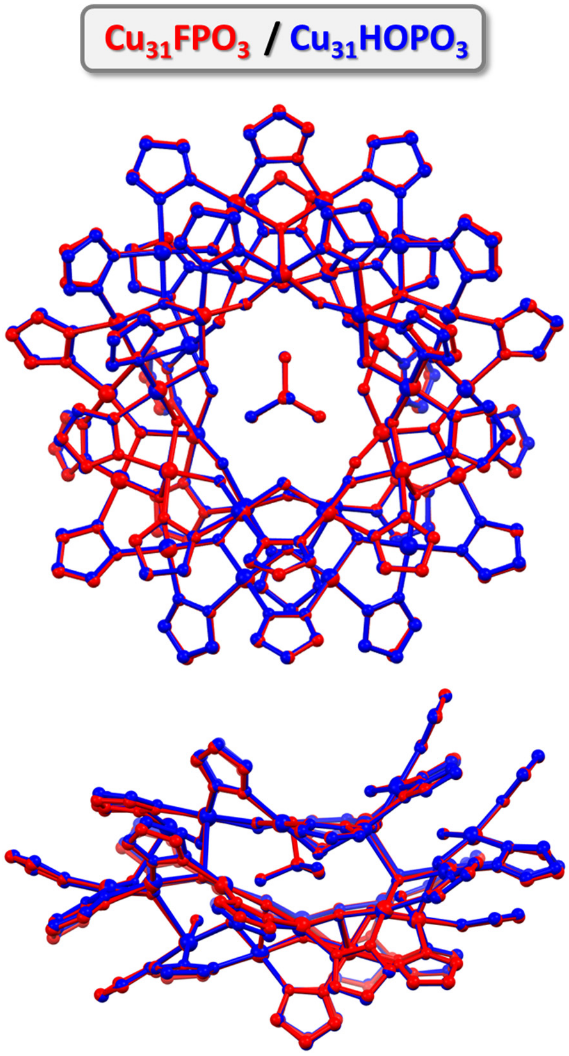

Figure S38. Overlay (top- and side-views) of the structures of **$Cu_{31}FPO_3$** (**5a**; red) and **$Cu_{31}HOPO_3$** (blue). H atoms, counterions and solvent molecules are omitted for clarity, and only the major component is shown for disordered moieties.

Table S1. Crystallographic data for the HPO_4^{2-} (**1** and **2**) and HPO_3^{2-} (**3**) nanojars.

	**1**	**2**	**3**
Formula (sum)	$C_{167.67}H_{291.86}Cu_{29}N_{60}O_{33}P$	$C_{302.25}H_{433.54}Cl_{10.71}Cu_{58}N_{120}O_{67}P_2$	$C_{153.68}H_{224.16}Cl_{9.36}Cu_{29}N_{60}O_{32.31}P$
Formula (moiety)	$C_{87}H_{116}Cu_{29}N_{58}O_{29}$, HPO_4, 2($C_{16}H_{36}N$), 5.905(C_7H_{16}), 1.049(C_7H_8)	2($C_{87}H_{116}Cu_{29}N_{58}O_{29}$), 2($HPO_4$), 4($C_{16}H_{36}N$), 10.708($C_6H_5Cl$), H_2O	$C_{87}H_{116}Cu_{29}N_{58}O_{29}$, HPO_3, 2($C_{16}H_{36}N$), 4.68($C_6H_4Cl_2$), 1.32(C_5H_{12})
FW (g·mol^{-1})	5537.51	5768.88	5635.05
Crystal system	Triclinic	Monoclinic	Monoclinic
Space group	$P\bar{1}$ (No. 2)	C2/c (No. 15)	$P2_1/c$ (No. 14)
a (Å)	22.0805(10)	25.3246(8)	25.8079(9)
b (Å)	28.8451(13)	33.8836(10)	34.0001(11)
c (Å)	39.5089(16)	26.5825(12)	26.4545(9)
α (deg)	105.898(2)	90.000	90.000
β (deg)	94.776(2)	113.9690(10)	114.5057(15)
γ (deg)	109.175(2)	90.000	90.000
V (Å^3)	22439.1(17)	20843.1(13)	21122.0(13)
Z	4	2	4
D_{calc} (g·cm^{-3})	1.643	1.744	1.772
μ (mm^{-1})	3.533	4.418	4.831
θ range (deg)	1.711–80.681	2.608–80.670	2.249–66.805
Reflns collected	357524	335464	183258
R_{int}	0.0473	0.0400	0.0666
Obsd reflns [$I > 2\sigma(I)$]	81698	21896	30189
Data/restraints/parameters	93817/5448/6240	22773/3421/2074	37204/2920/3278
GOF (on F^2)	1.054	1.042	1.092
R factors [$I > 2\ \sigma(I)$]	R_1 = 0.0543<br>wR_2 = 0.1380	R_1 = 0.0428<br>wR_2 = 0.1170	R_1 = 0.0499<br>wR_2 = 0.1343
R factors (all data)	R_1 = 0.0611<br>wR_2 = 0.1419	R_1 = 0.0439<br>wR_2 = 0.1179	R_1 = 0.0639<br>wR_2 = 0.1406
Maximum peak/hole (e·Å^{-3})	1.119/−0.869	1.052/−0.669	1.689/−0.812
CCDC number	2514876	2514877	2514878

Table S2. Crystallographic data for the HPO_3^{2-} nanojars (**4a–c**).

	**4a**	**4b**	**4c**
Formula (sum)	$C_{137.70}H_{207.46}Cl_{4.23}Cu_{31}N_{64}O_{35}P$	$C_{136.04}H_{207.38}Cl_{4.19}Cu_{31}N_{60.96}O_{38.04}P$	$C_{146.80}H_{231.90}Cl_{3.49}Cu_{31}N_{64}O_{35}P$
Formula (moiety)	$C_{93}H_{124}Cu_{31}N_{62}O_{31}$, HPO_3, 2($C_{16}H_{36}N$), 2.12($C_6H_4Cl_2$), H_2O, solvent	$C_{91.48}H_{124}Cu_{31}N_{58.96}$ $O_{34.04}$, HPO_3, 2($C_{16}H_{36}N$), 2.09($C_6H_4Cl_2$), H_2O, solvent	$C_{93}H_{124}Cu_{31}N_{62}O_{31}$, HPO_3, 2($C_{16}H_{36}N$), 1.74($C_6H_4Cl_2$), 1.62(C_7H_{16}), H_2O
FW (g·mol^{-1})	5470.51	5454.78	5578.15
Crystal system	Triclinic	Triclinic	Triclinic
Space group	$P\bar{1}$ (No. 2)	$P\bar{1}$ (No. 2)	$P\bar{1}$ (No. 2)
a (Å)	18.718(7)	18.8216(9)	18.784(7)
b (Å)	20.326(8)	20.4459(9)	20.356(5)
c (Å)	29.049(11)	28.7419(13)	28.863(11)
α (deg)	77.633(13)	76.506(2)	75.90(3)
β (deg)	77.218(14)	77.959(2)	77.85(2)
γ (deg)	81.991(18)	81.972(2)	81.81(2)
V (Å^3)	10479(7)	10470.4(8)	10415(6)
Z	2	2	2
D_{calc} (g·cm^{-3})	1.734	1.730	1.779
μ (mm^{-1})	3.206	3.208	3.218
θ range (deg)	1.801–28.425	2.269–30.555	1.811–28.440
Reflns collected	189871	163900	173495
R_{int}	0.0790	0.0470	0.0682
Obsd reflns [$I > 2σ(I)$]	34673	40072	38453
Data/restraints/parameters	51357/2781/3192	59325/3957/3428	51515/2299/3333
GOF (on F^2)	1.000	1.016	1.010
R factors [$I > 2\ σ(I)$]	R_1 = 0.0818<br>wR_2 = 0.2048	R_1 = 0.0489<br>wR_2 = 0.1253	R_1 = 0.0758<br>wR_2 = 0.1870
R factors (all data)	R_1 = 0.1186<br>wR_2 = 0.2323	R_1 = 0.0807<br>wR_2 = 0.1452	R_1 = 0.0972<br>wR_2 = 0.1971
Maximum peak/hole (e·Å^{-3})	1.434/−1.539	1.485/−0.989	1.408/−1.103
CCDC number	2514879	2514880	2514881

Table S3. Crystallographic data for the FPO_3^{2-} nanojars (**5a–c**).

	**5a**	**5b**	**5c**
Formula (sum)	$C_{140.44}H_{206.30}Cl_{5.15}Cu_{31}FN_{64}O_{34}P$	$C_{160.78}H_{248.19}Cl_{3.58}Cu_{31}FN_{64}O_{34}P$	$C_{164.05}H_{228.54}Br_{1.25}Cu_{31}FN_{69.26}O_{44.53}P$
Formula (moiety)	$C_{93}H_{124}Cu_{31}N_{62}O_{31}$, FPO_3, 2($C_{16}H_{36}N$), 2.58($C_6H_4Cl_2$), solvent	$C_{93}H_{124}Cu_{31}N_{62}O_{31}$, FPO_3, 2($C_{16}H_{36}N$), 3.58(C_6H_5Cl), 2.86(C_5H_{12})	$C_{93}H_{124}Cu_{31}N_{62}O_{31}$, FPO_3, 2($C_{16}H_{36}N$), 5.26($C_6H_5NO_2$), 1.25(C_6H_5Br)
FW (g·mol^{-1})	5537.51	5768.88	6002.72
Crystal system	Triclinic	Triclinic	Triclinic
Space group	$P\bar{1}$ (No. 2)	$P\bar{1}$ (No. 2)	$P\bar{1}$ (No. 2)
a (Å)	20.630(4)	20.6299(13)	20.4301(18)
b (Å)	23.319(6)	23.0729(15)	20.5742(18)
c (Å)	25.671(7)	25.7923(17)	28.909(3)
α (deg)	102.956(13)	103.170(3)	91.133(3)
β (deg)	94.933(9)	94.789(2)	107.142(3)
γ (deg)	110.744(8)	111.185(2)	97.135(3)
V (Å^3)	11068(5)	10958.7(12)	11501.4(18)
Z	2	2	2
D_{calc} (g·cm^{-3})	1.662	1.748	1.733
μ (mm^{-1})	3.047	3.063	3.101
θ range (deg)	1.987–28.370	1.077–26.996	1.869–28.402
Reflns collected	318828	226586	148806
R_{int}	0.0869	0.0714	0.0542
Obsd reflns [$I > 2\sigma(I)$]	36760	41535	39672
Data/restraints/parameters	53064/2927/3129	44758/3736/3475	56672/2447/3291
GOF (on F^2)	1.012	1.007	1.012
R factors [$I > 2\sigma(I)$]	R_1 = 0.0768<br>wR_2 = 0.2006	R_1 = 0.0449<br>wR_2 = 0.1255	R_1 = 0.0685<br>wR_2 = 0.1878
R factors (all data)	R_1 = 0.1158<br>wR_2 = 0.2223	R_1 = 0.0482<br>wR_2 = 0.1286	R_1 = 0.0977<br>wR_2 = 0.2041
Maximum peak/hole (e·Å^{-3})	1.064/−1.332	0.919/−1.029	1.315/−1.021
CCDC number	2514882	2514883	2514884

Table S4. Comparison of bond lengths (Å) and angles (°), Cu···O distances shorter than the sum of the van der Waals radii of Cu and O (2.92 Å) and H-bonding (with D···A distances shorter than 3.22 Å) in **1** and **2** (based on the major component in the case of disordered moieties).

	**$Cu_{29}HPO_4$ (1)** (UNIT 1)	**$Cu_{29}HPO_4$ (1)** (UNIT 2)	**$Cu_{29}HPO_4$ (2)**
Cu–O within Cu_n rings	1.887(3)–1.961(3) avg: 1.925(3)	1.880(3)–1.961(3) avg: 1.925(3)	1.877(3)–1.963(2) avg: 1.926(3)
Cu–N within Cu_n rings	1.953(4)–2.024(3) avg: 1.979(3)	1.945(4)–2.032(3) avg: 1.979(3)	1.956(3)–2.010(2) avg: 1.979(3)
N–Cu–O (*trans*) within Cu_{13}	161.93(14)–175.53(15) avg: 170.2(2)	161.69(14)–175.65(14) avg: 170.2(2)	164.8(2)–173.70(15) avg: 170.3(2)
N–Cu–O (*trans*) within Cu_7 (or Cu_8) ring	158.66(13)–177.49(12) avg: 170.1(2)	159.39(13)–177.90(13) avg: 170.3(2)	163.08(11)–176.42(11) avg: 169.9(1)
N–Cu–O (*trans*) within Cu_9 (or Cu_8) ring	163.65(14)–179.31(15) avg: 172.2(2)	165.19(13)–179.51(13) avg: 172.4(2)	163.08(11)–176.42(11) avg: 169.9(1)
Average of all N–Cu–O (trans) angles	*170.8(2)*	*171.0(2)*	*170.0(1)*
N–Cu–O (*cis*) within Cu_{13} ring	83.82(14)–88.48(14) avg: 86.9(2)	84.03(14)–88.90(13) avg: 87.0(2)	83.85(10)–90.68(16) avg: 87.1(1)
N–Cu–O (*cis*) within Cu_7 or (Cu_8) ring	82.84(12)–86.50(13) avg: 84.8(2)	82.83(12)–86.73(13) avg: 84.8(2)	83.63(10)–89.16(16) avg: 85.5(1)
N–Cu–O (*cis*) within Cu_9 or (Cu_8)ring	82.97(12)–87.84(12) avg: 84.9(2)	82.95(12)–88.11(12) avg: 84.8(2)	83.63(10)–89.16(16) avg: 85.5(1)
Average of all N–Cu–O (cis) angles	*85.5(2)*	*85.5(2)*	*86.0(1)*
Cu···O between Cu_7 (or Cu_8) and Cu_{13} rings	2.388(3)–2.856(4) (7 interactions) avg: 2.568(3)	2.389(3)–2.815(3) (7 interactions) avg: 2.560(3)	2.3378(19)–2.774(4) (7 interactions) avg: 2.559(3)
Cu···O between Cu_9 (or Cu_8) and Cu_{13} rings	2.365(3)–2.858(3) (5 interactions) avg: 2.501(3)	2.375(3)–2.835(3) (5 interactions) avg: 2.500(3)	2.3378(19)–2.774(4) (7 interactions) avg: 2.559(3)
Average of all Cu···O interactions between Cu_n rings	*2.535(3) (12 interactions)*	*2.530(3) (12 interactions)*	*2.559(3) (14 interactions)*
Cu···Cu distances in Cu_{13} ring	3.1850(8)–3.4306(11) avg: 3.296(1)	3.1288(19)–3.3846(19) avg: 3.262(2)	3.2051(11)–3.4668(12) avg: 3.327(1)
Cu···Cu distances in Cu_7 (or Cu_8) ring	3.2251(8)–3.4083(10) avg: 3.314(1)	3.0572(18)–3.3700(18) avg: 3.264(2)	3.2844(10)–3.3624(10) avg: 3.328(1)
Cu···Cu distances in Cu_9 (or Cu_8) ring	3.1764(8)–3.3952(11) avg: 3.328(1)	3.2539(19)–3.3688(17) avg: 3.319(2)	3.2844(10)–3.3624(10) avg: 3.328(1)
Average of all Cu···Cu distances in Cu_n rings	*3.313(1)*	*3.282(2)*	*3.328(1)*
H-bonded O···O distances between Cu_7 (or Cu_8) and Cu_{13} rings	2.689(4)–2.923(3) (6 interactions) avg: 2.804(4)	2.685(4)–2.908(3) (6 interactions) avg: 2.801(4)	2.747(3)–3.039(4) (7 interactions) avg: 2.851(3)
H-bonded O···O distances between Cu_9 (or Cu_8) and Cu_{13} rings	2.717(5)–2.952(5) (6 interactions) avg: 2.821(5)	2.733(5)–2.949(5) (6 interactions) avg: 2.821(5)	2.747(3)–3.039(4) (7 interactions) avg: 2.851(3)
Average of all H-bonded O···O distances between Cu_n rings	*2.813(5) (12 interactions)*	*2.811(5) (12 interactions)*	*2.851(3) (14 interactions)*
H-bonded O···O distances between Cu_n rings and HPO_4^{2-}	2.697(4)–3.162(4) (14 interactions) avg: 2.882(4)	2.698(4)–3.211(4) (14 interactions) avg: 2.889(4)	2.589(7)–2.976(6) (13 interactions) avg: 2.822(7)

Table S5. Comparison of bond lengths (Å) and angles (°), Cu···O distances shorter than the sum of the van der Waals radii of Cu and O (2.92 Å) and H-bonding (with D···A distances shorter than 3.2 Å) in **3** and **4a**–**4c** (based on the major component in the case of disordered moieties).

	$Cu_{29}HPO_3$ (3)	$Cu_{31}HPO_3$ (4a)	$Cu_{31}HPO_3$ (4b)	$Cu_{31}HPO_3$ (4c)
Cu–O within Cu_n rings	1.874(3)–1.954(3)<br>avg: 1.922(3)	1.880(6)–1.960(6)<br>avg: 1.924(6)	1.885(3)–1.983(3)<br>avg: 1.925(3)	1.882(5)–1.996(6)<br>avg: 1.924(5)
Cu–N within Cu_n rings	1.949(4)–2.034(10)<br>avg: 1.979(4)	1.866(16)–2.010(7)<br>avg: 1.967(8)	1.915(12)–2.064(18)<br>avg: 1.972(4)	1.924(7)–2.066(17)<br>avg: 1.972(7)
N–Cu–O (*trans*) within Cu_{13} (or Cu_{14}) ring	163.08(15)–176.3(4)<br>avg: 169.9(2)	161.3(3)–175.0(3)<br>avg: 169.5(3)	161.36(15)–174.61(15)<br>avg: 169.3(2)	161.4(3)–175.2(3)<br>avg: 169.4(3)
N–Cu–O (*trans*) within Cu_8 (or Cu_9) ring	155.22(15)–176.41(18)<br>avg: 170.4(2)	168.2(3)–177.2(3)<br>avg: 174.0(3)	167.82(14)–177.90(16)<br>avg: 173.7(2)	167.7(2)–178.1(3)<br>avg: 174.2(3)
N–Cu–O (*trans*) within Cu_8 ring	160.02(15)–176.19(14)<br>avg: 170.9(2)	163.8(6)–177.0(3)<br>avg: 171.9(3)	163.6(4)–177.48(12)<br>avg: 171.5(2)	166.1(3)–177.0(2)<br>avg: 172.6(3)
Average of all N–Cu–O (trans) angles	*170.4(2)*	*171.8(3)*	*171.5(2)*	*172.1(3)*
N–Cu–O (*cis*) within Cu_{13} (or Cu_{14}) ring	83.82(15)–90.5(4)<br>avg: 86.5(2)	85.9(3)–89.3(3)<br>avg: 87.5(3)	86.01(14)–88.63(14)<br>avg: 87.5(2)	86.0(2)–89.4(2)<br>avg: 87.6(3)
N–Cu–O (*cis*) within Cu_8 or (Cu_9) ring	83.51(14)–88.81(15)<br>avg: 85.2(2)	83.2(3)–86.9(3)<br>avg: 85.1(3)	80.6(6)–87.48(13)<br>avg: 84.8(2)	83.7(3)–87.4(3)<br>avg: 85.2(3)
N–Cu–O (*cis*) within Cu_8 ring	83.99(14)–87.98(14)<br>avg: 85.2(2)	82.6(6)–86.4(3)<br>avg: 84.5(3)	82.5(4)–89.9(4)<br>avg: 85.0(2)	83.3(6)–88.2(6)<br>avg: 84.9(3)
Average of all N–Cu–O (cis) angles	*85.6(2)*	*85.7(3)*	*85.8(2)*	*85.9(3)*
Cu···O between Cu_8 (or Cu_9) and Cu_{13} (or Cu_{14}) rings	2.331(3)–2.758(4)<br>(6 interactions)<br>avg: 2.530(4)	2.334(6)–2.879(6)<br>(5 interactions)<br>avg: 2.535(6)	2.339(3)–2.853(3)<br>(5 interactions)<br>avg: 2.514(3)	2.330(5)–2.848(6)<br>(5 interactions)<br>avg: 2.526(6)
Cu···O between Cu_8 and Cu_{13} (or Cu_{14}) rings	2.348(3)–2.717(3)<br>(7 interactions)<br>avg: 2.552(3)	2.418(5)–2.857(5)<br>(8 interactions)<br>avg: 2.536(6)	2.421(3)–2.868(3)<br>(8 interactions)<br>avg: 2.535(3)	2.406(5)–2.857(5)<br>(8 interactions)<br>avg: 2.534(6)
Average of all Cu···O interactions between Cu_n rings	*2.541(4)<br>(13 interactions)*	*2.535(6)<br>(13 interactions)*	*2.524(3)<br>(13 interactions)*	*2.530(6)<br>(13 interactions)*
Cu···Cu distances in Cu_{13} (or Cu_{14}) ring	3.2562(10)–3.4197(10)<br>avg: 3.328(1)	3.1288(19)–3.3846(19)<br>avg: 3.262(2)	3.1346(8)–3.3858(8)<br>avg: 3.264(1)	3.1360(16)–3.3859(15)<br>avg: 3.262(2)
Cu···Cu distances in Cu_8 (or Cu_9) ring	3.2656(10)–3.3751(12)<br>avg: 3.331(1)	3.0572(18)–3.3700(18)<br>avg: 3.264(2)	3.0715(8)–3.4431(8)<br>avg: 3.272(1)	3.0658(15)–3.3843(16)<br>avg: 3.266(2)
Cu···Cu distances in Cu_8 ring	3.2655(10)–3.3715(11)<br>avg: 3.325(1)	3.2539(19)–3.3688(17)<br>avg: 3.319(2)	3.2613(6)–3.3847(5)<br>avg: 3.332(1)	3.2576(15)–3.3692(17)<br>avg: 3.325(2)
Average of all Cu···Cu distances in Cu_n rings	*3.328(1)*	*3.282(2)*	*3.289(1)*	*3.284(2)*
H-bonded O···O distances between Cu_8 (or Cu_9) and Cu_{13} (or Cu_{14}) rings	2.757(5)–3.042(4)<br>(6 interactions)<br>avg: 2.844(5)	2.757(10)–2.881(8)<br>(6 interactions)<br>avg: 2.814(9)	2.753(5)–2.932(4)<br>(6 interactions)<br>avg: 2.816(4)	2.734(8)–2.908(7)<br>(6 interactions)<br>avg: 2.809(8)
H-bonded O···O distances between Cu_8 and Cu_{13} (or Cu_{14}) rings	2.734(4)–2.923(5)<br>(6 interactions)<br>avg: 2.808(5)	2.753(7)–2.894(11)<br>(6 interactions)<br>avg: 2.814(9)	2.748(3)–2.889(5)<br>(6 interactions)<br>avg: 2.819(4)	2.759(6)–2.892(10)<br>(6 interactions)<br>avg: 2.819(8)
Average of all H-bonded O···O distances between Cu_n rings	*2.826(5)<br>(12 interactions)*	*2.814(9)<br>(12 interactions)*	*2.817(4)<br>(12 interactions)*	*2.814(8)<br>(12 interactions)*
H-bonded O···O distances between Cu_n rings and HPO_3^{2-}	2.685(14)–3.175(14)<br>(14 interactions)<br>avg: 2.920(14)	2.820(11)–3.163(14)<br>(13 interactions)<br>avg: 2.925(11)	2.827(8)–3.184(13)<br>(14 interactions)<br>avg: 2.957(10)	2.789(11)–3.038(12)<br>(12 interactions)<br>avg: 2.913(12)

Table S6. Comparison of bond lengths (Å) and angles (°), Cu···O distances shorter than the sum of the van der Waals radii of Cu and O (2.92 Å) and H-bonding (with D···A distances shorter than 3.2 Å) in **5a**–**5c** (based on the major component in the case of disordered moieties).

	**$Cu_{31}FPO_3$ (5a)**	**$Cu_{31}FPO_3$ (5b)**	**$Cu_{31}FPO_3$ (5c)**
Cu–O within Cu_n rings	1.889(8)–1.966(8) avg: 1.925(8)	1.893(3)–1.973(3) avg: 1.924(3)	1.897(5)–1.974(5) avg: 1.926(5)
Cu–N within Cu_n rings	1.936(14)–2.067(16) avg: 1.979(11)	1.953(4)–2.017(4) avg: 1.975(4)	1.944(6)–2.013(6) avg: 1.976(6)
N–Cu–O (*trans*) within Cu_{14} ring	158.0(4)–176.1(4) avg: 169.2(4)	158.3(3)–175.47(16) avg: 169.6(2)	159.7(3)–174.6(3) avg: 169.8(3)
N–Cu–O (*trans*) within Cu_9 ring	169.6(4)–179.6(4) avg: 174.4(4)	169.50(15)–178.23(16) avg: 174.0(2)	169.0(2)–178.1(2) avg: 173.9(2)
N–Cu–O (*trans*) within Cu_8 ring	166.6(4)–178.1(4) avg: 172.2(4)	165.68(16)–177.33(16) avg: 172.4(2)	165.9(2)–177.1(2) avg: 172.9(2)
Average of all N–Cu–O (trans) angles	*171.9(4)*	*172.0(2)*	*172.2(2)*
N–Cu–O (*cis*) within Cu_{14} ring	85.7(4)–89.0(8) avg: 87.7(4)	85.55(17)–88.9(6) avg: 87.8(2)	85.2(2)–89.1(2) avg: 87.7(2)
N–Cu–O (*cis*) within Cu_9 ring	81.3(6)–87.2(4) avg: 84.6(4)	82.5(3)–86.75(17) avg: 84.6(2)	83.6(2)–87.9(2) avg: 84.8(2)
N–Cu–O (*cis*) within Cu_8 ring	82.3(4)–86.4(3) avg: 84.5(4)	82.92(14)–86.55(16) avg: 84.7(2)	83.1(2)–86.4(2) avg: 84.8(2)
Average of all N–Cu–O (cis) angles	*85.6(4)*	*85.7(2)*	*85.8(2)*
Cu···O between Cu_9 and Cu_{14} rings	2.382(9)–2.711(7) (5 interactions) avg: 2.521(8)	2.373(3)–2.695(3) (5 interactions) avg: 2.522(3)	2.342(4)–2.750(5) (5 interactions) avg: 2.509(5)
Cu···O between Cu_8 and Cu_{14} rings	2.419(8)–2.866(7) (8 interactions) avg: 2.534(8)	2.430(3)–2.915(3) (8 interactions) avg: 2.530(3)	2.436(5)–2.698(6) (8 interactions) avg: 2.525(5)
Average of all Cu···O interactions between Cu_n rings	*2.527(8) (13 interactions)*	*2.526(3) (13 interactions)*	*2.517(5) (13 interactions)*
Cu···Cu distances in Cu_{14} ring	3.143(2)–3.372(3) avg: 3.261(3)	3.1549(10)–3.3728(14) avg: 3.259(1)	3.1392(17)–3.3625(13) avg: 3.264(2)
Cu···Cu distances in Cu_9 ring	3.122(3)–3.378(2) avg: 3.287(3)	3.1191(11)–3.3724(11) avg: 3.285(1)	3.1456(13)–3.3797(14) avg: 3.281(2)
Cu···Cu distances in Cu_8 ring	3.287(2)–3.3895(19) avg: 3.343(3)	3.2855(10)–3.3904(9) avg: 3.340(1)	3.2762(13)–3.4011(12) avg: 3.345(2)
Average of all Cu···Cu distances in Cu_n rings	*3.297(3)*	*3.295(1)*	*3.297(2)*
H-bonded O···O distances between Cu_9 and Cu_{14} rings	2.784(14)–2.889(11) (6 interactions) avg: 2.834(12)	2.785(5)–2.904(5) (6 interactions) avg: 2.833(5)	2.788(7)–2.878(8) (6 interactions) avg: 2.833(7)
H-bonded O···O distances between Cu_8 and Cu_{14} rings	2.782(12)–2.885(10) (6 interactions) avg: 2.833(12)	2.781(5)–2.904(4) (6 interactions) avg: 2.837(5)	2.784(6)–2.893(7) (6 interactions) avg: 2.844(7)
Average of all H-bonded O···O distances between Cu_n rings	*2.833(12) (12 interactions)*	*2.835(5) (12 interactions)*	*2.838(7) (12 interactions)*
H-bonded O···O distances between Cu_n rings and FPO_3^{2-}	2.833(10)–3.133(11) (13 interactions) avg: 2.953(11)	2.825(4)–3.126(4) (13 interactions) avg: 2.949(4)	2.819(7)–3.166(7) (13 interactions) avg: 2.946(7)

Table S7. Comparison of the dihedral, twist and fold angles (°) between pyrazolate moieties and adjacent Cu–O–Cu units (as defined in reference 2) in **1** and **2** (based on the major component in the case of disordered moieties).

	DIHEDRAL ANGLE	TWIST ANGLE	FOLD ANGLE
**$Cu_{29}HPO_4$ (1)** Cu_{13}-ring UNIT 1	6.7(3)–53.7(3) avg: 41.3(3)	0.01(17)–12.68(16) avg: 4.4(2)	4.5(3)–53.7(3) avg: 36.7(3)
**$Cu_{29}HPO_4$ (1)** Cu_7-ring UNIT 1	9.81(17)–64.6(2) avg: 47.7(2)	0.38(17)–6.75(18) avg: 3.6(2)	8.87(18)–66.3(2) avg: 47.6(2)
**$Cu_{29}HPO_4$ (1)** Cu_9-ring UNIT 1	6.48(17)–60.8(2) avg: 45.5(2)	0.05(15)–6.30(16) avg: 3.1(2)	1.6(3)–60.7(2) avg: 46.2(3)
Avg. **$Cu_{29}HPO_4$ (1)** UNIT 1	*44.8(3)*	*3.7(2)*	*43.5(3)*
**$Cu_{29}HPO_4$ (1)** Cu_{13}-ring UNIT 2	39.3(2)–72.9(2) avg: 55.1(2)	39.0(3)–73.9(2) avg: 56.1(3)	2.0(2)–53.4(3) avg: 20.0(3)
**$Cu_{29}HPO_4$ (1)** Cu_7-ring UNIT 2	25.3(2)–64.1(2) avg: 42.0(2)	4.7(2)–58.3(2) avg: 31.6(2)	15.6(2)–42.3(5) avg: 27.2(2)
**$Cu_{29}HPO_4$ (1)** Cu_9-ring UNIT 2	6.90(18)–62.7(2) avg: 44.8(2)	0.97(16)–6.31(15) avg: 3.5(2)	2.8(3)–62.7(2) avg: 45.8(3)
Avg. **$Cu_{29}HPO_4$ (1)** UNIT 2	*47.3(2)*	*30.4(3)*	*31.0(3)*
**$Cu_{29}HPO_4$ (2)** Cu_{13}-ring	12.10(17)–48.9(2) avg: 37.6(2)	2.0(4)–15.64(14) avg: 6.0(2)	3.7(3)–48.8(2) avg: 36.9(2)
**$Cu_{29}HPO_4$ (2)** Cu_8-ring	2.8(3)–64.79(19) avg: 42.3(2)	0.08(12)–6.95(11) avg: 1.6(1)	2.7(3)–64.79(19) avg: 42.1(2)
**$Cu_{29}HPO_4$ (2)** Cu_8-ring	2.8(3)–64.79(19) avg: 42.3(2)	0.08(12)–6.95(11) avg: 1.6(1)	2.7(3)–64.79(19) avg: 42.1(2)
Avg. **$Cu_{29}HPO_4$ (2)**	*40.7(2)*	*3.1(2)*	*40.4(2)*

Table S8. Comparison of the dihedral, twist and fold angles (°) between pyrazolate moieties and adjacent Cu–O–Cu units (as defined in reference 2) in **3–5** (based on the major component in the case of disordered moieties).

	DIHEDRAL ANGLE	TWIST ANGLE	FOLD ANGLE
**$Cu_{29}HPO_3$ (3)** Cu_{13}-ring	4.64(17)–51.2(2)<br>avg: 37.5(2)	0.18(13)–15.75(18)<br>avg: 5.9(2)	3.5(2)–68.6(6)<br>avg: 38.9(2)
**$Cu_{29}HPO_3$ (3)** Cu_8-ring	8.1(2)–65.4(2)<br>avg: 40.5(2)	0.81(16)–8.14(17)<br>avg: 2.8(2)	7.5(2)–65.4(2)<br>avg: 40.3(2)
**$Cu_{29}HPO_3$ (3)** Cu_8-ring	4.7(3)–64.3(2)<br>avg: 42.6(2)	1.12(15)–7.62(14)<br>avg: 3.5(2)	4.1(3)–64.3(2)<br>avg: 44.4(3)
Avg. **$Cu_{29}HPO_3$ (3)**	*40.2(2)*	*4.1(2)*	*41.2(2)*
**$Cu_{31}HPO_3$ (4a)** Cu_{14}-ring	9.4(5)–56.9(5)<br>avg: 42.4(5)	0.1(3)–10.4(3)<br>avg: 5.1(4)	9.4(5)–56.7(5)<br>avg: 42.1(5)
**$Cu_{31}HPO_3$ (4a)** Cu_9-ring	29.0(6)–74.7(4)<br>avg: 53.6(5)	0.0(4)–6.6(3)<br>avg: 2.5(4)	28.5(6)–74.7(4)<br>avg: 53.5(5)
**$Cu_{31}HPO_3$ (4a)** Cu_8-ring	24.2(7)–75.6(7)<br>avg: 48.9(5)	0.0(4)–7.7(3)<br>avg: 3.4(4)	23.8(7)–75.6(7)<br>avg: 48.8(5)
Avg. **$Cu_{31}HPO_3$ (4a)**	*48.3(5)*	*3.7(4)*	*48.1(5)*
**$Cu_{31}HPO_3$ (4b)** Cu_{14}-ring	9.3(2)–56.3(3)<br>avg: 41.9(3)	0.7(2)–10.5(2)<br>avg: 4.9(2)	8.8(3)–56.1(3)<br>avg: 41.6(3)
**$Cu_{31}HPO_3$ (4b)** Cu_9-ring	25.8(3)–74.7(2)<br>avg: 53.3(3)	1.3(2)–5.92(14)<br>avg: 2.8(2)	25.7(3)–74.6(2)<br>avg: 53.7(3)
**$Cu_{31}HPO_3$ (4b)** Cu_8-ring	27.5(5)–63.4(2)<br>avg: 44.3(3)	1.67(18)–9.3(4)<br>avg: 4.4(2)	27.2(5)–63.4(2)<br>avg: 44.2(3)
Avg. **$Cu_{31}HPO_3$ (4b)**	*46.5(3)*	*4.0(2)*	*46.5(3)*
**$Cu_{31}HPO_3$ (4c)** Cu_{14}-ring	9.1(4)–57.6(5)<br>avg: 42.2(5)	0.5(3)–10.9(3)<br>avg: 5.1(4)	9.1(4)–57.4(5)<br>avg: 41.9(5)
**$Cu_{31}HPO_3$ (4c)** Cu_9-ring	30.8(5)–75.8(4)<br>avg: 53.4(5)	0.6(4)–6.3(3)<br>avg: 3.0(4)	30.5(5)–75.8(4)<br>avg: 53.4(5)
**$Cu_{31}HPO_3$ (4c)** Cu_8-ring	30.1(5)–64.9(4)<br>avg: 49.2(5)	0.6(3)–11.2(7)<br>avg: 4.7(4)	29.7(4)–64.9(4)<br>avg: 49.0(5)
Avg. **$Cu_{31}HPO_3$ (4c)**	*48.3(5)*	*4.3(4)*	*48.1(5)*
**$Cu_{31}FPO_3$ (5a)** Cu_{14}-ring	2.2(7)–56.0(6)<br>avg: 40.4(7)	0.5(5)–10.8(6)<br>avg: 4.7(6)	2.1(7)–59.5(10)<br>avg: 40.7(7)
**$Cu_{31}FPO_3$ (5a)** Cu_9-ring	36.6(7)–78.8(5)<br>avg: 52.3(7)	0.1(4)–8.2(5)<br>avg: 3.9(5)	36.1(7)–79.0(5)<br>avg: 52.0(7)
**$Cu_{31}FPO_3$ (5a)** Cu_8-ring	18.4(5)–67.0(7)<br>avg: 46.8(7)	0.8(4)–8.7(5)<br>avg: 3.3(5)	18.0(5)–67.2(7)<br>avg: 46.7(7)
Avg. **$Cu_{31}FPO_3$ (5a)**	*46.5(7)*	*4.0(5)*	*46.5(7)*
**$Cu_{31}FPO_3$ (5b)** Cu_{14}-ring	4.8(5)–56.2(3)<br>avg: 41.0(3)	0.1(4)–11.2(3)<br>avg: 5.6(2)	4.8(5)–58.0(4)<br>avg: 41.1(3)
**$Cu_{31}FPO_3$ (5b)** Cu_9-ring	36.4(3)–77.9(2)<br>avg: 52.3(3)	0.3(2)–9.5(2)<br>avg: 4.1(2)	35.8(3)–77.9(2)<br>avg: 52.0(3)
**$Cu_{31}FPO_3$ (5b)** Cu_8-ring	18.1(2)–64.8(3)<br>avg: 47.0(3)	0.07(15)–10.02(18)<br>avg: 3.2(2)	17.5(4)–65.3(3)<br>avg: 46.9(3)
Avg. **$Cu_{31}FPO_3$ (5b)**	*46.8(3)*	*4.3(2)*	*46.7(3)*
**$Cu_{31}FPO_3$ (5c)** Cu_{14}-ring	21.0(6)–55.7(4)<br>avg: 43.2(4)	0.8(3)–15.6(3)<br>avg: 6.2(3)	20.8(6)–67.9(7)<br>avg: 44.2(4)
**$Cu_{31}FPO_3$ (5c)** Cu_9-ring	34.9(4)–70.9(3)<br>avg: 51.7(4)	0.6(2)–8.6(3)<br>avg: 4.5(3)	34.0(5)–71.3(3)<br>avg: 52.1(4)
**$Cu_{31}FPO_3$ (5c)** Cu_8-ring	23.5(4)–64.5(4)<br>avg: 47.1(4)	0.4(3)–7.5(3)<br>avg: 3.6(3)	22.4(4)–64.5(4)<br>avg: 47.3(4)
Avg. **$Cu_{31}FPO_3$ (5c)**	*47.3(4)*	*4.8(3)*	*47.9(4)*

Table S9. Comparison of the dihedral, twist and fold angles (°) between adjacent pyrazolate moieties (as defined in reference 3) in **1** and **2** (based on the major component in the case of disordered moieties).

	DIHEDRAL ANGLE	TWIST ANGLE	FOLD ANGLE	CENTROID-CENTROID DISTANCE	
				D. A. > 35°	D. A. < 35°
**$Cu_{29}HPO_4$ (1)** Cu_{13}-ring UNIT 1	39.8(2)–71.0(2) avg: 54.8(3)	39.0(3)–71.6(3) avg: 55.8(3)	1.5(3)–63.0(5) avg: 21.6(3)	4.757(3)–4.967(3) avg. of 13: 4.835(3)	–
**$Cu_{29}HPO_4$ (1)** Cu_7-ring UNIT 1	26.3(2)–66.0(2) avg: 42.7(2)	5.1(2)–60.3(2) avg: 32.4(2)	11.3(2)–48.5(6) avg: 28.6(2)	4.894(3)–4.949(3) avg of 4: 4.921(3)	5.067(2)–5.089(2) avg of 3: 5.081(3)
**$Cu_{29}HPO_4$ (1)** Cu_9-ring UNIT 1	16.5(2)–47.7(2) avg: 35.8(2)	5.3(2)–49.1(2) avg: 32.5(2)	2.7(3)–42.3(8) avg: 21.3(3)	4.796(3)–5.034(4) avg of 6: 4.927(3)	5.127(3)–5.202(4) avg of 3: 5.157(3)
Avg. **$Cu_{29}HPO_4$ (1)** UNIT 1	*44.4(3)*	*40.2(3)*	*23.8(3)*	*4.894(3)*	*5.119(3)*
**$Cu_{29}HPO_4$ (1)** Cu_{13}-ring UNIT 2	39.3(2)–72.9(2) avg: 55.1(2)	39.0(3)–73.9(2) avg: 56.1(2)	2.0(2)–53.4(3) avg: 20.0(3)	4.777(4)–4.974(3) avg. of 13: 4.837(3)	–
**$Cu_{29}HPO_4$ (1)** Cu_7-ring UNIT 2	25.3(2)–64.1(2) avg: 42.0(2)	4.7(2)–58.3(2) avg: 31.6(2)	15.6(2)–42.3(5) avg: 27.2(2)	4.886(4)–4.961(3) avg of 4: 4.920(3)	5.058(2)–5.092(2) avg of 3: 5.078(3)
**$Cu_{29}HPO_4$ (1)** Cu_9-ring UNIT 2	18.2(2)–46.5(2) avg: 35.5(2)	8.8(2)–47.9(2) avg: 32.5(2)	2.2(3)–39.2(6) avg: 20.3(3)	4.827(3)–5.064(3) avg of 6: 4.935(3)	5.119(3)–5.172(3) avg of 3: 5.140(3)
Avg. **$Cu_{29}HPO_4$ (1)** UNIT 2	*44.2(2)*	*40.1(2)*	*22.5(3)*	*4.897(3)*	*5.109(3)*
**$Cu_{29}HPO_4$ (2)** Cu_{13}-ring	39.84(18)–64.23(17) avg: 51.5(2)	35.96(19)–62.72(18) avg: 51.5(2)	0.0(6)–21.38(19) avg: 12.1(2)	4.755(9)–4.929(5) avg. of 13: 4.830(3)	–
**$Cu_{29}HPO_4$ (2)** Cu_8-ring	26.10(14)–60.72(12) avg: 43.5(2)	7.57(15)–54.86(13) avg: 38.1(2)	6.11(18)–32.81(14) avg: 20.4(2)	4.854(3)–4.957(2) avg of 6: 4.909(3)	5.089(3), 5.091(3) avg of 2: 5.090(3)
**$Cu_{29}HPO_4$ (2)** Cu_8-ring	26.10(14)–60.72(12) avg: 43.5(2)	7.57(15)–54.86(13) avg: 38.1(2)	6.11(18)–32.81(14) avg: 20.4(2)	4.854(3)–4.957(2) avg of 6: 4.909(3)	5.089(3), 5.091(3) avg of 2: 5.090(3)
Avg. **$Cu_{29}HPO_4$ (2)**	*46.2(2)*	*42.6(2)*	*17.6(2)*	*4.883(3)*	*5.090(3)*

Table S10. Comparison of the dihedral, twist and fold angles (°) between adjacent pyrazolate moieties (as defined in reference 3) in **3–5** (based on the major component in the case of disordered moieties).

	DIHEDRAL ANGLE	TWIST ANGLE	FOLD ANGLE	CENTROID-CENTROID DISTANCE	
				D. A. > 35°	D. A. < 35°
**$Cu_{29}HPO_3$ (3)** Cu_{13}-ring	39.5(2)–65.49(18) avg: 51.6(2)	31.3(2)–64.53(19) avg: 50.9(2)	0.5(3)–44.3(3) avg: 14.9(3)	4.664(5)–4.936(6) avg. of 13: 4.841(5)	–
**$Cu_{29}HPO_3$ (3)** Cu_8-ring	24.6(2)–67.26(19) avg: 44.0(2)	10.9(2)–58.4(2) avg: 39.2(2)	3.7(2)–41.7(2) avg: 20.0(2)	4.822(3)–4.947(3) avg of 6: 4.903(4)	5.092(3), 5.142(4) avg of 2: 5.117(4)
**$Cu_{29}HPO_3$ (3)** Cu_8-ring	26.45(17)–56.59(17) avg: 43.9(2)	3.3(2)–52.45(18) avg: 37.8(2)	4.8(2)–42.5(4) avg: 22.4(2)	4.883(3)–4.941(3) avg of 6: 4.914(3)	5.069(3), 5.105(4) avg of 2: 5.087(4)
Avg. **$Cu_{29}HPO_3$ (3)**	*46.5(2)*	*42.6(2)*	*19.1(2)*	*4.886(4)*	*5.102(4)*
**$Cu_{31}HPO_3$ (4a)** Cu_{14}-ring	40.9(5)–63.6(5) avg: 56.0(6)	33.2(6)–65.8(5) avg: 55.7(6)	2.6(7)–32.7(6) avg: 14.5(6)	4.713(8)–4.945(6) avg. of 14: 4.829(7)	–
**$Cu_{31}HPO_3$ (4a)** Cu_9-ring	12.4(6)–58.4(4) avg: 42.7(5)	3.2(6)–57.0(4) avg: 38.0(5)	0.4(5)–36.6(5) avg: 13.6(5)	4.835(6)–5.057(6) avg of 7: 4.915(6)	5.138(6), 5.154(7) avg of 2: 5.146(7)
**$Cu_{31}HPO_3$ (4a)** Cu_8-ring	29.1(5)–63.8(4) avg: 46.6(5)	16.4(5)–62.7(7) avg: 42.1(5)	2.6(5)–31.7(7) avg: 19.2(5)	4.850(6)–5.033(9) avg of 6: 4.964(6)	5.032(6), 5.056(5) avg of 2: 5.044(6)
Avg. **$Cu_{31}HPO_3$ (4a)**	*48.4(5)*	*45.3(5)*	*15.8(5)*	*4.903(6)*	*5.095(6)*
**$Cu_{31}HPO_3$ (4b)** Cu_{14}-ring	38.5(2)–63.7(3) avg: 55.2(3)	32.9(3)–65.0(3) avg: 55.4(3)	3.1(4)–32.6(3) avg: 13.3(3)	4.721(4)–4.934(3) avg. of 14: 4.825(4)	–
**$Cu_{31}HPO_3$ (4b)** Cu_9-ring	6.8(9)–54.0(2) avg: 40.3(3)	3.6(10)–54.1(2) avg: 36.4(3)	0.1(3)–35.9(4) avg: 12.1(3)	4.825(3)–5.036(5) avg of 7: 4.923(4)	5.138(9), 5.152(3) avg of 2: 5.145(4)
**$Cu_{31}HPO_3$ (4b)** Cu_8-ring	27.8(8)–61.2(2) avg: 40.3(3)	15.2(3)–59.1(3) avg: 33.5(3)	2.5(3)–33.2(5) avg: 21.3(3)	4.828(5)–5.019(6) avg of 5: 4.922(4)	4.950(8)–5.040(3) avg of 3: 5.009(4)
Avg. **$Cu_{31}HPO_3$ (4b)**	*45.3(3)*	*41.8(3)*	*15.6(3)*	*4.890(4)*	*5.077(4)*
**$Cu_{31}HPO_3$ (4c)** Cu_{14}-ring	41.7(4)–65.8(5) avg: 56.0(5)	35.7(5)–66.1(5) avg: 55.9(5)	2.8(5)–31.7(5) avg: 14.2(5)	4.711(7)–4.945(6) avg. of 14: 4.829(6)	–
**$Cu_{31}HPO_3$ (4c)** Cu_9-ring	9.5(5)–60.8(4) avg: 42.1(5)	2.4(5)–59.6(4) avg: 38.0(5)	0.8(5)–34.7(5) avg: 13.3(5)	4.843(5)–5.089(6) avg of 7: 4.919(6)	5.122(6), 5.139(6) avg of 2: 5.131(6)
**$Cu_{31}HPO_3$ (4c)** Cu_8-ring	27.8(11)–63.5(4) avg: 41.2(5)	16.4(4)–61.3(4) avg: 34.6(4)	1.1(5)–35.9(9) avg: 21.4(5)	4.843(5)–5.023(8) avg of 5: 4.946(5)	5.040(5)–5.061(12) avg of 3: 5.049(5)
Avg. **$Cu_{31}HPO_3$ (4c)**	*46.4(5)*	*42.8(5)*	*16.3(5)*	*4.898(6)*	*5.090(6)*
**$Cu_{31}FPO_3$ (5a)** Cu_{14}-ring	42.8(9)–67.0(7) avg: 54.6(7)	38.6(9)–68.2(7) avg: 54.7(7)	0.5(9)–57.3(14) avg: 16.3(8)	4.597(14)–4.941(9) avg. of 14: 4.822(9)	–
**$Cu_{31}FPO_3$ (5a)** Cu_9-ring	16.4(6)–66.0(6) avg: 40.6(6)	6.4(6)–64.5(6) avg: 38.3(6)	0.6(7)–22.4(7) avg: 12.4(7)	4.859(7)–4.972(10) avg of 5: 4.889(9)	4.911(8)–5.263(10) avg of 4: 5.081(9)
**$Cu_{31}FPO_3$ (5a)** Cu_8-ring	25.8(5)–56.9(6) avg: 37.2(6)	16.6(6)–54.4(6) avg: 32.4(6)	2.7(6)–24.8(6) avg: 16.8(6)	4.876(10)–4.963(10) avg of 3: 4.905(10)	4.980(10)–5.106(9) avg of 5: 5.052(9)
Avg. **$Cu_{31}FPO_3$ (5a)**	*44.1(6)*	*41.8(6)*	*15.2(7)*	*4.872(9)*	*5.067(9)*
**$Cu_{31}FPO_3$ (5b)** Cu_{14}-ring	45.4(3)–63.3(3) avg: 55.3(3)	45.2(4)–64.5(3) avg: 55.2(3)	0.6(3)–51.3(6) avg: 16.4(3)	4.665(8)–4.912(4) avg. of 14: 4.815(4)	–
**$Cu_{31}FPO_3$ (5b)** Cu_9-ring	13.0(3)–64.3(2) avg: 40.8(3)	4.5(3)–62.1(2) avg: 38.5(3)	1.5(3)–24.6(2) avg: 12.6(3)	4.855(3)–4.947(4) avg of 5: 4.892(4)	4.918(3)–5.200(5) avg of 4: 5.069(4)
**$Cu_{31}FPO_3$ (5b)** Cu_8-ring	26.3(2)–57.9(3) avg: 34.5(3)	15.9(2)–54.3(3) avg: 32.7(3)	1.6(3)–25.5(3) avg: 16.9(3)	4.874(4)–4.936(4) avg of 4: 4.918(4)	5.020(3)–5.116(4) avg of 4: 5.074(4)
Avg. **$Cu_{31}FPO_3$ (5b)**	*43.5(3)*	*42.1(3)*	*15.3(3)*	*4.875(4)*	*5.072(4)*
**$Cu_{31}FPO_3$ (5c)** Cu_{14}-ring	45.3(5)–73.6(4) avg: 56.5(4)	21.7(4)–69.6(4) avg: 54.3(4)	0.5(5)–49.9(7) avg: 24.3(5)	4.683(6)–4.948(6) avg. of 14: 4.822(6)	–
**$Cu_{31}FPO_3$ (5c)** Cu_9-ring	13.0(3)–60.1(4) avg: 39.6(4)	2.4(3)–55.8(4) avg: 35.6(4)	3.4(3)–29.0(4) avg: 16.8(4)	4.819(5)–4.907(6) avg of 6: 4.873(6)	5.032(5)–5.173(5) avg of 3: 5.109(5)
**$Cu_{31}FPO_3$ (5c)** Cu_8-ring	19.9(3)–55.7(3) avg: 37.6(3)	12.0(3)–54.1(3) avg: 32.3(3)	0.8(4)–27.8(3) avg: 17.1(3)	4.894(5)–4.950(5) avg of 4: 4.925(5)	5.009(5)–5.111(4) avg of 4: 5.080(5)
Avg. **$Cu_{31}FPO_3$ (5c)**	*44.6(4)*	*40.7(4)*	*19.4(4)*	*4.873(6)*	*5.095(5)*

Table S11. Copper coordination geometry indexes, $\tau_4 = (360 - \beta - \alpha)/141$ and $\tau_5 = (\beta - \alpha)/60$ (as defined in references 4 and 5) in different Cu_x rings in **1** and **2** (where β and α are the two largest angles in the four- or five-coordinate species).

	τ_4	τ_5
**$Cu_{29}HPO_4$ (1)** Cu_{13}-ring UNIT 1	0.07–0.21 avg: 0.14	0.01–0.12 avg: 0.05
**$Cu_{29}HPO_4$ (1)** Cu_7-ring UNIT 1	0.10–0.21 avg: 0.14	0.00–0.21 avg: 0.11
**$Cu_{29}HPO_4$ (1)** Cu_9-ring UNIT 1	0.03–0.16 avg: 0.11	0.02–0.16 avg: 0.08
Avg. **$Cu_{29}HPO_4$ (1)** UNIT 1	*0.13*	*0.08*
**$Cu_{29}HPO_4$ (1)** Cu_{13}-ring UNIT 2	0.07–0.21 avg: 0.14	0.00–0.11 avg: 0.05
**$Cu_{29}HPO_4$ (1)** Cu_7-ring UNIT 2	0.10–0.20 avg: 0.14	0.01–0.21 avg: 0.11
**$Cu_{29}HPO_4$ (1)** Cu_9-ring UNIT 2	0.03–0.16 avg: 0.11	0.03–0.16 avg: 0.08
Avg. **$Cu_{29}HPO_4$ (1)** UNIT 2	*0.12*	*0.06*
**$Cu_{29}HPO_4$ (2)** Cu_{13}-ring	0.08–0.26 avg: 0.16	0.00–0.26 avg: 0.08
**$Cu_{29}HPO_4$ (2)** Cu_8-ring	0.10–0.21 avg: 0.14	0.04–0.22 avg: 0.08
**$Cu_{29}HPO_4$ (2)** Cu_8-ring	0.10–0.21 avg: 0.14	0.04–0.22 avg: 0.08
Avg. **$Cu_{29}HPO_4$ (2)**	*0.15*	*0.08*

Table S12. Copper coordination geometry indexes, $\tau_4 = (360 - \beta - \alpha)/141$ and $\tau_5 = (\beta - \alpha)/60$ (as defined in references 4 and 5) in different Cu_x rings in **3–5** (where β and α are the two largest angles in the four- or five-coordinate species).

	τ_4	τ_5
**$Cu_{29}HPO_3$ (3)** Cu_{13}-ring	0.07–0.19<br>avg: 0.14	0.00–0.14<br>avg: 0.05
**$Cu_{29}HPO_3$ (3)** Cu_8-ring	0.08–0.21<br>avg: 0.14	0.00–0.34<br>avg: 0.07
**$Cu_{29}HPO_3$ (3)** Cu_8-ring	0.09–0.17<br>avg: 0.13	0.01–0.27<br>avg: 0.09
Avg. **$Cu_{29}HPO_3$ (3)**	*0.14*	*0.07*
**$Cu_{31}HPO_3$ (4a)** Cu_{14}-ring	0.07–0.22<br>avg: 0.15	0.00–0.11<br>avg: 0.04
**$Cu_{31}HPO_3$ (4a)** Cu_9-ring	0.05–0.16<br>avg: 0.08	0.01–0.05<br>avg: 0.03
**$Cu_{31}HPO_3$ (4a)** Cu_8-ring	0.09–0.16<br>avg: 0.12	0.05–0.17<br>avg: 0.12
Avg. **$Cu_{31}HPO_3$ (4a)**	*0.12*	*0.06*
**$Cu_{31}HPO_3$ (4b)** Cu_{14}-ring	0.08–0.22<br>avg: 0.15	0.01–0.11<br>avg: 0.05
**$Cu_{31}HPO_3$ (4b)** Cu_9-ring	0.05–0.16<br>avg: 0.09	0.00–0.10<br>avg: 0.05
**$Cu_{31}HPO_3$ (4b)** Cu_8-ring	0.09–0.17<br>avg: 0.12	0.03–0.20<br>avg: 0.11
Avg. **$Cu_{31}HPO_3$ (4b)**	*0.12*	*0.07*
**$Cu_{31}HPO_3$ (4c)** Cu_{14}-ring	0.08–0.22<br>avg: 0.15	0.01–0.10<br>avg: 0.04
**$Cu_{31}HPO_3$ (4c)** Cu_9-ring	0.05–0.15<br>avg: 0.08	0.00–0.07<br>avg: 0.03
**$Cu_{31}HPO_3$ (4c)** Cu_8-ring	0.09–0.16<br>avg: 0.12	0.02–0.20<br>avg: 0.12
Avg. **$Cu_{31}HPO_3$ (4c)**	*0.12*	*0.06*
**$Cu_{31}FPO_3$ (5a)** Cu_{14}-ring	0.07–0.23<br>avg: 0.15	0.01–0.18<br>avg: 0.07
**$Cu_{31}FPO_3$ (5a)** Cu_9-ring	0.01–0.13<br>avg: 0.08	0.00–0.08<br>avg: 0.03
**$Cu_{31}FPO_3$ (5a)** Cu_8-ring	0.08–0.13<br>avg: 0.11	0.00–0.17<br>avg: 0.10
Avg. **$Cu_{31}FPO_3$ (5a)**	*0.11*	*0.06*
**$Cu_{31}FPO_3$ (5b)** Cu_{14}-ring	0.07–0.24<br>avg: 0.15	0.00–0.16<br>avg: 0.04
**$Cu_{31}FPO_3$ (5b)** Cu_9-ring	0.04–0.13<br>avg: 0.09	0.01–0.09<br>avg: 0.04
**$Cu_{31}FPO_3$ (5b)** Cu_8-ring	0.08–0.12<br>avg: 0.11	0.00–0.19<br>avg: 0.10
Avg. **$Cu_{31}FPO_3$ (5b)**	*0.11*	*0.06*
**$Cu_{31}FPO_3$ (5c)** Cu_{14}-ring	0.08–0.23<br>avg: 0.14	0.01–0.14<br>avg: 0.05
**$Cu_{31}FPO_3$ (5c)** Cu_9-ring	0.05–0.15<br>avg: 0.09	0.00–0.05<br>avg: 0.02
**$Cu_{31}FPO_3$ (5c)** Cu_8-ring	0.08–0.12<br>avg: 0.10	0.01–0.18<br>avg: 0.09
Avg. **$Cu_{31}FPO_3$ (5c)**	*0.11*	*0.06*

Table S13. Comparison of the deviations (Å) of Cu atoms in different Cu_x rings from the Cu_x mean-planes in **1** and **2**.

	**Deviation from Cu_m mean-plane**
**$Cu_{29}HPO_4$ (1)** Cu_{13}-ring UNIT 1	0.049–1.213 avg: 0.651
**$Cu_{29}HPO_4$ (1)** Cu_7-ring UNIT 1	0.044–0.439 avg: 0.280
**$Cu_{29}HPO_4$ (1)** Cu_9-ring UNIT 1	0.235–0.794 avg: 0.556
Avg. **$Cu_{29}HPO_4$ (1)** UNIT 1	*0.496*
**$Cu_{29}HPO_4$ (1)** Cu_{13}-ring UNIT 2	0.047–1.182 avg: 0.636
**$Cu_{29}HPO_4$ (1)** Cu_7-ring UNIT 2	0.049–0.432 avg: 0.275
**$Cu_{29}HPO_4$ (1)** Cu_9-ring UNIT 2	0.196–0.793 avg: 0.540
Avg. **$Cu_{29}HPO_4$ (1)** UNIT 2	*0.484*
**$Cu_{29}HPO_4$ (2)** Cu_{13}-ring	0.006–0.833 avg: 0.370
**$Cu_{29}HPO_4$ (2)** Cu_8-ring	0.029–0.303 avg: 0.158
**$Cu_{29}HPO_4$ (2)** Cu_8-ring	0.029–0.303 avg: 0.158
Avg. **$Cu_{29}HPO_4$ (2)**	*0.229*

Table S14. Comparison of the deviations (Å) of Cu atoms in different Cu_x rings from the Cu_x mean-planes in **3–5**.

	**Deviation from Cu_m mean-plane**
**$Cu_{29}HPO_3$ (3)** Cu_{13}-ring	0.003–0.914<br>avg: 0.379
**$Cu_{29}HPO_3$ (3)** Cu_8-ring	0.026–0.296<br>avg: 0.167
**$Cu_{29}HPO_3$ (3)** Cu_8-ring	0.037–0.317<br>avg: 0.169
Avg. **$Cu_{29}HPO_3$ (3)**	*0.238*
**$Cu_{31}HPO_3$ (4a)** Cu_{14}-ring	0.149–1.667<br>avg: 0.766
**$Cu_{31}HPO_3$ (4a)** Cu_9-ring	0.051–0.848<br>avg: 0.454
**$Cu_{31}HPO_3$ (4a)** Cu_8-ring	0.287–0.555<br>avg: 0.412
Avg. **$Cu_{31}HPO_3$ (4a)**	*0.544*
**$Cu_{31}HPO_3$ (4b)** Cu_{14}-ring	0.097–1.691<br>avg: 0.762
**$Cu_{31}HPO_3$ (4b)** Cu_9-ring	0.100–0.847<br>avg: 0.459
**$Cu_{31}HPO_3$ (4b)** Cu_8-ring	0.306–0.549<br>avg: 0.417
Avg. **$Cu_{31}HPO_3$ (4b)**	*0.546*
**$Cu_{31}HPO_3$ (4c)** Cu_{14}-ring	0.127–1.650<br>avg: 0.759
**$Cu_{31}HPO_3$ (4c)** Cu_9-ring	0.091–0.838<br>avg: 0.464
**$Cu_{31}HPO_3$ (4c)** Cu_8-ring	0.297–0.545<br>avg: 0.413
Avg. **$Cu_{31}HPO_3$ (4c)**	*0.545*
**$Cu_{31}FPO_3$ (5a)** Cu_{14}-ring	0.090–1.496<br>avg: 0.709
**$Cu_{31}FPO_3$ (5a)** Cu_9-ring	0.051–0.750<br>avg: 0.419
**$Cu_{31}FPO_3$ (5a)** Cu_8-ring	0.267–0.516<br>avg: 0.391
Avg. **$Cu_{31}FPO_3$ (5a)**	*0.506*
**$Cu_{31}FPO_3$ (5b)** Cu_{14}-ring	0.063–1.483<br>avg: 0.708
**$Cu_{31}FPO_3$ (5b)** Cu_9-ring	0.047–0.766<br>avg: 0.416
**$Cu_{31}FPO_3$ (5b)** Cu_8-ring	0.262–0.527<br>avg: 0.393
Avg. **$Cu_{31}FPO_3$ (5b)**	*0.506*
**$Cu_{31}FPO_3$ (5c)** Cu_{14}-ring	0.007–1.619<br>avg: 0.717
**$Cu_{31}FPO_3$ (5c)** Cu_9-ring	0.022–0.784<br>avg: 0.422
**$Cu_{31}FPO_3$ (5c)** Cu_8-ring	0.249–0.527<br>avg: 0.383
Avg. **$Cu_{31}FPO_3$ (5c)**	*0.507*

Table S15. Selected bond lengths for UNIT 1 of **1** (Cu1_3–Cu9_3: Cu_9-ring; Cu10_3–Cu22_3: Cu_{13}-ring; Cu23_3–Cu29_3: Cu_7-ring; O1_1–O4_1: incarcerated HPO_4^{2-} anion).

P1_1–O1_1 1.516(3)	Cu7_3–N12_3 2.013(4)	Cu15_3–O15_3 1.913(3)	Cu22_3–N42_3 1.972(3)
P1_1–O2_1 1.610(3)	Cu7_3–O18_3 2.375(3)	Cu15_3–O14_3 1.918(3)	Cu23_3–O29_3 1.920(3)
P1_1–O3_1 1.519(3)	Cu8_3–O7_3 1.887(3)	Cu15_3–N29_3 1.954(4)	Cu23_3–O23_3 1.960(3)
P1_1–O4_1 1.503(3)	Cu8_3–O8_3 1.914(3)	Cu15_3–N28_3 1.976(4)	Cu23_3–N45_3 1.972(3)
Cu1_3–O9_3 1.915(3)	Cu8_3–N15_3 1.970(4)	Cu16_3–O15_3 1.915(3)	Cu23_3–N58_3 2.006(3)
Cu1_3–O1_3 1.943(3)	Cu8_3–N14_3 1.973(4)	Cu16_3–O16_3 1.924(4)	Cu23_3–O22_3 2.388(3)
Cu1_3–N1_3 1.969(3)	Cu9_3–O9_3 1.933(3)	Cu16_3–N30_3 1.954(5)	Cu24_3–O23_3 1.923(3)
Cu1_3–N18_3 1.982(4)	Cu9_3–O8_3 1.952(3)	Cu16_3–N31_3 1.966(4)	Cu24_3–O24_3 1.942(3)
Cu2_3–O2_3 1.904(3)	Cu9_3–N16_3 2.001(4)	Cu17_3–O17_3 1.909(3)	Cu24_3–N47_3 2.000(3)
Cu2_3–O1_3 1.929(3)	Cu9_3–N17_3 2.017(4)	Cu17_3–O16_3 1.933(3)	Cu24_3–N46_3 2.016(3)
Cu2_3–N2_3 1.959(3)	Cu9_3–O21_3 2.365(3)	Cu17_3–N32_3 1.953(4)	Cu25_3–O24_3 1.937(3)
Cu2_3–N3_3 1.968(3)	Cu10_3–O22_3 1.918(3)	Cu17_3–N33_3 1.984(4)	Cu25_3–O25_3 1.947(3)
Cu3_3–O3_3 1.929(3)	Cu10_3–O10_3 1.937(3)	Cu18_3–O18_3 1.930(3)	Cu25_3–N49_3 1.975(3)
Cu3_3–O2_3 1.943(3)	Cu10_3–N19_3 1.964(4)	Cu18_3–O17_3 1.941(3)	Cu25_3–N48_3 2.009(3)
Cu3_3–N4_3 1.996(3)	Cu10_3–N44_3 1.969(4)	Cu18_3–N34_3 1.956(4)	Cu26_3–O25_3 1.929(3)
Cu3_3–N5_3 2.009(3)	Cu11_3–O10_3 1.916(3)	Cu18_3–N35_3 1.976(4)	Cu26_3–O26_3 1.931(3)
Cu3_3–O12_3 2.422(3)	Cu11_3–O11_3 1.944(3)	Cu19_3–O18_3 1.918(3)	Cu26_3–N51_3 1.980(3)
Cu4_3–O3_3 1.915(3)	Cu11_3–N20_3 1.966(4)	Cu19_3–O19_3 1.937(3)	Cu26_3–N50_3 1.984(4)
Cu4_3–O4_3 1.945(3)	Cu11_3–N21_3 1.967(4)	Cu19_3–N37_3 1.968(4)	Cu27_3–O27_3 1.930(3)
Cu4_3–N7_3 1.999(3)	Cu12_3–O12_3 1.919(3)	Cu19_3–N36_3 1.982(4)	Cu27_3–O26_3 1.961(3)
Cu4_3–N6_3 2.012(4)	Cu12_3–O11_3 1.928(3)	Cu20_3–O20_3 1.920(3)	Cu27_3–N52_3 1.971(4)
Cu5_3–O4_3 1.909(3)	Cu12_3–N22_3 1.956(4)	Cu20_3–O19_3 1.931(3)	Cu27_3–N53_3 2.024(3)
Cu5_3–O5_3 1.909(3)	Cu12_3–N23_3 1.959(3)	Cu20_3–N38_3 1.980(4)	Cu27_3–O17_3 2.398(3)
Cu5_3–N9_3 1.960(4)	Cu13_3–O13_3 1.918(3)	Cu20_3–N39_3 1.987(4)	Cu28_3–O28_3 1.891(3)
Cu5_3–N8_3 1.977(4)	Cu13_3–O12_3 1.924(3)	Cu21_3–O20_3 1.923(3)	Cu28_3–O27_3 1.922(3)
Cu6_3–O6_3 1.905(3)	Cu13_3–N25_3 1.960(3)	Cu21_3–O21_3 1.931(3)	Cu28_3–N55_3 1.982(4)
Cu6_3–O5_3 1.919(3)	Cu13_3–N24_3 1.960(3)	Cu21_3–N40_3 1.972(3)	Cu28_3–N54_3 1.987(3)
Cu6_3–N10_3 1.966(4)	Cu14_3–O13_3 1.913(3)	Cu21_3–N41_3 1.984(4)	Cu29_3–O28_3 1.907(3)
Cu6_3–N11_3 1.993(4)	Cu14_3–O14_3 1.919(3)	Cu22_3–O21_3 1.916(3)	Cu29_3–O29_3 1.913(3)
Cu7_3–O7_3 1.932(3)	Cu14_3–N26_3 1.954(4)	Cu22_3–O22_3 1.932(3)	Cu29_3–N56_3 1.971(3)
Cu7_3–O6_3 1.937(3)	Cu14_3–N27_3 1.957(4)	Cu22_3–N43_3 1.959(4)	Cu29_3–N57_3 1.990(3)
Cu7_3–N13_3 2.013(4)			

Table S16. Selected bond lengths for UNIT 2 of **1** (Cu1_4–Cu9_4: Cu_9-ring; Cu10_4–Cu22_4: Cu_{13}-ring; Cu23_4–Cu29_4: Cu_7-ring; O1_2–O4_2: incarcerated HPO_4^{2-} anion).

P1_2–O1_2 1.518(3)	Cu7_4–N13_4 1.996(3)	Cu15_4–O15_4 1.917(3)	Cu22_4–N43_4 1.970(3)
P1_2–O2_2 1.607(3)	Cu7_4–N12_4 2.007(3)	Cu15_4–O14_4 1.922(3)	Cu23_4–O23_4 1.910(3)
P1_2–O3_2 1.517(3)	Cu7_4–O18_4 2.428(3)	Cu15_4–N28_4 1.961(4)	Cu23_4–O29_4 1.914(3)
P1_2–O4_2 1.505(3)	Cu8_4–O7_4 1.902(3)	Cu15_4–N29_4 1.974(4)	Cu23_4–N45_4 1.974(3)
Cu1_4–O9_4 1.936(3)	Cu8_4–O8_4 1.926(3)	Cu16_4–O15_4 1.913(3)	Cu23_4–N58_4 1.995(4)
Cu1_4–O1_4 1.949(3)	Cu8_4–N15_4 1.958(3)	Cu16_4–O16_4 1.924(3)	Cu24_4–O23_4 1.880(3)
Cu1_4–N18_4 2.004(4)	Cu8_4–N14_4 1.972(3)	Cu16_4–N30_4 1.952(4)	Cu24_4–O24_4 1.919(3)
Cu1_4–N1_4 2.012(4)	Cu9_4–O9_4 1.917(3)	Cu16_4–N31_4 1.975(4)	Cu24_4–N46_4 1.982(4)
Cu1_4–O22_4 2.375(3)	Cu9_4–O8_4 1.950(3)	Cu17_4–O17_4 1.911(3)	Cu24_4–N47_4 1.983(4)
Cu2_4–O2_4 1.887(3)	Cu9_4–N16_4 1.973(3)	Cu17_4–O16_4 1.917(3)	Cu25_4–O24_4 1.934(3)
Cu2_4–O1_4 1.918(3)	Cu9_4–N17_4 1.976(3)	Cu17_4–N32_4 1.945(4)	Cu25_4–O25_4 1.961(3)
Cu2_4–N2_4 1.967(4)	Cu10_4–O10_4 1.924(3)	Cu17_4–N33_4 1.961(4)	Cu25_4–N49_4 1.975(3)
Cu2_4–N3_4 1.972(4)	Cu10_4–O22_4 1.929(3)	Cu18_4–O17_4 1.921(3)	Cu25_4–N48_4 2.032(3)
Cu3_4–O2_4 1.920(3)	Cu10_4–N19_4 1.966(3)	Cu18_4–O18_4 1.922(3)	Cu25_4–O13_4 2.390(3)
Cu3_4–O3_4 1.934(3)	Cu10_4–N44_4 1.978(4)	Cu18_4–N35_4 1.959(3)	Cu26_4–O25_4 1.928(3)
Cu3_4–N4_4 2.004(4)	Cu11_4–O10_4 1.916(3)	Cu18_4–N34_4 1.961(3)	Cu26_4–O26_4 1.933(3)
Cu3_4–N5_4 2.022(4)	Cu11_4–O11_4 1.940(3)	Cu19_4–O18_4 1.923(3)	Cu26_4–N51_4 1.984(4)
Cu3_4–O12_4 2.379(3)	Cu11_4–N20_4 1.980(4)	Cu19_4–O19_4 1.932(3)	Cu26_4–N50_4 1.984(3)
Cu4_4–O3_4 1.900(3)	Cu11_4–N21_4 1.981(4)	Cu19_4–N37_4 1.956(4)	Cu27_4–O27_4 1.943(3)
Cu4_4–O4_4 1.922(3)	Cu12_4–O12_4 1.921(3)	Cu19_4–N36_4 1.956(3)	Cu27_4–O26_4 1.945(3)
Cu4_4–N7_4 1.967(4)	Cu12_4–O11_4 1.935(3)	Cu20_4–O20_4 1.918(3)	Cu27_4–N52_4 1.981(3)
Cu4_4–N6_4 1.981(4)	Cu12_4–N22_4 1.961(4)	Cu20_4–O19_4 1.935(3)	Cu27_4–N53_4 2.003(3)
Cu5_4–O4_4 1.909(3)	Cu12_4–N23_4 1.991(4)	Cu20_4–N39_4 1.962(3)	Cu28_4–O28_4 1.927(3)
Cu5_4–O5_4 1.911(3)	Cu13_4–O12_4 1.925(3)	Cu20_4–N38_4 1.967(3)	Cu28_4–O27_4 1.936(3)
Cu5_4–N8_4 1.966(4)	Cu13_4–O13_4 1.934(3)	Cu21_4–O21_4 1.917(3	Cu28_4–N54_4 2.002(3)
Cu5_4–N9_4 1.975(4)	Cu13_4–N24_4 1.969(4)	Cu21_4–O20_4 1.935(3)	Cu28_4–N55_4 2.014(3)
Cu6_4–O6_4 1.918(3)	Cu13_4–N25_4 1.971(4)	Cu21_4–N40_4 1.965(3)	Cu29_4–O29_4 1.919(3)
Cu6_4–O5_4 1.944(3)	Cu14_4–O13_4 1.920(3)	Cu21_4–N41_4 1.971(3)	Cu29_4–O28_4 1.961(3)
Cu6_4–N10_4 1.994(4)	Cu14_4–O14_4 1.933(3)	Cu22_4–O22_4 1.917(3)	Cu29_4–O21_4 2.389(3)
Cu6_4–N11_4 2.022(3)	Cu14_4–N27_4 1.963(4)	Cu22_4–O21_4 1.939(3)	Cu29_4–N56_4 1.970(3)
Cu7_4–O6_4 1.925(3)	Cu14_4–N26_4 1.976(4)	Cu22_4–N42_4 1.961(4)	Cu29_4–N57_4 2.008(3)
Cu7_4–O7_4 1.939(3)			

Table S17. Selected bond lengths for **2** (Cu1_2–Cu8_2 and Cu1_2^{i}–Cu8_2^{i}: Cu_8-rings; Cu9_2–Cu15_2 and Cu9_2^{i}–Cu14_2^{i}: Cu_{13}-ring; O1_1–O4_1: entrapped HPO_4^{2-} anion disordered over two positions around a C_2 axis). Symmetry code: (i) $-x+1, y, -z+1/2$.

P1_1–O3_1 1.455(4)	Cu4_2–N7_2 1.969(3)	Cu8_2–N14_2 1.962(2)	Cu22_2–O11_2 1.778(7)
P1_1–O2_1 1.482(4)	Cu4_2–N6_2 1.974(2)	Cu8_2–N15_2 1.990(3)	Cu22_2–O12_2 1.823(7)
P1_1–O1_1 1.546(5)	Cu4_2–O4B_2 2.026(5)	Cu9_2–O9_2^{i} 1.923(2)	Cu13_2–O13_2 1.9240(19)
P1_1–O4_1 1.611(5)	Cu5_2–O4B_2 1.793(4)	Cu9_2–O9_2 1.924(2)	Cu13_2–O12_2 1.925(2)
Cu1_2–O8_2 1.939(2)	Cu5_2–O5_2 1.905(2)	Cu9_2–N17_2 1.972(3)	Cu13_2–N24_2 1.956(3)
Cu1_2–O1_2 1.941(2)	Cu5_2–N8_2 1.977(2)	Cu9_2–N17_2^{i} 1.972(3)	Cu13_2–N24B_2 1.977(14)
Cu1_2–N1_2 1.999(3)	Cu5_2–N9_2 1.978(3)	Cu10_2–O10_2 1.921(2)	Cu13_2–N25_2 1.979(3)
Cu1_2–N16_2 2.010(2)	Cu5_2–O4_2 2.001(5)	Cu10_2–O9_2 1.924(2)	Cu14_2–O13_2 1.928(2)
Cu1_2–O9_2 2.338(2)	Cu6_2–O5_2 1.931(3)	Cu10_2–N19_2 1.965(3)	Cu14_2–O14_2 1.931(2)
Cu2_2–O2_2 1.877(3)	Cu6_2–O6_2 1.945(2)	Cu10_2–N18_2 1.969(3)	Cu14_2–N26_2 1.976(3)
Cu2_2–O1_2 1.913(2)	Cu6_2–N10_2 1.995(3)	Cu11_2–N21B_2 1.919(15)	Cu14_2–N27_2 1.996(8)
Cu2_2–N3_2 1.956(3)	Cu6_2–N11_2 2.009(3)	Cu11_2–O10_2 1.920(2)	Cu15_2–N28_2 1.967(4)
Cu2_2–N2_2 1.982(3)	Cu6_2–O13_2^{i} 2.380(2)	Cu11_2–O11_2 1.934(2)	Cu15_2–O15_2 1.928(4)
Cu3_2–O2_2 1.923(2)	Cu7_2–O7_2 1.914(2)	Cu11_2–N21_2 1.984(3)	Cu15_2–O14_2 1.955(2)
Cu3_2–O3_2 1.946(2)	Cu7_2–O6_2 1.920(2)	Cu11_2–N20_2 1.992(3)	Cu15_2–N29_2 1.972(5)
Cu3_2–N4_2 1.984(3)	Cu7_2–N12_2 1.977(3)	Cu12_2–O11_2 1.963(2)	Cu16_2–O15_2 1.925(4)
Cu3_2–N5_2 1.989(3)	Cu7_2–N13_2 1.980(3)	Cu12_2–O12_2 1.926(2)	Cu16_2–N30_2 1.982(5)
Cu4_2–O4_2 1.871(5)	Cu8_2–O7_2 1.917(2)	Cu12_2–N22_2 1.956(3)	Cu16_2–N28B_2 1.982(5)
Cu4_2–O3_2 1.925(2)	Cu8_2–O8_2 1.921(2)	Cu12_2–N23_2 1.976(3)	

Table S18. Selected bond lengths for **3** (Cu1–Cu8: Cu_8-ring; Cu9–Cu21: Cu_{13}-ring; Cu22–Cu29: Cu_8-ring; O30–O33 and O30B–O33B: HPO_3^{2-} anion disordered over two positions with 0.63/0.37 occupancy).

P1–O30 1.456(9)	Cu7–O7 1.897(3)	Cu14–N27 1.950(4)	Cu22–N43 1.976(4)
P1–O31 1.524(10)	Cu7–O6 1.919(3)	Cu14–N26B 2.034(10)	Cu22–N58 1.978(4)
P1–O32 1.470(9)	Cu7–N13 1.965(4)	Cu15–O15 1.906(3)	Cu23–O23 1.897(3)
P1B–O30B 1.457(11)	Cu7–N12 1.997(4)	Cu15–O14 1.942(3)	Cu23–O22 1.927(3)
P1B–O31B 1.500(14)	Cu8–O7 1.897(3)	Cu15–N28 1.970(4)	Cu23–N45 1.975(4)
P1B–O32B 1.481(13)	Cu8–O8 1.925(3)	Cu15–N29 2.004(4)	Cu23–N44 1.981(4)
Cu1–O8 1.931(3)	Cu8–N15 1.973(4)	Cu16–O16 1.923(3)	Cu24–O23 1.904(3)
Cu1–O1 1.936(3)	Cu8–N14 1.991(4)	Cu16–O15 1.926(3)	Cu24–O24 1.917(3)
Cu1–N1 1.987(4)	Cu9–O21 1.921(3)	Cu16–N30 1.959(4)	Cu24–N46 1.971(3)
Cu1–N16 2.018(4)	Cu9–O9 1.928(3)	Cu16–N31 1.974(4)	Cu24–N47 1.987(4)
Cu1–O21 2.377(3)	Cu9–N17 1.958(4)	Cu17–O16 1.922(3)	Cu25–O24 1.933(3)
Cu2–O1 1.895(3)	Cu9–N42 1.983(4)	Cu17–O17 1.923(3)	Cu25–O25 1.947(3)
Cu2–O2 1.907(3)	Cu10–O10 1.921(3)	Cu17–N33 1.964(4)	Cu25–N48 1.999(4)
Cu2–N3 1.970(4)	Cu10–O9 1.929(3)	Cu17–N32 1.976(4)	Cu25–N49 2.001(4)
Cu2–N2 1.974(4)	Cu10–N19 1.984(4)	Cu18–O18 1.914(3)	Cu25–O12 2.404(3)
Cu3–O2 1.932(3)	Cu10–N18 1.987(4)	Cu18–O17 1.932(3)	Cu26–O26 1.892(3)
Cu3–O3 1.938(3)	Cu11–O10 1.918(3)	Cu18–N34 1.965(4)	Cu26–O25 1.930(3)
Cu3–N5 1.967(4)	Cu11–O11 1.922(3)	Cu18–N35 1.970(4)	Cu26–N50 1.966(4)
Cu3–N4 1.976(4)	Cu11–N21 1.956(4)	Cu19–O18 1.921(3)	Cu26–N51 1.977(4)
Cu4–O4 1.909(3)	Cu11–N20 1.971(4)	Cu19–O19 1.922(3)	Cu27–O26 1.911(3)
Cu4–O3 1.933(3)	Cu12–O12 1.924(3)	Cu19–N37 1.986(4)	Cu27–O27 1.926(3)
Cu4–N7 1.982(4)	Cu12–O11 1.930(3)	Cu19–N36 1.992(4)	Cu27–N52 1.968(4)
Cu4–N6 1.985(4)	Cu12–N22 1.964(4)	Cu20–O20 1.909(3)	Cu27–N53 1.983(4)
Cu5–O4 1.874(3)	Cu12–N23 1.970(4)	Cu20–O19 1.950(3)	Cu28–O27 1.931(3)
Cu5–O5 1.907(3)	Cu13–O12 1.921(3)	Cu20–N38 1.949(4)	Cu28–O28 1.940(3)
Cu5–N8 1.955(4)	Cu13–O13 1.935(3)	Cu20–N39 1.985(4)	Cu28–N55 1.996(4)
Cu5–N9 1.976(4)	Cu13–N25 1.958(12)	Cu21–O21 1.929(3)	Cu28–N54 2.013(4)
Cu6–O5 1.936(3)	Cu13–N25B 1.978(11)	Cu21–O20 1.930(3)	Cu28–O17 2.347(3)
Cu6–O6 1.954(3)	Cu13–N24 1.980(4)	Cu21–N40 1.954(4)	Cu29–O29 1.887(3)
Cu6–N11 1.999(4)	Cu14–O13 1.912(3)	Cu21–N41 1.975(4)	Cu29–O28 1.910(3)
Cu6–N10 2.005(4)	Cu14–O14 1.938(3)	Cu22–O29 1.909(3)	Cu29–N57 1.959(4)
Cu6–O16 2.331(3)	Cu14–N26 1.941(10)	Cu22–O22 1.953(3)	Cu29–N56 1.968(4)

Table S19. Selected bond lengths for **4a** (Cu1–Cu9: Cu_9-ring; Cu10–Cu23: Cu_{14}-ring; Cu24–Cu31: Cu_8-ring; O32–O34 and O32B–O34B: HPO_3^{2-} anion disordered over two positions with 0.85/0.15 occupancy).

P1–O32 1.528(9)	Cu8–O8 1.920(6)	Cu16–N30 1.969(8)	Cu25–O24 1.888(6)
P1–O33 1.500(8)	Cu8–O7 1.924(6)	Cu16–N31 1.980(7)	Cu25–O25 1.957(6)
P1–O34 1.529(7)	Cu8–N15 1.963(7)	Cu17–O17 1.898(6)	Cu25–N48 2.001(7)
P1B–O32B 1.529(19)	Cu8–N14 1.963(8)	Cu17–O16 1.923(6)	Cu25–N49 1.921(16)
P1B–O33B 1.495(19)	Cu9–O8 1.930(6)	Cu17–N32 1.949(7)	Cu25–N49B 2.034(16)
P1B–O34B 1.517(19)	Cu9–O9 1.986(6)	Cu17–N33 1.963(7)	Cu26–O26 1.915(5)
Cu1–O1 1.931(6)	Cu9–O22 2.335(5)	Cu18–O17 1.917(6)	Cu26–O25 1.940(7)
Cu1–O9 1.936(6)	Cu9–N17 1.975(7)	Cu18–O18 1.928(6)	Cu26–N50 1.981(15)
Cu1–N1 1.950(8)	Cu9–N16 1.981(7)	Cu18–N35 1.963(7)	Cu26–N50B 1.992(15)
Cu1–N18 1.967(8)	Cu10–O23 1.904(6)	Cu18–N34 1.967(8)	Cu26–N51 1.866(16)
Cu2–O2 1.916(6)	Cu10–O10 1.923(6)	Cu19–O18 1.906(6)	Cu26–N51B 2.118(16
Cu2–O1 1.922(6)	Cu10–N19 1.969(8)	Cu19–O19 1.928(6)	Cu27–O26 1.928(6)
Cu2–N3 1.953(8)	Cu10–N46 1.972(7)	Cu19–N37 1.947(8)	Cu27–O27 1.960(6)
Cu2–N2 1.977(7)	Cu11–O10 1.911(6)	Cu19–N36 1.969(7)	Cu27–N52 1.929(15)
Cu3–O3 1.939(5)	Cu11–O11 1.912(6)	Cu20–O19 1.912(6)	Cu27–N52B 2.108(17)
Cu3–O2 1.939(6)	Cu11–N21 1.951(8)	Cu20–O20 1.948(6)	Cu27–N53 1.986(7)
Cu3–N4 2.005(7)	Cu11–N20 1.964(9)	Cu20–N39 1.961(8)	Cu27–O14 2.427(6)
Cu3–N5 2.010(7)	Cu12–O12 1.922(6)	Cu20–N38 1.982(8)	Cu28–O28 1.880(6)
Cu4–O4 1.911(6)	Cu12–O11 1.948(7)	Cu21–O20 1.919(6)	Cu28–O27 1.922(5)
Cu4–O3 1.924(6)	Cu12–N23 1.960(8)	Cu21–O21 1.923(6)	Cu28–N54 1.960(7)
Cu4–N7 1.986(7)	Cu12–N22 1.968(8)	Cu21–N40 1.961(8)	Cu28–N55 1.961(7)
Cu4–N6 1.993(8)	Cu13–O12 1.917(6)	Cu21–N41 1.975(9)	Cu29–O28 1.918(6)
Cu5–O4 1.906(6)	Cu13–O13 1.917(6)	Cu22–O22 1.922(6)	Cu29–O29 1.929(5)
Cu5–O5 1.915(6)	Cu13–N25 1.955(7)	Cu22–O21 1.927(5)	Cu29–N56 1.961(7)
Cu5–N8 1.949(7)	Cu13–N24 1.961(8)	Cu22–N42 1.957(8)	Cu29–N57 1.996(7)
Cu5–N9 1.977(7)	Cu14–O13 1.910(6)	Cu22–N43 1.980(7)	Cu29–O17 2.419(6)
Cu6–O6 1.931(6)	Cu14–O14 1.917(6)	Cu23–O23 1.895(6)	Cu30–O29 1.904(5)
Cu6–O5 1.956(6)	Cu14–N26 1.949(8)	Cu23–O22 1.932(5)	Cu30–O30 1.942(5)
Cu6–N10 1.986(7)	Cu14–N27 1.951(8)	Cu23–N44 1.931(7)	Cu30–N58 1.971(7)
Cu6–N11 1.992(8)	Cu15–O15 1.909(6)	Cu23–N45 1.974(7)	Cu30–N59 1.982(7)
Cu6–O18 2.355(6)	Cu15–O14 1.930(6)	Cu24–O24 1.908(6)	Cu31–O31 1.906(5)
Cu7–O6 1.896(6)	Cu15–N28 1.940(8)	Cu24–O31 1.948(5)	Cu31–O30 1.952(5)
Cu7–O7 1.941(6)	Cu15–N29 1.970(7)	Cu24–N47 1.980(7)	Cu31–N60 1.967(7)
Cu7–N13 1.946(8)	Cu16–O16 1.921(6)	Cu24–N62 1.996(7)	Cu31–N61 1.990(7)
Cu7–N12 1.981(7)	Cu16–O15 1.922(5)		

Table S20. Selected bond lengths for **4b** (Cu1–Cu9: Cu_9-ring; Cu10–Cu23: Cu_{14}-ring; Cu24–Cu31: Cu_8-ring; O32–O34 and O32B–O34B: HPO_3^{2-} anion disordered over two positions with 0.75/0.25 occupancy).

P1–O32 1.513(7)	Cu7–N12 1.977(4)	Cu16–N31 1.973(3)	Cu25–N48 1.991(3)
P1–O33 1.516(7)	Cu8–O8 1.915(3)	Cu16–N30 1.973(3)	Cu25–N49 1.885(12)
P1–O34 1.522(6)	Cu8–O7 1.932(3)	Cu17–O17 1.900(3)	Cu25–N49B 2.050(11)
P1B–O34B 1.488(15)	Cu8–N14 1.955(4)	Cu17–O16 1.922(3)	Cu26–O26 1.915(3)
P1B–O33B 1.503(15)	Cu8–N15 1.966(4)	Cu17–N32 1.950(3)	Cu26–O25 1.951(3)
P1B–O32B 1.526(15)	Cu9–O8 1.928(3)	Cu17–N33 1.972(4)	Cu26–O42 2.02(2)
Cu1–O9 1.927(3)	Cu9–O9 1.983(3)	Cu18–O17 1.922(3)	Cu26–N50 1.997(12)
Cu1–O1 1.927(3)	Cu9–O38 1.947(16)	Cu18–O18 1.926(3)	Cu26–N50B 1.964(12)
Cu1–O39 1.931(12)	Cu9–O22 2.339(3)	Cu18–N35 1.956(4)	Cu26–N51 1.915(12)
Cu1–N1 1.945(4)	Cu9–N16 1.977(4)	Cu18–N34 1.969(4)	Cu26–N51B 2.048(15)
Cu1–N18 1.989(7)	Cu9–N17 1.994(10)	Cu19–O18 1.921(3)	Cu27–O26 1.925(3)
Cu2–O2 1.918(3)	Cu10–O23 1.905(3)	Cu19–O19 1.925(3)	Cu27–O27 1.949(3)
Cu2–O1 1.928(3)	Cu10–O10 1.928(3)	Cu19–N37 1.956(4)	Cu27–O43 1.93(2)
Cu2–N3 1.972(4)	Cu10–N19 1.963(4)	Cu19–N36 1.976(4)	Cu27–O14 2.437(3)
Cu2–N2 1.975(3)	Cu10–N46 1.974(4)	Cu20–O19 1.920(3)	Cu27–N52 1.943(13)
Cu3–O3 1.930(3)	Cu11–O10 1.919(3)	Cu20–O20 1.935(3)	Cu27–N52B 2.086(15)
Cu3–O2 1.935(3)	Cu11–O11 1.927(3)	Cu20–N39 1.955(4)	Cu27–N53 1.978(3)
Cu3–O36 1.959(5)	Cu11–N21 1.959(4)	Cu20–N38 1.970(4)	Cu28–O28 1.895(3)
Cu3–N4 1.981(3)	Cu11–N20 1.959(4)	Cu21–O20 1.928(3)	Cu28–O27 1.920(3)
Cu3–N5 2.053(19)	Cu12–O12 1.918(3)	Cu21–O21 1.933(3)	Cu28–N55 1.963(3)
Cu4–O4 1.914(3)	Cu12–O11 1.935(3)	Cu21–N40 1.955(4	Cu28–N54 1.971(3)
Cu4–O3 1.922(3)	Cu12–N23 1.964(4)	Cu21–N41 1.971(4)	Cu29–O29 1.927(3)
Cu4–O37 1.964(5)	Cu12–N22 1.964(4)	Cu22–O22 1.922(3)	Cu29–O28 1.933(3)
Cu4–N7 1.970(3)	Cu13–O13 1.907(3)	Cu22–O21 1.933(3)	Cu29–O40 2.01(2)
Cu4–N6 2.064(18)	Cu13–O12 1.922(3)	Cu22–N42 1.961(4)	Cu29–O17 2.421(3)
Cu5–O4 1.900(3)	Cu13–N24 1.948(4)	Cu22–N43 1.966(4)	Cu29–N56 1.966(3)
Cu5–O5 1.917(3)	Cu13–N25 1.964(4)	Cu23–O23 1.906(3)	Cu29–N57 2.001(6)
Cu5–N8 1.948(4)	Cu14–O13 1.909(3)	Cu23–O22 1.928(3)	Cu30–O29 1.927(3)
Cu5–N9 1.972(4)	Cu14–O14 1.911(3)	Cu23–N44 1.951(4)	Cu30–O30 1.948(3)
Cu6–O6 1.936(3)	Cu14–N26 1.953(4)	Cu23–N45 1.976(4)	Cu30–O41 1.960(14)
Cu6–O5 1.954(3)	Cu14–N27 1.961(4)	Cu24–O24 1.913(3)	Cu30–N59 1.978(3)
Cu6–N10 1.985(4)	Cu15–O15 1.915(3)	Cu24–O31 1.935(3)	Cu30–N58 1.997(5)
Cu6–N11 1.989(4)	Cu15–O14 1.922(3)	Cu24–N47 1.976(4)	Cu31–O31 1.932(3)
Cu6–O18 2.357(3)	Cu15–N29 1.953(3)	Cu24–N62 1.996(4)	Cu31–O30 1.946(3)
Cu7–O6 1.899(3)	Cu15–N28 1.969(4)	Cu25–O24 1.885(3)	Cu31–N60 1.975(3)
Cu7–O7 1.934(3)	Cu16–O15 1.912(3)	Cu25–O25 1.948(3)	Cu31–N61 1.988(3)
Cu7–N13 1.945(4)	Cu16–O16 1.920(3)		

Table S21. Selected bond lengths for **4c** (Cu1–Cu9: Cu_9-ring; Cu10–Cu23: Cu_{14}-ring; Cu24–Cu31: Cu_8-ring; O32–O34 and O32B–O34B: HPO_3^{2-} anion disordered over two positions with 0.77/0.23 occupancy).

P1–O32 1.502(7)	Cu7–O18 2.330(5)	Cu16–N31 1.961(7)	Cu24–O10 2.406(5)
P1–O33 1.510(8)	Cu8–O8 1.918(5)	Cu16–N30 1.979(6)	Cu25–O25 1.892(5)
P1–O34 1.522(8)	Cu8–O7 1.923(5)	Cu17–O17 1.919(5)	Cu25–O24 1.917(5)
P1B–O32B 1.496(16)	Cu8–N15 1.945(7)	Cu17–O16 1.927(6)	Cu25–N49 1.958(6)
P1B–O33B 1.498(16)	Cu8–N14 1.959(8)	Cu17–N32 1.950(7)	Cu25–N48 1.966(6)
P1B–O34B 1.509(16)	Cu9–O9 1.924(5)	Cu17–N33 1.955(7)	Cu26–O26 1.926(5)
Cu1–O9 1.935(5)	Cu9–O8 1.930(5)	Cu18–O18 1.927(5)	Cu26–O25 1.929(5)
Cu1–O1 1.938(5)	Cu9–N17 1.972(6)	Cu18–O17 1.928(5)	Cu26–N50 1.967(6)
Cu1–N18 1.996(6)	Cu9–N16 1.972(7)	Cu18–N34 1.954(7)	Cu26–N51 2.005(6)
Cu1–N1 2.011(7)	Cu10–O23 1.903(5)	Cu18–N35 1.974(6)	Cu26–O13 2.412(5)
Cu2–O2 1.917(5)	Cu10–O10 1.922(5)	Cu19–O19 1.908(5)	Cu27–O26 1.921(5)
Cu2–O1 1.919(5)	Cu10–N46 1.951(7)	Cu19–O18 1.931(5)	Cu27–O27 1.949(5)
Cu2–N2 1.976(7)	Cu10–N19 1.954(7)	Cu19–N36 1.940(6)	Cu27–N53 1.984(6)
Cu2–N3 1.981(6)	Cu11–O11 1.921(5)	Cu19–N37 1.974(7)	Cu27–N52 1.986(6)
Cu3–O2 1.890(5)	Cu11–O10 1.923(5)	Cu20–O19 1.901(5)	Cu28–O28 1.922(5)
Cu3–O3 1.909(5)	Cu11–N21 1.953(6)	Cu20–O20 1.917(5)	Cu28–O27 1.938(5)
Cu3–N4 1.952(6)	Cu11–N20 1.959(7)	Cu20–N39 1.964(7)	Cu28–N54 1.981(6)
Cu3–N5 1.982(6)	Cu12–O11 1.906(5)	Cu20–N38 1.979(7)	Cu28–N55 1.992(6)
Cu4–O4 1.928(5)	Cu12–O12 1.921(5)	Cu21–O21 1.912(5)	Cu29–O29 1.918(5)
Cu4–O3 1.958(5)	Cu12–N22 1.967(6)	Cu21–O20 1.923(5)	Cu29–O28 1.935(5)
Cu4–N7 1.976(7)	Cu12–N23 1.970(6)	Cu21–N40 1.957(7)	Cu29–N57 1.976(7)
Cu4–N6 1.988(6)	Cu13–O13 1.902(5)	Cu21–N41 1.969(7)	Cu29–N56 2.001(6)
Cu4–O14 2.349(5)	Cu13–O12 1.934(5)	Cu22–O22 1.913(5)	Cu30–O29 1.882(5)
Cu5–O4 1.899(6)	Cu13–N24 1.947(6)	Cu22–O21 1.934(6)	Cu30–O30 1.946(5)
Cu5–O5 1.936(5)	Cu13–N25 1.971(7)	Cu22–N42 1.950(7)	Cu30–N58 1.988(6)
Cu5–N9 1.924(7)	Cu14–O13 1.922(5)	Cu22–N43 1.967(7)	Cu30–N59 1.91(2)
Cu5–N8 1.983(7)	Cu14–O14 1.937(5)	Cu23–O23 1.916(5)	Cu30–N59B 2.007(15)
Cu6–O6 1.914(5)	Cu14–N26 1.964(7)	Cu23–O22 1.930(5)	Cu31–O31 1.905(5)
Cu6–O5 1.932(6)	Cu14–N27 1.968(6)	Cu23–N45 1.958(7)	Cu31–O30 1.951(5)
Cu6–N11 1.952(7)	Cu15–O14 1.911(5)	Cu23–N44 1.961(6)	Cu31–N60 1.988(18)
Cu6–N10 1.954(7)	Cu15–O15 1.922(5)	Cu24–O31 1.927(5)	Cu31–N60B 1.968(15)
Cu7–O6 1.936(5)	Cu15–N29 1.949(7)	Cu24–O24 1.952(5)	Cu31–N61 1.927(15)
Cu7–O7 1.996(6)	Cu15–N28 1.971(6)	Cu24–N47 1.985(6)	Cu31–N61B 2.059(15)
Cu7–N12 1.981(7)	Cu16–O15 1.913(5)	Cu24–N62 1.949(16)	
Cu7–N13 1.982(7)	Cu16–O16 1.939(5)	Cu24–N62B 2.066(17)	

Table S22. Selected bond lengths for **5a** (Cu1–Cu9: Cu_9-ring; Cu10–Cu23: Cu_{14}-ring; Cu24–Cu31: Cu_8-ring; O32–O34: FPO_3^{2-} anion).

P1–O32 1.500(8)
P1–O33 1.500(7)
P1–O34 1.503(8)
P1–F1 1.599(7)
Cu1–O9 1.936(8)
Cu1–O1 1.942(7)
Cu1–N1 2.024(9)
Cu1–N18 2.067(16)
Cu1–N18B 1.98(3)
Cu2–O1 1.912(8)
Cu2–O2 1.919(9)
Cu2–N2 1.949(9)
Cu2–N3 1.967(11)
Cu3–O2 1.907(10)
Cu3–O3 1.928(7)
Cu3–N5 1.969(11)
Cu3–N4 1.972(10)
Cu4–O3 1.947(8)
Cu4–O4 1.948(8)
Cu4–N7 1.991(8)
Cu4–N6 2.018(9)
Cu4–O14 2.383(8)
Cu5–O5 1.889(8)
Cu5–O4 1.934(7)
Cu5–N9 1.976(9)
Cu5–N8 1.991(9)
Cu6–O6 1.911(8)
Cu6–O5 1.924(7)
Cu6–N11 1.965(9)
Cu6–N10 1.995(9)
Cu7–O6 1.940(8)
Cu7–O7 1.965(7)
Cu7–N13 1.971(9)
Cu7–N12 1.977(10)
Cu7–O18 2.386(7)
Cu8–O8 1.910(8)
Cu8–O7 1.917(8)
Cu8–N15 1.958(10)
Cu8–N14 1.961(9)
Cu9–O8 1.910(9)
Cu9–O9 1.918(8)
Cu9–N16 1.959(10)
Cu9–N17 1.951(16)
Cu9–N17B 2.07(3)
Cu10–O23 1.922(7)
Cu10–O10 1.934(8)
Cu10–N19 1.955(9)
Cu10–N46 1.964(10)
Cu11–O10 1.913(7)
Cu11–O11 1.935(8)
Cu11–N21 1.941(10)
Cu11–N20 1.992(10)
Cu12–O12 1.917(9)
Cu12–O11 1.934(8)
Cu12–N22 1.980(10)
Cu12–N23 1.966(12)
Cu12–N23B 1.96(3)
Cu13–O12 1.927(9)
Cu13–O13 1.892(8)
Cu13–N24 1.991(12)
Cu13–N24B 1.92(3)
Cu13–N25 1.944(12)
Cu14–O13 1.926(8)
Cu14–O14 1.932(7)
Cu14–N26 1.948(10)
Cu14–N27 1.972(11)
Cu15–O14 1.927(8)
Cu15–O15 1.933(8)
Cu15–N29 1.983(11)
Cu15–N28 1.983(12)
Cu16–O15 1.921(7)
Cu16–O16 1.924(9)
Cu16–N31 1.967(9)
Cu16–N30 1.985(11)
Cu17–O17 1.930(8)
Cu17–O16 1.930(8)
Cu17–N32 1.942(9)
Cu17–N33 1.944(10)
Cu18–O18 1.928(8)
Cu18–O17 1.930(8)
Cu18–N34 1.946(11)
Cu18–N35 1.955(11)
Cu19–O19 1.918(7)
Cu19–O18 1.925(8)
Cu19–N36 1.966(9)
Cu19–N37 2.008(11)
Cu20–O20 1.913(8)
Cu20–O19 1.917(8)
Cu20–N38 1.968(10)
Cu20–N39 1.98(2)
Cu20–N39B 1.91(3)
Cu21–O20 1.910(9)
Cu21–O21 1.927(9)
Cu21–N40 1.994(19)
Cu21–N40B 1.94(3)
Cu21–N41 1.97(3)
Cu21–N41B 1.950(16)
Cu22–O22 1.919(8)
Cu22–O21 1.922(9)
Cu22–N42 2.06(3)
Cu22–N42B 1.936(14)
Cu22–N43 2.027(14)
Cu22–N43B 1.84(3)
Cu23–O23 1.901(8)
Cu23–O22 1.923(8)
Cu23–N44 2.022(14)
Cu23–N44B 1.86(2)
Cu23–N45 1.955(10)
Cu24–O24 1.910(8)
Cu24–O31 1.954(7)
Cu24–N47 1.996(9)
Cu24–N62 2.010(11)
Cu25–O24 1.926(7)
Cu25–O25 1.966(8)
Cu25–N49 1.977(9)
Cu25–N48 2.013(9)
Cu25–O10 2.419(8)
Cu26–O26 1.893(7)
Cu26–O25 1.915(7)
Cu26–N50 1.977(9)
Cu26–N51 1.978(10)
Cu27–O26 1.920(7)
Cu27–O27 1.941(7)
Cu27–N52 1.967(9)
Cu27–N53 1.995(10)
Cu28–O27 1.921(8)
Cu28–O28 1.936(7)
Cu28–N54 1.978(9)
Cu28–N55 2.009(9)
Cu29–O29 1.928(8)
Cu29–O28 1.958(7)
Cu29–N56 1.977(10)
Cu29–N57 2.017(9)
Cu30–O30 1.902(7)
Cu30–O29 1.925(7)
Cu30–N59 1.976(10)
Cu30–N58 2.016(10)
Cu31–O30 1.914(8)
Cu31–O31 1.945(8)
Cu31–N60 1.978(10)
Cu31–N61 1.991(11)

Table S23. Selected bond lengths for **5b** (Cu1–Cu9: Cu_9-ring; Cu10–Cu23: Cu_{14}-ring; Cu24–Cu31: Cu_8-ring; O32–O34: FPO_3^{2-} anion).

P1–O34 1.498(3)	Cu8–O8 1.908(3)	Cu15–N28 1.970(4)	Cu23–N45 1.968(4)
P1–O33 1.499(3)	Cu8–O7 1.933(3)	Cu16–O15 1.926(3)	Cu24–O24 1.917(3)
P1–O32 1.506(3)	Cu8–N15 1.957(4)	Cu16–O16 1.928(3)	Cu24–O31 1.959(3)
P1–F1 1.601(3)	Cu8–N14 1.966(4)	Cu16–N30 1.956(4)	Cu24–N62 1.994(4)
Cu1–O1 1.937(3)	Cu9–O8 1.917(4)	Cu16–N31 1.959(4)	Cu24–N47 1.998(4)
Cu1–O9 1.946(3)	Cu9–O9 1.921(3)	Cu17–O17 1.932(3)	Cu25–O24 1.920(3)
Cu1–N1 2.017(4)	Cu9–N16 1.968(4)	Cu17–O16 1.933(3)	Cu25–O25 1.952(3)
Cu1–N18 2.001(10)	Cu9–N17 1.957(9)	Cu17–N33 1.960(4)	Cu25–N49 1.989(4)
Cu1–N18B 2.03(2)	Cu9–N17B 2.05(2)	Cu17–N32 1.968(4)	Cu25–N48 2.012(4)
Cu2–O2 1.906(4)	Cu10–O23 1.913(3)	Cu18–O18 1.920(3)	Cu25–O10 2.430(3)
Cu2–O1 1.920(3)	Cu10–O10 1.919(3)	Cu18–O17 1.928(3)	Cu26–O26 1.893(3)
Cu2–N3 1.972(4)	Cu10–N19 1.954(4)	Cu18–N35 1.958(4)	Cu26–O25 1.926(3)
Cu2–N2 1.976(4)	Cu10–N46 1.960(4)	Cu18–N34 1.959(4)	Cu26–N50 1.963(4)
Cu3–O2 1.901(4)	Cu11–O10 1.924(3)	Cu19–O19 1.917(3)	Cu26–N51 1.972(4)
Cu3–O3 1.925(3)	Cu11–O11 1.927(3)	Cu19–O18 1.927(3)	Cu27–O26 1.917(3)
Cu3–N4 1.955(4)	Cu11–N21 1.963(4)	Cu19–N36 1.961(4)	Cu27–O27 1.936(3)
Cu3–N5 1.976(4)	Cu11–N20 1.971(4)	Cu19–N37 1.967(4)	Cu27–N52 1.981(4)
Cu4–O4 1.939(3)	Cu12–O11 1.908(3)	Cu20–O19 1.905(3)	Cu27–N53 2.000(4)
Cu4–O3 1.957(3)	Cu12–O12 1.915(4)	Cu20–O20 1.930(3)	Cu28–O27 1.922(3)
Cu4–N7 1.992(4)	Cu12–N22 1.972(5)	Cu20–N38 1.959(5)	Cu28–O28 1.942(3)
Cu4–N6 2.005(4)	Cu12–N23 1.959(18)	Cu20–N39 1.969(16)	Cu28–N54 1.982(4)
Cu4–O14 2.395(3)	Cu12–N23B 1.987(12)	Cu20–N39B 1.943(17)	Cu28–N55 1.998(4)
Cu5–O5 1.917(3)	Cu13–O13 1.913(3)	Cu21–O21 1.911(3)	Cu29–O29 1.919(3)
Cu5–O4 1.917(3)	Cu13–O12 1.919(4)	Cu21–O20 1.915(4)	Cu29–O28 1.950(3)
Cu5–N9 1.971(4)	Cu13–N24 1.933(18)	Cu21–N40 1.982(14)	Cu29–N56 1.980(4)
Cu5–N8 1.984(4)	Cu13–N24B 1.969(14)	Cu21–N40B 1.970(17)	Cu29–N57 2.007(4
Cu6–O6 1.917(3)	Cu13–N25 1.959(5)	Cu21–N41 1.964(5)	Cu30–O30 1.912(3)
Cu6–O5 1.920(3)	Cu14–O13 1.919(3)	Cu22–O22 1.921(3)	Cu30–O29 1.933(3)
Cu6–N10 1.959(4)	Cu14–O14 1.922(3)	Cu22–O21 1.921(3)	Cu30–N59 1.970(4)
Cu6–N11 1.980(4)	Cu14–N27 1.961(4)	Cu22–N42 1.960(4)	Cu30–N58 2.008(4)
Cu7–O6 1.935(3)	Cu14–N26 1.969(4)	Cu22–N43 1.964(4)	Cu31–O30 1.897(3)
Cu7–O7 1.973(3)	Cu15–O14 1.922(3)	Cu23–O23 1.912(3)	Cu31–O31 1.930(3)
Cu7–N12 1.984(4)	Cu15–O15 1.939(3	Cu23–O22 1.927(3)	Cu31–N61 1.978(4)
Cu7–N13 1.987(4)	Cu15–N29 1.962(4)	Cu23–N44 1.953(4)	Cu31–N60 1.991(4)
Cu7–O18 2.373(3)			

Table S24. Selected bond lengths for **5c** (Cu1–Cu14: Cu_{14}-ring; Cu15–Cu23: Cu_9-ring; Cu24–Cu31: Cu_8-ring; O32–O34: FPO_3^{2-} anion).

P7–O32 1.506(5)	Cu8–O7 1.939(5)	Cu16–N30 1.963(6)	Cu24–O31 1.926(5)
P7–O33 1.508(5)	Cu8–N14 1.949(6)	Cu16–N31 1.971(6)	Cu24–O24 1.960(5)
P7–O34 1.509(5)	Cu8–N15 1.983(6)	Cu17–O17 1.948(5)	Cu24–N47 1.984(6)
P7–F7 1.593(5)	Cu9–O9 1.914(5)	Cu17–O16 1.972(5)	Cu24–N62 2.011(6
Cu1–O14 1.923(5)	Cu9–O8 1.930(5)	Cu17–N33 1.989(6)	Cu24–O14 2.436(5)
Cu1–O1 1.932(5)	Cu9–N17 1.969(6)	Cu17–N32 1.992(6)	Cu25–O25 1.900(5)
Cu1–N28 1.957(7)	Cu9–N16 1.976(6)	Cu17–O4 2.361(5)	Cu25–O24 1.938(5)
Cu1–N1 1.969(7)	Cu10–O9 1.901(5)	Cu18–O18 1.909(5)	Cu25–N48 1.972(6)
Cu2–O2 1.910(5)	Cu10–O10 1.936(5)	Cu18–O17 1.911(5)	Cu25–N49 1.978(6)
Cu2–O1 1.919(5)	Cu10–N18 1.979(6)	Cu18–N34 1.954(6)	Cu26–O25 1.912(5)
Cu2–N2 1.951(7)	Cu10–N19 1.980(6)	Cu18–N35 1.979(6)	Cu26–O26 1.945(5)
Cu2–N3 1.957(7)	Cu11–O10 1.902(5)	Cu19–O19 1.912(5	Cu26–N50 1.985(6)
Cu3–O3 1.911(5)	Cu11–O11 1.912(5)	Cu19–O18 1.916(5)	Cu26–N51 2.003(5)
Cu3–O2 1.918(5)	Cu11–N21 1.944(6)	Cu19–N36 1.964(6)	Cu27–O26 1.921(5)
Cu3–N4 1.964(7)	Cu11–N20 1.979(6)	Cu19–N37 1.977(7)	Cu27–O27 1.942(5)
Cu3–N5 1.966(6)	Cu12–O12 1.919(5)	Cu20–O19 1.941(5)	Cu27–N52 1.996(6)
Cu4–O3 1.905(5)	Cu12–O11 1.929(5)	Cu20–O20 1.974(5)	Cu27–N53 2.001(5)
Cu4–O4 1.925(5)	Cu12–N23 1.961(6)	Cu20–N39 1.987(6)	Cu28–O28 1.926(5)
Cu4–N7 1.949(6)	Cu12–N22 1.973(6)	Cu20–N38 2.000(7)	Cu28–O27 1.959(5)
Cu4–N6 1.989(7)	Cu13–O13 1.923(5)	Cu20–O8 2.342(5)	Cu28–N54 1.972(5)
Cu5–O4 1.927(5)	Cu13–O12 1.932(4)	Cu21–O21 1.897(5)	Cu28–N55 1.993(6)
Cu5–O5 1.936(5)	Cu13–N24 1.949(7)	Cu21–O20 1.919(5)	Cu29–O29 1.914(4)
Cu5–N8 1.970(6)	Cu13–N25 1.965(6)	Cu21–N41 1.961(6)	Cu29–O28 1.929(5)
Cu5–N9 1.980(6)	Cu14–O13 1.912(5)	Cu21–N40 1.979(6)	Cu29–N57 1.980(6)
Cu6–O5 1.917(5)	Cu14–O14 1.918(5)	Cu22–O21 1.918(5)	Cu29–N56 2.007(6)
Cu6–O6 1.938(5)	Cu14–N27 1.958(6)	Cu22–O22 1.920(5)	Cu30–O29 1.899(4)
Cu6–N11 1.946(7)	Cu14–N26 1.969(6)	Cu22–N43 1.966(6)	Cu30–O30 1.925(5)
Cu6–N10 1.966(7)	Cu15–O23 1.931(5)	Cu22–N42 1.970(6)	Cu30–N59 1.983(6)
Cu7–O7 1.924(5)	Cu15–O15 1.931(5)	Cu23–O23 1.937(5	Cu30–N58 1.988(6)
Cu7–O6 1.937(5)	Cu15–N29 1.970(7)	Cu23–O22 1.942(5)	Cu31–O31 1.925(5)
Cu7– N12 1.944(6)	Cu15–N46 1.992(6)	Cu23–N45 2.008(6)	Cu31–O30 1.959(5)
Cu7–N13 1.971(6)	Cu16–O15 1.897(6)	Cu23–N44 2.013(6)	Cu31–N61 1.985(6)
Cu8–O8 1.920(5)	Cu16–O16 1.932(5)	Cu23–O13 2.439(5)	Cu31–N60 1.995(6)

Table S25. Hydrogen bonding data for UNIT 1 of **1** (O1_3–O9_3: Cu_9-ring; O10_3–O22_3: Cu_{13}-ring; O23_3–O29_3: Cu_7-ring; O1_1–O4_1: entrapped HPO_4^{2-} anion).

D*—H···*A	***D*—H (Å)**	**H···*A* (Å)**	***D*···*A* (Å)**	***D*—H···*A* (°)**
O2_1—H2A_1···O1_3	0.77(6)	2.08(7)	2.820(4)	160(7)
O1_3—H1O_3···O3_1	0.836(19)	2.51(4)	3.162(4)	136(5)
O2_3—H2O_3···O1_1	0.825(19)	2.11(2)	2.914(4)	166(5)
O3_3—H3O_3···O1_1	0.818(19)	1.96(2)	2.779(4)	175(5)
O4_3—H4O_3···O1_1	0.82(2)	2.27(3)	3.038(4)	155(5)
O6_3—H6O_3···O4_1	0.83(2)	1.88(2)	2.697(4)	170(6)
O7_3—H7O_3···O4_1	0.82(2)	2.20(3)	2.987(4)	160(6)
O8_3—H8O_3···O3_1	0.82(2)	2.28(2)	3.087(4)	166(5)
O9_3—H9O_3···O3_1	0.834(19)	1.94(2)	2.764(4)	170(5)
O10_3—H10O_3···O23_3	0.836(19)	1.96(2)	2.787(4)	172(5)
O11_3—H11O_3···O2_3	0.832(19)	2.05(2)	2.868(4)	168(5)
O12_3—H12O_3···O24_3	0.804(19)	2.09(2)	2.893(4)	174(5)
O13_3—H13O_3···O3_3	0.826(19)	1.95(2)	2.773(4)	176(5)
O14_3—H14O_3···O25_3	0.839(19)	2.10(2)	2.923(4)	165(5)
O15_3—H15O_3···O4_3	0.84(2)	2.14(2)	2.952(4)	165(6)
O16_3—H16O_3···O26_3	0.85(2)	2.01(2)	2.844(4)	169(6)
O17_3—H17O_3···O6_3	0.84(2)	1.91(2)	2.745(4)	169(6)
O18_3—H18O_3···O27_3	0.83(2)	1.88(3)	2.689(4)	163(6)
O19_3—H19O_3···O7_3	0.82(2)	2.28(3)	3.054(5)	156(5)
O20_3—H20O_3···O8_3	0.83(2)	2.07(3)	2.871(4)	162(5)
O21_3—H21O_3···O29_3	0.828(19)	1.87(2)	2.689(4)	172(6)
O22_3—H22O_3···O9_3	0.827(19)	1.90(2)	2.717(4)	167(5)
O23_3—H23O_3···O3_1	0.820(19)	2.05(3)	2.796(4)	151(5)
O24_3—H24O_3···O1_1	0.819(19)	2.09(2)	2.889(4)	165(5)
O25_3—H25O_3···O1_1	0.827(19)	2.07(2)	2.893(4)	170(5)
O26_3—H26O_3···O4_1	0.831(19)	2.06(3)	2.852(4)	160(5)
O27_3—H27O_3···O4_1	0.84(2)	1.95(2)	2.763(4)	162(5)
O28_3—H28O_3···O3_1	0.824(19)	2.55(3)	3.288(4)	150(5)
O29_3—H29O_3···O3_1	0.824(19)	1.93(2)	2.723(4)	162(5)

Table S26. Hydrogen bonding data for UNIT 2 of **1** (O1_4–O9_4: Cu_9-ring; O10_4–O22_4: Cu_{13}-rins; O23_4–O29_4: Cu_7-ring; O1_2–O4_2: entrapped HPO_4^{2-} anion).

D*—H···*A	***D*—H (Å)**	**H···*A* (Å)**	***D*···*A* (Å)**	***D*—H···*A* (°)**
O2_2—H2A_2···O8_4	0.71(6)	2.13	2.803(4)	158.7
O1_4—H1O_4···O1_2	0.843(19)	2.25(3)	3.072(4)	163(4)
O2_4—H2O_4···O4_2	0.84(2)	2.18(3)	2.986(4)	162(6)
O3_4—H3O_4···O4_2	0.83(2)	1.89(2)	2.698(4)	167(6)
O4_4—H4O_4···O4_2	0.84(2)	2.58(3)	3.359(4)	156(5)
O5_4—H5O_4···O3_2	0.82(2)	2.30(3)	3.075(4)	157(5)
O6_4—H6O_4···O3_2	0.823(19)	1.98(2)	2.782(4)	166(5)
O7_4—H7O_4···O3_2	0.828(19)	2.09(2)	2.913(4)	170(5)
O8_4—H8O_4···O1_2	0.823(19)	2.57(4)	3.211(4)	135(5)
O9_4—H9O_4···O1_2	0.828(19)	1.95(2)	2.775(4)	173(5)
O10_4—H10O_4···O1_4	0.84(2)	2.03(2)	2.858(4)	170(5)
O11_4—H11O_4···O2_4	0.84(2)	2.36(3)	3.140(5)	155(5)
O12_4—H12O_4···O24_4	0.83(2)	1.86(2)	2.685(4)	174(6)
O13_4—H13O_4···O3_4	0.847(19)	1.90(2)	2.740(4)	170(5)
O14_4—H14O_4···O25_4	0.83(2)	2.03(2)	2.845(4)	166(6)
O15_4—H15O_4···O5_4	0.84(2)	2.13(2)	2.949(4)	165(5)
O16_4—H16O_4···O26_4	0.82(2)	2.10(2)	2.908(4)	166(5)
O17_4—H17O_4···O6_4	0.831(19)	1.93(2)	2.765(4)	178(5)
O18_4—H18O_4···O27_4	0.824(19)	2.07(2)	2.890(3)	170(5)
O19_4—H19O_4···O7_4	0.833(19)	2.07(2)	2.883(4)	165(5)
O20_4—H20O_4···O28_4	0.83(2)	1.96(2)	2.788(4)	174(5)
O21_4—H21O_4···O9_4	0.827 (19)	1.91(2)	2.733(4)	172(5)
O22_4—H22O_4···O29_4	0.831(19)	1.86(2)	2.687(4)	176(5)
O23_4—H23O_4···O1_2	0.832(19)	2.49(3)	3.251(4)	152(5)
O24_4—H24O_4···O4_2	0.823(19)	1.97(2)	2.793(4)	174(5)
O25_4—H25O_4···O4_2	0.836(19)	2.05(2)	2.856(4)	163(5)
O26_4—H26O_4···O3_2	0.831(19)	2.10(3)	2.894(4)	159(5)
O27_4—H27O_4···O3_2	0.822(19)	2.05(2)	2.864(4)	171(5)
O28_4—H28O_4···O1_2	0.823(19)	2.00(2)	2.808(4)	166(5)
O29_4—H29O_4···O1_2	0.834(19)	1.91(2)	2.724(4)	163(5)

Table S27. Hydrogen bonding data for **2** (O1_2–O8_2 and O1_2^{i}–O8_2^{i}: Cu_8-rings; O9_2–O15_2 and O9_2^{i}–O14_2^{i}: Cu_{13}-ring; O1_1–O4_1 and O1_1^{i}–O4_1^{i}: incarcerated HPO_4^{2-} anion disordered over two positions around a C_2 axis). Symmetry code: (i) $-x+1, y, -z+1/2$.

D*—H···*A	***D*—H (Å)**	**H···*A* (Å)**	***D*···*A* (Å)**	***D*—H···*A* (°)**
O1_2—H1O_2···O1_1	0.806(19)	2.57(3)	3.289(7)	150(4)
O1_2—H1O_2···O2_1^{i}	0.806(19)	1.90(2)	2.705(5)	174(4)
O2_2—H2O_2···O1_1	0.83(3)	2.62(8)	2.589(7)	78(5)
O3_2—H3O_2···O1_1	0.804(19)	2.15(3)	2.883(6)	152(4)
O3_2—H3O_2···O3_1^{i}	0.804(19)	2.10(3)	2.853(5)	157(4)
O4_2—H4O_2···O3_1	0.84(2)	2.00(10)	2.707(7)	140(9)
O4B_2—H4BO_2···O3_1	0.84(2)	2.20(9)	2.956(7)	149(10)
O4_2^{i}—H4O_2^{i}···O4_1	0.84(2)	2.91(11)	3.019(8)	89(7)
O5_2—H5O_2···O3_1	0.798(15)	1.991(15)	2.788(5)	177(6)
O6_2—H6O_2···O1_1^{i}	0.813(19)	1.99(3)	2.739(6)	153(4)
O6_2—H6O_2···O3_1	0.813(19)	2.50(2)	3.272(6)	159(4)
O7_2—H7O_2···O1_1^{i}	0.828(19)	2.21(3)	2.976(6)	154(4)
O7_2—H7O_2···O2_1	0.828(19)	2.10(3)	2.877(5)	156(4)
O8_2—H8O_2···O2_1	0.821(19)	2.06(2)	2.840(5)	158(4)
O8_2—H8O_2···O2_1^{i}	0.821(19)	2.14(2)	2.945(5)	166(4)
O4_1—H4_1···O7_2^{i}	0.85(2)	2.11(2)	2.823(6)	141(4)
O9_2—H9O_2···O8_2^{i}	0.830(19)	1.92(2)	2.747(3)	172(4)
O10_2—H10O_2···O1_2	0.842(19)	1.96(2)	2.802(3)	178(5)
O11_2—H11O_2···O2_2	0.817(19)	2.26(3)	3.039(4)	159(5)
O12_2—H12O_2···O6_2^{i}	0.821(19)	1.96(2)	2.764(3)	167(4)
O13_2—H13O_2···O3_2	0.812(19)	1.97(2)	2.779(3)	171(4)
O14_2—H14O_2···O5_2^{i}	0.814(19)	2.13(3)	2.891(4)	156(4)
O15_2—H15O_2···O4B_2	0.84(11)	2.2(2)	2.932(6)	151(31)
O5_1—H5B_1···O7_2	0.84(2)	2.16(2)	2.975(11)	164(10)
O5_1—H5A_1···O4_2	0.84(2)	2.46(2)	3.248(12)	156(11)

Table S28. Hydrogen bonding data for **3** (O1–O8: Cu_8-ring; O9–O21: Cu_{13}-ring; O22–O29: Cu_8-ring; O30–O33 and O30b–O33b: HPO_3^{2-} anion disordered over two positions with 0.63/0.37 occupancy).

D*—H⋯*A	***D*—H (Å)**	**H⋯*A* (Å)**	***D*⋯*A* (Å)**	***D*—H⋯*A* (°)**
O2—H2O⋯O30	0.82(2)	1.99(4)	2.784(12)	163(5)
O2—H2O⋯O30B	0.82(2)	2.26(5)	3.050(18)	160(5)
O3—H3O⋯O30	0.82(2)	2.11(6)	2.92(2)	170(5)
O3—H3O⋯O30B	0.82(2)	2.02(6)	2.83(3)	166(4)
O4—H4O⋯O32	0.84(2)	2.19(6)	2.988(18)	158(6)
O5—H5O⋯O32	0.82(2)	1.86(5)	2.685(14)	179(5)
O5—H5O⋯O32B	0.82(2)	2.00(6)	2.81(3)	167(5)
O6—H6O⋯O32	0.82(2)	2.36(5)	3.175(14)	176(4)
O6—H6O⋯O32B	0.82(2)	2.13(5)	2.94(2)	173(6)
O7—H7O⋯O31	0.83(2)	2.15(4)	2.938(12)	160(5)
O7—H7O⋯O31B	0.83(2)	2.22(5)	2.97(2)	152(5)
O8—H8O⋯O31	0.83(2)	2.02(4)	2.847(14)	171(5)
O8—H8O⋯O31B	0.83(2)	1.76(4)	2.59(3)	174(5)
O9—H9O⋯O1	0.82(2)	2.26(4)	3.042(4)	159(5)
O10—H10O⋯O2	0.82(2)	2.13(5)	2.923(4)	163(5)
O11—H11O⋯O24	0.84(2)	1.97(4)	2.792(4)	169(4)
O12—H12O⋯O3	0.83(2)	1.99(4)	2.806(5)	169(5)
O13—H13O⋯O25	0.83(2)	2.03(5)	2.801(5)	153(4)
O15—H15O⋯O5	0.83(2)	1.95(4)	2.770(4)	172(6)
O16—H16O⋯O27	0.84(2)	1.90(4)	2.734(4)	174(4)
O17—H17O⋯O6	0.83(2)	1.94(4)	2.768(4)	175(6)
O18—H18O⋯O28	0.84(2)	2.01(4)	2.840(4)	174(5)
O19—H19O⋯O29	0.84(2)	2.15(4)	2.923(5)	153(5)
O20—H20O⋯O8	0.84(2)	1.92(4)	2.757(5)	174(5)
O21—H21O⋯O22	0.83(2)	1.93(4)	2.755(5)	173(5)
O22—H22O⋯O31	0.83(2)	2.25(5)	3.034(15)	157(5)
O22—H22O⋯O31B	0.83(2)	2.05(5)	2.84(3)	155(5)
O23—H23O⋯O30	0.84(2)	2.12(5)	2.937(15)	163(6)
O23—H23O⋯O30B	0.84(2)	2.23(5)	3.01(2)	156(5)
O24—H24O⋯O30	0.81(2)	2.12(5)	2.890(14)	157(6)
O24—H24O⋯O30B	0.81(2)	1.94(5)	2.72(2)	160(5)
O25—H25O⋯O30B	0.83(2)	2.35(5)	3.07(3)	146(5)
O26—H26O⋯O32	0.82(2)	1.99(3)	2.799(16)	170(5)
O26—H26O⋯O32B	0.82(2)	2.13(4)	2.91(3)	159(5)
O27—H27O⋯O32	0.82(2)	2.21(5)	3.006(11)	152(5)
O27—H27O⋯O32B	0.82(2)	2.01(4)	2.784(17)	159(5)
O28—H28O⋯O31	0.83(2)	2.45(5)	3.174(14)	146(5)
O29—H29O⋯O31	0.83(2)	1.90(5)	2.700(17)	164(6)
O29—H29O⋯O31B	0.83(2)	2.07(6)	2.83(3)	153(6)

Table S29. Hydrogen bonding data for **4a** (O1–O9: Cu_9-ring; O10–O23: Cu_{14}-ring; O24–O31: Cu_8-ring; O32–O34 and O32B–O34B: HPO_3^{2-} anion disordered over two positions with 0.85/0.15 occupancy).

D*—H···*A	***D*—H (Å)**	**H···*A* (Å)**	***D*···*A* (Å)**	***D*—H···*A* (°)**
O1—H1O···O32	0.840(2)	2.61(2)	3.242(12)	133(3)
O1—H1O···O32B	0.840(2)	2.24(6)	2.90(5)	135(3)
O2—H2O···O33	0.840(2)	2.09(3)	2.914(11)	165(11)
O2—H2O···O33B	0.840(2)	2.36(7)	3.16(6)	161(10)
O3—H3O···O33	0.840(2)	2.20(2)	2.928(12)	145(3)
O3—H3O···O33B	0.840(2)	2.31(8)	2.98(6)	137(4)
O5—H5O···O34	0.840(2)	1.998(13)	2.834(9)	174(9)
O5—H5O···O34B	0.840(2)	2.18(5)	3.00(4)	167(9)
O6—H6O···O34	0.840(2)	2.038(18)	2.870(9)	171(10)
O6—H6O···O34B	0.840(2)	1.94(3)	2.78(3)	178(10)
O7—H7O···O34B	0.840(2)	2.48(8)	3.16(6)	139(7)
O8—H8O···O32	0.840(2)	2.07(3)	2.876(11)	161(10)
O8—H8O···O32B	0.840(2)	2.25(7)	3.03(5)	154(10)
O9—H9O···O32	0.840(2)	2.09(5)	2.857(9)	152(9)
O9—H9O···O32B	0.840(2)	1.89(4)	2.70(2)	161(10)
O11—H11O···O25	0.840(2)	2.07(2)	2.893(9)	168(10)
O12—H12O···O25	0.840(2)	2.07(6)	2.881(8)	163(9)
O13—H13O···O26	0.840(2)	1.99(3)	2.795(8)	161(10)
O14—H14O···O3	0.840(2)	1.994(13)	2.832(9)	175(11)
O15—H15O···O27	0.840(2)	2.07(4)	2.855(8)	156(9)
O17—H17O···O5	0.840(2)	1.918(9)	2.756(7)	175(7)
O18—H18O···O29	0.840(2)	1.94(3)	2.755(8)	164(9)
O19—H19O···O6	0.840(2)	2.03(5)	2.795(8)	152(9)
O20—H20O···O30	0.840(2)	2.093(19)	2.822(8)	145(3)
O21—H21O···O8	0.840(2)	1.991(18)	2.790(8)	159(5)
O22—H22O···O31	0.840(2)	2.08(6)	2.765(7)	139(9)
O23—H23O···O9	0.840(2)	2.03(3)	2.831(8)	161(8)
O24—H24O···O32	0.840(2)	2.19(3)	3.005(15)	165(10)
O24—H24O···O32B	0.840(2)	2.16(9)	2.97(8)	161(10)
O25—H25O···O33	0.840(2)	2.40(5)	3.163(14)	152(9)
O25—H25O···O33B	0.840(2)	2.43(8)	3.21(7)	154(9)
O26—H26O···O33	0.840(2)	2.05(3)	2.841(11)	156(8)
O26—H26O···O33B	0.840(2)	1.97(5)	2.74(4)	152(8)
O27—H27O···O33	0.840(2)	2.30(6)	3.038(10)	147(10)
O27—H27O···O33B	0.840(2)	1.99(8)	2.75(5)	151(10)
O28—H28O···O34	0.840(2)	2.17(3)	2.974(10)	160(8)
O28—H28O···O34B	0.840(2)	2.48(7)	3.29(7)	162(8)
O29—H29O···O34	0.840(2)	2.00(2)	2.820(11)	166(8)
O29—H29O···O34B	0.840(2)	2.15(8)	2.99(8)	173(8)
O31—H31O···O32	0.840(2)	2.16(5)	2.900(12)	147(9)
O31—H31O···O32B	0.840(2)	2.44(8)	3.14(6)	141(9)
O35—H35A···O1	0.840(2)	2.15(3)	2.977(12)	168(11)
O35—H35B···O7	0.840(2)	2.05(3)	2.868(11)	165(9)

Table S30. Hydrogen bonding data for **4b** (O1–O9: Cu_9-ring; O10–O23: Cu_{14}-ring; O24–O31: Cu_8-ring; O32–O34 and O32B–O34B: HPO_3^{2-} anion disordered over two positions with 0.75/0.25 occupancy).

D*—H···*A	***D*—H (Å)**	**H···*A* (Å)**	***D*···*A* (Å)**	***D*—H···*A* (°)**
O1—H1O···O32	0.840(2)	2.40(2)	3.184(13)	155(5)
O1—H1O···O32B	0.840(2)	2.14(4)	2.94(4)	160(5)
O2—H2O···O33	0.840(2)	2.12(2)	2.935(12)	163(5)
O2—H2O···O33B	0.840(2)	2.14(4)	2.95(4)	164(5)
O3—H3O···O33	0.840(2)	2.199(16)	3.010(7)	162(5)
O3—H3O···O33B	0.840(2)	2.00(2)	2.83(2)	167(5)
O4—H4O···O33B	0.840(2)	2.54(4)	3.33(3)	157(5)
O5—H5O···O34	0.840(2)	2.011(11)	2.844(7)	172(5)
O5—H5O···O34B	0.840(2)	2.24(2)	3.06(2)	169(5)
O6—H6O···O34	0.840(2)	2.040(10)	2.876(7)	173(5)
O6—H6O···O34B	0.840(2)	1.97(2)	2.80(2)	172(5)
O7—H7O···O34B	0.840(2)	2.49(4)	3.20(3)	144(5)
O8—H8O···O32	0.840(2)	2.052(18)	2.873(10)	166(5)
O8—H8O···O32B	0.840(2)	2.27(3)	3.08(3)	162(5)
O9—H9O···O32	0.840(2)	2.09(2)	2.884(7)	157(5)
O9—H9O···O32B	0.840(2)	1.97(2)	2.77 (15)	162(5)
O11—H11O···O25	0.840(2)	2.054(9)	2.889(4)	172(5)
O12—H12O···O2	0.840(2)	2.10(4)	2.932(4)	169(4)
O13—H13O···O26	0.840(2)	1.958(8)	2.794(4)	174(5)
O14—H14O···O3	0.840(2)	1.976(5)	2.815(4)	179(5)
O15—H15O···O27	0.840(2)	2.027(11)	2.853(4)	168(5)
O17—H17O···O5	0.840(2)	1.924(10)	2.753(4)	169(5)
O18—H18O···O29	0.840(2)	1.924(13)	2.748(4)	167(5)
O19—H19O···O6	0.840(2)	1.953(16)	2.768(4)	163(5)
O20—H20O···O30	0.840(2)	2.020(9)	2.854(4)	172(5)
O21—H21O···O8	0.840(2)	2.002(15)	2.816(4)	163(5)
O22—H22O···O31	0.840(2)	1.935(4)	2.775(4)	179(6)
O23—H23O···O9	0.840(2)	1.969(5)	2.809(4)	178(5)
O24—H24O···O32	0.840(2)	2.208(19)	3.037(16)	169(5)
O24—H24O···O32B	0.840(2)	2.07(5)	2.91(5)	172(5)
O25—H25O···O33	0.840(2)	2.37(2)	3.173(8)	159(5)
O26—H26O···O33	0.840(2)	1.998(13)	2.827(8)	169(5)
O26—H26O···O33B	0.840(2)	2.11(3)	2.94(3)	173(5)
O27—H27O···O33	0.840(2)	2.153(15)	2.987(13)	172(5)
O27—H27O···O33B	0.840(2)	2.09(4)	2.92(4)	174(5)
O28—H28O···O34	0.840(2)	2.157(15)	2.984(10)	168(5)
O28—H28O···O34B	0.840(2)	2.41(4)	3.24(3)	168(5)
O29—H29O···O34	0.840(2)	2.010(15)	2.833(10)	166(5)
O29—H29O···O34B	0.840(2)	2.04(4)	2.88(3)	172(5)
O30—H30O···O34B	0.840(2)	2.44(4)	3.21(3)	154(4)
O31—H31O···O32	0.840(2)	2.120(17)	2.946(13)	168(5)
O31—H31O···O32B	0.840(2)	2.31(4)	3.11(4)	160(5)
O35—H35A···O1	0.840(2)	2.147(19)	2.965(6)	164(7)
O35—H35B···O7	0.840(2)	2.08(2)	2.890(5)	162(7)

Table S31. Hydrogen bonding data for **4c** (O1–O9: Cu_9-ring; O10–O23: Cu_{14}-ring; O24–O31: Cu_8-ring; O32–O34 and O32B–O34B: HPO_3^{2-} anion disordered over two positions with 0.77/0.23 occupancy).

D*—H···*A	***D*—H (Å)**	**H···*A* (Å)**	***D*···*A* (Å)**	***D*—H···*A* (°)**
O1—H1O···O33	0.84(2)	2.07(3)	2.899(11)	173(9)
O1—H1O···O33B	0.84(2)	2.23(4)	3.05(3)	167(9)
O2—H2O···O34B	0.83(2)	2.50(7)	3.21(3)	144(9)
O3—H3O···O34	0.84(2)	2.13(5)	2.894(9)	151(9)
O3—H3O···O34B	0.84(2)	1.99(5)	2.761(19)	152(9)
O4—H4O···O34	0.83(2)	2.16(7)	2.840(11)	139(9)
O4—H4O···O34B	0.83(2)	2.21(7)	2.94(3)	146(9)
O5—H5O···O32	0.880(17)	2.658(17)	3.529(12)	170(8)
O6—H6O···O32	0.84(2)	2.08(3)	2.896(11)	166(10)
O6—H6O···O32B	0.84(2)	2.06(4)	2.88(3)	166(9)
O7—H7O···O32	0.84(2)	2.03(5)	2.811(9)	155(9)
O7—H7O···O32B	0.84(2)	2.15(5)	2.95(2)	158(9)
O9—H9O···O33	0.83(2)	2.22(5)	2.968(13)	149(9)
O9—H9O···O33B	0.83(2)	2.03(6)	2.81(4)	156(9)
O10—H10O···O1	0.83(2)	2.02(3)	2.842(8)	172(9)
O11—H11O···O24	0.83(2)	2.13(6)	2.849(7)	144(9)
O13—H13O···O3	0.83(2)	1.98(10)	2.734(8)	149(8)
O14—H14O···O26	0.83(2)	2.00(5)	2.759(7)	153(9)
O15—H15O···O4	0.84(2)	1.95(3)	2.768(7)	165(9)
O16—H16O···O27	0.83(2)	2.08(5)	2.839(7)	152(9)
O17—H17O···O6	0.83(2)	2.03(7)	2.801(9)	151(9)
O18—H18O···O28	0.83(2)	1.95(2)	2.778(7)	173(9)
O19—H19O···O7	0.84(2)	2.02(5)	2.800(7)	154(9)
O20—H20O···O7	0.84(2)	2.25(3)	3.080(8)	171(10)
O21—H21O···O30	0.84(2)	2.12(5)	2.893(7)	153(10)
O22—H22O···O9	0.84(2)	2.20(6)	2.909(8)	143(9)
O23—H23O···O31	0.84(2)	1.96(3)	2.792(7)	170(10)
O24—H24O···O33	0.84(2)	2.13(3)	2.953(13)	167(9)
O24—H24O···O33B	0.84(2)	2.36(5)	3.18(4)	166(9)
O25—H25O···O34	0.83(2)	2.20(3)	3.014(15)	166(9)
O25—H25O···O34B	0.83(2)	2.17(6)	2.99(5)	167(9)
O26—H26O···O34	0.83(2)	1.97(3)	2.789(11)	167(9)
O26—H26O···O34B	0.83(2)	2.19(5)	3.01(4)	165(9)
O27—H27O···O32B	0.84(2)	2.56(4)	3.38(3)	170(6)
O28—H28O···O32	0.84(2)	2.18(3)	3.007(10)	169(9)
O28—H28O···O32B	0.84(2)	1.89(4)	2.72(3)	171(9)
O29—H29O···O32	0.84(2)	2.23(4)	3.038(12)	161(9)
O29—H29O···O32B	0.84(2)	2.10(5)	2.92(4)	166(10)
O30—H30O···O33	0.83(2)	2.48(5)	3.241(11)	152(9)
O30—H30O···O33B	0.83(2)	2.17(6)	2.94(3)	154(9)
O31—H31O···O33	0.84(2)	2.03(3)	2.851(11)	169(9)
O31—H31O···O33B	0.84(2)	1.99(5)	2.79(3)	160(9)
O35—H35A···O8	0.84(2)	2.19(6)	2.979(10)	156(13)
O35—H35B···O5	0.84(2)	2.13(8)	2.884(10)	149(15)

Table S32. Hydrogen bonding data for **5a** (O1–O9: Cu_9-ring; O10–O23: Cu_{14}-ring; O24–O31: Cu_8-ring; O32–O34: FPO_3^{2-} anion).

D*—H···*A	***D*—H (Å)**	**H···*A* (Å)**	***D*···*A* (Å)**	***D*—H···*A* (°)**
O1—H1O···O32	0.863(18)	2.125(17)	2.980(10)	170(10)
O2—H2O···O34	0.85(2)	2.57(2)	3.378(11)	159(7)
O3—H3O···O34	0.838(17)	2.002(17)	2.833(10)	171(11)
O4—H4O···O34	0.86(2)	2.21(2)	3.006(12)	154(6)
O5—H5O···F1	0.857(19)	2.422(19)	3.239(10)	160(7)
O6—H6O···O33	0.84(2)	2.23(7)	2.950(10)	144(10)
O7—H7O···O33	0.83(2)	2.19(10)	2.859(11)	137(8)
O8—H8O···F1	0.856(19)	2.421(19)	3.254(12)	164(9)
O9—H9O···O32	0.84(2)	2.38(10)	3.014(11)	133(12)
O10—H10O···O1	0.85(2)	2.11(2)	2.880(11)	150(5)
O11—H11O···O25	0.83(2)	2.094(19)	2.884(10)	158(7)
O12—H12O···O26	0.85(2)	2.412(19)	3.242(12)	164(8)
O13—H13O···O3	0.84(2)	2.01(2)	2.793(10)	155(6)
O14—H14O···O27	0.832(19)	1.989(19)	2.798(11)	164(9)
O15—H15O···O4	0.836(19)	1.992(19)	2.785(11)	158(7)
O16—H16O···O28	0.84(2)	2.05(5)	2.863(11)	163(13)
O17—H17O···O6	0.846(18)	2.007(18)	2.834(11)	166(9)
O18—H18O···O29	0.84(2)	1.94(2)	2.780(10)	177(13)
O19—H19O···O7	0.845(19)	2.014(19)	2.826(10)	161(7)
O20—H20O···O7	0.841(18)	2.311(18)	3.140(11)	169(10)
O21—H21O···O8	0.863(18)	2.332(18)	3.182(14)	168(10)
O22—H22O···O9	0.84(2)	2.10(6)	2.890(12)	157(13)
O23—H23O···O24	0.852(19)	2.015(19)	2.832(11)	160(7)
O24—H24O···O32	0.84(2)	2.10(5)	2.902(10)	159(12)
O25—H25O···O32	0.84(2)	2.37(8)	3.085(10)	143(11)
O26—H26O···O34	0.85(2)	2.255(14)	2.982(11)	144(4)
O27—H27O···O34	0.84(2)	2.07(7)	2.837(11)	152(12)
O29—H29O···O33	0.84(2)	2.10(2)	2.833(10)	146(5)
O30—H30O···O33	0.85(2)	2.20(2)	2.977(10)	152(5)
O31—H31O···O32	0.84 (2)	2.37(7)	3.133 (11)	152(12)

Table S33. Hydrogen bonding data for **5b** (O1–O9: Cu_9-ring; O10–O23: Cu_{14}-ring; O24–O31: Cu_8-ring; O32–O34: FPO_3^{2-} anion).

D*—H···*A	***D*—H (Å)**	**H···*A* (Å)**	***D*···*A* (Å)**	***D*—H···*A* (°)**
O1—H1O···O32	0.82(2)	2.16(4)	2.960(5)	163(6)
O3—H3O···O34	0.80(2)	2.03(4)	2.825(4)	175(5)
O4—H4O···O34	0.81(2)	2.24(3)	3.023(5)	164(5)
O5—H5O···F1	0.82(2)	2.62(7)	3.236(5)	133(6)
O6—H6O···O33	0.82(2)	2.14(6)	2.938(5)	165(6)
O7—H7O···O33	0.82(2)	2.04(6)	2.831(5)	164(7)
O8—H8O···F1	0.81(2)	2.44(5)	3.232(5)	166(6)
O9—H9O···O32	0.81(2)	2.19(6)	2.984(6)	171(5)
O10—H10O···O1	0.81(2)	2.12(5)	2.904(5)	165(7)
O11—H11O···O25	0.83(2)	2.07(2)	2.904(4)	178(6)
O13—H13O···O3	0.81(2)	2.01(5)	2.785(5)	162(6)
O14—H14O···O27	0.83(2)	1.97(2)	2.799(4)	176(4)
O15—H15O···O4	0.82(2)	1.99(7)	2.797(6)	170(5)
O16—H16O···O28	0.83(2)	2.01(4)	2.841(5)	178(5)
O17—H17O···O6	0.82(2)	2.02(7)	2.816(6)	167(5)
O18—H18O···O29	0.82(2)	2.00(5)	2.781(5)	161(4)
O19—H19O···O7	0.84(2)	1.99(4)	2.828(4)	170(5)
O21—H21O···O31	0.83(2)	2.12(5)	2.884(6)	155(6)
O22—H22O···O9	0.82(2)	2.06(3)	2.868(5)	170(6)
O23—H23O···O24	0.80(2)	2.05(6)	2.813(5)	157(5)
O24—H24O···O32	0.80(2)	2.11(5)	2.905(5)	169(5)
O25—H25O···O32	0.81(2)	2.31(5)	3.089(5)	161(4)
O26—H26O···O34	0.81(2)	2.22(4)	2.983(5)	158(7)
O27—H28O···O34	0.81(2)	2.03(7)	2.832(6)	168(6)
O29—H29O···O33	0.82(2)	2.05(4)	2.846(5)	168(7)
O30—H30O···O33	0.80(2)	2.24(4)	2.994(4)	157(5)
O31—H31O···O32	0.82(2)	2.35(3)	3.126(4)	158(6)

Table S34. Hydrogen bonding data for **5c** (O1–O14: Cu_{14}-ring; O15–O23: Cu_9-ring; O24–O31: Cu_8-ring; O32–O34: FPO_3^{2-} anion).

D*—H···*A	***D*—H (Å)**	**H···*A* (Å)**	***D*···*A* (Å)**	***D*—H···*A* (°)**
O1—H1O···O24	0.84(2)	2.05(3)	2.883(7)	167(9)
O2—H2O···O16	0.84(2)	2.47(7)	3.142(7)	137(8)
O3—H3O···O16	0.85(2)	2.01(4)	2.813(7)	158(9)
O4—H4O···O26	0.83(2)	1.99(3)	2.817(7)	170(9)
O5—H5O···O17	0.84(2)	2.01(3)	2.834(7)	167(9)
O6—H6O···O27	0.826(18)	2.087(18)	2.874(7)	159(6)
O7—H7O···O19	0.83(2)	1.99(2)	2.818(7)	176(9)
O8—H8O···O28	0.84(2)	1.95(2)	2.784(7)	175(9)
O9—H9O···O20	0.84(2)	1.96(3)	2.788(7)	169(9)
O10—H10O···O29	0.868(19)	2.336(19)	3.132(7)	152(5)
O11—H11O···O30	0.814(15)	2.081(15)	2.892(7)	174(9)
O12—H12O···O22	0.84(2)	2.05(3)	2.877(7)	169(9)
O13—H13O···O31	0.83(2)	1.98(2)	2.811(6)	174(9)
O14—H14O···O23	0.83(2)	2.04(3)	2.865(7)	170(9)
O15—H15O···F7	0.893(17)	2.59(7)	3.254(7)	131(7)
O15—H15O···O34	0.893(17)	2.557(16)	3.431(7)	166(6)
O16—H16O···O34	0.84(2)	2.04(3)	2.855(7)	162(8)
O17—H17O···O34	0.84(2)	2.14(3)	2.934(7)	158(7)
O18—H18O···F7	0.84(2)	2.35(5)	3.084(6)	146(7)
O19—H19O···O33	0.84(2)	2.18 (4)	2.977(7)	157(9)
O20—H20O···O33	0.84(2)	2.022)	2.855(7)	172(9)
O21—H21O···O33	0.84(2)	2.62(5)	3.386(7)	153(9)
O22—H22O···O32	0.837(18)	2.159(18)	2.957(7)	159(6)
O23—H23O···O32	0.83(2)	2.17(3)	2.979(7)	163(7)
O24—H24O···O32	0.84(2)	2.34(2)	3.166(7)	170(6)
O25—H25O···O34	0.84(2)	2.23(4)	2.993(7)	152(7)
O26—H26O···O34	0.84(2)	2.01(2)	2.841(7)	173(7)
O28—H28O···O33	0.84(2)	1.98(2)	2.819(7)	178(9)
O29—H29O···O33	0.83(2)	2.20(5)	2.963(7)	152(8)
O30—H30O···O32	0.84(2)	2.25(3)	3.077(7)	167(8)
O31—H31O···O32	0.842(17)	2.068(18)	2.878(7)	161(6)

4. NMR SPECTROSCOPIC DATA

Table S35. Variable-temperature ^{1}H NMR chemical shifts (ppm) of **Cu_nHPO_4** (in magenta; second line in the table for **Cu_{31}** is for the pure **$Cu_{31}HPO_4$** sample), **Cu_nHPO_3** (in blue) and **Cu_nFPO_3** (in green) nanojar mixtures, compared to the corresponding values of **Cu_nCO_3** (in orange) in DMSO-d_6. Missing values are due to lack of significant amounts of a particular species in the sample and/or to inability to unambiguously assign peaks due to overlap or excessive broadening.

NANOJAR	20 °C	30 °C	40 °C	50 °C	60 °C	70 °C	80 °C	90 °C	100 °C	110 °C	120 °C	130 °C	140 °C	150 °C
**Cu_{27} (6+12+9)**														
Cu_9-ring, pz-4-*H*	–	–	–	–	–	–	–	–	–	–	–	–	–	–
	33.89	33.75	33.45	33.07	32.83	32.50	32.17	31.85	31.53	31.22	30.89	30.58	–	–
	33.97	33.84	33.56	33.26	32.95	32.63	32.30	31.95	31.63	31.29	30.99	–	–	–
	37.63	37.23	36.75	36.29	35.84	35.41	35.00	34.59	34.20	33.82	33.44	33.09	32.72	32.39
Cu_9-ring, pz-3,5-H_2	–	–	–	–	–	–	–	–	–	–	–	–	–	–
	29.90	29.78	29.55	29.30	29.04	28.78	28.51	28.26	27.99	~27.7	27.46	27.20	–	–
	29.87	29.77	29.55	29.32	29.09	28.84	28.58	28.32	28.06	27.79	~27.5	–	–	–
	33.24	32.88	32.45	32.04	31.64	31.26	30.89	30.53	30.17	29.83	29.50	29.19	28.85	28.56
Cu_6-ring, pz-4-*H*	–	–	–	–	–	–	–	–	–	–	–	–	–	–
	31.72	31.74	31.75	31.73	31.69	31.64	31.58	31.50	31.41	31.33	31.21	31.11	–	–
	32.31	32.32	32.32	32.28	32.22	32.15	32.06	31.99	31.84	31.72	31.61	–	–	–
	31.26	31.34	31.41	31.44	31.45	31.43	31.39	31.34	31.27	31.18	31.09	31.00	30.88	30.76
Cu_6-ring, pz-3,5-H_2	–	–	–	–	–	–	–	–	–	–	–	–	–	–
	28.52	28.52	28.53	28.52	28.49	28.45	~28.4	28.33	28.26	28.18	28.09	28.00	–	–
	29.01	29.02	29.02	29.00	28.95	28.88	28.82	28.74	28.64	28.57	~28.5	–	–	–
	28.24	28.30	28.36	28.40	28.43	28.47	~28.4	~28.3	~28.2	28.16	28.09	28.00	27.89	27.78
Cu_{12}-ring, pz-4-*H(9)*	–	–	–	–	–	–	–	–	–	–	–	–	–	–
	27.76	27.78	27.82	27.85	27.87	27.88	27.88	27.88	27.86	27.84	27.82	27.78	–	–
	27.88	27.91	27.95	27.98	28.01	28.02	28.03	28.02	28.01	27.99	27.96	27.93	–	–
	27.77	27.81	27.84	27.87	27.89	27.90	27.90	27.89	27.87	27.85	27.82	27.78	27.74	27.69
Cu_{12}-ring, pz-4-*H(6)*	–	–	–	–	–	–	–	–	–	–	–	–	–	–
	26.17	26.19	26.23	26.25	26.26	26.26	26.25	26.24	26.21	26.18	26.15	26.10	–	–
	26.25	26.27	26.30	26.33	26.34	26.35	26.34	26.33	26.31	26.28	26.24	26.19	–	–
	26.35	26.39	26.42	26.44	26.46	26.47	26.47	26.46	26.43	26.40	26.37	26.33	26.28	26.23
Cu_{12}-ring, pz-3,5-H_2*(9)*	–	–	–	–	–	–	–	–	–	–	–	–	–	–
	22.40	22.41	22.42	22.43	22.44	22.43	22.42	22.41	22.39	22.36	22.33	22.30	–	–
	22.48	22.49	22.51	22.52	22.53	22.53	22.52	22.50	22.48	22.46	22.43	22.39	–	–
	22.45	22.47	22.49	22.49	22.50	22.49	22.48	22.46	22.44	22.41	22.37	22.34	22.29	22.25
Cu_{12}-ring, pz-3,5-H_2*(6)*	–	–	–	–	–	–	–	–	–	–	–	–	–	–
	22.30	22.31	22.33	22.34	22.34	22.33	22.32	22.30	22.28	22.25	22.21	22.17	–	–
	22.37	22.38	22.40	22.41	22.41	22.41	22.40	22.38	22.36	22.33	22.30	22.25	–	–
	22.43	22.45	22.46	22.46	22.47	22.46	22.45	22.43	22.40	22.37	22.33	22.30	22.25	22.20
Cu_{12}-ring, O*H(6)*	–	–	–	–	–	–	–	–	–	–	–	–	–	–
	−26.42	−26.47	−26.56	−26.64	−26.69	−26.71	−26.72	−26.71	−26.77	−26.67	~−26.6	~−26.6	–	–
	−25.93	−25.97	−26.0	−26.11	−26.16	−26.20	−26.23	−26.26	−26.28	−26.30	~−26.3	~−26.3	–	–
	−29.65	−29.69	−29.72	−29.73	−29.72	−29.69	−29.64	−29.66	−29.50	−29.40	−29.30	−29.14	~−29.0	~−28.9
Cu_{12}-ring, O*H(9)*	–	–	–	–	–	–	–	–	–	–	–	–	–	–
	−36.14	−36.16	−36.16	−36.11	−36.03	−35.92	−35.81	−35.70	−35.63	−35.43	−35.25	−35.14	–	–
	−36.72	−36.74	−36.73	−36.66	−36.55	−36.41	−36.23	−36.03	−35.82	−35.65	−35.38	~−35.0	–	–
	−32.50	−32.77	−33.05	−33.25	−33.41	−33.53	−33.63	−33.76	−33.69	−33.71	−33.70	−33.66	−33.61	−33.55
Cu_6-ring, O*H*	–	–	–	–	–	–	–	–	–	–	–	–	–	–
	−41.33	−41.33	−41.30	−41.25	−41.16	−41.07	−40.98	−40.74	−40.56	−40.40	−40.20	−39.98	–	–
	−41.88	−41.82	−41.79	−41.72	−41.63	−41.51	−41.36	−41.19	−40.99	−40.79	−40.53	−40.32	–	–
	−41.25	−41.30	−41.34	−41.35	−41.35	−41.33	−41.28	−41.21	−41.11	−41.00	−40.86	−40.72	−40.59	−40.35
Cu_9-ring, O*H*	–	–	–	–	–	–	–	–	–	–	–	–	–	–
	−55.98	−55.86	−55.56	−55.18	−54.73	−54.25	−53.73	−53.18	−52.60	−52.04	−51.42	−50.81	–	–
	−51.31	−51.32	−51.26	−51.11	−50.90	−50.62	−50.28	−49.89	−49.47	−49.01	−48.51	−48.03	–	–
	−68.08	−66.96	−65.63	−64.38	−63.19	−62.04	−60.95	−59.91	−58.90	−57.90	−56.98	−56.07	−55.21	−54.36

Table S35 (continued).

NANOJAR	20 °C	30 °C	40 °C	50 °C	60 °C	70 °C	80 °C	90 °C	100 °C	110 °C	120 °C	130 °C	140 °C	150 °C
**Cu_{29} (7+13+9)**														
Cu_7-ring, pz-4-*H*	31.88	31.74	31.57	31.39	31.20	31.01	30.81	30.61	30.41	30.21	30.00	29.80	–	–
	30.80	30.81	30.81	30.79	30.76	30.72	30.68	30.62	30.55	30.49	30.40	~30.3	~30.2	~30.2
	31.06	31.06	31.06	31.04	31.01	30.97	30.91	30.84	30.77	30.69	30.61	–	–	–
	30.81	30.84	30.85	30.85	30.84	30.81	30.76	30.71	30.65	30.58	30.49	30.42	30.32	30.22
Cu_7-ring, pz-3,5-H_2	28.11	28.01	27.87	27.74	27.59	27.43	27.27	27.11	26.94	26.78	~26.6	~26.4	–	–
	27.29	27.29	27.28	27.27	27.25	27.22	27.18	27.13	27.08	~27.0	~26.9	26.86	26.73	~26.7
	27.56	27.56	27.56	27.53	27.48	27.50	27.43	27.37	27.31	27.24	27.17	–	–	–
	27.41	27.43	27.44	27.43	27.42	27.38	27.35	27.30	27.24	27.18	27.10	27.02	26.93	26.88
Cu_9-ring, pz-4-*H*	32.92	32.75	32.54	32.32	32.08	31.83	31.57	31.30	31.05	30.83	30.53	30.28	–	–
	30.75	30.67	30.53	30.38	30.22	30.07	29.92	29.75	29.60	29.46	29.28	29.13	28.97	28.83
	30.46	30.39	30.25	30.10	29.96	29.80	29.70	29.50	29.36	29.20	29.06	–	–	–
	33.87	33.68	33.44	33.19	32.95	32.70	32.46	32.23	31.99	31.76	31.51	31.29	31.05	30.81
Cu_9-ring, pz-3,5-H_2	29.00	28.86	28.70	28.52	28.33	28.13	27.93	27.72	27.51	27.29	27.08	26.86	–	–
	27.56	27.49	27.36	27.22	27.08	26.94	26.80	26.66	26.51	26.37	~26.1	~26.0	~25.9	~25.8
	27.45	27.39	27.25	27.12	26.99	26.84	26.72	26.56	26.42	26.28	~26.1	–	–	–
	29.79	29.62	29.42	29.21	29.00	28.79	28.59	~28.3	~28.2	27.96	~27.8	27.57	27.36	27.16
Cu_{13}-ring, pz-4-*H*	27.15	27.20	27.25	27.29	27.32	27.34	27.36	27.37	27.37	27.36	27.35	27.32	27.29	27.25
	27.16	27.19	27.25	27.30	27.34	27.37	27.39	27.40	27.40	27.40	27.39	27.37	27.34	27.31
	27.41	27.44	27.49	27.53	27.56	27.58	27.59	27.60	27.59	27.58	27.56	–	–	–
	27.12	27.18	27.24	27.30	27.34	27.38	27.40	27.42	27.43	27.42	27.41	27.40	27.37	27.33
Cu_{13}-ring, pz-3,5-H_2	21.96	21.99	22.02	22.04	22.06	22.07	22.07	22.07	22.06	22.04	22.02	21.99	21.96	21.92
	22.07	22.09	22.12	22.14	22.15	22.16	22.16	22.16	22.15	22.13	22.11	22.08	22.05	22.01
	22.26	22.27	22.30	22.31	22.32	22.32	22.32	22.31	22.29	22.27	22.24	–	–	–
	22.02	22.05	22.08	22.11	22.13	22.14	22.15	22.15	22.14	22.13	22.11	22.09	22.06	22.02
Cu_{13}-ring, O*H*	−29.43	−29.42	−29.41	−29.38	−29.35	~−29.3	–	–	–	–	–	–	–	–
	−28.92	−28.95	−28.99	−29.02	−29.04	~−29.0	~−29.0	~−29.0	~−29.1	−29.26	−29.37	−29.45	−29.53	−29.59
	−29.23	−29.25	−29.28	−29.29	−29.29	~−29.3	~−29.2	~−29.3	−29.25	−29.50	−29.57	–	–	–
	−29.32	−29.37	−29.42	−29.47	−29.53	−29.56	~−29.6	~−29.6	~−29.5	~−29.4	~−29.3	–	–	–
Cu_7-ring, O*H*	−37.67	−37.59	−37.51	−37.40	−37.27	–	–	–	–	–	–	–	–	–
	–	–	–	–	–	–	–	–	–	–	–	–	–	–
	−36.11	−36.09	−36.02	−35.93	−35.85	−35.78	−35.67	−35.52	−35.41	−35.27	−35.10	–	–	–
	−36.88	−36.83	−36.83	−36.65	−36.53	−36.42	−36.27	−36.13	−35.96	−35.81	−35.64	–	–	–
Cu_9-ring, O*H*	−43.00	−42.88	−42.71	−42.51	–	–	–	–	–	–	–	–	–	–
	−43.63	−43.40	−42.95	−42.50	−42.01	−41.57	−41.11	~−40.8	−40.35	−39.93	−39.48	−39.08	−38.69	−38.29
	−39.76	−39.58	−39.28	−38.95	−38.59	−38.27	−37.94	−37.62	−37.30	−36.99	−36.65	–	–	–
	−47.36	−46.88	−46.28	−45.68	−45.09	−44.51	−43.95	−43.40	−42.85	−42.31	−41.79	–	–	–
**Cu_{29} (8+13+8)**														
Cu_8-ring, pz-4-*H*	–	–	27.84	27.84	27.85	27.85	27.93	28.06	28.02	27.97	27.92	27.85	27.78	27.71
	–	–	~27.8	~27.8	~27.8	27.84	27.81	27.77	27.73	27.69	27.63	27.58	27.53	27.47
	–	–	27.71	27.86	27.84	27.81	27.78	27.74	27.70	27.66	27.61	27.54	27.50	27.45
	28.52	28.51	28.49	28.47	28.44	28.41	28.37	28.33	28.28	28.23	28.18	28.13	28.06	27.99
Cu_8-ring, pz-3,5-H_2	–	–	25.14	25.14	25.12	25.10	25.08	25.05	25.01	24.97	24.92	24.86	24.80	24.74
	25.13	25.11	25.09	25.07	25.05	25.02	24.99	24.95	24.91	24.87	24.82	24.76	24.71	24.65
	25.14	25.14	25.13	25.11	25.09	25.07	25.03	25.00	24.96	24.91	24.87	24.81	24.75	24.69
	25.58	25.58	25.56	25.54	25.52	25.49	25.45	25.41	25.37	25.32	25.27	25.22	25.15	25.08
Cu_{13}-ring, pz-4-*H*	26.18	26.23	26.28	26.29	26.35	26.41	26.47	26.51	26.55	26.57	26.59	26.59	26.59	26.58
	26.37	26.39	26.42	26.45	26.46	26.52	26.57	26.61	26.64	26.66	26.67	26.68	26.67	26.66
	26.35	26.39	26.48	26.55	26.62	26.68	26.71	26.76	26.78	26.80	26.80	26.80	26.79	26.77
	25.91	26.00	26.11	26.20	26.28	26.36	26.42	26.47	26.51	26.54	26.55	26.56	26.56	26.56
Cu_{13}-ring, pz-3,5-H_2	21.20	21.26	21.31	21.36	21.40	21.43	21.45	21.47	21.48	21.49	21.48	21.47	21.45	21.44
	21.36	21.39	21.44	21.48	21.52	21.55	21.57	21.59	21.59	21.60	21.59	21.58	21.56	21.53
	21.49	21.52	21.57	21.61	21.64	21.67	21.68	21.69	21.70	21.69	21.68	21.67	21.64	21.61
	21.21	21.27	21.33	21.39	21.43	21.47	21.50	21.53	21.54	21.55	21.54	21.54	21.52	21.50
Cu_{13}-ring, O*H*	−27.74	−27.88	−28.02	−28.28	−28.48	−28.66	−28.82	−28.98	−29.16	−29.31	–	–	–	–
	−31.73	−31.44	−30.88	−30.33	−29.78	~−29.2	~−29.0	~−29.0	~−29.1	−29.04	−28.99	−28.92	−28.84	−28.79
	−31.72	−31.42	−30.85	−30.29	−29.74	~−29.3	~−29.2	~−29.3	−29.38	−29.17	−29.07	−29.02	–	–
	−26.70	−26.91	−27.14	−27.34	−27.53	−27.74	−27.92	−28.10	−28.25	−28.39	−28.54	−28.65	−28.78	−28.86
Cu_8-ring, O*H*	–	–	–	–	–	–	–	–	–	–	–	–	–	–
	–	–	–	–	–	–	–	–	–	–	–	–	–	–
	−40.93	−40.59	−39.87	~−39.2	~−38.6	~−38.3	~−37.9	~−37.6	−37.30	−36.99	−36.62	−36.38	–	–
	−33.61	−33.34	−33.05	−32.81	−32.53	−32.29	−32.05	−31.83	−31.63	−31.44	−31.27	−31.10	−30.96	−30.81

Table S35 (continued).

NANOJAR	20 °C	30 °C	40 °C	50 °C	60 °C	70 °C	80 °C	90 °C	100 °C	110 °C	120 °C	130 °C	140 °C	150 °C
**Cu_{31} (8+14+9)**														
Cu_9-ring, pz-4-*H*	–<br>–	–<br>–	–<br>~33.3	~32.9<br>~33.0	32.67<br>32.73	32.44<br>32.48	32.20<br>32.24	31.97<br>32.01	31.75<br>31.79	31.52<br>31.56	31.30<br>31.34	31.10<br>31.13	30.89<br>30.91	30.69<br>–
	–	–	–	–	31.63	31.43	31.22	31.04	30.87	30.70	30.53	30.36	30.21	30.05
	–	–	–	–	~31.1	~30.9	30.77	30.61	30.46	30.31	30.17	30.01	29.86	29.71
	–	–	33.86	33.64	33.43	33.21	33.00	32.78	32.56	32.34	32.13	31.92	–	–
Cu_9-ring, pz-3,5-H_2	–<br>–	–<br>–	–<br>~28.7	~28.4<br>~28.5	~28.3<br>~28.4	~28.2<br>28.22	28.09<br>28.04	27.82<br>27.86	27.65<br>27.68	27.47<br>27.50	27.30<br>27.32	27.13<br>27.15	26.95<br>26.97	26.79<br>–
	–	–	–	–	27.71	27.57	27.42	27.28	27.13	27.00	26.85	26.71	26.57	26.44
	–	–	–	–	~27.4	27.31	27.18	27.05	26.93	26.80	26.68	26.54	26.41	26.28
	–	–	~29.3	~29.1	~29.0	~28.8	~28.6	~28.3	~28.1	~28.0	~27.8	27.57	–	–
Cu_8-ring, pz-4-*H*	–<br>–	–<br>–	–<br>~29.8	–<br>29.81	29.77<br>29.80	29.75<br>29.79	29.73<br>29.77	29.70<br>29.74	29.66<br>29.70	29.61<br>29.65	29.55<br>29.59	29.49<br>29.53	29.42<br>29.45	29.35<br>–
	–	–	–	–	29.73	29.72	29.69	29.65	29.60	29.55	29.49	29.42	29.35	29.28
	–	–	–	–	29.75	29.73	29.66	29.66	29.61	29.55	29.49	29.42	29.35	29.27
	–	–	~30.9	~30.85	30.75	30.66	30.58	~30.5	~30.4	30.30	30.20	30.11	–	–
Cu_8-ring, pz-3,5-H_2	–<br>–	–<br>–	–<br>~26.3	–<br>26.28	26.23<br>26.27	26.23<br>26.26	26.21<br>26.24	26.19<br>26.21	26.15<br>26.18	26.11<br>26.13	26.05<br>26.08	25.99<br>26.02	25.92<br>25.95	25.86<br>–
	–	–	–	–	26.27	26.26	26.24	26.20	26.16	26.11	26.06	26.00	25.93	25.86
	–	–	–	–	26.34	~26.3	26.30	26.26	26.22	26.17	26.12	26.05	25.98	25.91
	–	–	~27.1	27.09	27.02	26.96	26.89	26.82	26.74	~26.6	~26.5	~26.5	–	–
Cu_{14}-ring, pz-4-*H*	28.30<br>–	28.36<br>28.36	28.40<br>28.40	28.44<br>28.43	28.46<br>28.46	28.48<br>28.47	28.48<br>28.48	28.48<br>28.48	28.46<br>28.46	28.44<br>28.44	28.41<br>28.41	28.37<br>28.37	28.33<br>28.32	28.27<br>–
	~28.2	~28.2	28.26	28.31	28.34	28.37	28.39	28.40	28.39	28.38	28.36	28.33	28.29	28.24
	28.34	28.36	28.42	28.46	28.50	28.52	28.53	28.53	28.53	28.51	28.48	28.45	28.40	28.35
	28.24	28.30	28.36	28.41	28.44	28.46	28.48	28.48	28.47	28.45	28.43	28.40	–	–
Cu_{14}-ring, pz-3,5-H_2	22.60<br>22.60	22.62<br>22.62	22.63<br>22.64	22.64<br>22.64	22.64<br>22.64	22.63<br>22.63	22.62<br>22.62	22.60<br>22.60	22.57<br>22.57	22.54<br>22.54	22.50<br>22.50	22.45<br>22.45	22.40<br>22.40	22.35<br>–
	22.49	22.52	22.55	22.57	22.58	22.58	22.57	22.56	22.54	22.51	22.48	22.44	22.39	22.35
	22.61	22.63	22.65	22.66	22.67	22.67	22.66	22.64	22.62	22.59	22.55	22.51	22.46	22.41
	22.66	22.68	22.70	22.71	22.71	22.70	22.69	22.67	22.65	22.61	22.57	22.53	–	–
Cu_{14}-ring, O*H*	−26.53<br>~−27.4	−26.61<br>~−27.3	−26.70<br>−27.20	−26.77<br>−27.13	−26.83<br>−27.07	−26.89<br>−27.00	−26.89<br>−26.94	−26.87<br>−26.86	−26.82<br>−26.78	−26.73<br>−26.69	−26.66<br>−26.62	−26.51<br>−26.49	−26.37<br>–	−26.23<br>–
	–	–	–	–	~−26.7	~−26.7	~−26.6	−26.46	−26.39	−26.32	−26.22	−26.12	−26.02	−25.91
	–	–	–	–	~−26.6	~−26.6	−26.55	−26.49	−26.41	−26.33	−26.22	−26.16	−26.04	−25.92
	–	–	–	–	–	–	–	–	–	–	–	–	–	–
Cu_8-ring, O*H*	–<br>–	–<br>–	–<br>–	–<br>–	–<br>~−34.8	–<br>~−34.7	–<br>~−34.6	–<br>~−34.5	–<br>~−34.4	–<br>–	–<br>–	–<br>–	–<br>–	–<br>–
	–	–	–	–	−33.33	−32.99	−32.74	−32.52	−32.32	−32.15	−31.99	−31.83	−31.78	−31.63
	–	–	–	–	~−33.3	−33.06	−32.87	−32.68	−32.50	−32.33	−32.15	−32.06	−31.94	−31.82
	–	–	–	–	–	–	–	–	–	–	–	–	–	–
Cu_9-ring, O*H*	–<br>–	–<br>–	–<br>~−42.3	–<br>~−41.7	–<br>−41.12	–<br>−40.56	–<br>−39.95	–<br>−39.41	–<br>−38.91	–<br>−38.46	–<br>–	–<br>–	–<br>–	–<br>–
	–	–	–	–	~−40.0	−39.33	−38.58	−37.99	−37.44	−36.95	−36.42	−35.95	−35.51	−35.08
	–	–	–	–	–	–	−34.93	−34.53	−34.18	−33.84	−33.48	−33.21	−32.91	−32.63
	–	–	–	–	–	–	–	–	–	–	–	–	–	–

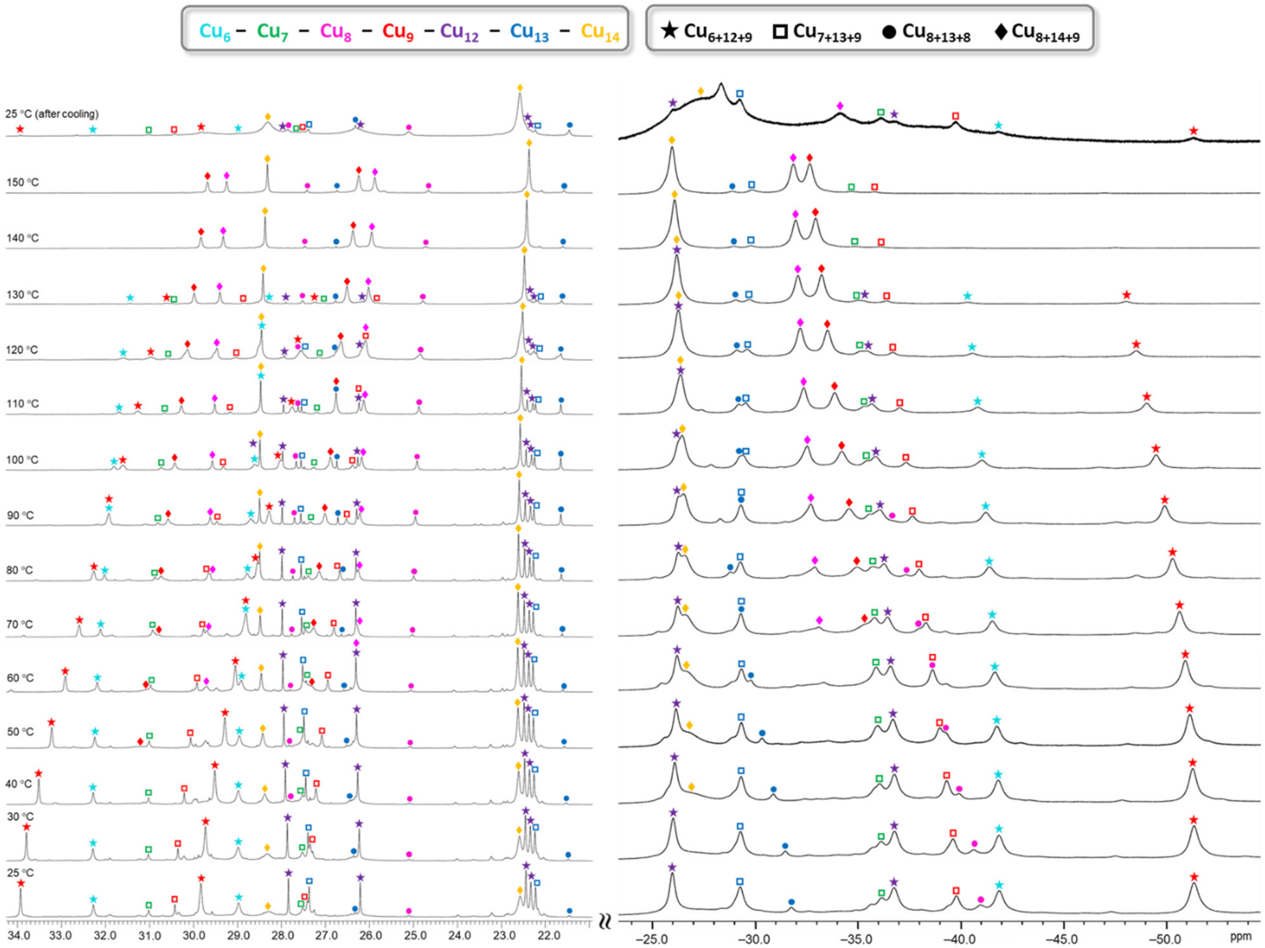

Figure S39. Variable-temperature ^1H NMR spectra of the Cu_nFPO_3 (n = 27−29, 31) nanojar mixture in DMSO-d_6, showing pyrazolate and OH proton signals in the 21 to 34 and −25 to −53 ppm windows, respectively. The temperatures shown are the target temperatures of the probe.

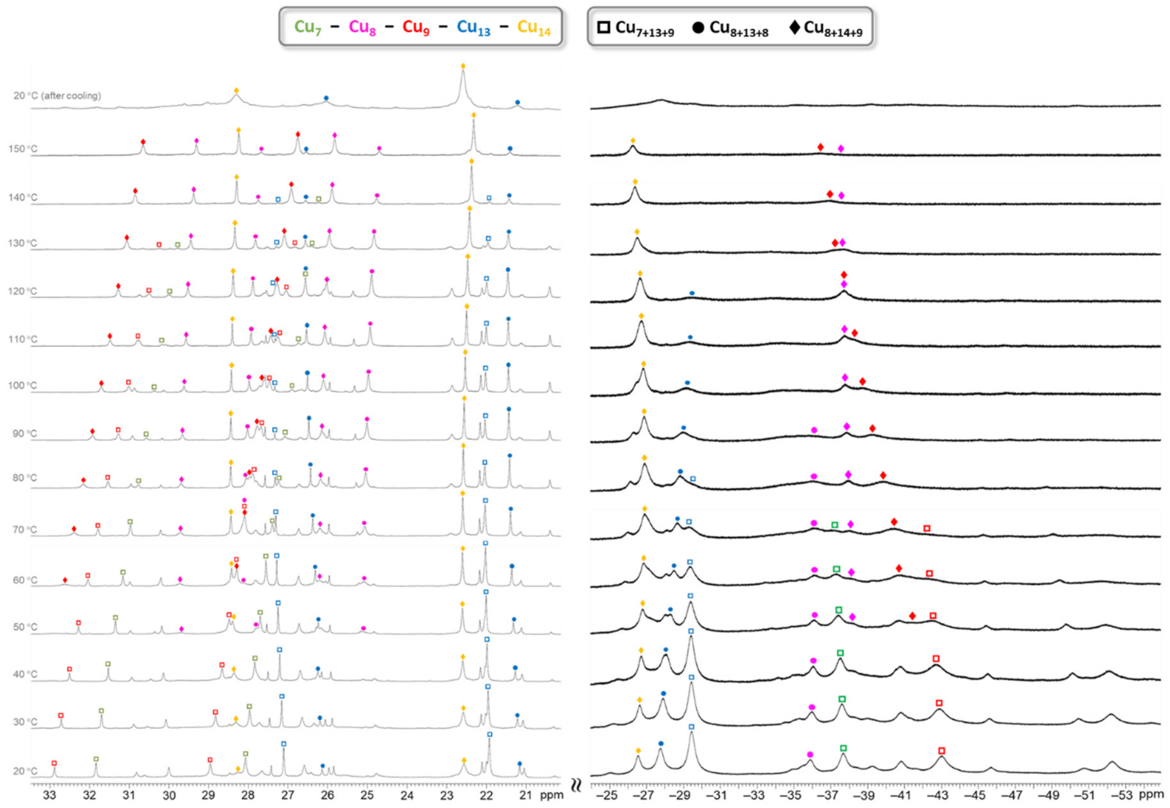

Figure S40. Variable-temperature ^{1}H NMR spectra of the Cu_nHPO_4 (n = 29, 31) nanojar mixture in DMSO-d_6, showing pyrazolate and OH proton signals in the 21 to 33 and −25 to −53 ppm windows, respectively. The temperatures shown are the target temperatures of the probe.

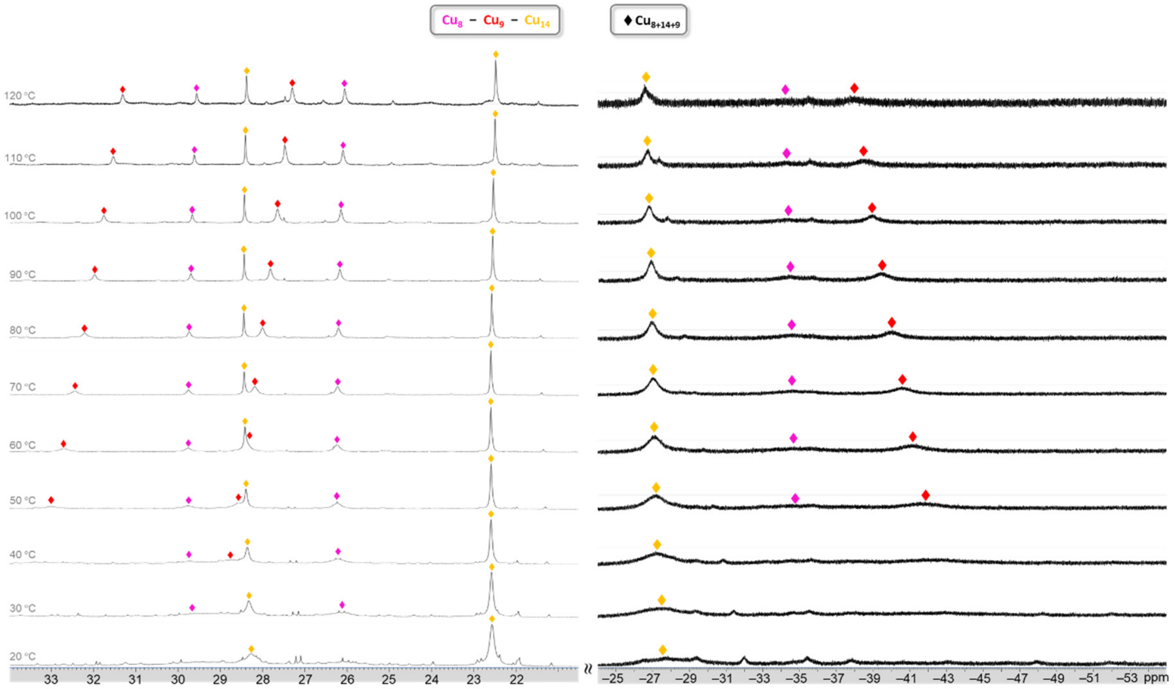

Figure S41. Variable-temperature ^{1}H NMR spectra of the almost pure $Cu_{31}HPO_4$ nanojar in DMSO-d_6, showing pyrazolate and OH proton signals in the 21 to 33 and −25 to −53 ppm windows, respectively. The temperatures shown are the target temperatures of the probe.

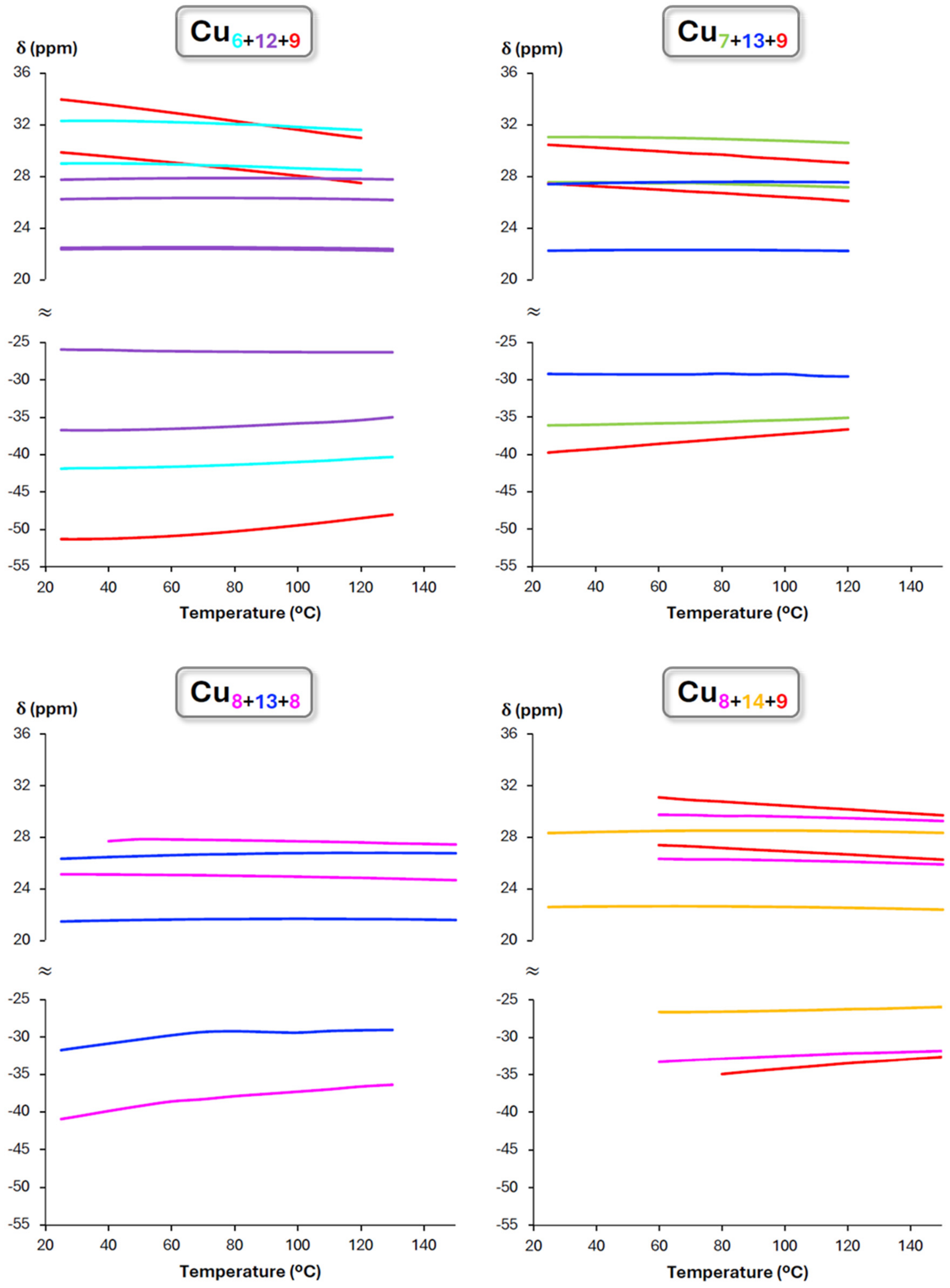

Figure S42. Temperature-dependent variation of the ^{1}H NMR chemical shifts (δ) in DMSO-d_6 of the different Cu_x ring protons in **Cu_nFPO_3**.

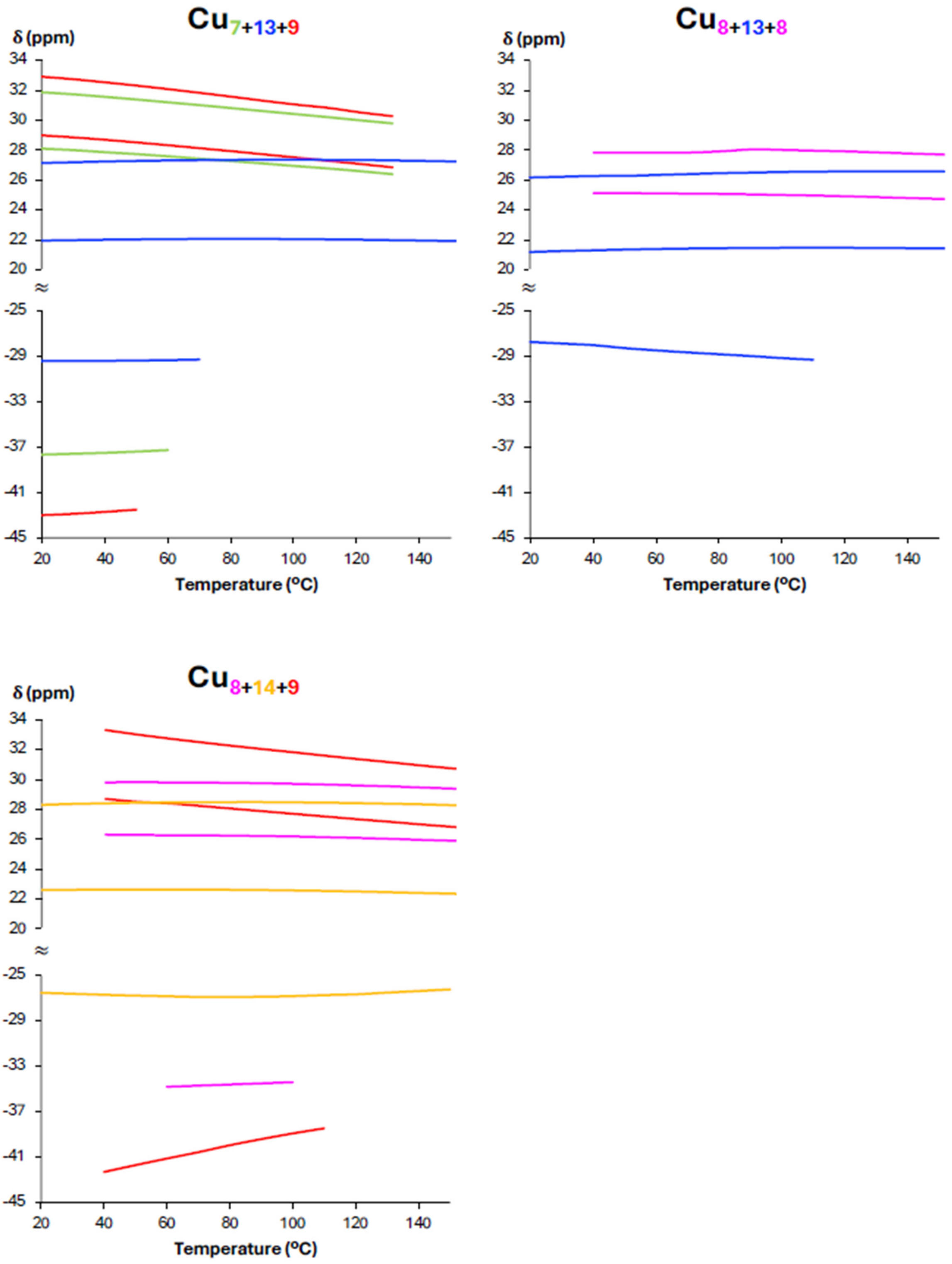

Figure S43. Temperature-dependent variation of the ^{1}H NMR chemical shifts (δ) in DMSO-d_6 of the different Cu_x ring protons in $\mathbf{Cu_nHPO_4}$.

5. REFERENCES

[1] Al Isawi, W. A.; Zeller, M.; Mezei, G. Tribenzyl(methyl)ammonium: A Versatile Counterion for the Crystallization of Nanojars with Incarcerated Selenite and Phosphite Ions and Tethered Pyrazole Ligands. *Cryst. Growth Des.* **2022**, *22*, 1398–1411. DOI: 10.1021/acs.cgd.1c01361

[2] Al Isawi, W. A.; Zeller, M.; Mezei, G. Isomorphous but No Dead Ringer: Contrasting the Supramolecular Binding of Tetrafluoroberyllate and Sulfate Ions by Nanojars. *Cryst. Growth Des.* **2023**, *23*, 1676–1688. DOI: 10.1021/acs.cgd.2c01261

[3] Singh, P.; Zeller, M.; Mezei, G. Supramolecular Binding of Phosphonate Dianions by Nanojars and Nanojar Clamshells. *Inorg. Chem.* **2024**, *63*, 14216−14230. DOI: 10.1021/acs.inorgchem.4c02386

[4] Yang, L.; Powell, D. R.; Houser, R. P. Structural Variation in Copper(I) Complexes with Pyridylmethylamide Ligands: Structural Analysis with a New Four-Coordinate Geometry Index, τ_4. *Dalton Trans.* **2007**, 955–964. DOI: 10.1039/B617136B

[5] Addison, A. W.; Rao, T. N.; Reedijk, J.; van Rijn, J.; Verschoor, G. C. Synthesis, Structure, and Spectroscopic Properties of Copper(II) Compounds Containing Nitrogen–Sulphur Donor Ligands; The Crystal and Molecular Structure of Aqua[1,7-bis(*N*-methylbenzimidazol-2′-yl)-2,6-dithiaheptane]copper(II) Perchlorate. *Dalton Trans.* **1984**, 1349–1356. DOI: 10.1039/DT9840001349
